# Exploring the Phe-Gly Dipeptide-Derived Piperazinone Scaffold in the Search for Antagonists of the Thrombin Receptor PAR1

**DOI:** 10.3390/molecules19044814

**Published:** 2014-04-16

**Authors:** Ángel M. Valdivielso, M. Teresa García-López, Marta Gutiérrez-Rodríguez, Rosario Herranz

**Affiliations:** Instituto de Química Médica (CSIC), Juan de la Cierva 3, 28006 Madrid, Spain; E-Mails: angel@iqm.csic.es (Á.M.V.); iqmgl37@iqm.csic.es (M.T.G.-L.); mgutierrez@iqm.csic.es (M.G.-R.)

**Keywords:** peptidomimetics, regioselectivity, piperazinones, platelet antiaggregant activity, PAR1 antagonists

## Abstract

A series of Phe-Gly dipeptide-derived piperazinones containing an aromatic urea moiety and a basic amino acid has been synthesized and evaluated as inhibitors of human platelet aggregation induced by the PAR1 agonist SFLLRN and as cytotoxic agents in human cancer cells. The synthetic strategy involves coupling of a protected basic amino acid benzyl amide to 1,2- and 1,2,4-substituted-piperazinone derivatives, through a carbonylmethyl group at the N_1-_position, followed by formation of an aromatic urea at the exocyclic moiety linked at the C_2_ position of the piperazine ring and removal of protecting groups. None of the compounds showed activity in the biological evaluation.

## 1. Introduction

Most cellular effects of thrombin are mediated by activation of the protease-activated receptor 1 (PAR1) [1,2]. This receptor is mainly expressed in platelets, where its activation induces aggregation. Therefore, PAR1 is considered a therapeutic target in cardiovascular diseases [1,3,4,5], whose inactivation could inhibit platelet aggregation without affecting thrombin’s role in the coagulation cascade [3,6,7]. In addition, numerous studies have shown that PAR1 is overexpressed in invasive and metastatic tumors and that its expression levels directly correlate with the degree of invasiveness of the cancer [8,9,10,11,12,13]. Based on these facts, this receptor is starting to be also considered a promising target for cancer therapy, particularly in the search of angiogenesis inhibitors [2].

Activation of PAR1 by thrombin involves the proteolytic cleavage of the N-terminal exodomain between Arg^41^ and Ser^42^. This cleavage unveils the recognition sequence SFLLRN that acts as a tethered ligand, auto-activating the receptor [14]. The binding of this tethered ligand is followed by the coupling of the receptor to heterotrimeric G proteins and activation of signal transduction. This particular intramolecular activation mechanism makes PAR1 a target particularly difficult to address. 

**Figure 1:**
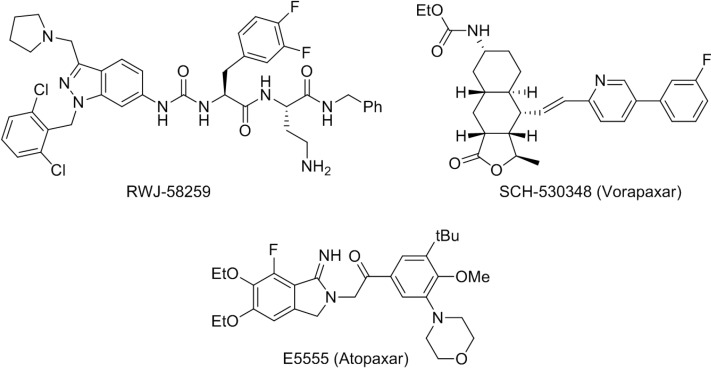
Reference PAR1 antagonists in pharmacological studies and/or advanced clinical development.

The first potent PAR1 antagonists were SFLLRN-based peptidomimetic ureas, represented by the optimized antagonist RWJ-58259 (Figure 1) [7], which is considered a standard reference in pharmacological studies on PAR1 receptor [15]. Later, a few series of antagonists have been discovered by HTS of diverse libraries of non-peptide small molecules [7,16]. Up to now, only two of these PAR1 antagonists are in advanced clinical development for the treatment of patients with acute coronary syndrome, SCH-530348 (named vorapaxar, in phase III clinical trials) [17] and E-5555 (named atopaxar, in phase II clinical trials) [18] (Figure 1).

**Figure 2:**
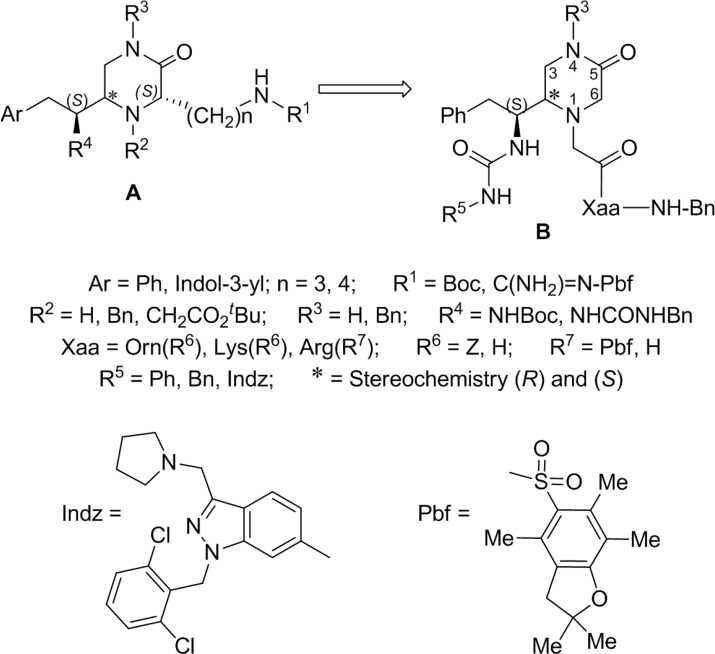
Piperazinone derivatives proposed as PAR1 antagonists.

Taking as reference the peptidomimetic antagonist RWJ-58259 we initiated a project directed to the search of new PAR1 antagonists using a diversity oriented synthesis (DOS) strategy. To this aim, we planned the synthesis of diverse small directed libraries of different scaffolds able to assemble, at least, one or two aromatic groups and one or two basic groups at variable distances and orientations [19]. Among the scaffolds, we focused our attention on the piperazine ring, since this system is recognized as a privileged scaffold, due to its recurrent presence in biological active compounds [20,21]. Firstly, we synthesized the series of 1,2,4,6-tetrasusbtituted-piperazinone derivatives of general formula **A** (Figure 2). Some of these derivatives showed moderate antagonist activity [22]. Trying to improve this activity and to establish structure-activity relationships, we have synthesized and report herein the analogues **B**, where the basic amino acid side chain has been moved from the piperazine C_6_ position to the N_1_. Now, the indazole moiety of the PAR1 antagonist RWJ-58259 has also been included among the selected arylureido groups at the piperazine C_2_-substituent. The new piperazinone derivatives **B** have been evaluated as human PAR1 antagonists in a platelet aggregation assay and as cytotoxic agents in human cancer cell lines.

## 2. Results and Discussion

**Scheme 1:**
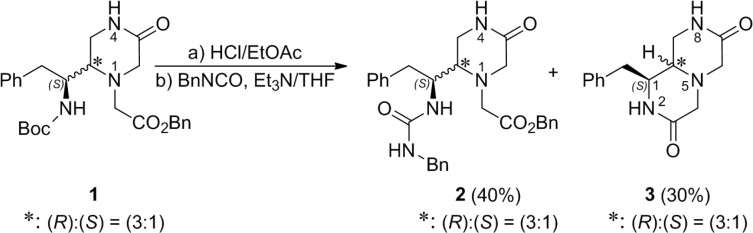
Synthesis of the ureas **2** and the 1*H*-pyrazino[1,2-*a*]pyrazines **3**.

Two alternative retrosynthetic routes were considered for the building of the desired piperazinones derivatives **B** from the starting 1-(benzyloxycarbonyl)methyl-piperazinones **1** [23]. These routes differ in the order of incorporation of the basic amino acid and the urea moieties. Firstly, we attempted the formation of the urea at the exocyclic 1-amino-2-phenylethyl moiety, before coupling the basic amino acid residue Xaa. However, as shown in Scheme 1, the Boc removal from the (3:1) epimeric mixture of N_4_-unsubstituted-piperazinones **1**, by treatment with a 3 N solution of HCl in EtOAc, followed by reaction with benzyl isocyanate in the presence of Et_3_N, led to the corresponding epimeric mixture of ureas **2** in 40% yield, along with 30% of the 1*H*-pyrazino[1,2-*a*]pyrazines **3**. These bicyclic derivatives resulted from the nucleophilic attack of the exocyclic amine, generated by the Boc removal, at the (benzyloxycarbonyl)methyl group in the N_1_-position. To minimize this cyclization, both the Boc removal and the urea formation were carried out at 0 °C with an excess of benzyl isocyanate (2 equiv.) to accelerate the urea formation. Nevertheless, in none of these attempts was the yield of the ureas **2** improved significantly.

**Scheme 2:**
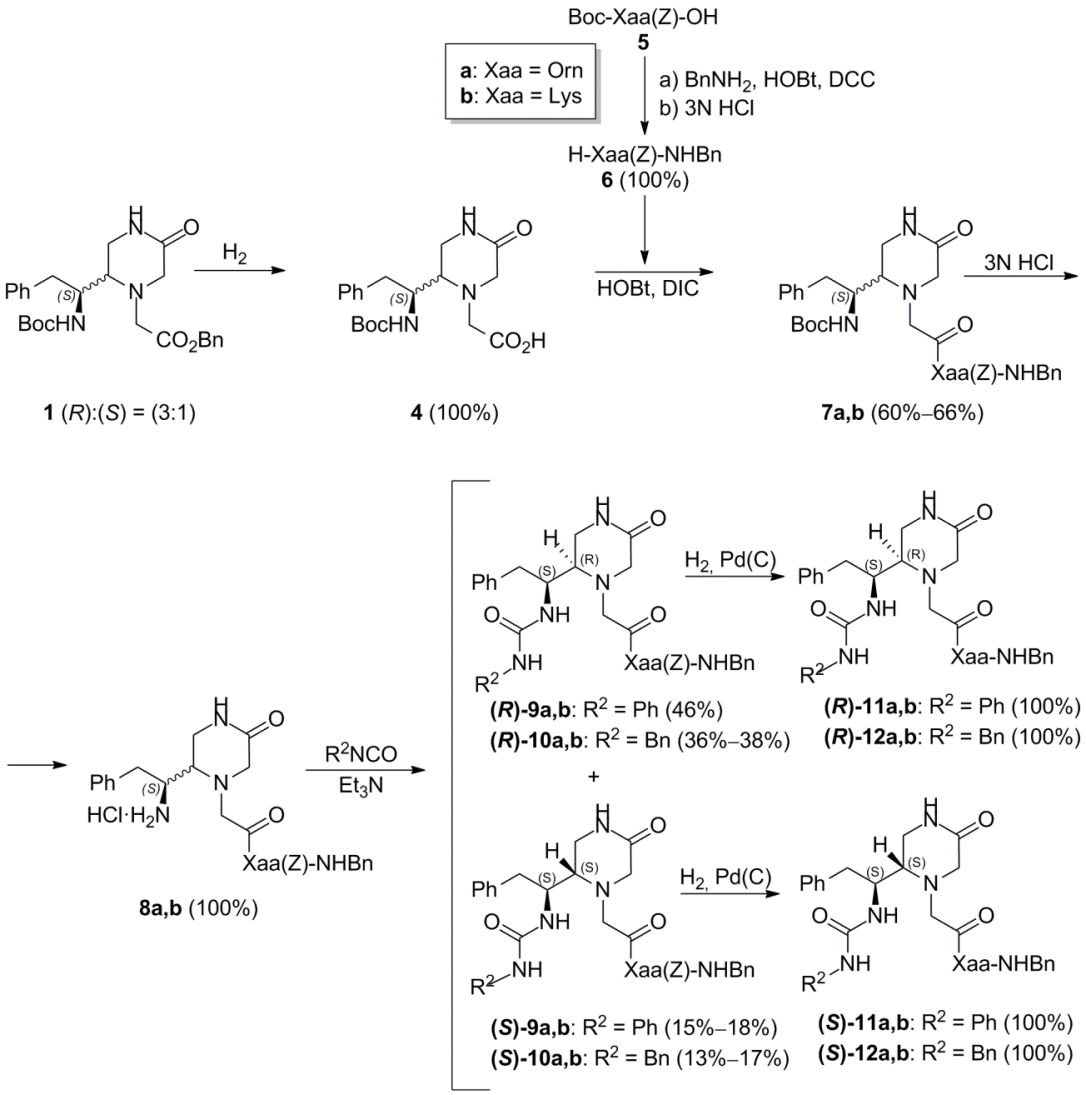
Synthesis of the 4-unsubstituted-piperazinone derivatives **11a**,**b** and **12a**,**b**.

To avoid the cyclization, we decided to incorporate the basic amino acid prior to the urea formation. As shown in Scheme 2, the Pd (C) catalyzed hydrogenolysis of the benzyl ester of **1**, followed by coupling with H-Orn(Z)-NHBn (**6a**) and H-Lys(Z)-NHBn (**6b**), using diisopropylcarbodiimide (DIC) and 1-hydroxybenzotriazol (HOBt) as coupling agents, provided the corresponding epimeric mixtures **7a**,**b** in 60%–70% yield. The subsequent Boc removal, followed by reaction with phenyl or benzyl isocyanate in the presence of Et_3_N, gave the respective epimeric mixtures of ureas **9a**,**b** and **10a**,**b**, which were chromatographically resolved into their respective (*R*)- and (*S*)-epimers. Finally, the removal of the Z protecting group from the basic side chain, by Pd(C)-catalysed hydrogenolysis, provided the corresponding deprotected pseudotripeptides **11a**,**b** and **12a**,**b**. The (3:1) epimer ratio remained constant throughout the synthetic route.

**Scheme 3:**
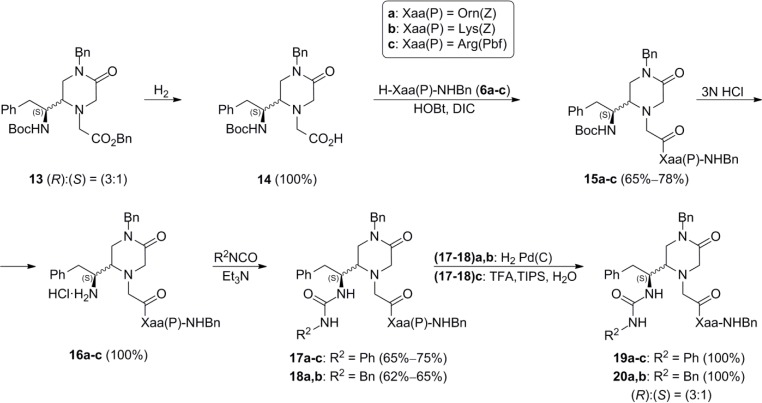
Synthesis of the N_4_-benzyl-piperazinone derivatives **19a**–**c** and **20a**,**b**.

In view of the good results in the synthesis of the 4-unsubstituted-piperazinone derivatives **11a**,**b** and **12a**,**b**, a parallel synthetic scheme was applied to the synthesis of the 4-benzyl-piperazinone derivatives **19a**–**c** and **20a**,**b** from the (3:1) epimeric mixture of 4-benzyl-piperazinones **13** [23] (Scheme 3). Based on the biological results of the previous library **A**, besides ornithine (**a**) and lysine (**b**), arginine (**c**) was also included in this series. The Pbf protection was used for the guanidino group of the side chain of this amino acid. This protection was removed in the last step of the synthesis by treatment with a 90% solution of TFA in H_2_O in the presence of triisopropylsilane (TIPS). The final arylureido derivatives **19a**–**c** and **20a**,**b** were obtained in 39%–58% overall yields from **13**, as (3:1) epimeric mixtures that could not be separated in none of their synthetic steps. 

**Scheme 4:**
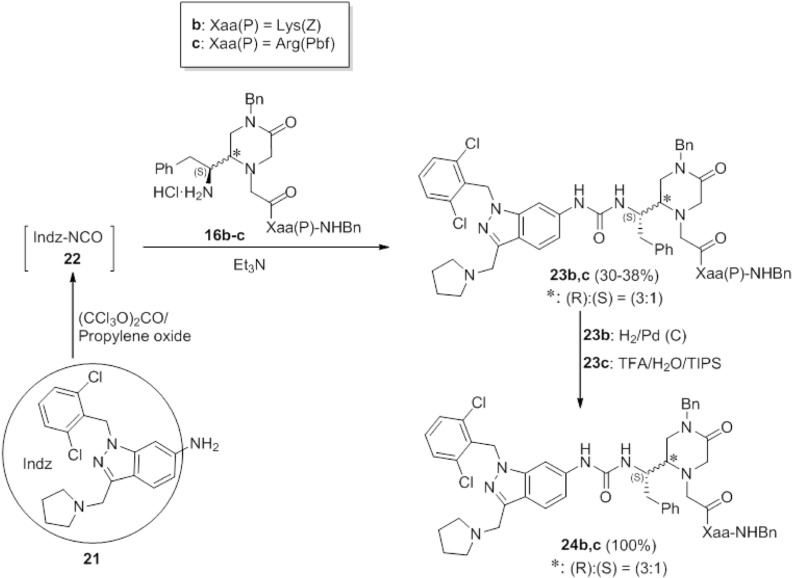
Synthesis of the RWJ-58259 analogues **24b**,**c**.

The 4-benzyl-piperazinones **16b**,**c** were also used for the preparation of indazol-6-yl-ureido derivatives analogues of the reference antagonist RWJ-58259. These analogues were prepared according to our procedure developed for the synthesis of RWJ-58259 [24], which involves the *in situ* formation of the isocyanate **22** (Scheme 4), by reaction of the corresponding 6-amino-indazole **21** with triphosgene in the presence of propylene oxide as HCl acceptor, followed by reaction with the epimeric mixture of the 4-benzyl-piperazinones **16b**,**c**. The Z- or Pbf-removal, by hydrogenolysis and TFA treatment, respectively, provided the proposed ureas **24b**,**c** as (3:1) epimeric mixtures that, like the analogues **19** and **20**, could not be resolved at any of their synthetic steps.

To evaluate the PAR1 antagonist activity, all new compounds were screened as inhibitors of human platelet aggregation induced by a 30 μM concentration of the PAR1 agonist SFLLRN [22]. The antagonist RWJ-58259 was used as a reference. At 10 μM concentration, this antagonist inhibited 98% the platelet aggregation. However, none of the new compounds displayed significant activity at 0.1 mg/mL (≈150 μM). In the structural comparison of the inactive deprotected indazole-derived ureas **24b**,**c** with the potent peptidomimetic urea PAR1 antagonists, to which the reference antagonist RWJ-58259 belongs [25], the main difference is localized at the linkage between the aromatic and the basic amino acids. Thus, the peptide bond of RWJ-58259 is replaced by the piperazinone ring and an additional Gly residue in **24b**,**c**. The results show that this replacement is completely detrimental for PAR1 antagonist activity.

In a HTS of antitumor agents, none of the compound showed cytotoxicity on three representative human cancer cell lines, such as breast (MDA-MB-231), lung (A549), and colon (HT-29).

## 3. Experimental

### 3.1. General

All reagents were of commercial quality. Solvents were dried and purified by standard methods. Analytical TLC was performed on aluminum sheets coated with a 0.2 mm layer of silica gel 60 F_254_. Silica gel 60 (230–400 mesh) was used for flash chromatography. Analytical HPLC was performed on a Sunfire C_18_ (4.6 × 150 mm, 3.5 μm) column, with a flow rate of 1 mL/min, and using a tunable UV detector set at 214 nm. 10%–100% gradient of CH_3_CN (solvent A) in 0.05% of TFA in H_2_O (solvent B) in 30 min was used as mobile phase. ^1^H-NMR spectra were recorded at 300 or 400 MHz, using TMS as reference, and ^13^C-NMR spectra were recorded at 75 or 100 MHz. The NMR spectra assignment was based on COSY, HSQC, and HMBC spectra. ESI-MS spectra were performed, in positive mode, using MeOH as solvent. MW experiments were carried out in a EmrysTM Synthesizer MW reactor (Biotage AB, surface IR sensor). Elemental analyses were obtained on a CH-O-RAOID apparatus. Optical rotations were determined in a Perkin Elmer 141 polarimeter.

### 3.2. Synthesis of Benzyl 2-[(2*RS*)-[(1*S*)-(3-benzylureido)-2-phenylethyl]-5-oxopiperazin-1-yl]acetate (2) and (1*S*,9a*RS*)- 1-benzyl-3,7-dioxooctahydro-1*H*-pyrazino[1,2-*a*]pyrazine (**3**)

The epimeric mixture of piperazinones **1** [23] [(*R*:*S*) = (3:1)] (500 mg, 1.07 mmol) was dissolved in a solution of HCl in EtOAc (3.4 N, 20 mL) and the mixture was stirred at room temperature for 30 min. Afterwards, the solvent was evaporated to dryness, the residue was dissolved in CH_3_CN/H_2_O (1:3, 8 mL) and the solution was lyophilized. Benzyl isocyanate (199 μL, 1.61 mmol) and Et_3_N (224 μL, 1.61 mmol) were added to a solution of the lyophilized powder in THF (40 mL) and the mixture was stirred for 1 h. Afterwards, the solvent was removed under low pressure and the residue was dissolved in CH_2_Cl_2_ (60 mL). The solution was washed with H_2_O (2 × 10 mL), brine (10 mL), dried over Na_2_SO_4_ and evaporated to dryness. The residue was purified by flash chromatography, with 0%–5% MeOH gradient in EtOAc as mobile phase, to afford the epimeric mixture of ureas **2** [(*R*:*S*) = (3:1)] as a foam (215 mg, 40%), along with the 1*H*-pyrazino[1,2-*a*]pyrazines **3** [23] (83 mg, 30%).

*Benzyl 2-[(2*RS*)-[(1*S*)-(3-benzylureido)-2-phenylethyl]-5-oxopiperazin-1-yl]acetate* (**2**). HPLC *t*_R_: 20.02 min [(***R***)-**2**] and 21.24 min [(***S***)-**2**]; ^1^H-NMR (300 MHz, CDCl_3_). (***R***)-**2** *δ* (ppm): 2.56 (dd, 1H, *J* = 10.5 and 13.5 Hz, *CH_2_*-Ph), 2.82 (dd, 1H, *J* = 4 and 13.5 Hz, *CH_2_*-Ph), 3.06 (dt, 1H, *J* = 4.5 and 9 Hz, 2-H), 3.24 (m, 1H, 3-H), 3.26 (d, *J* = 18 Hz, 6-H), 3.43 (s, 2H, *CH_2_*CO_2_Bn), 3.46 (d, 1H, *J* = 18 Hz, 6-H), 3.60 (m, 1H, 3-H), 3.89 (m, 1H, 2-*CH*), 4.32 [d, 2H, *J* = 5.5 Hz, CH_2_ (NH*Bn*)], 5.04 [m, 1H, *NH*Bn], 5.10 [s, 2H, CH_2_ (CO_2_*Bn*)], 5.45 (m, 1H, 4-H), 5.70 (m, 1H, 2-CH*NH*), 7.14–7.35 (m, 15H, Ar). (***S***)-**2** *δ* (ppm): 2.56 (m, 1H, *CH_2_*-Ph), 2.82 (m, 1H, *CH_2_*-Ph), 3.06 (m, 1H, 2-H), 3.24 (m, 1H, 3-H), 3.50 (d, *J* = 17.5 Hz, 6-H), 3.43 (s, 2H, *CH_2_*CO_2_Bn), 3.58 (d, 1H, *J* = 17.5 Hz, 6-H), 3.60 (m, 1H, 3-H), 3.89 (m, 1H, 2-*CH*), 4.32 [m, 2H, CH_2_ (NH*Bn*)], 5.04 (m, 1H, *NH*Bn), 5.10 [s, 2H, CH_2_ (CO_2_*Bn*)], 5.45 (m, 1H, 4-H), 5.70 (m, 1H, 2-CH*NH*), 7.14–7.35 (m, 15H, Ar); ^13^C-NMR (75 MHz, CDCl_3_). *(**R**)*-**2** *δ* (ppm): 36.7 [C_3_], 37.4 [*CH_2_*-Ph], 44.5 [CH_2_ (NH*Bn*)], 51.3 [C_6_ and *CH_2_*CO_2_Bn], 53.0 [C_2_-*CH*], 58.0 [C_2_], 66.9 [CH_2_ (CO_2_*Bn*)], 127.3, 127.4, 127.6, 128.4, 128.5, 128.6, 128.7, 129.0, 129.2 [15CH (Ar)], 135.2 [C (CO_2_*Bn*)], 136.0 [C (Ph)], 139.3 [C (NH*Bn*)], 158.2 [CO (Urea)], 169.2 [C_5_], 171.0 [CO_2_]. *(**S**)*-**2** *δ* (ppm): 36.7 [C_3_], 37.4 [*CH_2_*-Ph], 44.5 [CH_2_ (NH*Bn*)], 51.3 [*CH_2_*CO_2_Bn], 53.0 [C_2_-*CH*], 55.6 [C_6_], 58.0 [C_2_], 66.9 [CH_2_ (CO_2_*Bn*)], 127.3, 127.4, 127.6, 128.4, 128.5, 128.6, 128.7, 129.0, 129.2 [CH (Ar)], 135.2 [C (CO_2_*Bn*)], 136.0 [C (Ph)], 139.3 [C (NH*Bn*)], 158.2 [CO (Urea)], 169.2 [C_5_], 171.0 [CO_2_]; ES-MS *m/z* 501.2 [M+1]^+^; C_29_H_32_N_4_O_5_ (%): C: 69.58, H: 6.44, N: 11.19. Found (%): C: 69.73, H: 6.32, N: 11.45.

### 3.3. General Procedure for the Synthesis of the Piperazinone-Derived Acids **4** and **14**

Pd(C) (10%) was added to a solution of the corresponding epimeric mixture of piperazinones **1** [23] or **13** [23] [(*R*:*S*) = (3:1)] (1.00 mmol) in MeOH (50 mL) and the mixture was hydrogenated at 1 atm of H_2_ at room temperature for 1 h. Afterwards, the reaction mixture was filtered and the solvent was evaporated under reduced pressure to obtain the epimeric mixture of the corresponding acids **4** or **14** [(*R*:*S*) = (3:1)].

*2-[(2*RS*)-[(1*S*)-((*tert*-Butoxycarbonyl)amino)-2-phenyl-ethyl]-5-oxopiperazin-1-yl] acetic acid* (**4**). Foam (377.4 mg, 100%); HPLC *t*_R_: 13.99 min [(***R***)-**4**] and 13.39 min [(***S***)-**4**]; ^1^H-NMR (500 MHz, DMSO-*d_6_*). (***R***)-**4** *δ* (ppm): 1.24 (s, 9H, Boc), 2.56 (dd, 1H, *J* = 10 and 10.5 Hz, *CH_2_*-Ph), 2.88 (m, 1H, 2-H), 2.97 (dd, 1H, *J* = 3.5 and 10.5 Hz, *CH_2_*-Ph), 3.19 (m, 2H, 3-H), 3.30 (m, 1H, 6-H), 3.47 (d, 3H, *J* = 17 Hz, 6-H and *CH_2_*CO_2_H), 3.80 (m, 1H, 2-*CH*), 6.80 (d, 1H, *J* = 9.5 Hz, *NH*Boc), 7.02–7.36 (m, 5H, Ph), 7.76 (s, 1H, 4-H). (***S***)-**4** *δ* (ppm): 1.25 (s, 9H, Boc), 2.47 (m, 1H, *CH_2_*-Ph), 2.82 (dd, 1H, *J* = 2 and 13.5 Hz, *CH_2_*-Ph), 2.92 (m, 1H, 2-H), 3.19 (m, 2H, 3-H), 3.31 (m, 1H, 6-H), 3.42 (m, 1H, 6-H), 3.47 (m, 2H, *CH_2_*CO_2_H), 3.80 (m, 1H, 2-*CH*), 6.89 (d, 1H, *J* = 9.5 Hz, *NH*Boc), 7.02–7.36 (m, 5H, Ph), 7.75 (s, 1H, 4-H); ^13^C-NMR (125 MHz, DMSO-*d_6_*). *(**R**)*-**4** *δ* (ppm): 28.6 [3CH3 (Boc)], 37.9 [*CH_2_*-Ph], 38.9 [C_3_], 51.8 [C_2_-*CH*], 53.3 [*CH_2_*CO_2_H], 54.0 [C_6_], 58.4 [C_2_], 78.1 [C (Boc)], 126.2, 128.4, 129.7 [5CH (Ph)], 139.6 [C (Ph)], 155.8 [CO (Boc)], 168.3 [C_5_], 172.6 [CO_2_]. *(**S**)*-**4** *δ* (ppm): 28.6 [3CH3 (Boc)], 35.7 [*CH_2_*-Ph], 38.8 [C_3_], 52.0 [C_2_-*CH*], 53.0[C_6_], 54.1 [*CH_2_*CO_2_H], 59.0 [C_2_], 78.0 [C (Boc)], 126.3, 128.4, 129.4 [5CH (Ph)], 139.8 [C (Ph)], 155.5 [CO (Boc)], 169.2 [C_5_], 172.4 [CO_2_]; ES-MS *m/z* 378.0 [M+1]^+^; C_19_H_27_N_3_O_5_ (%): C: 60.46, H: 7.21, N: 11.13. Found (%): C: 60.60, H: 7.02, N: 11.25.

### 3.4. General Procedure for the Synthesis of the Piperazinone-Derived Pseudotripeptides **7a**,**b**

HOBt (136 mg, 1.00 mmol), DIC (309 μL, 2.00 mmol) and a solution of the corresponding benzylamides H-Orn(Boc)-NHBn (**6a**) [26] and H-Lys(Boc)-NHBn (**6b**) [27] (1.50 mmol) in dry DMF (4 mL) were added to a solution of the epimeric mixture of the piperazinone-derived acid **4** (1.00 mmol) in dry CH_2_Cl_2_ (16 mL) and stirred for 24 h. Afterwards, the solvent was removed under reduced pressure and the residue was dissolved in EtOAc (100 mL). This solution was washed with a solution of 10% citric acid (2 × 20 mL), a saturated solution of NaHCO_3_ (2 × 20 mL) and brine (20 mL), dried over Na_2_SO_4_, and evaporated to dryness. The residue was purified by flash chromatography, with 1%–10% MeOH gradient in CH_2_Cl_2_ as mobile phase to afford the corresponding epimeric mixture of piperazinone derivatives **7a**,**b** [(*R*:*S*) = (3:1)].

N*-[2-[(2*RS*)-[(1*S*)-((*tert*-Butoxycarbonyl)amino)-2-phenylethyl]-5-oxopiperazin-1-yl]acetyl]-Orn(Z)-NHBn* (**7a**). Foam (429 mg, 60%); HPLC *t*_R_: 21.07 min; ^1^H-NMR (400 MHz, CDCl_3_) (***R***)-**7a** *δ* (ppm): 1.31 (s, 9H, Boc), 1.52 (m, 2H, γ-H), 1.67 (m, 1H, β-H), 1.84 (m, 1H, β-H), 2.82 (m, 1H, *CH_2_*-Ph), 2.86 (m, 1H, 2-H), 3.02 (m, 1H, *CH_2_*-Ph), 3.12 (m, 1H, δ-H), 3.30 (m, 1H, *CH_2_*CO), 3.34 (m, 1H, *CH_2_*CO), 3.36 (m, 3H, 3-H and 6-H), 3.42 (m, 1H, δ-H), 3.44 (m, 1H, 6-H), 4.04 (m, 1H, 2-*CH*), 4.34 [dd, 1H, *J* = 5.5 and 15 Hz, CH_2_ (NH*Bn*)], 4.42 [dd, 1H, *J* = 5.5 and 15 Hz, CH_2_ (NH*Bn*)], 4.70 (m, 3H, α-H and *NH*Boc), 4.83 [d, 1H, *J* = 12 Hz, CH_2_ (Z)], 4.93 [d, 1H, *J* = 12 Hz, CH_2_ (Z)], 5.12 (t, 1H, *J* = 6 Hz, *NH*Z), 6.38 (m, 1H, 4-H), 7.11–7.39 (m, 16H, Ar and *NH*Bn), 7.79 (d, 1H, *J* = 8 Hz, α-NH). (***S***)-**7a** *δ* (ppm): 1.31 (s, 9H, Boc), 1.64(m, 1H, β-H), 1.86 (m, 1H, β-H), 3.26 (m, 1H, *CH_2_*CO), 3.32 (m, 1H, 6-H), 3.38 (m, 1H, *CH_2_*CO), 3.46 (m, 1H, 6-H), 3.94 (m, 1H, 2-*CH*), 4.35, 4.47 [m, 2H, CH_2_ (NH*Bn*)], 4.82 [m, 1H, CH_2_ (Z)], 4.95 [m, 1H, CH_2_ (Z)], 5.04 (m, 1H, *NH*Z), 6.62 (m, 1H, 4-H), 7.11–7.39 (m, 16H, Ar and *NH*Bn), 7.79 (d, 1H, *J* = 8 Hz, α-NH); ^13^C-NMR (100 MHz, CDCl_3_) (***R***)-**7a** *δ* (ppm): 26.3 [C_γ_], 28.2 [3CH3 (Boc)], 30.3 [C_β_], 37.6 [*CH_2_*-Ph], 39.4 [C_3_], 39.7 [C_δ_], 43.5 [CH_2_ (NH*Bn*)], 51.3 [C_2_-*CH*], 51.5 [C_α_], 54.0 [C_6_], 55.7 [*CH_2_*CO], 58.8 [C_2_], 66.6 [CH_2_ (Z)], 79.9 [C (Boc)], 126.7, 127.4, 127.7, 127.9, 128.1, 128.4, 128.6, 129.3 [15CH (Ar)], 136.4 [C (Ph)], 137.0 [C (Z)], 138.0 [C (NH*Bn*)], 155.6 [CO (Boc)], 157.1 [CO (Z)], 168.9 [C_5_], 169.7 [CO], 171.5 [α-CONH]. (***S***)-**7a** *δ* (ppm): 28.2 [3CH_3_ (Boc)], 43.5 [CH_2_ (NH*Bn*)], 66.6 [CH_2_ (Z)], 79.9 [C (Boc)], 126.6, 127.4, 127.6, 127.9, 128.0, 128.4, 128.6, 129.3 [15CH (Ar)], 136.4 [C (Ph)], 137.0 [C (Z)], 138.0 [C (NH*Bn*)], 155.6 [CO (Boc)], 157.1 [CO (Z)], 171.5 [α-CONH]; ES-MS *m/z* 715.6 [M+1]^+^; C_39_H_50_N_6_O_7_ (%): C: 65.53, H: 7.05, N: 11.76. Found (%): C: 65.71, H: 6.98, N: 11.89.

N*-[2-[(2*RS*)-[(1*S*)-((*tert*-Butoxycarbonyl)amino)-2-phenylethyl]-5-oxopiperazin-1-yl]acetyl]-Lys(Z)-NHBn* (**7b**). Foam (481 mg, 66%); HPLC *t*_R_: 21.56 min [(***R***)-**7b**] and 21.41 min [(***S***)-**7b**]; ^1^H-NMR (400 MHz, CDCl_3_) (***R***)-**7b** *δ* (ppm): 1.27 (s, 9H, Boc), 1.29 (m, 2H, γ-H), 1.44 (m, 2H, δ-H), 1.65 (m, 1H, β-H), 1.83 (m, 1H, β-H), 2.77 (m, 1H, *CH_2_*-Ph), 2.78 (m, 1H, 2-H), 2.95 (d, 1H, *J* = 10 Hz, *CH_2_*-Ph), 3.05 (m, 2H, ε-H), 3.20 (m, 1H, 6-H), 3.22 (m, 2H, *CH_2_*CO), 3.23 (m, 2H, 3-H), 3.35 (m, 1H, 6-H), 4.00 (m, 1H, 2-*CH*), 4.34 [dd, 1H, *J* = 8 and 15 Hz, CH_2_ (NH*Bn*)], 4.40 [dd, 1H, *J* = 8 and 15 Hz, CH_2_ (NH*Bn*)], 4.48 (m, 1H, α-H), 4.81 (d, 1H, *J* = 8 Hz, *NH*Boc), 5.03 [m, 2H, CH_2_ (Z)], 5.25 (m, 1H, *NH*Z), 6.85 (m, 1H, 4-H), 7.08–7.40 (m, 16H, Ar and *NH*Bn), 7.79 (d, 1H, *J* = 8 Hz, α-NH). (***S***)-**7b** *δ* (ppm): 1.27 (s, 9H, Boc), 1.29 (m, 2H, γ-H), 1.44 (m, 2H, δ-H), 1.65 (m, 1H, β-H), 1.83 (m, 1H, β-H), 2.78 (m, 1H, 2-H), 3.10 (m, 1H, 6-H), 3.15 (m, 2H, *CH_2_*CO), 3.23 (m, 2H, 3-H), 3.36 (m, 1H, 6-H), 3.90 (m, 1H, 2-*CH*), 4.28, 4.42 [m, 2H, CH_2_ (NH*Bn*)], 4.46 (m, 1H, α-H), 5.03 [m, 2H, CH_2_ (Z)], 5.25 (m, 1H, *NH*Z), 6.77 (m, 1H, 4-H), 7.08–7.40 (m, 16H, Ar and *NH*Bn), 7.73 (d, 1H, *J* = 8 Hz, α-NH); ^13^C-NMR (100 MHz, CDCl_3_) (***R***)-**7b** *δ* (ppm): 22.6 [C_γ_], 28.1 [3CH_3_ (Boc)], 29.2 [C_δ_], 32.1 [C_β_], 37.6 [*CH_2_*-Ph], 39.5 [C_3_], 40.5 [C_ε_], 43.4 [CH_2_ (NH*Bn*)], 51.6 [C_2_-*CH*], 52.7 [C_α_], 53.9 [C_6_], 55.7 [*CH_2_*CO], 58.7 [C_2_], 66.4 [CH_2_ (Z)], 79.6 [C (Boc)], 126.7, 127.3, 127.6, 128.0, 128.5, 128.6, 129.2 [15CH (Ar)], 136.6 [C (Ph)], 137.1 [C (Z)], 138.1 [C (NH*Bn*)], 155.6 [CO (Boc)], 156.5 [CO (Z)], 169.4 [C_5_], 169.8 [CO], 171.5 [α-CONH]. (***S***)-**7b** *δ* (ppm): 22.6 [C_γ_], 28.1 [3CH_3_ (Boc)], 29.6 [C_δ_], 31.8 [C_β_], 39.4 [C_6_], 43.4 [CH_2_ (NH*Bn*)], 51.9 [C_2_-*CH*], 52.7 [C_α_], 53.9 [C_6_], 55.7 [*CH_2_*CO], 59.0 [C_2_], 66.4 [CH_2_ (Z)], 79.7 [C (Boc)], 126.6, 127.3, 127.6, 128.0, 128.5, 128.6, 129.2 [15CH (Ar)], 136.6 [C (Ph)], 137.1 [C (Z)], 138.1 [C (NH*Bn*)], 155.8 [CO (Boc)], 156.5 [CO (Z)], 169.4 [C_5_], 170.3 [CO], 171.6 [α-CONH]; ES-MS *m/z* 729.3 [M+1]^+^; C_40_H_52_N_6_O_7_ (%): C: 65.91, H: 7.19, N: 11.53. Found (%): C: 65.72, H: 7.40, N: 11.68.

### 3.5. General Procedure for the N-Boc Removal in **7a**,**b**. Synthesis of the Hydrochlorides **8a**,**b**

The epimeric corresponding epimeric mixture of piperazine derivatives **7a**,**b** [(*R*:*S*) = (3:1)] (0.60 mmol) was dissolved in 3.4 N HCl in EtOAc (15 mL) and the mixture was stirred at room temperature for 30 min. Afterwards, the solvent was evaporated to dryness, the residue was dissolved in CH_3_CN/H_2_O (1:3, 5 mL), and the solution was lyophilized. The desired epimeric mixture of hydrochlorides [(*R*:*S*) = (3:1)] was obtained quantitatively.

N*-[2-[(2*RS*)-[(1*S*)-Amino-2-phenylethyl]-5-oxopiperazin-1-yl]acetyl]-Orn(Z)-NHBn hydrochloride* (**8a**). Amorphous solid (391 mg, 100%); HPLC *t*_R_: 14.86 min; ^1^H-NMR (400 MHz, DMSO-*d_6_*) (***R***)-**8a** *δ* (ppm): 1.30 (m, 2H, γ-H), 1.57 (m, 1H, β-H), 1.72 (m, 1H, β-H), 2.59 (dd, 1H, *J* = 9 and 14 Hz, *CH_2_*-Ph), 2.86 (dd, 1H, *J* = 6.5 and 14 Hz, *CH_2_*-Ph and 2-H), 2.95 (m, 4H, δ-H and 3-H), 3.02 (d, 1H, *J* = 18 Hz, 6-H), 3.23 (d, 1H, *J* = 16.5 Hz, *CH_2_*CO), 3.33 (d, 1H, *J* = 16.5 Hz, *CH_2_*CO), 3.52 (d, 1H, *J* = 18 Hz, 3-H), 4.16 (m, 1H, 2-*CH*), 4.28 (m, 1H, α-H), 4.40 [m, 2H, CH_2_ (NH*Bn*)], 4.97 [m, 2H, CH_2_ (Z)], 7.15–7.40 (m, 16H, Ar and *NH*Z), 7.63 (m, 1H, 4-H), 8.03 (m, 3H, NH_2_·HCl), 8.14 (d, 1H, *J* = 8.5 Hz, α-NH), 8.51 (t, 1H, *J* = 6 Hz, *NH*Bn). (***S***)-**8a** *δ* (ppm): 1.30 (m, 2H, γ-H), 1.57 (m, 1H, β-H), 1.72 (m, 1H, β-H), 4.32 (m, 1H, α-H), 4.40 [m, 2H, CH_2_ (NH*Bn*)], 4.98 [m, 2H, CH_2_ (Z)], 7.15–7.40 (m, 16H, Ar and *NH*Z), 8.03 (m, 3H, NH_2_·HCl), 8.20 (m, 1H, α-NH), 8.57 (m, 1H, *NH*Bn); ^13^C-NMR (100 MHz, DMSO-*d_6_*) (***R***)-**8a** *δ* (ppm): 26.1 [C_γ_], 28.8 [C_β_], 34.9 [C_3_], 36.0 [*CH_2_*-Ph], 40.5 [C_δ_], 42.0 [CH_2_ (NH*Bn*)], 49.7 [C_6_], 50.2 [C_2_-*CH*], 52.4 [C_α_], 55.9 [C_2_], 58.5 [*CH_2_*CO], 65.1 [CH_2_ (Z)], 126.7, 127.0, 127.7, 128.3, 128.4, 128.6, 128.8 [15CH (Ar)], 136.6 [C (Ph)], 137.2 [C (Z)], 139.4 [C (NH*Bn*)], 156.1 [CO (Z)], 168.2 [C_5_], 169.7 [CO], 171.6 [α-CONH]. (***S***)-**8a** *δ* (ppm): 26.0 [C_γ_], 28.8 [C_β_], 42.0 [CH_2_ (NH*Bn*)], 52.3 [C_α_], 65.1 [CH_2_ (Z)], 126.6, 127.1, 127.8, 128.3, 128.4, 128.6, 128.8 [15CH (Ar)], 136.6 [C (Ph)], 137.2 [C (Z)], 139.4 [C (NH*Bn*)], 156.1 [CO (Z)], 171.6 [α-CONH]; ES-MS *m/z* 615.8 [M−Cl]^+^; C_34_H_42_N_6_O_5_·HCl (%): C: 62.71, H: 6.66, N: 12.91. Found (%): C: 62.53, H: 6.78, N: 12.98.

N*-[2-[(2*RS*)-[(1*S*)-Amino-2-phenylethyl]-5-oxopiperazin-1-yl]acetyl]-Lys(Z)-NHBn hydrochloride* (**8b**). Amorphous solid (399 mg, 100%); HPLC *t*_R_: 15.25 min; ^1^H-NMR (400 MHz, DMSO-*d_6_*) (***R***)-**8b** *δ* (ppm): 1.22 (m, 2H, γ-H), 1.35 (m, 2H, δ-H), 1.66 (m, 2H, β-H), 2.59 (dd, 1H, *J* = 9 and 14 Hz, *CH_2_*-Ph), 2.84 (m, 1H, *CH_2_*-Ph), 2.88 (m, 1H, 2-H), 3.94 (m, 2H, ε-H), 3.01 (m, 2H, 3-H), 3.02 (d, 1H, *J* = 18 Hz, 6-H), 3.23 (d, 1H, *J* = 16.5 Hz, *CH_2_*CO), 3.37 (d, 1H, *J* = 16.5 Hz, *CH_2_*CO), 3.54 (d, 1H, *J* = 18 Hz, 6-H), 4.18 (m, 1H, 2-*CH*), 4.24 [m, 2H, CH_2_ (NH*Bn*)], 4.26 (m, 1H, α-H), 4.98 [m, 2H, CH_2_ (Z)], 7.14–7.41 (m, 16H, Ar and *NH*Z), 7.63 (m, 1H, 4-H), 8.07 (m, 3H, NH_2_·HCl), 8.15 (d, 1H, *J* = 8.5 Hz, α-NH), 8.55 (t, 1H, *J* = 6 Hz, *NH*Bn). (***S***)-**8b** *δ* (ppm): 1.22 (m, 2H, γ-H), 1.35 (m, 2H, δ-H), 1.66 (m, 2H, β-H), 3.01 (m, 1H, 3-H), 3.37 (m, 1H, *CH_2_*CO), 3.38 (m, 1H, *CH_2_*CO), 3.55 (m, 1H, 6-H), 4.24 [m, 2H, CH_2_ (NH*Bn*)], 4.26 (m, 1H, α-H), 4.98 [m, 2H, CH_2_ (Z)], 7.14–7.41 (m, 16H, Ar and *NH*Z), 8.07 (m, 3H, NH_2_·HCl), 8.23 (m, 1H, α-NH), 8.58 (m, 1H, *NH*Bn); ^13^C-NMR (100 MHz, DMSO-*d_6_*) (***R***)-**8b** *δ* (ppm): 22.9 [C_γ_], 29.0 [C_δ_], 31.2 [C_β_], 34.8 [C_3_], 36.0 [*CH_2_*-Ph], 39.5 [C_ε_], 42.0 [CH_2_ (NH*Bn*)], 49.6 [C_6_], 51.6 [C_2_-*CH*], 52.8 [C_α_], 55.9 [C_2_], 58.5 [*CH_2_*CO], 65.1 [CH_2_ (Z)], 126.6, 127.0, 127.7, 128.2, 128.3, 128.6, 128.8 [15CH (Ar)], 136.6 [C (Ph)], 137.3 [C (Z)], 139.4 [C (NH*Bn*)], 156.1 [CO (Z)], 168.2 [C_5_], 169.6 [CO], 171.7 [α-CONH]. (***S***)-**8b** *δ* (ppm): 22.9 [C_γ_], 29.0 [C_δ_], 31.2 [C_β_], 42.0 [CH_2_ (NH*Bn*)], 49.6 [C_6_], 58.5 [*CH_2_*CO], 65.1 [CH_2_ (Z)], 126.6, 127.0, 127.7, 128.2, 128.3, 128.6, 128.8 [15CH (Ar)], 136.6 [C (Ph)], 137.3 [C (Z)], 139.4 [C (NH*Bn*)], 156.1 [CO (Z)], 171.7 [α-CONH]; ES-MS *m/z* 629.7 [M-Cl]^+^; C_35_H_44_N_6_O_5_·HCl (%): C: 63.19, H: 6.82, N: 12.63. Found (%): C: 63.02, H: 6.94, N: 12.74.

### 3.6. General Procedure for the Synthesis of the Piperazinone-Derived Ureas **9a**,**b** and **10a**,**b**

Et_3_N (168 μL, 1.20 mmol) and the corresponding isocyanate (phenyl or benzyl isocyanate) (1.20 mmol) were added to a solution of the corresponding hydrochloride **8a**,**b** (0.60 mmol) in dry THF (30 mL). After stirring for 1 h at room temperature, the solvent was removed under reduced pressure and the residue was dissolved in CH_2_Cl_2_ (100 mL). The solution was washed with H_2_O (2 × 20 mL), brine (20 mL), dried over Na_2_SO_4_, and evaporated to dryness. The residue was purified by flash chromatography using 1%–8% MeOH gradient in EtOAc as mobile phase. The respective (*R*)- and (*S*)-epimers were resolved in this purification. The purified compounds were dissolved in CH_3_CN/H_2_O (1:2, 2 mL) and the solution was lyophilized, to afford the desired ureas **9a**,**b** and **10a**,**b**.

N*-[2-[5-Oxo-(2*R*)-[2-phenyl-(1*S*)-(3-phenylureido)ethyl]piperazin-1-yl]acetyl]-Orn(Z)-NHBn* [(***R***)-**9a**]. Amorphous solid (176 mg, 46%); 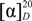 = −0.1 (*c* 1, MeOH); HPLC *t*_R_: 20.30 min; ^1^H-NMR (400 MHz, CDCl_3_) *δ* (ppm): 1.30 (m, 2H, γ-H), 1.50 (m, 1H, β-H), 1.70 (m, 1H, β-H), 2.82 (m, 1H, *CH_2_*-Ph), 2.83 (m, 1H, δ-H), 2.84 (m, 1H, 2-H), 2.88 (m, 1H, *CH_2_*-Ph), 3.03 (m, 1H, *CH_2_*CO), 3.14 (m, 1H, 6-H), 3.35 (m, 1H, 6-H), 3.37 (m, 1H, 3-H), 3.24 (m, 1H, δ-H), 3.44 (m, 1H, *CH_2_*CO), 4.20 (m, 1H, 3-H), 4.25 (m, 1H, 2-*CH*), 4.26 [m, 1H, CH_2_ (NH*Bn*)], 4.36 [m, 1H, CH_2_ (NH*Bn*)], 4.60 (m, 1H, α-H), 4.82 [d, 1H, *J* = 12.5 Hz, CH_2_ (Z)], 4.91 [d, 1H, *J* = 12.5 Hz, CH_2_ (Z)], 5.25 (m, 1H, *NH*Z), 5.97 (m, 1H, 4-H), 6.12 (m, 1H, 2-CH*NH*), 6.91–7.35 (m, 20H, Ar), 7.46 (m, 1H, *NH*Bn), 7.65 [m, 1H, *NH*Ph], 7.88 (m, 1H, α-NH); ^13^C-NMR (100 MHz, CDCl_3_) *δ* (ppm): 26.7 [C_γ_], 29.9 [C_β_], 37.9 [*CH_2_*-Ph], 39.7 [C_δ_], 40.1 [C_3_], 43.7 [CH_2_ (NH*Bn*)], 51.6 [C_2_-*CH*], 52.0 [C_α_], 54.9 [C_6_], 57.8 [*CH_2_*CO], 59.6 [C_2_], 67.0 [CH_2_ (Z)], 116.7, 119.7, 123.0, 127.0, 127.9, 120.4, 128.8, 129.0, 129.3 [20CH (Ar)], 134.3 [C (Ph)], 136.1 [C (Z)], 137.9 [C (NH*Bn*)], 139.5 [C (NH*Ph*)], 157.2 [CO (Z) and CO (Urea)], 168.7 [C_5_], 170.0 [CO], 171.1 [α-CONH]; ES-MS *m/z* 734.4 [M+1]^+^; C_41_H_47_N_7_O_6_ (%): C: 67.10, H: 6.46, N: 13.36. Found (%): C: 67.28, H: 6.59, N: 13.19.

N*-[2-[5-Oxo-(2*S*)-[2-phenyl-(1*S*)-(3-phenylureido)ethyl]piperazin-1-yl]acetyl]-Orn(Z)-NHBn* [(*S*)-**9a**]. Amorphous solid (79 mg, 18%); 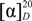 = +9.2 (*c* 1.5, MeOH); *t*_R_: 21.41 min; ^1^H-NMR (500 MHz, CDCl_3_) *δ* (ppm): 1.32 (m, 1H, γ-H), 1.40 (m, 1H, γ-H), 1.53 (m, 1H, β-H), 1.72 (m, 1H, β-H), 2.54 (dd, 1H, *J* = 11 and 13.5 Hz, *CH_2_*-Ph), 2.90 (dd, 1H, *J* = 4 and 13.5 Hz, *CH_2_*-Ph), 2.92 (m, 1H, δ-H), 3.08 (m, 1H, 5-H), 3.10 (m, 1H, 3-H), 3.14 (m, 1H, 6-H), 3.32 (m, 2H, *CH_2_*CO), 3.35 (m, 1H, δ-H), 3.54 (d, 1H, *J* = 18 Hz, 6-H), 3.92 (m, 1H, 2-*CH*), 3.95 (m, 1H, 3-H), 4.28 [dd, 1H, *J* = 5 and 15 Hz, CH_2_ (NH*Bn*)], 4.44 [dd, 1H, *J* = 6 and 15 Hz, CH_2_ (NH*Bn*)], 4.60 [d, 1H, *J* = 13 Hz, CH_2_ (Z)], 4.71 (m, 1H, α-H), 4.83 [d, 1H, *J* = 13 Hz, CH_2_ (Z)], 4.95 (m, 1H, *NH*Z), 5.67 (m, 1H, 4-H), 5.94 (d, 1H, *J* = 6 Hz, 2-CH*NH*), 6.83–7.35 (m, 20H, Ar), 7.53 (m, 1H, *NH*Bn), 7.93 [m, 1H, *NH*Ph], 7.97 (d, 1H, *J* = 8.5 Hz, α-NH); ^13^C-NMR (125 MHz, CDCl_3_) *δ* (ppm): 26.4 [C_γ_], 31.1 [C_β_], 35.8 [C_3_], 37.8 [*CH_2_*-Ph], 38.9 [C_δ_], 43.8 [CH_2_ (NH*Bn*)], 50.8 [C_α_], 51.9 [C_6_], 52.3 [C_2_-*CH*], 57.7 [*CH_2_*CO], 58.6 [C_2_], 66.7 [CH_2_ (Z)], 118.3, 122.2, 127.5, 127.8, 128.2, 128.5, 128.8, 128.9, 129.0, 129.3 [20CH (Ar)], 135.6 [C (Ph)], 136.2 [C (Z)], 137.3 [C (NH*Bn*)], 139.6 [C (NH*Ph*)], 155.6 [CO (Z)], 157.6 [CO (Urea)], 168.8 [C_5_], 169.2 [CO], 172.9 [α-CONH]; ES-MS *m/z* 734.5 [M+1]^+^; C_41_H_47_N_7_O_6_ (%): C: 67.10, H: 6.46, N: 13.36. Found (%): C: 67.21, H: 6.30, N: 13.49.

N*-[2-[5-Oxo-(2*R*)-[2-phenyl-(1*S*)-(3-phenylureido)ethyl]piperazin-1-yl]acetyl]-Lys(Z)-NHBn* [(***R***)-**9b**]. Amorphous solid (206 mg, 46%); 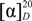 = −3.7 (*c* 1.5, MeOH); HPLC *t*_R_: 20.09 min; ^1^H-NMR (400 MHz, CDCl_3_) *δ* (ppm): 1.30 (m, 2H, γ-H), 1.40 (m, 2H, δ-H), 1.50 (m, 1H, β-H), 1.73 (m, 1H, β-H), 2.80 (m, 1H, *CH_2_*-Ph), 2.87 (m, 1H, *CH_2_*-Ph), 2.85 (m, 1H, ε-H), 2.92 (m, 1H, 2-H), 3.03 (m, 1H, *CH_2_*CO), 3.16 (m, 1H, 6-H), 3.20 (m, 2H, 3-H and ε-H), 3.38 (m, 1H, 6-H), 3.44 (m, 1H, *CH_2_*CO), 4.25 (m, 1H, 3-H), 4.28 (m, 1H, 2-*CH*), 4.30 [m, 1H, CH_2_ (NH*Bn*)], 4.38 [m, 1H, CH_2_ (NH*Bn*)], 4.50 (m, 1H, α-H), 5.01 [m, 2H, CH_2_ (Z)], 5.27 (m, 1H, *NH*Z), 5.98 (m, 1H, 2-CH*NH*), 6.23 (m, 1H, 4-H), 6.78–7.59 (m, 21H, Ar and *NH*Bn), 7.64 (m, 1H, *NH*Ph), 7.90 (m, 1H, α-NH); ^13^C-NMR (100 MHz, CDCl_3_) *δ* (ppm): 23.1 [C_γ_], 29.3 [C_δ_], 31.9 [C_β_], 38.3 [*CH_2_*-Ph], 39.6 [C_3_], 40.7 [C_ε_], 43.6 [CH_2_ (NH*Bn*)], 51.7 [C_6_], 53.9 [C_2_-*CH*], 54.8 [C_α_], 59.2 [*CH_2_*CO], 59.8 [C_2_], 66.8 [CH_2_ (Z)], 119.2, 119.9, 123.1, 127.1, 127.5, 127.6, 127.7, 128.1, 128.4, 128.8, 128.9, 129.0, 129.1 [20CH (Ar)], 136.8 [C (Ph)], 137.5 [C (Z)], 138.2 [C (NH*Bn*)], 139.2 [C (NH*Ph*)], 156.1 [CO (Z)], 156.9 [CO (Urea)], 170.5 [C_5_ and CO], 172.4 [α-CONH]; ES-MS *m/z* 748.6 [M+1]^+^; C_42_H_49_N_7_O_6_ (%): C: 67.45, H: 6.60, N: 13.11. Found (%): C: 67.62, H: 6.74, N: 13.02.

N*-[2-[5-Oxo-(2*S*)-[2-phenyl-(1*S*)-(3-phenylureido)ethyl]piperazin-1-yl]acetyl]-Lys(Z)-NHBn* [(***S***)-**9b**]. Amorphous solid (67 mg, 15%); 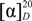 = +6.7 (*c* 0.9, MeOH); HPLC *t*_R_: 21.76 min; ^1^H-NMR (500 MHz, CDCl_3_) *δ* (ppm): 1.23 (m, 2H, γ-H), 1.34 (m, 2H, δ-H), 1.67 (m, 1H, β-H), 1.82 (m, 1H, β-H), 2.58 (t, 1H, *J* = 12.5 Hz, *CH_2_*-Ph), 2.87 (m, 1H, *CH_2_*-Ph), 2.90 (m, 1H, ε-H), 2.98 (m, 1H, 2-H), 3.05 (m, 1H, ε-H), 3.10 (m, 1H, 3-H), 3.18 (m, 1H, 6-H), 3.30 (m, 1H, *CH_2_*CO), 3.42 (m, 1H, *CH_2_*CO), 3.62 (m, 1H, 6-H), 3.95 (m, 1H, 2-*CH*), 4.05 (m, 1H, 3-H), 4.35 [m, 1H, CH_2_ (NH*Bn*)], 4.50 [m, 2H, CH_2_ (NH*Bn*) and α-H], 4.98 [m, 2H, CH_2_ (Z)], 5.02 (m, 1H, *NH*Z), 5.69 (m, 1H, 2-CH*NH*), 5.81 (m, 1H, 4-H), 6.94 (t, 1H, *J* = 7.5 Hz, *NH*Bn), 6.98–7.14 (m, 20H, Ar), 7.98 (m, 1H, *NH*Ph), 8.09 (m, 1H, α-NH); ^13^C-NMR (125 MHz, CDCl_3_) *δ* (ppm): 22.2 [C_γ_], 29.1 [C_δ_], 32.6 [C_β_], 35.7 [C_3_], 37.7 [*CH_2_*-Ph], 40.2 [C_ε_], 43.8 [CH_2_ (NH*Bn*)], 52.0 [C_6_ and C_2_-*CH*], 52.8 [C_α_], 57.8 [*CH_2_*CO], 58.7 [C_2_], 66.5 [CH_2_ (Z)], 118.4, 122.3, 127.7, 127.8, 128.1, 128.5, 128.9, 129.0, 129.3 [20CH (Ar)], 135.6 [C (Ph)], 136.5 [C (Z)], 137.1 [C (NH*Bn*)], 139.6 [C (NH*Ph*)], 155.6 [CO (Z)], 156.7 [CO (Urea)], 168.6 [C_5_], 169.9 [CO], 172.0 [α-CONH]; ES-MS *m/z* 748.7 [M+1]^+^; C_42_H_49_N_7_O_6_ (%): C: 67.45, H: 6.60, N: 13.11. Found (%): C: 67.31, H: 6.81, N: 13.25.

N*-[2-[-(2*R*)-[(1*S*)-(3-Benzylureido)-2-phenylethyl]-5-oxo-piperazin-1-yl]acetyl]-Orn(Z)-NHBn* [(***R***)-**10a**]. Amorphous solid (170 mg, 38%); 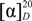 = −3.8 (*c* 1.2, MeOH); HPLC *t*_R_: 20.79 min; ^1^H-NMR (400 MHz, CDCl_3_) *δ* (ppm): 1.40 (m, 2H, γ-H), 1.55 (m, 1H, β-H), 1.70 (m, 1H, β-H), 2.57 (dd, 1H, *J* = 11 and 13.5, *CH_2_*-Ph), 2.86 (dd, 1H, *J* = 3.5 and 13.5, *CH_2_*-Ph), 3.00 (m, 1H, 2-H), 3.04 (m, 1H, δ-H), 3.09 (m, 1H, 3-H), 3.15 (m, 1H, 3-H), 3.25 (m, 2H, *CH_2_*CO), 3.32 (m, 1H, δ-H), 3.50 (d, 1H, *J* = 18 Hz, 6-H), 3.85 (m, 1H, 3-H), 3.94 (m, 1H, 2-*CH*), 4.17 [dd, 1H, *J* = 5 and 15 Hz, CH_2_ (NH*Bn*)], 4.23 [dd, 1H, *J* = 6 and 15 Hz, CH_2_ (NH*Bn*)], 4.27 [dd, 1H, *J* = 5 and 15 Hz, CH_2_ (NH*Bn*, Urea)], 4.36 [dd, 1H, *J* = 5.5 and 15 Hz, CH_2_ (NH*Bn*, Urea)], 4.66 (m, 1H, α-H), 4.79 [d, 1H, *J* = 13 Hz, CH_2_ (Z)], 4.89 [d, 1H, *J* = 13 Hz, CH_2_ (Z)], 5.30 (t, 1H, *J* = 6 Hz, *NH*Z), 5.75 (m, 1H, 2-CH*NH*), 5.90 (m, 1H, 4-H), 6.07 [t, 1H, *J* = 5.5 Hz, *NH*Bn (Urea)], 7.08–7.39 (m, 20H, Ar), 7.50 (t, 1H, *J* = 5.5 Hz, *NH*Bn), 7.99 [d, 1H, *J* = 8.5, α-NH); ^13^C-NMR (100 MHz, CDCl_3_) *δ* (ppm): 26.1 [C_γ_], 30.6 [C_β_], 35.8 [C_3_], 37.5 [*CH_2_*-Ph], 39.3 [C_δ_], 43.5 [CH_2_ (NH*Bn*)], 44.4 [CH_2_ (NH*Bn*, Urea)], 51.0 [C_α_], 51.8 [C_6_], 52.5 [C_2_-*CH*], 57.5 [*CH_2_*CO], 58.3 [C_2_], 66.5 [CH_2_ (Z)], 127.0, 127.4, 127.5, 127.8, 128.1, 128.4, 128.5, 128.7, 129.0, 129.2 [20CH (Ar)], 135.8 [C (Ph)], 136.5 [C (Z)], 137.5 [C (NH*Bn*)], 139.6 [C (NH*Bn*, Urea)], 157.2 [CO (Z)], 158.5 [CO (Urea)], 168.9 [C_5_], 170.0 [CO], 172.2 [α-CONH]; ES-MS *m/z* 748.6 [M+1]^+^; C_42_H_49_N_7_O_6_ (%): C: 67.45, H: 6.60, N: 13.11. Found (%): C: 67.28, H: 6.82, N: 13.20.

N*-[2-[-(2*S*)-[(1*S*)-(3-Benzylureido)-2-phenylethyl]-5-oxo-piperazin-1-yl]acetyl]-Orn(Z)-NHBn* [(***S***)-**10a**]. Amorphous solid (76 mg, 17%); 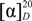 = −8.2 (*c* 1.0, MeOH); HPLC *t*_R_: 20.09 min; ^1^H-NMR (500 MHz, (CD_3_)_2_CO) *δ* (ppm): 1.55 (m, 2H, γ-H), 1.71 (m, 1H, β-H), 1.87 (m, 1H, β-H), 2.80 (dd, 1H, *J* = 10 and 14, *CH_2_*-Ph), 2.98 (dd, 1H, *J* = 6 and 13, 2-H), 3.06 (dd, 1H, *J* = 4 and 14, *CH_2_*-Ph), 3.12 (m, 1H, δ-H), 3.16 (d, 1H, *J* = 16.5 Hz, 6-H), 3.35 (s, 2H, *CH_2_*CO), 3.40 (d, 1H, *J* = 16.5 Hz, 6-H), 3.42 (m, 1H, 3-H), 3.51 (ddd, 1H, *J* = 4, 13 and 15 Hz, 3-H), 4.16 [dd, 1H, *J* = 6 and 15 Hz, CH_2_ (NH*Bn*, Urea)], 4.27 [dd, 1H, *J* = 5.5 and 15 Hz, CH_2_ (NH*Bn*, Urea)], 4.38 (m, 1H, 2-*CH*), 4.33 [d, 2H, *J* = 6 Hz, CH_2_ (NH*Bn*)], 4.56 (dt, 1H, *J* = 5 and 9 Hz, α-H), 5.00 [d, 1H, *J* = 4.5 Hz, CH_2_ (Z)], 4.89 [d, 1H, *J* = 13 Hz, CH_2_ (Z)], 6.00 [t, 1H, *J* = 6 Hz, *NH*Bn (Urea)], 5.89 (d, 1H, *J* = 9 Hz, 2-CH*NH*), 5.90 (m, 1H, 4-H), 6.46 (t, 1H, *J* = 5.5 Hz, *NH*Z), 7.07–7.35 (m, 21H, Ar and 1-H), 8.03 (t, 1H, *J* = 6 Hz, *NH*Bn), 8.14 [d, 1H, *J* = 8.5, α-NH); ^13^C-NMR (125 MHz, (CD_3_)_2_CO) *δ* (ppm): 27.8 [C_γ_], 31.5 [C_β_], 39.4 [*CH_2_*-Ph], 40.9 [C_3_], 41.6 [C_δ_], 44.1 [CH_2_ (NH*Bn*)], 44.8 [CH_2_ (NH*Bn*, Urea)], 53.5 [C_2_-*CH*], 54.1 [C_α_], 55.9 [C_6_], 59.6 [*CH_2_*CO], 62.5 [C_2_], 67.1 [CH_2_ (Z)], 127.6, 128.0, 128.3, 128.4, 128.8, 129.2, 129.3, 129.7, 129.8, 130.9 [20CH (Ar)], 139.2 [C (Z)], 140.5 [C (Ph)], 140.9 [C (NH*Bn*)], 142.3 [C (NH*Bn*, Urea)], 158.1 [CO (Z)], 159.8 [CO (Urea)], 170.7 [C_5_], 171.5 [CO], 173.5 [α-CONH]; ES-MS *m/z* 748.4 [M+1]^+^; C_42_H_49_N_7_O_6_ (%): C: 67.45, H: 6.60, N: 13.11. Found (%): C: 67.60, H: 6.85, N: 13.01.

N*-[2-[(2*R*)-[(1*S*)-(3-Benzylureido)-2-phenylethyl]-5-oxo-piperazin-1-yl]acetyl]-Lys(Z)-NHBn* [(***R***)-**10b**]. Amorphous solid (165 mg, 36%); 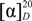 = −5.6 (*c* 0.8, MeOH); HPLC *t*_R_: 21.05 min; ^1^H-NMR (400 MHz, CDCl_3_) *δ* (ppm): 1.25 (m, 2H, γ-H), 1.42 (m, 2H, δ-H), 1.59 (dt, 1H, *J* = 7.5 and 14 Hz, β-H), 1.75 (m, 1H, β-H), 2.54 (dd, 1H, *J* = 11 and 13 Hz, *CH_2_*-Ph), 2.86 (dd, 1H, *J* = 2.5 and 13 Hz, *CH_2_*-Ph), 3.01 (m, 1H, 2-H), 3.04 (m, 1H, ε-H), 3.10 (m, 1H, ε-H), 3.12 (m, 1H, 6-H), 3.16 (m, 1H, 3-H), 3.22 (m, 1H, *CH_2_*CO), 3.28 (m, 1H, *CH_2_*CO), 3.53 (d, 1H, *J* = 18 Hz, 6-H), 3.86 (m, 1H, 3-H), 3.94 (m, 1H, 2-*CH*), 4.24 [d, 2H, *J* = 5.5 Hz, CH_2_ (NH*Bn*)], 4.27 [d, 1H, *J* = 4.5 Hz, CH_2_ (NH*Bn*, Urea)], 4.37 [d, 1H, *J* = 4.5 Hz, CH_2_ (NH*Bn*, Urea)], 4.43 (m, 1H, α-H), 5.00 [d, 2H, *J* = 7 Hz, CH_2_ (Z)], 5.24 (t, 1H, *J* = 5 Hz, *NH*Z), 5.66 (m, 1H, 2-CH*NH*), 5.77 (m, 1H, 4-H), 6.04 [m, 1H, (*NH*Bn, Urea)], 7.09–7.41 (m, 21H, Ar and *NH*Bn), 7.97 (d, 1H, *J* = 8 Hz, α-NH); ^13^C-NMR (100 MHz, CDCl_3_) *δ* (ppm): 22.4 [C_γ_], 29.2 [C_δ_], 32.5 [C_β_], 35.9 [C_3_], 37.5 [*CH_2_*-Ph], 40.4 [C_ε_], 43.6 [CH_2_ (NH*Bn*)], 44.2 [CH_2_ (NH*Bn*, Urea)], 51.7 [C_6_], 52.4 [C_2_-*CH*], 52.6 [C_α_], 57.7 [*CH_2_*CO], 58.5 [C_2_], 66.6 [CH_2_ (Z)], 127.1, 127.5, 127.6, 127.7, 127.9, 128.1, 128.5, 128.7, 129.0, 129.2 [20CH (Ar)], 135.8 [C (Ph)], 136.5 [C (Z)], 137.4 [C (NH*Bn*)], 139.6 [C (NH*Bn*, Urea)], 156.7 [CO (Z)], 158.3 [CO (Urea)], 168.6 [C_5_], 169.9 [CO], 172.0 [α-CONH]; ES-MS *m/z* 763.2 [M+1]^+^; C_43_H_51_N_7_O_6_ (%): C: 67.79, H: 6.75, N: 12.87. Found (%): C: 67.60, H: 7.01, N: 12.69.

N*-[2-[(2*S*)-[(1*S*)-(3-Benzylureido)-2-phenylethyl]-5-oxo-piperazin-1-yl]acetyl]-Lys(Z)-NHBn* [(***S***)-**10b**]. Amorphous solid (59 mg, 13%); 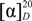 = −11.2 (*c* 0.9, MeOH); HPLC *t*_R_: 20.47 min; ^1^H-NMR (500 MHz, (CD_3_)_2_CO) *δ* (ppm): 1.30 (m, 2H, δ-H), 1.56 (m, 2H, γ-H), 1.68 (m, 1H, β-H), 1.83 (m, 1H, β-H), 2.82 (m, 1H, *CH_2_*-Ph), 3.02 (m, 1H, 2-H), 3.05 (m, 1H, *CH_2_*-Ph), 3.18 (m, 3H, ε-H and 6-H), 3.37 (m, 2H, *CH_2_*CO), 3.16 (m, 1H, 3-H), 3.40 (m, 1H, 6-H), 3.55 (m, 2H, 3-H), 4.15 [m, 1H, CH_2_ (NH*Bn*, Urea)], 4.25 [m, 1H, CH_2_ (NH*Bn*, Urea)], 4.28 [m, 2H, CH_2_ (NH*Bn*)], 4.40 (m, 1H, 2-*CH*), 4.51 (m, 1H, α-H), 5.00 [m, 2H, CH_2_ (Z)], 5.98 (d, 1H, *J* = 7 Hz, 2-CH*NH*), 6.04 [m, 1H, (*NH*Bn, Urea)], 6.45 (m, 1H, *NH*Z), 7.03 (m, 1H, 4-H), 7.08–7.41 (m, 20H, Ar), 8.04 (m, 1H, NH*Bn*), 8.15 (d, 1H, *J* = 8.5 Hz, α-NH); ^13^C-NMR (125 MHz, (CD_3_)_2_CO) *δ* (ppm): 26.2 [C_γ_], 29.7 [C_δ_], 29.9 [C_β_], 37.9 [*CH_2_*-Ph], 39.2 [C_3_], 39.9 [C_ε_], 42.5 [CH_2_ (NH*Bn*)], 43.2 [CH_2_ (NH*Bn*, Urea)], 52.0 [C_2_-*CH*], 52.6 [C_α_], 54.4 [C_6_], 57.6 [*CH_2_*CO], 61.0 [C_2_], 65.5 [CH_2_ (Z)], 126.1, 126.5, 126.6, 126.8, 127.3, 127.7, 127.8, 128.3, 128.4, 129.3, 129.6 [20CH (Ar)], 137.5 [C (Z)], 138.9 [C (Ph)], 139.4 [C (NH*Bn*)], 140.7 [C (NH*Bn*, Urea)], 156.5 [CO (Z)], 158.3 [CO (Urea)], 169.0 [C_5_], 169.7 [CO], 171.9 [α-CONH]; ES-MS *m/z* 763.3 [M+1]^+^; C_43_H_51_N_7_O_6_ (%): C: 67.79, H: 6.75, N: 12.87. Found (%): C: 67.96, H: 6.93, N: 12.70.

### 3.7. General Procedure for the *N*-Z Removal in **9a**,**b** and **10a**,**b**. Synthesis of the Hydrochlorides (**R**)-(**11a**,**b** and **12a**,**b**) and (**S**)-(**11a**,**b** and **12a**,**b**)

Pd(C) (10%) and a 3.4 N solution of HCl in EtOAc (134 μL, 0.40 mmol) were added to a solution of (***R***)-(**9a**,**b** and -**10a**,**b**) and (***S***)-(**9a**,**b** and **10a**,**b**) (0.20 mmol) in MeOH (5 mL), and the mixture was hydrogenated at 1 atm of H_2_ and room temperature for 1 h. Afterwards, the reaction mixture was filtered through celite and the solvent was evaporated under reduced pressure. The residue was dissolved in CH_3_CN/H_2_O (1:3, 2 mL) and the solution was lyophilized. (***R***)-(**11a**,**b** and **12a**,**b**) and (***S***)-(**11a**,**b** and **12a**,**b**) were obtained quantitatively.

N*-[2-[5-Oxo-(2*R*)-[2-phenyl-(1*S*)-(3-phenylureido)ethyl]piperazin-1-yl]acetyl]-Orn-NHBn hydrochloride* [(***R***)-**11a**]. Amorphous solid (127 mg, 100%); 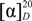 = +1.7 (*c* 0.7, MeOH); HPLC *t*_R_: 14.65 min; ^1^H-NMR (500 MHz, DMSO-*d_6_*) *δ* (ppm): 1.58 (m, 2H, γ-H), 1.63 (m, 1H, β-H), 1.80 (m, 1H, β-H), 2.62 (m, 1H, *CH_2_*-Ph), 2.63 (m, 1H, 2-H), 2.73 (m, 1H, *CH_2_*-Ph), 2.75 (m, 2H, δ-H), 3.05 (m, 1H, 3-H), 3.38 (m, 1H, 6-H), 3.40 (m, 2H, *CH_2_*CO and 3-H), 3.50 (m, 1H, *CH_2_*CO), 3.55 (m, 1H, 6-H), 4.05 (m, 1H, 2-*CH*), 4.24 [dd, 1H, *J* = 6 and 15 Hz, CH_2_ (NH*Bn*)], 4.31 [dd, 1H, *J* = 6 and 15 Hz, CH_2_ (NH*Bn*)], 4.39 (dd, 1H, *J* = 5 and 8 Hz, α-H), 6.58 (m, 1H, 2-CH*NH*), 6.80–7.35 (m, 15H, Ar), 7.86 (m, 4H, 4-H and NH_2_·HCl), ], 8.20 (m, 1H, α-NH), 8.65 (m, 1H, *NH*Bn), 8.74 [m, 1H, *NH*Ph]; ^13^C-NMR (125 MHz, DMSO-*d_6_*) *δ* (ppm): 23.5 [C_γ_], 29.2 [C_β_], 38.2 [C_δ_], 42.0 [CH_2_ (NH*Bn*)], 51.5 [C_α_], 117.6, 121.1, 126.1, 126.8, 127.1, 128.1, 128.3, 128.6, 129.3 [15CH (Ar)], 139.1 [C (NH*Bn*)], 140.2 [C (NH*Ph*)], 155.1 [CO (Urea)], 171.0 [α-CONH]; ES-MS *m/z* [M]^+^ calculated for C_33_H_41_N_7_O_4_: 600.2; found: 600.5.

N*-[2-[5-Oxo-(2*S*)-[2-phenyl-(1*S*)-(3-phenylureido)ethyl]piperazin-1-yl]acetyl]-Orn-NHBn hydrochloride* [(***S***)-**11a**]. Amorphous solid (127 mg, 100%); 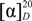 = −1.6 (*c* 1.1, MeOH); HPLC *t*_R_: 15.07 min; ^1^H-NMR (500 MHz, DMSO-*d_6_*) *δ* (ppm): 1.48 (m, 2H, γ-H), 1.58 (m, 1H, β-H), 1.74 (m, 1H, β-H), 2.62 (m, 2H, δ-H), 2.79 (m, 1H, *CH_2_*-Ph), 2.83 (m, 1H, 2-H), 2.89 (m, 1H, *CH_2_*-Ph), 3.00 (m, 1H, 3-H), 3.35 (m, 1H, *CH_2_*CO), 3.40 (m, 1H, 6-H), 3.42 (m, 1H, 3-H), 3.50 (m, 1H, *CH_2_*CO), 3.65 (m, 1H, 6-H), 4.04 (m, 1H, 2-*CH*), 4.25 [d, 2H, *J* = 6, CH_2_ (NH*Bn*)], 4.31 (dd, 1H, *J* = 5 and 8 Hz, α-H), 6.70 (m, 1H, 2-CH*NH*), 6.79–7.49 (m, 15H, Ar), 7.81 (m, 4H, 4-H and NH_2_·HCl), ], 8.21 (m, 1H, α-NH), 8.65 (t, 1H, *J* = 6 Hz, *NH*Bn), 8.92 [m, 1H, *NH*Ph]; ^13^C-NMR (125 MHz, DMSO-*d_6_*) *δ* (ppm): 23.4 [C_γ_], 28.9 [C_β_], 38.1 [C_δ_], 42.0 [CH_2_ (NH*Bn*)], 51.5 [C_α_], 117.6, 121.1, 126.4, 126.7, 127.0, 128.2, 128.4, 128.6, 129.0 [15CH (Ar)], 137.0 [C (Ph)], 139.1 [C (NH*Bn*)], 140.3 [C (NH*Ph*)], 155.3 [CO (Urea)], 170.9 [α-CONH]; ES-MS *m/z* [M]^+^ calculated for C_33_H_41_N_7_O_4_: 600.2; found: 600.5.

N*-[2-[5-Oxo-(2*R*)-[2-phenyl-(1*S*)-(3-phenylureido)ethyl]piperazin-1-yl]acetyl]-Lys-NHBn hydrochloride* [(***R***)-**11b**]. Amorphous solid (130 mg, 100%); 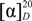 = −3.0 (*c* 1.7, MeOH); HPLC *t*_R_: 14.97 min; ^1^H-NMR (500 MHz, DMSO-*d_6_*) *δ* (ppm): 1.27 (m, 2H, γ-H), 1.52 (m, 2H, δ-H), 1.58 (m, 1H, β-H), 1.70 (m, 1H, β-H), 2.61 (m, 1H, *CH_2_*-Ph), 2.72 (m, 1H, ε-H), 2.84 (m, 1H, 2-H), 2.92 (m, 1H, ε-H), 2.95 (m, 1H, *CH_2_*-Ph), 3.00 (m, 2H, 3-H), 3.38 (m, 1H, 3-H), 3.40 (m, 1H, *CH_2_*CO), 3.42 (m, 1H, 6-H), 3.50 (m, 1H, *CH_2_*CO), 3.54 (m, 1H, 3-H), 4.22 [m, 1H, CH_2_ (NH*Bn*)], 4.23 (m, 1H, 2-*CH*), 4.31 [m, 1H, CH_2_ (NH*Bn*)], 4.32 (m, 1H, α-H), 6.80–7.37 (m, 16H, Ar and 2-CH*NH*), 7.88 (m, 4H, NH_2_·HCl and 4-H), 8.17 (m, 1H, α-NH), 8.61 (t, 1H, *J* = 6 Hz, *NH*Bn), 8.90 (m, 1H, *NH*Ph); ^13^C-NMR (125 MHz, DMSO-*d_6_*) *δ* (ppm): 22.2 [C_γ_], 26.5 [C_δ_], 30.7 [C_β_], 38.4 [C_ε_], 42.0 [CH_2_ (NH*Bn*)], 52.5 [C_α_], 117.7, 121.3, 126.3, 126.7, 127.0, 128.2, 128.3, 128.5, 129.2 [15CH (Ar)], 138.1 [C (Ph)], 139.2 [C (NH*Bn*)], 140.0 [C (NH*Ph*)], 155.3 [CO (Urea)], 171.3 [α-CONH]; ES-MS *m/z* [M]^+^ calculated for C_34_H_43_N_7_O_4_: 614.2; found: 614.5.

N*-[2-[5-Oxo-(2*S*)-[2-phenyl-(1*S*)-(3-phenylureido)ethyl]piperazin-1-yl]acetyl]-Lys-NHBn hydrochloride* [(***S***)-**11b**]. Amorphous solid (130 mg, 100%); 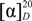 = −4.2 (*c* 0.4, MeOH); HPLC *t*_R_: 15.21 min; ^1^H-NMR (500 MHz, DMSO-*d_6_*) *δ* (ppm): 1.29 (m, 2H, γ-H), 1.50 (m, 2H, δ-H), 1.55 (m, 1H, β-H), 1.73 (m, 1H, β-H), 2.72 (m, 1H, *CH_2_*-Ph), 2.72 (m, 1H, ε-H), 2.74 (m, 2H, 3-H), 2.80 (m, 1H, ε-H), 2.89 (m, 1H, *CH_2_*-Ph), 2.90 (m, 1H, 2-H), 3.13 (m, 1H, *CH_2_*CO), 3.29 (m, 1H, 3-H), 3.33 (m, 1H, 6-H), 3.42 (m, 1H, *CH_2_*CO), 3.63 (m, 1H, 6-H), 4.10 (m, 1H, 2-*CH*), 4.18 [m, 1H, CH_2_ (NH*Bn*)], 4.20 (m, 1H, α-H), 4.35 [m, 1H, CH_2_ (NH*Bn*)], 6.55 (m, 1H, 2-CH*NH*), 6.80–7.40 (m, 16H, Ar), 7.69 (m, 4H, NH_2_·HCl and 4-H), 7.92 (d, 1H, *J* = 9 Hz, α-NH), 8.56 (m, 1H, *NH*Bn), 8.70 (m, 1H, *NH*Ph); ^13^C-NMR (125 MHz, DMSO-*d_6_*) *δ* (ppm): 22.1 [C_γ_], 26.8 [C_δ_], 30.7 [C_β_], 38.5 [C_ε_], 42.0 [CH_2_ (NH*Bn*)], 51.6 [C_α_], 116.5, 118.0, 127.0, 127.2, 127.5, 128.7, 128.9, 129.1, 129.5 [15CH (Ar)], 138.2 [C (Ph)], 139.4 [C (NH*Bn*)], 140.0 [C (NH*Ph*)], 171.2 [α-CONH]; ES-MS *m/z* [M]^+^ calculated for C_34_H_43_N_7_O_4_: 614.2; found: 614.5.

N*-[2-[(2*R*)-[(1*S*)-(3-Benzylureido)-2-phenylethyl]-5-oxo-piperazin-1-yl]acetyl]-Orn-NHBn hydrochloride* [(***R***)-**12a**]. Amorphous solid (130 mg, 100%); 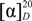 = −7.9 (*c* 1.3, MeOH); HPLC *t*_R_: 14.67 min; ^1^H-NMR (500 MHz, DMSO-*d_6_*) *δ* (ppm): 1.56 (m, 3H, γ-H and β-H), 1.78 (m, 1H, β-H), 2.60–3.16 (m, 6H, *CH_2_*-Ph, 2-H, δ-H and 3-H), 3.20–3.86 (m, 5H, 3-H, 6-H and *CH_2_*CO), 4.04 (m, 1H, 2-*CH*), 4.20 [m, 2H, CH_2_ (NH*Bn*, Urea)], 4.26 [d, 2H, *J* = 6 Hz, CH_2_ (NH*Bn*)], 4.34 (dd, 1H, *J* = 5 and 8 Hz, α-H), 6.53 (m, 1H, 2-CH*NH*), 6.74 [m, 1H, *NH*Bn (Urea)], 7.18–7.33 (m, 15H, Ar), 7.92 (m, 4H, NH_2_·HCl and 4-H), 8.34 [m, 1H, α-NH), 8.70 (t, 1H, *J* = 6 Hz, *NH*Bn); ^13^C-NMR (125 MHz, DMSO-*d_6_*) *δ* (ppm): 23.8 [C_γ_], 29.5 [C_β_], 39.5 [C_δ_], 42.5 [CH_2_ (NH*Bn*)], 43.4 [CH_2_ (NH*Bn*, Urea)], 52.0 [C_α_], 126.9, 127.0, 127.2, 127.4, 127.5, 128.6, 128.7, 128.9, 129.3 [15CH (Ar)], 137.5 [C (Ph)], 139.6 [C (NH*Bn*)], 141.0 [C (NH*Bn*, Urea)], 158.7 [CO (Urea)], 171.4 [α-CONH]; ES-MS *m/z* [M]^+^ calculated for C_34_H_43_N_7_O_4_: 614.2; found: 614.5.

N*-[2-[(2*S*)-[(1*S*)-(3-Benzylureido)-2-phenylethyl]-5-oxo-piperazin-1-yl]acetyl]-Orn-NHBn hydrochloride* [(***S***)-**12a**]. Amorphous solid (130 mg, 100%); 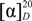 = −3.2 (*c* 1.2, MeOH); HPLC *t*_R_: 15.01 min; ^1^H-NMR (500 MHz, DMSO-*d_6_*) *δ* (ppm): 1.58 (m, 3H, γ-H), 1.60 (m, 1H, β-H) 1.88 (m, 1H, β-H), 2.52–3.97 (m, 6H, *CH_2_*-Ph, 2-H, δ-H and 3-H), 3.24–3.69 (m, 5H, 3-H, 6-H and *CH_2_*CO), 4.03 (m, 1H, 2-*CH*), 4.05 [m, 1H, CH_2_ (NH*Bn*, Urea)], 4.20 [m, 1H, CH_2_ (NH*Bn*, Urea)], 4.25 [m, 2H, CH_2_ (NH*Bn*)], 4.39 (m, 1H, α-H), 6.48 (m, 1H, 2-CH*NH*), 6.96–7.36 [m, 16H, Ar and *NH*Bn (Urea)], 7.90 (m, 4H, NH_2_·HCl and 4-H), 8.30 [m, 1H, α-NH), 8.70 (m, 1H, *NH*Bn); ^13^C-NMR (125 MHz, DMSO-*d_6_*) *δ* (ppm): 23.9 [C_γ_], 29.6 [C_β_], 38.7 [C_δ_], 42.5 [CH_2_ (NH*Bn*)], 43.1 [CH_2_ (NH*Bn*, Urea)], 52.1 [C_α_], 126.5, 126.8, 127.0, 127.1, 127.5, 128.5, 128.7, 129.7 [15CH (Ar)], 139.6 [C (NH*Bn*)], 141.0 [C (NH*Bn*, Urea)], 158.5 [CO (Urea)], 171.3 [α-CONH]; ES-MS *m/z* [M]^+^ calculated for C_34_H_43_N_7_O_4_: 614.2; found: 614.5.

N*-[2-[(2*R*)-[(1*S*)-(3-Benzylureido)-2-phenylethyl]-5-oxo-piperazin-1-yl]acetyl]-Lys-NHBn hydrochloride* [(***R***)-**12b**]. Amorphous solid (133 mg, 100%); 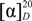 = −4.3 (c 0.6, MeOH); HPLC *t*_R_: 14.80 min; ^1^H-NMR (500 MHz, DMSO-*d_6_*) *δ* (ppm): 1.21 (m, 2H, γ-H), 1.49 (m, 2H, δ-H), 1.57 (m, 1H, β-H), 1.69 (m, 1H, β-H), 2.68 (m, 2H, ε-H), 2.75 (m, 1H, *CH_2_*-Ph), 2.80 (m, 1H, 2-H), 2.82 (m, 1H, *CH_2_*-Ph), 3.02 (m, 1H, 3-H), 3.25 (m, 1H, *CH_2_*CO), 3.37 (m, 1H, 6-H), 3.40 (m, 2H, *CH_2_*CO and 3-H), 3.60 (m, 1H, 6-H), 3.98 (m, 1H, 2-*CH*), 4.20 [m, 2H, CH_2_ (NH*Bn*, Urea)], 4.24 (m, 1H, α-H), 4.25 [m, 2H, CH_2_ (NH*Bn*)], 6.35 (m, 1H, 2-CH*NH*), 6.59 [m, 1H, (*NH*Bn, Urea)], 7.08–7.35 (m, 15H, Ar), 7.82 (m, 4H, NH_2_·HCl and 4-H), 8.08 (m, 1H, α-NH), 8.57 (t, 1H, *J* = 6 Hz, *NH*Bn); ^13^C-NMR (125 MHz, DMSO-*d_6_*) *δ* (ppm): 22.1 [C_γ_], 26.5 [C_δ_], 31.4 [C_β_], 38.5 [C_ε_], 42.0 [CH_2_ (NH*Bn*)], 42.9 [CH_2_ (NH*Bn*, Urea)], 52.0 [C_α_], 126.4, 126.5, 126.7, 126.9, 127.0, 128.2, 128.4, 129.1 [15CH (Ar)], 137.3 [C (Ph)], 139.3 [C (NH*Bn*)], 140.7 [C (NH*Bn*, Urea)], 158.0 [CO (Urea)], 171.2 [α-CONH]; ES-MS *m/z* [M]^+^ calculated for C_35_H_45_N_7_O_4_: 628.2; found: 628.5.

N*-[2-[(2*S*)-[(1*S*)-(3-Benzylureido)-2-phenylethyl]-5-oxo-piperazin-1-yl]acetyl]-Lys-NHBn hydrochloride* [(***S***)-**12b**]. Amorphous solid (133 mg, 100%); 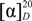 = −1.6 (c 0.6, MeOH); HPLC *t*_R_: 15.34 min; ^1^H-NMR (500 MHz, DMSO-*d_6_*) *δ* (ppm): 1.28 (m, 2H, γ-H), 1.47 (m, 1H, β-H), 1.50 (m, 2H, δ-H), 1.70 (m, 1H, β-H), 2.57–3.01 (m, 7H, *CH_2_*-Ph, ε-H, 2-H and 3-H), 3.38 (m, 1H, 6-H), 3.42 (m, 1H, *CH_2_*CO), 3.53 (m, 1H, *CH_2_*CO), 3.62 (m, 1H, 6-H), 4.00 [m, 1H, CH_2_ (NH*Bn*, Urea)], 4.17 [m, 1H, CH_2_ (NH*Bn*, Urea)], 4.20 (m, 1H, 2-*CH*), 4.26 [m, 2H, CH_2_ (NH*Bn*)], 4.30 (m, 1H, α-H), 6.45 (m, 1H, 2-CH*NH*), 6.96–7.34 [m, 16H, Ar and (*NH*Bn, Urea)], 7.84 (m, 4H, NH_2_·HCl and 4-H), 8.05 (m, 1H, α-NH), 8.60 (m, 1H, *NH*Bn); ^13^C-NMR (125 MHz, DMSO-*d_6_*) *δ* (ppm): 22.2 [C_γ_], 26.5 [C_δ_], 31.4 [C_β_], 38.5 [C_ε_], 42.0 [CH_2_ (NH*Bn*)], 42.6 [CH_2_ (NH*Bn*, Urea)], 52.5 [C_α_], 126.4, 126.6, 126.7, 127.0, 128.1, 128.2, 129.3 [15CH (Ar)], 137.4 [C (Ph)], 139.6 [C (NH*Bn*)], 141.0 [C (NH*Bn*, Urea)], 159.0 [CO (Urea)], 171.5 [α-CONH]; ES-MS *m/z* [M]^+^ calculated for C_35_H_45_N_7_O_4_: 628.2; found: 628.5.

### 3.8. Synthesis of 2-[4-Benzyl-(2RS)-[(1S)-((tert-butoxycarbonyl)amino)-2-phenylethyl]-5-oxopiperazin-1-yl]acetic Acid (**14**)

This compound was obtained from the benzyl ester **13** [23] by applying the general procedure of benzyl ester hydrogenolysis above indicated for the synthesis of **4**. Foam (467.6 mg, 100%); HPLC *t*_R_: 19.72 min [(***R***)-**14**] and 19.00 min [(***S***)-**14**]; ^1^H-NMR (500 MHz, CDCl_3_). (***R***)-**14** *δ* (ppm): 1.35 (s, 9H, Boc), 2.73 (m, 2H, *CH_2_*-Ph), 2.87 (m, 1H, 2-H), 3.23 (d, 1H, *J* = 7.5 and 13 Hz, 3-H), 3.33 (d, 1H, *J* = 5 and 13 Hz, 3-H), 3.38 (d, 1H, *J* = 17 Hz, *CH_2_*CO_2_H), 3.49 (d, 2H, *J* = 17 Hz, 6-H and *CH_2_*CO_2_H), 3.62 (d, 1H, *J* = 17 Hz, 6-H), 4.00 (m, 1H, 2-*CH*), 4.30 (d, 1H, *J* = 9 Hz, *NH*Boc), 4.52 [d, 1H, *J* = 14.5 Hz, 4-CH_2_ (Bn)], 4.68 [d, 1H, *J* = 14.5 Hz, 4-CH_2_ (Bn)], 6.93–7.40 (m, 10H, Ar). (***S***)-**14** *δ* (ppm): 1.35 (s, 9H, Boc), 2.73 (m, 2H, *CH_2_*-Ph), 2.97 (m, 1H, 2-H), 3.17 (m, 1H, 3-H), 3.33 (m, 1H, 3-H), 3.23 (m, 1H, *CH_2_*CO_2_H), 3.38 (m, 1H, 3-H), 3.49 (m, 1H, *CH_2_*CO_2_H), 3.63 (d, 1H, *J* = 17.5 Hz, 6-H), 3.82 (m, 1H, 2-*CH*), 4.30 (d, 1H, *J* = 9 Hz, *NH*Boc), 4.57 [m, 1H, 4-CH_2_ (Bn)], 4.82 [m, 1H, 4-CH_2_ (Bn)], 6.93–7.40 (m, 10H, Ar). ^13^C-NMR (125 MHz, DMSO-*d_6_*). *(**R**)*-**14** *δ* (ppm): 28.2 [3CH_3_ (Boc)], 37.5 [*CH_2_*-Ph], 44.1 [C_3_], 49.7 [4-CH_2_ (Bn)], 51.6 [C_2_-*CH*], 54.1 [C_6_], 54.4 [*CH_2_*CO_2_H], 58.7 [C_2_], 80.2 [C (Boc)], 126.7, 127.9, 128.6, 128.9 [10CH (Ar)], 136.2 [C (Bn)], 136.8 [C (Ph)], 155.7 [CO (Boc)], 167.8 [C_5_], 172.2 [CO_2_]. *(**S**)*-**14** *δ* (ppm): 28.2 [3CH_3_ (Boc)], 37.5 [*CH_2_*-Ph], 44.1 [C_3_], 49.6 [4-CH_2_ (Bn)], 51.6 [C_2_-*CH*], 54.1 [C_6_], 54.4 [*CH_2_*CO_2_H], 58.7 [C_2_], 80.2 [C (Boc)], 128.0, 128.3, 128.4, 129.1 [10CH (Ar)], 136.2 [C (Bn)], 136.8 [C (Ph)], 155.7 [CO (Boc)], 167.8 [C_5_], 172.2 [CO_2_]; ES-MS *m/z* 468.2 [M+1]^+^; C_26_H_33_N_3_O_5_ (%): C: 66.79, H: 7.11, N: 8.99. Found (%): C: 66.58, H: 7.25, N: 9.14.

### 3.9. General Procedure for the Synthesis of the Piperazinone-Derived Pseudotripeptides **15a**–**c**

These compounds were prepared by applying the general procedure described for the synthesis of **7a**,**b**.

N*-[2-[4-Benzyl-(2*RS*)-[(1*S*)-((*tert*-butoxycarbonyl)-amino)-2-phenylethyl]-5-oxopiperazin-1-yl]acetyl]-Orn(Z)-NHBn* (**15a**). Foam (523 mg, 65%); HPLC *t*_R_: 25.24 min; ^1^H-NMR (500 MHz, CDCl_3_) (***R***)-**15a** *δ* (ppm): 1.34 (s, 9H, Boc), 1.43 (m, 2H, γ-H), 1.67 (m, 1H, β-H), 1.86 (m, 1H, β-H), 2.76 (m, 1H, *CH_2_*-Ph), 2.83 (m, 1H, *CH_2_*-Ph), 2.86 (m, 1H, 2-H), 3.08 (m, 1H, 3-H), 3.23 (m, 2H, δ-H), 3.30 (m, 1H, 3-H), 3.35 (m, 1H, 6-H), 3.44 (m, 2H, *CH_2_*CO), 3.55 (d, 1H, *J* = 18 Hz, 6-H), 4.00 (m, 1H, 2-*CH*), 4.34 [dd, 1H, *J* = 6 and 15 Hz, CH_2_ (NH*Bn*)], 4.42 (m, 1H, α-H), 4.39 [m, 1H, CH_2_ (N*Bn*)], 4.44 [m, 1H, CH_2_ (NH*Bn*)], 4.70 (m, 1H, *NH*Boc), 4.75 [m, 1H, CH_2_ (N*Bn*)], 4.86 [m, 2H, CH_2_ (Z)], 5.06 (m, 1H, *NH*Z), 6.70 (m, 1H, *NH*Bn), 7.02–7.46 (m, 20H, Ar), 7.74 (m, 1H, α-NH). (***S***)-**15a** *δ* (ppm): 1.34 (s, 9H, Boc), 1.67 (m, 1H, β-H), 1.86 (m, 1H, β-H), 2.56 (m, 1H, *CH_2_*-Ph), 2.74 (m, 1H, *CH_2_*-Ph), 3.08 (m, 1H, 3-H), 3.23 (m, 2H, δ-H), 3.30 (m, 1H, 3-H), 3.85 (m, 1H, 2-*CH*), 4.34 [m, 1H, CH_2_ (NH*Bn*)], 4.42 (m, 1H, α-H), 4.44 [m, 1H, CH_2_ (NH*Bn*)], 4.44 [m, 1H, CH_2_ (NH*Bn*)], 4.50 [m, 1H, CH_2_ (N*Bn*)], 4.64 (m, 1H, *NH*Boc), 4.80 [m, 1H, CH_2_ (N*Bn*)], 4.86 [m, 2H, CH_2_ (Z)], 5.06 (m, 1H, *NH*Z), 6.70 (m, 1H, *NH*Bn), 7.02–7.46 (m, 20H, Ar), 7.74 (m, 1H, α-NH); ^13^C-NMR (125 MHz, CDCl_3_) (***R***)-**15a** *δ* (ppm): 26.7 [C_γ_], 28.4 [3CH3 (Boc)], 31.1 [C_β_], 37.7 [*CH_2_*-Ph], 39.5 [C_δ_], 43.7 [C_3_ and CH_2_ (NH*Bn*)], 50.1 [CH_2_ (N*Bn*)], 51.4 [C_2_-*CH*], 51.7 [C_α_], 54.6 [C_6_], 56.3 [*CH_2_*CO], 59.5 [C_2_], 66.8 [CH_2_ (Z)], 80.1 [C (Boc)], 126.9, 127.9, 128.0, 128.3, 128.5, 128.8, 129.0, 129.3 [20CH (Ar)], 136.5 [C (Ph) and C (N*Bn*)], 136.9 [C (Z)], 138.1 [C (NH*Bn*)], 155.6 [CO (Boc)], 157.3 [CO (Z)], 166.9 [C_5_], 169.6 [CO], 171.5 [α-CONH]. (***S***)-**15a** *δ* (ppm): 28.4 [3CH_3_ (Boc)], 30.5 [C_β_], 37.7 [*CH_2_*-Ph], 39.6 [C_δ_], 43.7 [C_3_ and CH_2_ (NH*Bn*)], 49.8 [CH_2_ (N*Bn*)], 51.4 [C_2_-*CH*], 51.7 [C_α_], 66.8 [CH_2_ (Z)], 80.1 [C (Boc)], 126.8, 127.8, 128.0, 128.6, 128.8, 129.1, 129.3 [20CH (Ar)], 136.5 [C (Ph) and C (N*Bn*)], 136.9 [C (Z)], 138.1 [C (NH*Bn*)], 155.6 [CO (Boc)], 157.3 [CO (Z)], 166.9 [C_2_], 169.6 [CO], 171.5 [α-CONH]; ES-MS *m/z* 806.6 [M+1]^+^; C_46_H_56_N_6_O_7_ (%): C: 68.63, H: 7.01, N: 10.44. Found (%): C: 68.50, H: 7.19, N: 10.62.

N*-[2-[4-Benzyl-(2*RS*)-[(1*S*)-((*tert*-butoxycarbonyl)-amino)-2-phenylethyl]-5-oxopiperazin-1-yl]acetyl]-Lys(Z)-NHBn* (**15b**). Foam (639 mg, 78%); HPLC *t*_R_: 25.42 min; ^1^H-NMR (500 MHz, CDCl_3_) (***R***)-**15b** *δ* (ppm): 1.31 (m, 11H, Boc and γ-H), 1.47 (m, 2H, δ-H), 1.65 (m, 1H, β-H), 1.86 (m, 1H, β-H), 2.69 (dd, 1H, *J* = 8 and 13 Hz, *CH_2_*-Ph), 2.78 (m, 1H, 2-H), 2.82 (m, 1H, *CH_2_*-Ph), 3.12 (m, 2H, ε-H), 3.19 (m, 1H, 6-H), 3.20 (m, 1H, *CH_2_*CO), 3.25 (m, 1H, 3-H), 3.35 (m, 1H, 6-H), 3.37 (m, 1H, *CH_2_*CO), 3.50 (d, 1H, *J* = 17 Hz, 3-H), 3.95 (m, 1H, 2-*CH*), 4.29 (d, 1H, *J* = 9 Hz, *NH*Boc), 4.39 [m, 1H, CH_2_ (N*Bn*], 4.42 [m, 1H, CH_2_ (NH*Bn*)], 4.44 (m, 1H, α-H), 4.45[m, 1H, CH_2_ (NH*Bn*)], 4.75 [d, 1H, *J* = 14.5 Hz, CH_2_ (N*Bn*)], 5.04 [m, 3H, CH_2_ (Z) and *NH*Z], 6.74 (m, 1H, *NH*Bn), 7.01–7.38 (m, 20H, Ar), 7.67 (d, 1H, *J* = 8 Hz, α-NH). (***S***)-**15b** *δ* (ppm): 1.31 (m, 9H, Boc), 1.65 (m, 1H, β-H), 1.86 (m, 1H, β-H), 3.12 (m, 1H, *CH_2_*CO), 3.38 (m, 1H, 6-H), 3.39 (m, 1H, *CH_2_*CO), 3.54 (m, 1H, 6-H), 3.82 (m, 1H, 2-*CH*), 4.33 (d, 1H, *J* = 9 Hz, *NH*Boc), 4.42 [m, 1H, CH_2_ (NH*Bn*)], 4.43 (m, 1H, α-H), 4.45[m, 1H, CH_2_ (NH*Bn*)], 4.72 [m, 1H, CH_2_ (N*Bn*], 4.79 [m, 1H, CH_2_ (N*Bn*], 5.04 [m, 3H, CH_2_ (Z) and *NH*Z], 6.96 (m, 1H, *NH*Bn), 7.01–7.38 (m, 20H, Ar), 7.55 (m, 1H, α-NH); ^13^C-NMR (125 MHz, CDCl_3_) (***R***)-**15b** *δ* (ppm): 22.7 [C_γ_], 28.2 [3CH_3_ (Boc)], 29.4 [C_δ_], 31.9 [C_β_], 37.7 [*CH_2_*-Ph], 40.5 [C_ε_], 43.6 [C_3_ and CH_2_ (NH*Bn*)], 49.8 [CH_2_ (N*Bn*)], 51.7 [C_2_-*CH*], 52.7 [C_α_], 54.4 [C_6_], 56.1 [*CH_2_*CO], 59.4 [C_2_], 66.5 [CH_2_ (Z)], 79.9 [C (Boc)], 127.0, 127.7, 127.8, 128.0, 128.2, 128.5, 128.6, 128.8, 128.9, 129.0, 129.3 [20CH (Ar)], 136.2 [C (N*Bn*)], 136.6 [C (Ph)], 136.7 [C (Z)], 138.0 [C (NH*Bn*)], 155.4 [CO (Boc)], 156.4 [CO (Z)], 166.9 [C_5_], 169.6 [CO], 171.2 [α-CONH]. (***S***)-**15b** *δ* (ppm): 28.2 [3CH3 (Boc)], 31.6 [C_β_], 43.6 [C_3_ and CH_2_ (NH*Bn*)], 49.6 [CH_2_ (N*Bn*)], 51.7 [C_2_-*CH*], 54.4 [C_6_], 56.1 [*CH_2_*CO], 66.5 [CH_2_ (Z)], 79.9 [C (Boc)], 126.8, 127.7, 127.8, 128.1, 128.2, 128.5, 128.7, 128.8, 128.9, 129.0, 129.2 [20CH (Ar)], 136.2 [C (N*Bn*)], 136.6 [C (Ph)], 136.7 [C (Z)], 138.0 [C (NH*Bn*)], 155.4 [CO (Boc)], 156.4 [CO (Z)], 169.6 [CO], 171.2 [α-CONH]; ES-MS *m/z* 819.7 [M+1]^+^; C_47_H_58_N_6_O_7_ (%): C: 68.93, H: 7.14, N: 10.26. Found (%): C: 68.67, H: 7.36, N: 10.20.

N*-[2-[4-Benzyl-(2*RS*)-[(1*S*)-((*tert*-butoxycarbonyl)-amino)-2-phenylethyl]-5-oxopiperazin-1-yl]acetyl]-Arg(Pbf)-NHBn* (**15c**). Foam (705 mg, 73%); HPLC *t*_R_: 26.88 min; ^1^H-NMR (500 MHz, CDCl_3_) (***R***)-**15c** *δ* (ppm): 1.28 (s, 9H, Boc), 1.45 [s, 6H, 2CH_3_ (Pbf)], 1.53 (m, 2H, γ-H), 1.67 (m, 1H, β-H), 1.90 (m, 1H, β-H), 2.07 [s, 3H, CH_3_ (Pbf)], 2.48 [s, 3H, CH_3_ (Pbf)], 2.55 [s, 3H, CH_3_ (Pbf)], 2.73 (d, 1H, *J* = 8.5 and 13.5 Hz, *CH_2_*-Ph), 2.82 (m, 1H, 5-H), 2.84 (m, 1H, *CH_2_*-Ph), 2.93 [m, 2H, CH_2_ (Pbf)], 3.24 (m, 5H, 3-H, *CH_2_*CO and δ-H), 3.30 (m, 1H, 3-H), 3.32 (m, 1H, 6-H), 3.49 (d, 1H, *J* = 16.5 Hz, 6-H), 3.95 (m, 1H, 2-*CH*), 4.31 [dd, 1H, *J* = 6 and 15 Hz, CH_2_ (NH*Bn*)], 4.38 [d, 1H, *J* = 14.5 Hz, CH_2_ (N*Bn*)], 4.41 [dd, 1H, *J* = 5.5 and 15 Hz, CH_2_ (NH*Bn*)], 4.50 (d, 1H, *J* = 9 Hz, *NH*Boc), 4.57 (dt, 1H, *J* = 4.5 and 9 Hz, α-H), 4.76 [d, 1H, *J* = 14.5 Hz, CH_2_ (N*Bn*)], 6.41 [m, 3H, NHC(NH_2_) = N], 6.81–7.24 (m, 15H, Ar), 7.60 (m, 1H, *NH*Bn), 7.74 (d, 1H, *J* = 8 Hz, α-NH). (***S***)-**15c** *δ* (ppm): 1.28 (s, 9H, Boc), 1.45 [s, 6H, 2CH_3_ (Pbf)], 1.67 (m, 1H, β-H), 1.90 (m, 1H, β-H), 2.07 [s, 3H, CH_3_ (Pbf)], 2.48 [s, 3H, CH_3_ (Pbf)], 2.55 [s, 3H, CH_3_ (Pbf)], 2.73 (m, 1H, *CH_2_*-Ph), 2.84 (m, 1H, *CH_2_*-Ph), 2.93 [m, 2H, CH_2_ (Pbf)], 3.20 (m, 1H, 3-H), 3.35 (m, 1H, 3-H), 3.83 (m, 1H, 2-*CH*), 4.31 [m, 1H, CH_2_ (N*Bn*)], 4.41 [m, 1H, CH_2_ (N*Bn*)], 4.50 (d, 1H, *J* = 9 Hz, *NH*Boc), 4.57 (m, 1H, α-H), 4.70 [m, 1H, CH_2_ (N*Bn*)], 4.86 [m, 1H, CH_2_ (N*Bn*)], 6.41 [m, 3H, NHC(NH_2_) = N], 6.81–7.24 (m, 15H, Ar), 7.62 (m, 1H, *NH*Bn), 7.83 (d, 1H, *J* = 8 Hz, α-NH); ^13^C-NMR (125 MHz, CDCl_3_) (***R***)-**15c** *δ* (ppm): 12.4, 18.0, 19.3 [3CH3 (Pbf)], 25.4 [C_γ_], 28.2 [3CH_3_ (Boc)], 28.6 [2CH3 (Pbf)], 31.0 [C_β_], 37.6 [*CH_2_*-Ph], 40.4 [C_δ_], 43.2 [CH_2_ (Pbf)], 43.4 [CH_2_ (NH*Bn*)], 44.0 [C_3_], 49.8 [CH_2_ (N*Bn*)], 51.7 [C_2_-*CH*], 52.2 [C_α_], 54.4 [C_6_], 56.5 [*CH_2_*CO], 59.4 [C_2_], 79.9 [C (Boc)], 86.4, 117.5, 124.6 [3C (Pbf)], 126.7, 127.2, 127.7, 127.9, 128.3, 128.5, 128.9, 129.1 [15CH (Ar)], 132.3 [2C (Pbf)], 136.1 [C (N*Bn*)], 136.9 [C (Ph)], 138.2 [C (NH*Bn*)], 138.4 [C (Pbf)], 155.5 [CO (Boc)], 156.3 [C (NH*C*(NH_2_) = N)], 158.8 [C (Pbf)], 167.3 [C_5_], 169.9 [CO], 171.3 [α-CONH]. (***S***)-**15c** *δ* (ppm): 12.4, 18.0, 19.3 [3CH_3_ (Pbf)], 28.2 [3CH_3_ (Boc)], 28.6 [2CH_3_ (Pbf)], 30.9 [C_β_], 37.6 [*CH_2_*-Ph], 43.2 [CH_2_ (Pbf)], 43.3 [CH_2_ (NH*Bn*)], 44.0 [C_3_], 49.6 [CH_2_ (N*Bn*)], 51.7 [C_2_-*CH*], 52.2 [C_α_], 79.9 [C (Boc)], 86.4, 117.5, 124.6 [3C (Pbf)], 126.7, 127.2, 127.6, 127.9, 128.3, 128.6, 128.9, 129.1 [15CH (Ar)], 132.3 [2C (Pbf)], 136.1 [C (N*Bn*)], 136.9 [C (Ph)], 138.2 [C (NH*Bn*)], 138.4 [C (Pbf)], 155.5 [CO (Boc)], 156.3 [C (NH*C*(NH_2_) = N)], 158.8 [C (Pbf)], 171.3 [α-CONH]; ES-MS *m/z* 966.8 [M+1]^+^; C_52_H_68_N_8_O_8_S (%): C: 64.71, H: 7.10, N: 11.61. Found (%): C: 64.58, H: 7.26, N: 11.81.

### 3.10. Synthesis of the Hydrochlorides **16a**–**c**

These compounds were obtained by applying the above indicated method of *N*-Boc removal.

N*-[2-[4-Benzyl-(2*RS*)-[(1*S*)-amino-2-phenylethyl]-5-oxo-piperazin-1-yl]acetyl]-Orn(Z)-NHBn hydrochloride* (**16a**). Amorphous solid (445 mg, 100%); HPLC *t*_R_: 16.76 min; ^1^H-NMR (500 MHz, DMSO-*d_6_*) (***R***)-**16a** *δ* (ppm): 1.40 (m, 1H, γ-H), 1.47 (m, 1H, γ-H), 1.57 (m, 1H, β-H), 1.70 (m, 1H, β-H), 2.85 (m, 1H, *CH_2_*-Ph), 2.90 (m, 1H, *CH_2_*-Ph), 2.98 (m, 1H, 2-H and δ-H), 3.21 (d, 1H, *J* = 17 Hz, 6-H), 3.30 (m, 2H, *CH_2_*CO), 3.46 (m, 2H, 3-H), 3.56 (d, 1H, *J* = 17 Hz, 6-H), 3.70 (m, 1H, 2-*CH*), 4.28 [m, 2H, CH_2_ (NH*Bn*)], 4.30 (m, 1H, α-H), 4.45 [d, 1H, *J* = 15 Hz, CH_2_ (N*Bn*)], 4.62 [d, 1H, *J* = 15 Hz, CH_2_ (N*Bn*)], 4.97 [m, 2H, CH_2_ (Z)], 7.11–7.37 (m, 21H, Ar and *NH*Z), 8.11 (m, 3H, NH_2_·HCl), 8.19 (d, 1H, *J* = 8 Hz, α-NH), 8.54 (m, 1H, *NH*Bn). (***S***)-**16a** *δ* (ppm): 1.40 (m, 1H, γ-H), 1.47 (m, 1H, γ-H), 1.57 (m, 1H, β-H), 1.70 (m, 1H, β-H), 3.15 (m, 1H, 2-H), 3.45 (m, 1H, 6-H), 3.46 (m, 2H, 3-H), 3.56 (m, 1H, 6-H), 3.60 (m, 1H, 2-*CH*), 4.28 [m, 3H, CH_2_ (NH*Bn*) and CH_2_ (N*Bn*)], 4.30 (m, 1H, α-H), 4.38 [d, 1H, *J* = 15 Hz, CH_2_ (N*Bn*)], 4.97 [m, 2H, CH_2_ (Z)], 7.11–7.37 (m, 21H, Ar and *NH*Z), 8.11 (m, 3H, NH_2_·HCl), 8.43 (d, 1H, *J* = 8 Hz, α-NH), 8.52 (m, 1H, *NH*Bn); ^13^C-NMR (125 MHz, DMSO-*d_6_*) (***R***)-**16a** *δ* (ppm): 26.0 [C_γ_], 29.5 [C_β_], 34.6 [*CH_2_*-Ph], 40.5 [C_δ_], 42.0 [CH_2_ (NH*Bn*)], 43.5 [C_3_], 48.8 [CH_2_ (N*Bn*)], 51.7 [C_2_-*CH*], 52.3 [C_α_], 54.7 [C_6_], 56.1 [*CH_2_*CO], 57.3 [C_2_], 65.1 [CH_2_ (Z)], 126.8, 127.0, 127.7, 127.8, 128.3, 127.4, 128.6, 129.2 [20CH (Ar)], 135.8 [C (Ph)], 137.0 [C (N*Bn*)], 137.2 [C (Z)], 139.2 [C (NH*Bn*)], 156.1 [CO (Z)], 167.1 [C_5_], 169.4 [CO], 171.4 [α-CONH]. (***S***)-**16a** *δ* (ppm): 26.2 [C_γ_], 29.3 [C_β_], 42.0 [CH_2_ (NH*Bn*)], 43.4 [C_3_], 48.9 [CH_2_ (N*Bn*)], 51.3 [C_2_-*CH*], 52.5 [C_α_], 54.7 [C_6_], 56.1 [*CH_2_*CO], 58.6 [C_2_], 65.1 [CH_2_ (Z)], 126.9, 127.4, 127.7, 127.8, 128.3, 127.4, 128.6, 129.3 [20CH (Ar)], 135.9 [C (Ph)], 136.9 [C (N*Bn*)], 137.2 [C (Z)], 139.2 [C (NH*Bn*)], 156.1 [CO (Z)], 167.1 [C_5_], 169.4 [CO], 171.4 [α-CONH]; ES-MS *m/z* [M+1]^+^ calculated for C_41_H_48_N_6_O_5_: 706.3; found: 706.5.

N*-[2-[4-Benzyl-(2*RS*)-[(1*S*)-amino-2-phenylethyl]-5-oxo-piperazin-1-yl]acetyl]-Lys(Z)-NHBn hydrochloride* (**16b**). Amorphous solid (453 mg, 100%); HPLC *t*_R_: 16.93 min; ^1^H-NMR (500 MHz, DMSO-*d_6_*) (***R***)-**16b** *δ* (ppm): 1.22 (m, 1H, γ-H), 1.27 (m, 1H, γ-H), 1.41 (m, 2H, δ-H), 1.58 (m, 1H, β-H), 1.70 (m, 1H, β-H), 2.83 (dd, 1H, *J* = 8 and 14 Hz, *CH_2_*-Ph), 2.94 (m, 2H, ε-H), 2.96 (m, 2H, 2-H and *CH_2_*-Ph), 3.20 (d, 1H, *J* = 17 Hz, 6-H), 3.24 (d, 1H, *J* = 16.5 Hz, *CH_2_*CO), 3.29 (d, 1H, *J* = 16.5 Hz, *CH_2_*CO), 3.38 (m, 1H, 3-H), 3.46 (m, 1H, 3-H), 3.53 (d, 1H, *J* = 17 Hz, 6-H), 3.72 (m, 1H, 2-*CH*), 4.26 [m, 2H, CH_2_ (NH*Bn*)], 4.28 (m, 1H, α-H), 4.45 [1d, 1H, J = 15 Hz, CH2 (NBn)], 4.63 [1d, 1H, J = 15 Hz, CH2 (NBn)], 4.98 [m, 2H, CH_2_ (Z)], 7.16–7.37 (m, 21H, Ar and *NH*Z), 8.19 (m, 3H, NH_2_·HCl), 8.21 (m, 1H, α-NH), 8.57 (t, 1H, *J* = 6 Hz, *NH*Bn). (***S***)-**16b** *δ* (ppm): 1.22 (m, 1H, γ-H), 1.27 (m, 1H, γ-H), 1.41 (m, 2H, δ-H), 1.58 (m, 1H, β-H), 1.70 (m, 1H, β-H), 3.15 (m, 1H, 2-H), 3.38 (m, 1H, 3-H), 3.46 (m, 1H, 3-H), 3.55 (m, 1H, 2-*CH*), 4.26 [m, 2H, CH_2_ (NH*Bn*)], 4.28 [m, 2H, α-H and CH_2_ (N*Bn*)], 4.40 [d, 1H, *J* = 15 Hz, CH_2_ (N*Bn*)], 4.98 [m, 2H, CH_2_ (Z)], 7.16–7.37 (m, 21H, Ar and *NH*Z), 8.19 (m, 3H, NH_2_·HCl), 8.43 (d, 1H, *J* = 8 Hz, α-NH), 8.57 (m, 1H, *NH*Bn); ^13^C-NMR (125 MHz, DMSO-*d_6_*) (***R***)-**16b** *δ* (ppm): 23.2 [C_γ_], 29.5 [C_δ_], 32.0 [C_β_], 35.0 [*CH_2_*-Ph], 41.1 [C_ε_], 42.4 [CH_2_ (NH*Bn*)], 44.0 [C_3_], 49.2 [CH_2_ (N*Bn*)], 52.1 [C_2_-*CH*], 53.1 [C_α_], 55.0 [C_6_], 56.6 [*CH_2_*CO], 57.7 [C_2_], 65.5 [CH_2_ (Z)], 127.1, 127.5, 128.1, 128.2, 128.7, 128.8, 129.0, 129.7 [20CH (Ar)], 136.2 [C (Ph)], 137.4 [C (N*Bn*)], 137.7 [C (Z)], 139.8 [C (NH*Bn*)], 156.5 [CO (Z)], 167.5 [C_5_], 169.8 [CO], 172.0 [α-CONH]. (***S***)-**16b** *δ* (ppm): 23.3 [C_γ_], 31.9 [C_β_], 42.4 [CH_2_ (NH*Bn*)], 43.8 [C_3_], 49.3 [CH_2_ (N*Bn*)], 51.7 [C_2_-*CH*], 53.3 [C_α_], 59.1 [C_2_], 65.5 [CH_2_ (Z)], 127.3, 127.5, 127.8, 128.2, 128.7, 129.0, 129.1, 129.7 [20CH (Ar)], 136.3 [C (Ph)], 137.3 [C (N*Bn*)], 137.7 [C (Z)], 139.2 [C (NH*Bn*)], 162.3 [CO (Z)], 172.0 [α-CONH]; ES-MS *m/z* [M+1]^+^ calculated for C_42_H_50_N_6_O_5_: 720.5; found: 720.8.

N*-[2-[4-Benzyl-(2*RS*)-[(1*S*)-amino-2-phenylethyl]-5-oxo-piperazin-1-yl]acetyl]-Arg(Pbf)-NHBn hydrochloride* (**16c**). Amorphous solid (541 mg, 100%); HPLC *t*_R_: 14.80 min [(***R***)-**16c**] and 19.68 min [(***S***)-**16c**]; ^1^H-NMR (500 MHz, DMSO-*d_6_*) (***R***)-**16c** *δ* (ppm): 1.38 [s, 6H, 2CH_3_ (Pbf)], 1.44 (m, 2H, γ-H), 1.56 (m, 1H, β-H), 1.70 (m, 1H, β-H), 1.98 [s, 3H, CH_3_ (Pbf)], 2.40 [s, 3H, CH_3_ (Pbf)], 2.46 [s, 3H, CH_3_ (Pbf)], 2.83 (d, 1H, *J* = 6.5 and 14 Hz, *CH_2_*-Ph), 2.94 (m, 1H, *CH_2_*-Ph), 2.95 (m, 1H, 2-H), 2.96 [m, 2H, CH_2_ (Pbf)], 3.02 (dd, 2H, *J* = 6.5 and 12 Hz, δ-H), 3.19 (d, 1H, *J* = 16.5 Hz, 6-H), 3.29 (m, 2H, *CH_2_*CO), 3.39 (m, 1H, 3-H), 3.44 (m, 1H, 3-H), 3.55 (d, 1H, *J* = 16.5 Hz, 6-H), 3.65 (m, 1H, 2-*CH*), 4.23 [m, 2H, CH_2_ (NH*Bn*)], 4.30 (m, 1H, α-H), 4.45 [d, 1H, *J* = 15 Hz, CH_2_ (N*Bn*)], 4.63 [d, 1H, *J* = 15 Hz, CH_2_ (N*Bn*)], 6.45 [m, 3H, NHC(NH_2_) = N], 6.91–7.37 (m, 15H, Ar), 8.18 (m, 3H, NH_2_·HCl), 8.23 (d, 1H, *J* = 8 Hz, α-NH), 8.59 (t, 1H, *J* = 6Hz, *NH*Bn). (***S***)-**16c** *δ* (ppm): 1.38 [s, 6H, 2CH_3_ (Pbf)], 1.98 [s, 3H, CH_3_ (Pbf)], 2.40 [s, 3H, CH_3_ (Pbf)], 2.46 [s, 3H, CH_3_ (Pbf)], 2.96 [m, 2H, CH_2_ (Pbf)], 3.15 (m, 1H, 2-H), 3.39 (m, 1H, 3-H), 3.44 (m, 1H, 3-H), 3.55 (m, 1H, 2-*CH*), 4.23 [m, 2H, CH_2_ (NH*Bn*)], 4.30 (m, 2H, α-H and CH_2_ (N*Bn*)), 4.40 [d, 2H, *J* = 15 Hz, CH_2_ (N*Bn*)], 6.45 [m, 3H, NHC(NH_2_) = N], 6.91–7.37 (m, 15H, Ar), 8.18 (m, 3H, NH_2_·HCl), 8.46 (d, 1H, *J* = 8 Hz, α-NH), 8.56 (m, 1H, *NH*Bn); ^13^C-NMR (125 MHz, DMSO-*d_6_*) (***R***)-**16c** *δ* (ppm): 12.7, 18.1, 19.4 [3CH_3_ (Pbf)], 26.1 [C_γ_], 28.2 [2CH_3_ (Pbf)], 29.9 [C_β_], 35.0 [*CH_2_*-Ph], 40.3 [C_δ_], 42.4 [CH_2_ (NH*Bn*)], 42.9 [CH_2_ (Pbf)], 44.0 [C_3_], 49.2 [CH_2_ (N*Bn*)], 52.1 [C_2_-*CH*], 52.8 [C_α_], 55.0 [C_6_], 56.7 [*CH_2_*CO], 57.8 [C_2_], 86.8, 116.8, 124.8 [4C (Pbf)], 127.1, 127.5, 127.8, 128.2, 128.7, 129.0, 129.7 [15CH (Ar)], 131.9, 134.5 [2C (Pbf)], 136.2 [C (Ph)], 137.7 [C (N*Bn*)], 137.8 [C (Pbf)], 139.7 [C (NH*Bn*)], 156.5 [C (NHC(NH_2_) = N)], 158.0 [C (Pbf)], 167.6 [C_5_], 169.9 [CO], 171.8 [α-CONH]. (***S***)-**16c** *δ* (ppm): 12.7, 18.1, 19.4 [3CH_3_ (Pbf)], 28.2 [2CH_3_ (Pbf)], 42.4 [CH_2_ (NH*Bn*)], 42.9 [CH_2_ (Pbf)], 43.9 [C_3_], 49.1 [CH_2_ (N*Bn*)], 51.8 [C_2_-*CH*], 53.0 [C_α_], 59.1 [C_2_], 86.8, 116.8, 124.8 [4C (Pbf)], 127.3, 127.5, 127.8, 128.2, 128.7, 129.0, 129.1, 129.7 [15CH (Ar)], 131.9, 134.5 [2C (Pbf)], 136.3 [C (Ph)], 137.4 [C (N*Bn*)], 137.8 [C (Pbf)], 139.7 [C (NH*Bn*)], 156.5 [C (NHC(NH_2_) = N)], 158.0 [C (Pbf)], 167.5 [C_5_], 171.8 [α-CONH]; ES-MS *m/z* [M+1]^+^ calculated for C_47_H_60_N_8_O_6_S: 866.6; found: 866.0.

### 3.11. General Procedure for the Synthesis of the Piperazinone-Derived Ureas **17a**–c and **18a**,**b**

These compounds were obtained by applying the already indicated procedure for the synthesis of the urea analogues **9a**,**b** and **10a**,**b**.

N*-[2-[4-Benzyl-5-oxo-(2*RS*)-[2-phenyl-(1*S*)-(3-phenyl-ureido)ethyl]-piperazin-1-yl]acetyl]-Orn(Z)-NHBn* (**17a**). Amorphous solid (*R*:*S*) = (3:1)] (346 mg, 70%); HPLC *t*_R_: 23.73 min [(***R***)-**17a**] and 24.44 min [(***S***)-**17a**]; ^1^H-NMR (500 MHz, CDCl_3_) (***R***)-**17a** *δ* (ppm): 1.50 (m, 2H, γ-H), 1.70 (m, 1H, β-H), 1.80 (m, 1H, β-H), 2.69 (dd, 1H, *J* = 6 and 14 Hz, *CH_2_*-Ph), 2.88 (m, 1H, *CH_2_*-Ph), 2.94 (m, 1H, 2-H), 3.10 (m, 1H, δ-H), 3.20 (m, 2H, 3-H and 6-H), 3.35 (m, 1H, *CH_2_*CO), 3.38 (m, 1H, 3-H), 3.40 (m, 1H, *CH_2_*CO), 3.42 (m, 1H, δ-H), 3.49 (d, 1H, *J* = 17 Hz, 6-H), 4.07 (m, 1H, 2-*CH*), 4.18 [m, 1H, CH_2_ (NH*Bn*)], 4.32 [m, 1H, CH_2_ (N*Bn*)], 4.40 [m, 1H, CH_2_ (NH*Bn*)], 4.78 [m, 2H, α-H and CH_2_ (Z)], 4.82 [m, 1H, CH_2_ (N*Bn*)], 4.87 [m, 1H, CH_2_ (Z)], 5.09 (t, 1H, *J* = 6 Hz, *NH*Z), 5.57 (m, 1H, 2-CH*NH*), 6.84–7.30 (m, 26H, Ar and *NH*Ph), 7.34 (m, 1H, *NH*Bn), 7.79 (m, 1H, α-NH). (***S***)-**17a** *δ* (ppm): 1.40 (m, 2H, γ-H), 1.70 (m, 1H, β-H), 1.80 (m, 1H, β-H), 2.81 (m, 1H, *CH_2_*-Ph), 2.94 (m, 1H, *CH_2_*-Ph), 3.05 (m, 1H, δ-H), 3.08 (m, 1H, 3-H), 3.35 (m, 1H, *CH_2_*CO), 3.39 (m, 1H, 3-H), 3.40 (m, 1H, *CH_2_*CO), 3.43 (m, 1H, δ-H), 4.07 (m, 1H, 2-*CH*), 4.16 [dd, 1H, *J* = 5 and 15 Hz, CH_2_ (NH*Bn*)], 4.38 [m, 1H, CH_2_ (NH*Bn*)], 4.50 [m, 2H, CH_2_ (N*Bn*)], 4.72 (m, 1H, α-H), 4.76 [m, 1H, CH_2_ (Z)], 4.85 [m, 1H, CH_2_ (Z)], 4.96 (m, 1H, *NH*Z), 5.39 (d, 1H, *J* = 6.5 Hz, 5-CH*NH*), 6.84–7.30 (m, 26H, Ar and *NH*Ph), 7.41 (m, 1H, *NH*Bn), 7.73 (m, 1H, α-NH); ^13^C-NMR (125 MHz, CDCl_3_) (***R***)-**17a** *δ* (ppm): 26.6 [C_γ_], 30.1 [C_β_], 36.9 [*CH_2_*-Ph], 39.1 [C_δ_], 42.0 [CH_2_ (NH*Bn*)], 44.8 [C_3_], 49.5 [CH_2_ (N*Bn*)], 51.2 [C_α_], 52.3 [C_2_-*CH*], 55.8 [C_6_], 59.0 [*CH_2_*CO], 61.2 [C_2_], 66.7 [CH_2_ (Z)], 120.0, 123.1, 126.7, 127.5, 127.8, 127.9, 128.1, 128.3, 128.5, 128.8 [25CH (Ar)], 136.1 [C (N*Bn*)], 136.2 [C (Z)], 137.2 [C (Ph)], 137.6 [C (NH*Bn*)], 138.7 [C (NH*Ph*)], 155.1 [CO (Z)], 157.5 [CO (Urea)], 167.9 [C_5_], 170.4 [CO], 172.7 [α-CONH]. (***S***)-**17a** *δ* (ppm): 26.5 [C_γ_], 30.6 [C_β_], 37.6 [*CH_2_*-Ph], 38.8 [C_δ_], 42.1 [CH_2_ (NH*Bn*)], 44.8 [C_3_], 49.9 [CH_2_ (N*Bn*)], 50.6 [C_α_], 51.9 [C_2_-*CH*], 66.7 [CH_2_ (Z)], 118.9, 122.3, 126.7, 127.5, 127.8, 127.9, 128.2, 128.5, 128.6, 128.7, 128.9, 129.4 [25CH (Ar)], 136.1 [C (N*Bn*)], 136.2 [C (Z)], 137.2 [C (Ph)], 137.4 [C (NH*Bn*)], 139.5 [C (NH*Ph*)], 155.0 [CO (Z)], 157.6 [CO (Urea)], 166.9 [C_5_], 169.7 [CO], 172.9 [α-CONH]; ES-MS *m/z* 825.7 [M+1]^+^; C_48_H_53_N_7_O_6_ (%): C: 69.97, H: 6.48, N: 11.90. Found (%): C: 69.75, H: 6.65, N: 12.02.

N*-[2-[4-Benzyl-5-oxo-(2*RS*)-[2-phenyl-(1*S*)-(3-phenyl-ureido)ethyl]-piperazin-1-yl]acetyl]-Lys(Z)-NHBn* (**17b**). Amorphous solid [(*R*:*S*) = (3:1)] (327 mg, 65%); HPLC *t*_R_: 24.06 min; ^1^H-NMR (500 MHz, CDCl_3_) (***R***)-**17b** *δ* (ppm): 1.33 (m, 2H, γ-H), 1.46 (m, 2H, δ-H), 1.67 (m, 1H, β-H), 1.85 (m, 1H, β-H), 2.68 (m, 1H, *CH_2_*-Ph), 2.82 (m, 1H, *CH_2_*-Ph), 2.89 (m, 1H, 2-H), 3.05 (m, 2H, ε-H), 3.15 (m, 1H, 6-H), 3.25 (m, 2H, 3-H), 3.31 (m, 2H, *CH_2_*CO), 3.53 (d, 1H, *J* = 16.5 Hz, 6-H), 4.20 (m, 1H, 2-*CH*), 4.25 [m, 3H, CH_2_ (NH*Bn* and N*Bn*)], 4.44 (m, 1H, α-H), 4.77 [d, 1H, *J* = 14.5 Hz, CH_2_ (N*Bn*], 5.00 [s, 2H, CH_2_ (Z)], 5.23 (m, 1H, *NH*Z), 5.45 (m, 1H, 2-CH*NH*), 6.84–7.52 (m, 27H, Ar, *NH*Bn and *NH*Ph), 7.79 (m, 1H, α-NH). (***S***)-**17b** *δ* (ppm): 2.78 (m, 1H, *CH_2_*-Ph), 2.89 (m, 1H, *CH_2_*-Ph), 2.94 (m, 1H, 2-H), 4.20 (m, 1H, 2-*CH*), 4.25 [m, 2H, CH_2_ (NH*Bn*)], 4.52 [d, 1H, *J* = 14.5 Hz, CH_2_ (N*Bn*], 4.58 [d, 1H, *J* = 14.5 Hz, CH_2_ (N*Bn*], 5.03 [m, 2H, CH_2_ (Z)], 5.09 (m, 1H, *NH*Z), 5.55 (m, 1H, 2-CH*NH*), 6.84–7.52 (m, 27H, Ar, *NH*Bn and *NH*Ph), 7.89 (m, 1H, α-NH); ^13^C-NMR (125 MHz, CDCl_3_) (***R***)-**17b** *δ* (ppm): 22.7 [C_γ_], 29.1 [C_δ_], 31.9 [C_β_], 37.2 [*CH_2_*-Ph], 40.3 [C_ε_], 43.6 [CH_2_ (NH*Bn*)], 44.6 [C_3_], 49.5 [CH_2_ (N*Bn*)], 52.0 [C_2_-*CH*], 53.0 [C_α_], 55.6 [C_6_], 58.8 [*CH_2_*CO], 60.9 [C_2_], 66.0 [CH_2_ (Z)], 120.1, 123.2, 126.8, 127.5, 128.0, 128.1, 128.4, 128.5, 128.7, 128.8, 128.9 [25CH (Ar)], 136.2 [C (N*Bn*)], 136.5 [C (Z)], 137.0 [C (Ph)], 137.7 [C (NH*Bn*)], 138.6 [C (NH*Ph*)], 155.3 [CO (Z)], 156.6 [CO (Urea)], 167.6 [C_5_], 170.1 [CO], 172.3 [α-CONH]. (***S***)-**17b** *δ* (ppm): 43.6 [CH_2_ (NH*Bn*)], 44.6 [C_3_], 52.1 [C_2_-*CH*], 60.8 [C_2_], 66.0 [CH_2_ (Z)], 120.1, 123.2, 126.8, 127.5, 128.0, 128.1, 128.4, 128.5, 128.7, 128.8, 129.0 [25CH (Ar)], 136.5 [C (Z)], 156.4 [CO (Z)], 172.3 [α-CONH]; ES-MS *m/z* 839.7 [M+1]^+^; C_49_H_55_N_7_O_6_ (%): C: 70.23, H: 6.62, N: 11.70. Found (%): C: 70.46, H: 6.75, N: 11.54.

N*-[2-[4-Benzyl-5-oxo-(2*RS*)-[2-phenyl-(1*S*)-(3-phenyl-ureido)ethyl]-piperazin-1-yl]acetyl]-Arg(Pbf)-NHBn* (**17c**). Amorphous solid [(*R*:*S)* = (3:1)] (443 mg, 75%); HPLC *t*_R_: 25.80 min [(***R***)-**17c**] and 23.82 min [(***S***)-**17c**]; ^1^H-NMR (500 MHz, CDCl_3_) (***R***)-**17c** *δ* (ppm): 1.45 [s, 3H, CH_3_ (Pbf)], 1.46 [s, 3H, CH_3_ (Pbf)], 1.40 (m, 2H, γ-H), 1.52 (m, 1H, β-H), 1.68 (m, 1H, β-H), 2.10 [s, 3H, CH_3_ (Pbf)], 2.50 [s, 3H, CH_3_ (Pbf)], 2.58 [s, 3H, CH_3_ (Pbf)], 2.64 (m, 1H, 2-H), 2.68 (m, 1H, *CH_2_*-Ph), 2.77 (m, 1H, *CH_2_*-Ph), 2.94 [s, 2H, CH_2_ (Pbf)], 2.98 (m, 1H, *CH_2_*CO), 3.06 (m, 1H, 6-H), 3.25 (m, 1H, δ-H), 3.28 (dd, 1H, *J* = 5 and 13 Hz, 3-H), 3.29 (m, 1H, δ-H), 3.50 (d, 1H, *J* = 15.5 Hz, *CH_2_*CO), 3.58 (m, 1H, 3-H), 3.64 (d, 1H, *J* = 16.5 Hz, 6-H), 4.13 [d, 1H, *J* = 14.5 Hz, CH_2_ (N*Bn*)], 4.30 (m, 1H, 2-*CH*), 4.32 (m, 1H, α-H), 4.36 [m, 2H, CH_2_ (NH*Bn*)], 5.00 [d, 1H, *J* = 14.5 Hz, CH_2_ (N*Bn*)], 5.98 (m, 1H, 2-CH*NH*), 6.18 [m, 2H, NHC(*NH_2_*) = N], 6.36 [m, 1H, *NH*C(NH_2_) = N], 6.86–7.37 (m, 21H, Ar and *NH*Ph), 7.64 (m, 1H, *NH*Bn), 7.88 (d, 1H, *J* = 8 Hz, α-NH). (***S***)-**17c** *δ* (ppm): 1.45 [s, 3H, CH_3_ (Pbf)], 1.46 [s, 3H, CH_3_ (Pbf)], 2.10 [s, 3H, CH_3_ (Pbf)], 2.50 [s, 3H, CH_3_ (Pbf)], 2.58 [s, 3H, CH_3_ (Pbf)], 2.68 (m, 1H, *CH_2_*-Ph), 2.77 (m, 1H, *CH_2_*-Ph), 2.94 [s, 2H, CH_2_ (Pbf)], 3.23 (m, 1H, δ-H), 3.29 (m, 1H, δ-H), 3.28 (m, 1H, 3-H), 3.58 (m, 1H, 3-H), 4.05 [m, 1H, CH_2_ (NH*Bn*)], 4.29 (m, 1H, α-H), 4.30 [m, 1H, CH_2_ (NH*Bn*)], 4.46 [d, 1H, *J* = 14 Hz, CH_2_ (N*Bn*)], 4.59 [d, 1H, *J* = 14 Hz, CH_2_ (N*Bn*)], 5.98 (m, 1H, 2-CH*NH*), 6.18 [m, 2H, NHC(*NH_2_*) = N], 6.36 [m, 1H, *NH*C(NH_2_) = N], 6.86–7.37 (m, 21H, Ar and *NH*Ph), 7.64 (m, 1H, *NH*Bn), 7.78 (d, 1H, *J* = 8 Hz, α-NH); ^13^C-NMR (125 MHz, CDCl_3_) (***R***)-**17c** *δ* (ppm): 12.5, 18.0, 19.4 [3CH_3_ (Pbf)], 25.4 [C_γ_], 28.6 [2CH3 (Pbf)], 29.3 [C_β_], 38.1 [*CH_2_*-Ph], 40.3 [C_δ_], 43.2 [2CH2 (Pbf and NHBn)], 44.3 [C_3_], 49.2 [CH_2_ (N*Bn*)], 51.2 [C_2_-*CH*], 53.1 [C_α_], 55.4 [C_6_], 59.5 [*CH_2_*CO], 60.2 [C_2_], 86.6, 117.8, 124.9 [3C (Pbf)], 119.5, 122.7, 126.7, 127.1, 127.3, 127.9, 128.1, 128.5, 128.6, 129.0, 129.3 [20CH (Ar)], 132.2 [2C (Pbf)], 136.2 [C (N*Bn*)], 137.2 [C (Ph)], 138.1 [C (NH*Bn*)], 138.3 [C (Pbf)], 139.0 [C (NH*Ph*)], 156.2 [CO (Urea)], 156.4 [C (NHC(NH_2_) = N)], 159.0 [C (Pbf)], 168.3 [C_5_], 171.0 [CO], 172.0 [α-CONH]. (***S***)-**17c** *δ* (ppm): 12.5, 18.0, 19.4 [3CH3 (Pbf)], 28.6 [2CH_3_ (Pbf)], 38.4 [*CH_2_*-Ph], 40.3 [C_δ_], 43.2 [2CH_2_ (Pbf and NHBn)], 44.2 [C_3_], 49.2 [CH_2_ (N*Bn*)], 53.1 [C_α_], 86.6, 117.8, 124.9 [3C (Pbf)], 118.9, 122.2, 126.6, 127.1, 127.2, 127.9, 128.1, 128.4, 128.7, 129.0, 129.3 [20CH (Ar)], 132.2 [2C (Pbf)], 136.1 [C (N*Bn*)], 137.1 [C (Ph)], 138.0 [C (NH*Bn*)], 138.3 [C (Pbf)], 139.4 [C (NH*Ph*)], 156.4 [C (NHC(NH_2_) = N)], 159.0 [C (Pbf)], 172.0 [α-CONH]; ES-MS *m/z* 985.1 [M+1]^+^; C_54_H_65_N_9_O_7_S (%): C: 65.90, H: 6.66, N: 12.81. Found (%): C: 65.72, H: 6.90, N: 12.63.

N*-[2-[4-Benzyl-(2*RS*)-[(1*S*)-(3-benzylureido)-2-phenyl-ethyl]-5-oxopiperazin-1-yl]acetyl]-Orn(Z)-NHBn* (**18a**). Amorphous solid [(*R*:*S*) = (3:1)] (375 mg, 65%); HPLC *t*_R_: 23.30 min [(***R***)-**18a**] and 23.82 min [(***S***)-**18a**]; ^1^H-NMR (500 MHz, CDCl_3_) (***R***)-**18a** *δ* (ppm): 1.52 (m, 2H, γ-H), 1.65 (m, 1H, β-H), 1.82 (m, 1H, β-H), 2.69 (m, 1H, *CH_2_*-Ph), 2.88 (m, 1H, *CH_2_*-Ph), 2.90 (m, 1H, 2-H), 3.11 (m, 1H, δ-H), 3.20 (m, 1H, *CH_2_*CO), 3.23 (m, 1H, 3-H), 3.32 (m, 2H, *CH_2_*CO and 6-H), 3.36 (m, 1H, 3-H), 3.42 (m, 1H, δ-H), 3.55 (m, 1H, 6-H), 4.05 [m, 1H, CH_2_ (NH*Bn*)], 4.08 [m, 1H, CH_2_ (NH*Bn*, Urea)], 4.15 [m, 1H, CH_2_ (NH*Bn*, Urea)], 4.18 (m, 1H, 2-*CH*), 4.25 [m, 1H, CH_2_ (NH*Bn*)], 4.32 [m, 1H, CH_2_ (N*Bn*)], 4.68 (m, 1H, α-H), 4.75 [m, 1H, CH_2_ (Z)], 4.79 [m, 1H, CH_2_ (N*Bn*)], 4.88 [d, 1H, *J* = 12.5 Hz, CH_2_ (Z)], 5.08 (m, 1H, 2-CH*NH*), 5.20 (m, 1H, *NH*Z), 5.95 [m, 1H, *NH*Bn (Urea)], 6.95–7.40 (m, 25H, Ar), 7.40 (m, 1H, *NH*Bn), 7.86 (d, 1H, *J* = 9 Hz, α-NH). (***S***)-**18a** *δ* (ppm): 1.46 (m, 2H, γ-H), 1.63 (m, 1H, β-H), 1.80 (m, 1H, β-H), 2.57 (m, 1H, *CH_2_*-Ph), 2.85 (m, 1H, *CH_2_*-Ph), 3.04 (m, 1H, 3-H), 3.07 (m, 1H, δ-H), 3.34 (m, 1H, 3-H), 3.38 (m, 1H, 6-H), 3.44 (m, 1H, δ-H), 3.57 (m, 1H, 6-H), 3.96 [dd, 1H, *J* = 5 and 15 Hz, CH_2_ (NH*Bn*)], 4.02 (m, 1H, 2-*CH*), 4.08 [m, 1H, CH_2_ (NH*Bn*, Urea)], 4.15 [m, 1H, CH_2_ (NH*Bn*, Urea)], 4.20 [m, 1H, CH_2_ (NH*Bn*)], 4.50 [m, 1H, CH_2_ (N*Bn*)], 4.66 [m, 1H, CH_2_ (Z)], 4.68 (m, 1H, α-H), 4.79 [m, 1H, CH_2_ (N*Bn*)], 4.82 [m, 1H, CH_2_ (Z)], 5.02 (t, 1H, *J* = 6 Hz, *NH*Z), 5.08 (m, 1H, 5-CH*NH*), 5.95 [m, 1H, *NH*Bn (Urea)], 6.95–7.40 (m, 25H, Ar), 7.40 (m, 1H, *NH*Bn), 7.78 (d, 1H, *J* = 8.5 Hz, α-NH); ^13^C-NMR (125 MHz, CDCl_3_) (***R***)-**18a** *δ* (ppm): 26.6 [C_γ_], 30.2 [C_β_], 37.3 [*CH_2_*-Ph], 39.0 [C_δ_], 43.5 [CH_2_ (NH*Bn*)], 44.0 [CH_2_ (NH*Bn*, Urea)], 44.6 [C_3_], 49.5 [CH_2_ (N*Bn*)], 51.1 [C_α_], 52.2 [C_2_-*CH*], 55.6 [C_6_ and *CH_2_*CO], 60.8 [C_2_], 66.7 [CH_2_ (Z)], 126.6, 127.0, 127.4, 127.6, 127.9, 128.1, 128.4, 128.5, 128.7, 128.9 [25CH (Ar)], 136.3 [C (N*Bn*) and C (Z)], 137.2 [C (Ph)], 137.7 [C (NH*Bn*)], 139.3 [C (NH*Bn*, Urea)], 155.4 [CO (Z)], 157.7 [CO (Urea)], 167.6 [C_5_], 170.0 [CO], 172.4 [α-CONH]. (***S***)-**18a** *δ* (ppm): 26.6 [C_γ_], 30.7 [C_β_], 38.2 [*CH_2_*-Ph], 38.7 [C_δ_], 43.5 [CH_2_ (NH*Bn*)], 43.8 [CH_2_ (NH*Bn*, Urea)], 45.0 [C_3_], 49.9 [CH_2_ (N*Bn*)], 51.4 [C_α_], 52.2 [C_2_-*CH*], 55.6 [*CH_2_*CO], 66.7 [CH_2_ (Z)], 126.9, 127.1, 127.4, 127.5, 127.8, 127.9, 128.2, 128.5, 128.6, 128.9, 129.6 [25CH (Ar)], 136.1 [C (N*Bn*) and C (Z)], 137.1 [C (Ph)], 137.7 [C (NH*Bn*)], 139.8 [C (NH*Bn*, Urea)], 155.2 [CO (Z)], 157.9 [CO (Urea)], 167.6 [C_5_], 170.0 [CO], 172.6 [α-CONH]; ES-MS *m/z* 839.6 [M+1]^+^; C_49_H_55_N_7_O_6_ (%): C: 70.23, H: 6.22, N: 11.70. Found (%): C: 70.01, H: 6.46, N: 11.59.

N*-[2-[4-Benzyl-(2*RS*)-[(1*S*)-(3-benzylureido)-2-phenyl-ethyl]-5-oxopiperazin-1-yl]acetyl]-Lys(Z)-NHBn* (**18b**). Amorphous solid [(*R*:*S)* = (3:1)] (317 mg, 62%); HPLC *t*_R_: 23.69 min [(***R***)-**18b**] and 24.16 min [(***S***)-**19b**]; ^1^H-NMR (500 MHz, CDCl_3_) (***R***)-**18b** *δ* (ppm): 1.27 (m, 2H, γ-H), 1.40 (m, 2H, δ-H), 1.60 (m, 1H, β-H), 1.78(m, 1H, β-H), 2.64 (m, 2H, *CH_2_*-Ph), 2.76 (m, 1H, 2-H), 3.08 (m, 1H, ε-H), 3.12 (m, 2H, *CH_2_*CO and 6-H), 3.14 (m, 1H, 3-H), 3.15 (m, 1H, ε-H), 3.24 (m, 1H, *CH_2_*CO), 3.25 (m, 1H, 3-H), 3.41 (d, 1H, *J* = 16.5 Hz, 6-H), 4.10 (m, 1H, 2-*CH*), 3.95 [dd, 1H, *J* = 5.5 and 15, CH_2_ (NH*Bn*, Urea)], 4.04 [m, 1H, CH_2_ (NH*Bn*, Urea)], 4.18 [m, 2H, CH_2_ (NH*Bn*)], 4.32 [m, 1H, CH_2_ (N*Bn*], 4.38 (m, 1H, α-H), 4.62 [d, 1H, *J* = 14.5 Hz, CH_2_ (N*Bn*], 4.92 (m, 1H, 2-CH*NH*), 5.00 [s, 2H, CH_2_ (Z)], 5.20 (m, 1H, *NH*Z), 5.70 [m, 1H, *NH*Bn (Urea)], 6.81–7.33 (m, 26H, Ar and *NH*Bn), 7.71 (d, 1H, *J* = 8 Hz, α-NH). (***S***)-**18b** *δ* (ppm): 1.27 (m, 2H, γ-H), 1.40 (m, 2H, δ-H), 1.60 (m, 1H, β-H), 1.78(m, 1H, β-H), 2.68 (m, 2H, *CH_2_*-Ph), 2.76 (m, 1H, 2-H), 2.98 (m, 1H, 3-H), 3.10 (m, 1H, *CH_2_*CO), 3.18 (m, 1H, 3-H), 3.22 (m, 1H, *CH_2_*CO), 3.76 (m, 1H, 2-*CH*), 3.95 [m, 1H, CH_2_ (NH*Bn*, Urea)], 4.04 [m, 1H, CH_2_ (NH*Bn*, Urea)], 4.10 [m, 1H, CH_2_ (NH*Bn*)], 4.18 [m, 1H, CH_2_ (NH*Bn*)], 4.34 (m, 1H, α-H), 4.43 [d, 1H, *J* = 14.5 Hz, CH_2_ (N*Bn*], 4.52 [d, 1H, *J* = 14.5 Hz, CH_2_ (N*Bn*], 4.92 (m, 1H, 2-CH*NH*), 4.96 [s, 2H, CH_2_ (Z)], 5.10 (m, 1H, *NH*Z), 5.70 [m, 1H, *NH*Bn (Urea)], 6.81–7.33 (m, 26H, Ar and *NH*Bn), 7.64 (d, 1H, *J* = 7.5 Hz, α-NH); ^13^C-NMR (125 MHz, CDCl_3_) (***R***)-**18b** *δ* (ppm): 22.7 [C_γ_], 29.2 [C_δ_], 31.9 [C_β_], 37.5 [*CH_2_*-Ph], 40.4 [C_ε_], 43.4 [CH_2_ (NH*Bn*)], 44.0 [CH_2_ (NH*Bn*, Urea)], 44.3 [C_3_], 49.5 [CH_2_ (N*Bn*)], 52.0 [C_2_-*CH*], 53.0 [C_α_], 55.3 [C_6_], 58.6 [*CH_2_*CO], 60.1 [C_2_], 66.6 [CH_2_ (Z)], 126.7, 127.0, 127.4, 127.5, 128.0, 128.2, 128.4, 128.5, 128.6, 128.7, 128.9 [25CH (Ar)], 136.4 [C (N*Bn*)], 136.6 [C (Z)], 137.1 [C (Ph)], 137.8 [C (NH*Bn*)], 139.1 [C (NH*Bn*, Urea)], 156.6 [CO (Z)], 157.7 [CO (Urea)], 167.8 [C_5_], 170.1 [CO], 172.1 [α-CONH]. (***S***)-**18b** *δ* (ppm): 22.4 [C_γ_], 29.7 [C_δ_], 30.9 [C_β_], 38.2 [*CH_2_*-Ph], 43.5 [CH_2_ (NH*Bn*)], 43.9 [CH_2_ (NH*Bn*, Urea)], 44.3 [C_3_], 49.7 [CH_2_ (N*Bn*)], 51.2 [C_2_-*CH*], 52.8 [C_α_], 58.6 [*CH_2_*CO], 60.0 [C_2_], 66.7 [CH_2_ (Z)], 126.7, 127.2, 127.4, 127.5, 128.0, 128.1, 128.4, 128.5, 128.6, 128.7, 128.9, 129.5 [25CH (Ar)], 136.1 [C (N*Bn*)], 136.6 [C (Z)], 137.1 [C (Ph)], 137.8 [C (NH*Bn*)], 139.6 [C (NH*Bn*, Urea)], 156.7 [CO (Z)], 158.0 [CO (Urea)], 170.1 [CO], 172.1 [α-CONH]; ES-MS *m/z* 853.7 [M+1]^+^; C_50_H_57_N_7_O_6_ (%): C: 70.48, H: 6.74, N: 11.51. Found (%): C: 70.31, H: 6.95, N: 11.69.

### 3.12. General Procedure for the Synthesis of the Hydrochlorides **19a**,**b** and **20a**,**b**

These compounds were prepared following the general procedure for the removal of the *N*-Z protecting group, already indicated for the synthesis of **11a**,**b** and **12a**,**b**.

N*-[2-[4-Benzyl-5-oxo-(2*RS*)-[2-phenyl-(1*S*)-(3-phenylureido)ethyl]-piperazin-1-yl]acetyl]-Orn-NHBn hydrochloride* (**19a**). Amorphous solid [(*R*:*S)* = (3:1)] (145 mg, 100%); HPLC *t*_R_: 16.27 min [(***R***)-**19a**] and 16.52 min [(***S***)-**19a**]; ^1^H-NMR (500 MHz, DMSO-*d_6_*) (***R***)-**19a** *δ* (ppm): 1.62 (m, 3H, γ-H and β-H), 1.82 (m, 1H, β-H), 2.70 (m, 1H, *CH_2_*-Ph), 2.75 (m, 3H, δ-H and 2-H), 2.91 (d, 1H, *J* = 11 Hz, *CH_2_*-Ph), 3.33–4.11 (m, 6H, 3-H, 6-H and *CH_2_*CO), 4.22 [m, 1H, CH_2_ (NH*Bn*)], 4.34 [m, 1H, CH_2_ (NH*Bn*)], 4.38 (m, 1H, 2-*CH*), 4.39 (m, 1H, α-H), 4.48 [m, 1H, CH_2_ (N*Bn*)], 4.62 [m, 1H, CH_2_ (N*Bn*)], 6.80 (m, 1H, 2-CH*NH*), 6.76–6.95 (m, 2H, Ar), 7.08–7.38 (m, 18H, Ar), 7.96 (m, 3H, NH_2_·HCl), 8.60 (m, 1H, α-NH), 8.72 (m, 1H, *NH*Bn), 8.85 (m, 1H, *NH*Ph). (***S***)-**19a** *δ* (ppm): 1.58 (m, 3H, γ-H and β-H), 1.78 (m, 1H, β-H), 2.55 (dd, 1H, *J* = 6 and 14 Hz, *CH_2_*-Ph), 2.75 (m, 3H, δ-H and 2-H), 2.85 (m, 1H, *CH_2_*-Ph), 3.33–4.11 (m, 7H, 3-H, 6-H, *CH_2_*CO and 2-*CH*), 4.22 [m, 1H, CH_2_ (NH*Bn*)], 4.34 [m, 1H, CH_2_ (NH*Bn*)], 4.51 [m, 1H, CH_2_ (N*Bn*)], 4.64 [m, 1H, CH_2_ (N*Bn*)], 6.80 (m, 1H, 2-CH*NH*), 6.76–6.95 (m, 2H, Ar), 7.08–7.38 (m, 18H, Ar), 7.96 (m, 3H, NH_2_·HCl), 8.60 (m, 1H, α-NH), 8.70 (m, 1H, *NH*Bn), 8.81 (m, 1H, *NH*Ph); ^13^C-NMR (125 MHz, DMSO-*d_6_*) (***R***)-**19a** *δ* (ppm): 23.4 [C_γ_], 28.9 [C_β_], 37.8 [*CH_2_*-Ph], 38.1 [C_δ_], 42.0 [CH_2_ (NH*Bn*)], 43.7 [C_3_], 49.2 [CH_2_ (N*Bn*)], 49.8 [C_2_-*CH*], 51.9 [C_α_], 53.6 [C_6_], 53.9 [*CH_2_*CO], 60.5 [C_2_], 117.7, 121.2, 126.2, 126.6, 127.0, 127.2, 127.5, 128.2, 128.4, 128.5, 129.1 [20CH (Ar)], 136.3 [C (N*Bn*)], 137.8 [C (Ph)], 139.1 [C (NH*Bn*)], 139.9 [C (NH*Ph*)], 155.2 [CO (Urea)], 170.7 [α-CONH]. (***S***)-**19a** *δ* (ppm): 23.3 [C_γ_], 29.0 [C_β_], 37.8 [*CH_2_*-Ph], 38.2 [C_δ_], 42.0 [CH_2_ (NH*Bn*)], 43.7 [C_3_], 49.2 [CH_2_ (N*Bn*)], 49.8 [C_2_-*CH*], 53.5 [C_6_], 53.9 [*CH_2_*CO], 60.2 [C_2_], 117.5, 121.0, 126.1, 126.6, 127.0, 127.3, 127.7, 127.9, 128.1, 128.4, 128.5, 129.1 [20CH (Ar)], 136.3 [C (N*Bn*)], 138.0 [C (Ph)], 139.1 [C (NH*Bn*)], 140.1 [C (NH*Ph*)], 155.0 [CO (Urea)], 170.8 [α-CONH]; ES-MS *m/z* [M+2]^+^ calculated for C_40_H_47_N_7_O_4_: 690.3; found: 690.6.

N*-[2-[4-Benzyl-5-oxo-(2*RS*)-[2-phenyl-(1*S*)-(3-phenylureido)ethyl]-piperazin-1-yl}acetyl}-Lys-NHBn hydrochloride* (**19b**). Amorphous solid [(*R*:*S)* = (3:1)] (148 mg, 100%); HPLC *t*_R_: 16.44 min; ^1^H-NMR (500 MHz, DMSO-*d_6_*) (***R***)-**19b** *δ* (ppm): 1.30 (m, 2H, γ-H), 1.50 (m, 2H, δ-H), 1.60 (m, 1H, β-H), 1.72 (m, 1H, β-H), 2.70 (m, 3H, ε-H and 2-H), 2.72 (m, 1H, *CH_2_*-Ph), 2.92 (m, 1H, *CH_2_*-Ph), 3.26–4.20 (m, 6H, 3-H, 6-H and *CH_2_*CO), 4.32 (m, 1H, 2-*CH*), 4.24 [dd, 1H, *J* = 6 and 15 Hz, CH_2_ (NH*Bn*)], 4.30 [m, 2H, α-H and CH_2_ (NH*Bn*)], 4.50 [m, 1H, CH_2_ (N*Bn*)], 4.70 [m, 1H, CH_2_ (N*Bn*)], 6.55 (m, 1H, 2-CH*NH*), 6.86 (t, 1H, *J* = 7 Hz, Ar), 6.97–7.41 (m, 19H, Ar), 7.84 (m, 3H, NH_2_·HCl), 8.51 (m, 1H, α-NH), 8.62 (m, 1H, *NH*Bn), 8.80 (m, 1H, *NH*Ph). (***S***)-**19b** *δ* (ppm): 1.60 (m, 1H, β-H), 1.72 (m, 1H, β-H), 2.55 (m, 1H, *CH_2_*-Ph), 2.88 (m, 1H, *CH_2_*-Ph), 4.24 [m, 1H, CH_2_ (NH*Bn*)], 4.30 [m, 1H, CH_2_ (NH*Bn*)], 6.55 (m, 1H, 2-CH*NH*), 6.86 (t, 1H, *J* = 7 Hz, Ar), 6.97–7.41 (m, 19H, Ar), 7.84 (m, 3H, NH_2_·HCl), 8.58 (m, 1H, *NH*Bn), 8.83 (m, 1H, *NH*Ph); ^13^C-NMR (125 MHz, DMSO-*d_6_*) (***R***)-**19b** *δ* (ppm): 22.2 [C_γ_], 26.5 [C_δ_], 31.3 [C_β_], 37.8 [*CH_2_*-Ph], 38.4 [C_ε_], 42.0 [CH_2_ (NH*Bn*)], 43.8 [C_3_], 49.2 [CH_2_ (N*Bn*) and C_2_-*CH*], 52.5 [C_α_], 53.7 [C_6_], 60.4 [C_2_], 117.8, 121.3, 126.7, 127.0, 127.3, 127.6, 128.2, 128.5, 128.6, 129.2 [20CH (Ar)], 136.5 [C (N*Bn*)], 137.9 [C (Ph)], 139.2 [C (NH*Bn*)], 139.9 [C (NH*Ph*)], 155.3 [CO (Urea)], 171.1 [α-CONH]. (***S***)-**19b** *δ* (ppm): 31.5 [C_β_], 37.8 [*CH_2_*-Ph], 42.0 [CH_2_ (NH*Bn*)], 117.8, 121.3, 126.3, 127.0, 127.3, 127.6, 128.2, 128.5, 128.6, 129.2 [20CH (Ar)], 136.5 [C (N*Bn*)], 137.9 [C (Ph)], 139.2 [C (NH*Bn*)], 139.9 [C (NH*Ph*)], 155.5 [CO (Urea)], 171.1 [α-CONH]; ES-MS *m/z* [M+2]^+^ calculated for C_41_H_49_N_7_O_4_: 705.3; found: 705.6.

N*-[2-[4-Benzyl-(2*RS*)-[(1*S*)-(3-benzylureido)-2-phenylethyl]-5-oxopiperazin-1-yl]acetyl]-Orn-NHBn hydrochloride* (**20a**). Amorphous solid [(*R*:*S)* = (3:1)] (148 mg, 100%); HPLC *t*_R_: 16.32 min [(***R***)-**20a**] and 16.78 min [(***S***)-**20a**]; ^1^H-NMR (500 MHz, DMSO-*d_6_*) (***R***)-**20a** *δ* (ppm): 1.60 (m, 2H, γ-H), 1.65 (m, 1H, β-H), 1.75 (m, 1H, β-H), 2.65 (dd, 1H, *J* = 10 and 14 Hz, *CH_2_*-Ph), 2.74 (m, 1H, 2-H), 2.78 (m, 1H, δ-H), 2.93 (m, 1H, *CH_2_*-Ph), 3.35–3.82 (m, 6H, 3-H, 6-H and *CH_2_*CO), 4.03 [d, 1H, *J* = 15 Hz, CH_2_ (NH*Bn*, Urea)], 4.15 [d, 1H, *J* = 15 Hz, CH_2_ (NH*Bn*, Urea)], 4.25 [m, 1H, CH_2_ (NH*Bn*)], 4.30 [m, 1H, CH_2_ (NH*Bn*)], 4.36 (m, 1H, 2-*CH*), 4.38 (m, 1H, α-H), 4.47 [d, 1H, *J* = 15 Hz, CH_2_ (N*Bn*)], 4.62 [d, 1H, *J* = 15 Hz, CH_2_ (N*Bn*)], 6.49 (m, 1H, 2-CH*NH*), 6.90–7.40 [m, 21H, Ar and *NH*Bn (Urea)], 7.92 (m, 3H, NH_2_·HCl), 8.56 (m, 1H, α-NH), 8.72 (t, 1H, *J* = 6 Hz, *NH*Bn). (***S***)-**20a** *δ* (ppm): 1.58 (m, 1H, β-H), 1.72 (m, 1H, β-H), 2.50 (m, 1H, *CH_2_*-Ph), 2.78 (m, 1H, δ-H), 2.79 (m, 1H, *CH_2_*-Ph), 3.97 [d, 1H, *J* = 15 Hz, CH_2_ (NH*Bn*, Urea) and 5-*CH*], 4.14 [m, 1H, CH_2_ (NH*Bn*, Urea)], 4.25 [m, 1H, CH_2_ (NH*Bn*)], 4.30 [m, 1H, CH_2_ (NH*Bn*)], 4.44 [m, 1H, CH_2_ (N*Bn*)], 4.58 [m, 1H, CH_2_ (N*Bn*)], 6.42 (m, 1H, 2-CH*NH*), 6.90–7.40 [m, 21H, Ar and *NH*Bn (Urea)], 7.92 (m, 3H, NH_2_·HCl), 8.74 (t, 1H, *J* = 6 Hz, *NH*Bn); ^13^C-NMR (125 MHz, DMSO-*d_6_*) (***R***)-**20a** *δ* (ppm): 23.4 [C_γ_], 29.2 [C_β_], 37.8 [*CH_2_*-Ph], 38.1 [C_δ_], 42.0 [CH_2_ (NH*Bn*)], 42.7 [CH_2_ (NH*Bn*, Urea)], 43.7 [C_3_], 49.1 [CH_2_ (N*Bn*)], 51.9 [C_α_], 49.7 [C_2_-*CH*], 53.7 [C_6_], 53.9 [*CH_2_*CO], 60.8 [C_2_], 126.4, 126.6, 126.7, 127.1, 127.6, 128.1, 128.3, 128.5, 129.2 [20CH (Ar)], 136.4 [C (N*Bn*)], 137.1 [C (Ph)], 139.0 [C (NH*Bn*)], 140.4 [C (NH*Bn*, Urea)], 158.1 [CO (Urea)], 170.9 [α-CONH]. (***S***)-**20a** *δ* (ppm): 29.4 [C_β_], 37.8 [*CH_2_*-Ph], 38.2 [C_δ_], 42.0 [CH_2_ (NH*Bn*)], 42.7 [CH_2_ (NH*Bn*, Urea)], 49.1 [CH_2_ (N*Bn*)], 49.7 [C_2_-*CH*], 126.4, 126.5, 126.7, 127.3, 127.8, 128.0, 128.3, 128.6, 129.2 [20CH (Ar)], 136.4 [C (N*Bn*)], 137.1 [C (Ph)], 139.0 [C (NH*Bn*)], 140.4 [C (NH*Bn*, Urea)], 157.8 [CO (Urea)], 170.9 [α-CONH]; ES-MS *m/z* [M+2]^+^ calculated for C_41_H_49_N_7_O_4_: 705.3; found: 705.6.

N*-[2-[4-Benzyl-(2RS)-[(1S)-(3-benzylureido)-2-phenylethyl]-5-oxopiperazin-1-yl]acetyl]-Lys-NHBn hydrochloride* (**20b**). Amorphous solid [(*R*:*S)* = (3:1)] (151 mg, 100%); HPLC *t*_R_: 15.83 min [(***R***)-**20b**] and 16.25 min [(***S***)-**20b**]; ^1^H-NMR (500 MHz, DMSO-*d_6_*) (***R***)-**20b** *δ* (ppm): 1.29 (m, 2H, γ-H), 1.52 (m, 2H, δ-H), 1.54 (m, 1H, β-H), 1.72 (m, 1H, β-H), 2.67 (m, 1H, 2-H), 2.68 (m, 2H, ε-H), 2.69 (m, 1H, *CH_2_*-Ph), 2.87 (m, 1H, *CH_2_*-Ph), 3.34–3.87 (m, 6H, 3-H, 6-H and *CH_2_*CO), 4.03 [d, 1H, *J* = 15 Hz, CH_2_ (NH*Bn*, Urea)], 4.14 [d, 1H, *J* = 15 Hz, CH_2_ (NH*Bn*, Urea)], 4.20 [m, 1H, CH_2_ (NH*Bn*)], 4.29 [m, 1H, CH_2_ (NH*Bn*)], 4.30 (m, 1H, α-H), 4.33 (m, 1H, 2-*CH*), 4.50 [m, 1H, CH_2_ (N*Bn*], 4.64 [m, 1H, CH_2_ (N*Bn*], 6.48 (m, 1H, 2-CH*NH*), 6.94–7.41 [m, 21H, Ar and *NH*Bn (Urea)], 7.87 (m, 3H, NH_2_·HCl), 8.50 (m, 1H, α-NH), 8.60 (m, 1H, *NH*Bn). (***S***)-**20b** *δ* (ppm): 1.25 (m, 1H, γ-H), 1.35 (m, 1H, γ-H), 1.54 (m, 1H, β-H), 1.72 (m, 1H, β-H), 2.52 (m, 1H, *CH_2_*-Ph), 2.80 (m, 1H, *CH_2_*-Ph), 3.98 [d, 1H, *J* = 15 Hz, CH_2_ (NH*Bn*, Urea)], 4.14 [m, 1H, CH_2_ (NH*Bn*, Urea)], 4.20 [m, 1H, CH_2_ (NH*Bn*)], 4.29 [m, 1H, CH_2_ (NH*Bn*)], 6.40 (m, 1H, 2-CH*NH*), 6.94–7.41 [m, 21H, Ar and *NH*Bn (Urea)], 7.87 (m, 3H, NH_2_·HCl), 8.62 (m, 1H, *NH*Bn); ^13^C-NMR (125 MHz, DMSO-*d_6_*) (***R***)-**20b** *δ* (ppm): 22.7 [C_γ_], 26.9 [C_δ_], 31.7 [C_β_], 38.2 [*CH_2_*-Ph], 38.9 [C_ε_], 42.5 [CH_2_ (NH*Bn*)], 43.0 [CH_2_ (NH*Bn*, Urea)], 44.2 [C_3_], 49.5 [C_2_-*CH*], 49.7 [CH_2_ (N*Bn*)], 53.0 [C_α_], 54.0 [C_6_], 60.1 [C_2_], 126.7, 126.8, 127.0, 127.2, 127.5, 128.0, 128.5, 128.6, 129.0, 129.6 [20CH (Ar)], 136.9 [C (N*Bn*)], 138.3 [C (Ph)], 139.7 [C (NH*Bn*)], 140.8 [C (NH*Bn*, Urea)], 158.6 [CO (Urea)], 171.5 [α-CONH]. (***S***)-**20b** *δ* (ppm): 22.7 [C_γ_], 31.5 [C_β_], 38.0 [*CH_2_*-Ph], 42.5 [CH_2_ (NH*Bn*)], 43.0 [CH_2_ (NH*Bn*, Urea)], 126.7, 126.8, 127.0, 127.2, 127.7, 128.2, 128.5, 128.6, 129.0, 129.6 [20CH (Ar)], 136.9 [C (N*Bn*)], 138.3 [C (Ph)], 139.7 [C (NH*Bn*)], 141.0 [C (NH*Bn*, Urea)], 158.2 [CO (Urea)], 171.5 [α-CONH]; ES-MS *m/z* [M+2]^+^ calculated for C_42_H_51_N_7_O_4_: 719.4; found: 719.9.

### 3.13. General Procedure for Removal of the N-Pbf Protecting Group. Synthesis of N-[2-[4-benzyl-5-oxo-(2RS)-[2-phenyl-(1S)-(3-phenyl-ureido)ethyl]piperazin-1-yl]acetyl]-Arg-NHBn Trifluoroacetate (**19c**)

The epimeric mixture of the Arg(Pbf) -derived phehylureido-piperazine **17c** [(*R*:*S*) = (3:1)] (295 mg, 0.30 mmol) was dissolved in TFA/H_2_O/TIS mixture (90:5:5; 5 mL) and the mixture was stirred at room temperature for 5 h. Afterwards, the TFA was evaporated under stream of argon and the residue was centrifuged three times in diethyl ether (10 mL) at 5000 rpm and −15 °C for 15 min. The residue was dissolved in CH_3_CN/H_2_O (1:3, 2 mL) and the solution was lyophilized. The epimeric mixture of trifluoroacetate salts **19c** [(*R*:*S)* = (3:1)] was obtained quantitatively (254 mg, 100%). HPLC *t*_R_: 16.60 min [(***R***)-**19c**] and 16.87 min [(***S***)-**19c**]; ^1^H-NMR (500 MHz, DMSO-*d_6_*) (***R***)-**19c** *δ* (ppm): 1.44 (m, 2H, γ-H), 1.63 (m, 1H, β-H), 1.72 (m, 1H, β-H), 2.71 (dd, 1H, *J* = 9 and 13.5 Hz, *CH_2_*-Ph), 2.95 (m, 1H, 2-H), 2.96 (m, 1H, *CH_2_*-Ph), 3.03 (m, 2H, δ-H), 3.20 (m, 1H, 3-H), 3.28 (m, 1H, 6-H), 3.30 (m, 1H, 3-H), 3.31 (m, 2H, *CH_2_*CO), 3.48 (d, 1H, *J* = 17 Hz, 6-H), 4.10 (m, 1H, 2-*CH*), 4.23 [m, 2H, CH_2_ (NH*Bn*)], 4.30 [m, 1H, CH_2_ (N*Bn*)], 4.32 (m, 1H, α-H), 4.72 [d, 1H, *J* = 15 Hz, CH_2_ (N*Bn*)], 6.49 (m, 1H, 2-CH*NH*), 6.73–7.36 (m, 20H, Ar), 7.76 [m, 1H, NHC(NH_2_·CF_3_CO_2_H) = NH], 8.23 (d, 1H, *J* = 7 Hz, α-NH), 8.57 (t, 1H, *J* = 6 Hz, *NH*Bn), 9.00 (m, 1H, *NH*Ph). (***S***)-**19c** *δ* (ppm): 2.97 (m, 1H, δ-H), 3.07 (m, 1H, δ-H), 3.12 (m, 1H, *CH_2_*CO), 3.14 (m, 1H, 3-H), 3.23 (m, 1H, 6-H), 3.30 (m, 1H, 3-H), 3.42 (m, 1H, *CH_2_*CO), 3.77 (d, 1H, *J* = 16.5 Hz, 6-H), 3.91 (m, 1H, 2-*CH*), 4.04 [m, 1H, CH_2_ (NH*Bn*)], 4.30 [m, 1H, CH_2_ (NH*Bn*)], 4.43 [d, 1H, *J* = 15 Hz, CH_2_ (N*Bn*)], 4.54 [d, 1H, *J* = 15 Hz, CH_2_ (N*Bn*)], 6.73–7.36 (m, 20H, Ar), 7.76 [m, 1H, NHC(NH_2_·CF_3_CO_2_H) = NH], 8.59 (m, 1H, *NH*Bn), 9.00 (m, 1H, *NH*Ph); ^13^C-NMR (125 MHz, DMSO-*d_6_*) (***R***)-**19c** *δ* (ppm): 25.4 [C_γ_], 29.7 [C_β_], 37.6 [*CH_2_*-Ph], 40.8 [C_δ_], 42.5 [CH_2_ (NH*Bn*)], 45.3 [C_3_], 49.5 [CH_2_ (N*Bn*)], 50.8 [C_2_-*CH*], 52.6 [C_α_], 54.9 [C_6_], 55.4 [*CH_2_*CO], 59.9 [C_2_], 118.2, 121.5, 126.4, 127.2, 217.3, 127.5, 128.1, 128.6, 128.7, 129.0, 129.7 [20CH (Ar)], 137.6 [C (N*Bn*)], 139.2 [C (Ph)], 139.7 [C (NH*Bn*)], 140.9 [C (NH*Ph*)], 157.1 [CO (Urea)], 155.4 [C (NHC(NH_2_) = N)], 167.1 [C_5_], 170.0 [CO], 171.8 [α-CONH]. (***S***)-**19c** *δ* (ppm): 40.8 [C_δ_], 42.3 [CH_2_ (NH*Bn*)], 46.1 [C_3_], 48.9 [CH_2_ (N*Bn*)], 51.6 [C_2_-*CH*], 54.9 [C_6_], 55.4 [*CH_2_*CO], 118.0, 121.5, 126.4, 127.2, 217.3, 127.6, 128.2, 128.4, 128.6, 129.0, 129.7 [20CH (Ar)], 138.0 [C (N*Bn*)], 139.2 [C (Ph)], 139.7 [C (NH*Bn*)], 140.9 [C (NH*Ph*)], 157.5 [CO (Urea)], 155.8 [C (NHC(NH_2_) = N)], 170.6 [CO], 173.0 [α-CONH]; ES-MS *m/z* [M+1]^+^ calculated for C_41_H_49_N_9_O_4_: 732.4; found: 732.7.

### 3.14. General Procedure for the Synthesis of the Indazole-Derived Ureas **23b**,**c**.

Propylene oxide (19 μL, 0.27 mmol) was added to a 0 °C cooled solution of 1-(2,6-dichlorobenzyl)-6-amino-3-(pyrrolidin-1-ylmethyl)-1*H*-indazol [24] (83 mg, 0.22 mmol) in dry THF (4 mL). Then, a solution of bis(trichloromethyl)carbonate (24 mg, 0.082 mmol) in dry THF (1 mL) was added dropwise and stirring was maintened at 0 °C for 15 min. Afterwards, the mixture was added dropwise to a 0 °C cooled solution of the corresponding epimeric mixture of hydrochlorides **16b**,**c** (0.22 mmol) and Et_3_N (17 μL, 0.48 mmol) in dry THF (5 mL) and stirred for 2h. Then, the solvent was removed under reduced pressure and the residue was dissolved in CH_2_Cl_2_ (50 mL). The solution was washed with H_2_O (2 × 10 mL), brine (10 mL), dried over Na_2_SO_4_, and evaporated to dryness. The residue was purified by reverse phase chromatography, using 10%–100% CH_3_CN gradient in 0.05% TFA solution in H_2_O as mobile phase, to afford the desired compounds **23b**,**c.**

N*-[2-[4-Benzyl-(2*RS*)-[(1*S*)-[3-(1-(2,6-dichlorobenzyl)-3-(pyrrolidin-1-ylmethyl)-1H-indazol-6-yl)ureido]-2-phenylethyl]-5-oxopiperazin-1-yl]acetyl]-Lys(Z)-NHBn* (**23b**). Amorphous solid [(*R*:*S)* = (3:1)] (74 mg, 30%); HPLC *t*_R_: 19.99 min; ^1^H-NMR (500 MHz, CDCl_3_) (***R***)-**23b** *δ* (ppm): 1.26 (m, 2H, γ-H), 1.33 (m, 2H, δ-H), 1.70 (m, 1H, β-H), 1.72 (m, 2H, pyrrolidine), 1.82 (m, 1H, β-H), 1.88 (m, 2H, pyrrolidine), 2.68 (dd, 1H, *J* = 6.5 and 14 Hz, *CH_2_*-Ph), 2.90 (m, 1H, *J* = 8 and 14 Hz, *CH_2_*-Ph), 2.96 (m, 1H, 2-H), 2.98 (m, 2H, ε-H), 3.00 (m, 2H, pyrrolidine), 3.27 (m, 1H, 6-H), 3.42 (m, 1H, 3-H), 3.45 (m, 4H, pyrrolidine and *CH_2_*CO), 3.47 (m, 1H, 3-H), 3.53 (d, 1H, *J* = 16 Hz, 6-H), 4.18 [m, 1H, *J* = 14.5 Hz, CH_2_ (N*Bn*)], 4.28 [m, 1H, CH_2_ (NH*Bn*), 4.30 (m, 1H, 2-*CH*), 4.32 [m, 1H, CH_2_ (NH*Bn*), 4.35 (s, 2H, CH_2_-pyrrolidine), 4.40 (m, 1H, α-H), 4.95 [m, 1H, CH_2_ (N*Bn*], 4.98 [s, 2H, CH_2_ (Z)], 5.36 (m, 1H, *NH*Z), 5.50 (s, 2H, CH_2_-diClPh), 6.83 (d, 2H, *J* = 8 Hz, Ar), 7.02 (d, 2H, *J* = 7 Hz, Ar), 7.12–7.35 (m, 22H, Ar and *NH*Bn), 7.91 (s, 1H, Ar), 8.06 (m, 1H, α-NH), 8.58 (m, 1H, 2-CH*NH*), 11.67 [m, 1H, Indz-*NH* (Urea)]. (***S***)-**23b** *δ* (ppm): 1.35 (m, 2H, δ-H), 1.72 (m, 2H, pyrrolidine), 1.88 (m, 2H, pyrrolidine), 3.00 (m, 2H, pyrrolidine), 3.24 (m, 1H, 3-H), 3.43 (m, 2H, pyrrolidine), 3.45 (m, 1H, 3-H), 4.35 (s, 2H, CH_2_-Pyrrolidine), 4.38 (m, 1H, α-H), 4.94 [s, 2H, CH_2_ (Z)], 5.36 (m, 1H, *NH*Z), 5.50 (s, 2H, CH_2_-diClPh), 6.76 (m, 2H, Ar), 7.02 (m, 2H, Ar), 7.12–7.35 (m, 22H, Ar and *NH*Bn), 7.91 (s, 1H, Ar), 8.10 (m, 1H, α-NH), 8.70 (m, 1H, 2-CH*NH*), 11.67 [m, 1H, Indz-*NH* (Urea)]; ^13^C-NMR (125 MHz, CDCl_3_) (***R***)-**23b** *δ* (ppm): 22.8 [C_γ_], 23.4 [2CH_2_ (pyrrolidine)], 29.1 [C_δ_], 31.5 [C_β_], 37.7 [*CH_2_*-Ph], 40.3 [C_ε_], 43.5 [CH_2_ (NH*Bn*)], 44.2 [C_3_], 47.6 [*CH_2_*-diClPh], 48.2 [*CH_2_*-pyrrolidine], 49.5 [CH_2_ (N*Bn*)], 51.6 [C_2_-*CH*], 52.4 [2CH_2_ (pyrrolidine)], 53.4 [C_α_], 55.2 [C_6_], 57.5 [*CH_2_*CO], 60.7 [C_2_], 66.6 [CH_2_ (Z)], 98.0, 116.0, 118.6 [3CH (Ar)], 119.2 [C (Ar)], 126.8, 127.3, 127.9, 128.0, 128.2, 128.4, 128.5, 128.6, 128.7, 128.9, 130.1 [23CH (Ar)], 131.3, 133.7 [2C (Ar)], 135.9 [C (N*Bn*)], 136.5 [C (Z)], 136.8 [3C (Ph and Ar)], 137.7 [C (NH*Bn*)], 139.3, 137.7 [2C (Ar)], 155.8 [CO (Z)], 156.8 [CO (Urea)], 166.9 [C_5_], 169.6 [CO], 172.5 [α-CONH]. (***S***)-**23b** *δ* (ppm): 23.4 [2CH_2_ (pyrrolidine)], 29.1 [C_δ_], 44.2 [C_3_], 47.6 [*CH_2_*-diClPh], 48.2 [*CH_2_*-pyrrolidine], 52.4 [2CH_2_ (pyrrolidine)], 53.4 [C_α_], 66.6 [CH_2_ (Z)], 98.0, 115.2, 117.5 [3CH (Ar)], 119.2 [C (Ar)], 126.8, 127.4, 127.9, 128.0, 128.2, 128.4, 128.5, 128.6, 128.7, 128.9, 130.1 [23CH (Ar)], 131.3, 133.7 [2C (Ar)], 135.9 [C (N*Bn*)], 136.5 [C (Z)], 136.8 [3C (Ph and Ar)], 137.7 [C (NH*Bn*)], 139.3, 137.7 [2C (Ar)], 155.8 [CO (Z)], 156.8 [CO (urea)], 172.5 [α-CONH]; ES-MS *m/z* 1120.9 [M+1]^+^; C_62_H_68_Cl_2_N_10_O_6_ (%): C: 66.48, H: 6.12, N: 12.50. Found (%): C: 66.79, H: 6.38, N: 12.34.

N*-[2-[4-Benzyl-(2*RS*)-[(1*S*)-[3-(1-(2,6-dichlorobenzyl)-3-(pyrrolidin-1-ylmethyl)-1H-indazol-6-yl)ureido]-2-phenylethyl]-5-oxopiperazin-1-yl]acetyl]-Arg(Pbf)-NHBn* (**23c**). Amorphous solid [(*R*:*S)* = (3:1)] (106 mg, 38%); HPLC *t*_R_: 20.38 min [(***R***)-**23c**] and 21.40 min [(***S***)-**23c**]; ^1^H-NMR (500 MHz, (CD_3_)_2_CO) (***R***)-**23c ***δ* (ppm): 1.30 [s, 6H, 2CH_3_ (Pbf)], 1.40 (m, 2H, γ-H), 1.60 (m, 1H, β-H), 1.73 (m, 1H, β-H), 1.86 [s, 3H, CH_3_ (Pbf)], 1.88 (m, 2H, pyrrolidine), 1.97 (m, 2H, pyrrolidine), 2.36 [s, 3H, CH_3_ (Pbf)], 2.44 [s, 3H, CH_3_ (Pbf)], 2.72 (m, 2H, *CH_2_*-Ph), 2.86 [s, 2H, CH_2_ (Pbf)], 3.00 (m, 1H, δ-H), 3.08 (m, 1H, 2-H), 3.10 (m, 1H, δ-H), 3.26 (m, 1H, *CH_2_*CO), 3.32 (m, 1H, 6-H), 3.34 (m, 2H, pyrrolidine), 3.52 (m, 1H, *CH_2_*CO), 3.57 (m, 1H, 6-H), 3.58 (m, 1H, 3-H), 3.62 (m, 1H, 3-H), 3.68 (m, 2H, pyrrolidine), 4.20 [m, 1H, CH_2_ (N*Bn*)], 4.34 [m, 1H, CH_2_ (NH*Bn*)], 4.38 [m, 2H, 2-*CH* and CH_2_ (NH*Bn*)], 4.39 (m, 1H, α-H), 4.66 (s, 2H, CH_2_-pyrrolidine), 4.88 [d, 1H, *J* = 15 Hz, CH_2_ (N*Bn*)], 5.55 (d, 2H, *J* = 6 Hz, CH_2_-diClPh), 6.39 [m, 3H, NHC(NH_2_) = N], 6.95–7.34 (m, 21H, Ar), 7.86 (m, 1H, *NH*Bn), 8.26 (d, 1H, *J* = 6 Hz, α-NH), 9.26 (m, 1H, 2-CH*NH*), 10.32 [m, 1H, Indz-*NH* (Urea)]. (***S***)-**23c** *δ* (ppm): 1.30 [s, 6H, 2CH_3_ (Pbf)], 1.86 [s, 3H, CH_3_ (Pbf)], 1.88 (m, 2H, pyrrolidine), 1.97 (m, 2H, pyrrolidine), 2.36 [s, 3H, CH_3_ (Pbf)], 2.44 [s, 3H, CH_3_ (Pbf)], 2.68 (m, 2H, *CH_2_*-Ph), 2.80 (m, 2H, *CH_2_*-Ph), 2.86 [s, 2H, CH_2_ (Pbf)], 3.34 (m, 2H, pyrrolidine), 3.68 (m, 2H, pyrrolidine), 4.10 [dd, 1H, *J* = 6 and 15 Hz, CH_2_ (NH*Bn*)], 4.37 (m, 1H, α-H), 4.40 [m, 1H, CH_2_ (NH*Bn*)], 4.43 [d, 1H, *J* = 14 Hz, CH_2_ (N*Bn*)], 4.58 [m, 1H, CH_2_ (N*Bn*)], 4.66 (m, 2H, CH_2_-pyrrolidine), 5.55 (m, 2H, *J* = 6 Hz, CH_2_-diClPh), 6.50 [m, 3H, NHC(NH_2_) = N], 6.95–7.34 (m, 21H, Ar), 7.91 (m, 1H, *NH*Bn), 8.26 (m, 1H, α-NH), 9.26 (m, 1H, 2-CH*NH*), 10.32 [m, 1H, Indz-*NH* (urea)]; ^13^C-NMR (125 MHz, (CD_3_)_2_CO) (***R***)-**23c** *δ* (ppm): 13.2, 19.0, 20.2 [3CH_3_ (Pbf)], 24.5 [2CH_2_ (pyrrolidine)], 27.5 [C_γ_], 28.6 [2CH3 (Pbf)], 31.3 [C_β_], 39.2 [*CH_2_*-Ph], 41.4 [C_δ_], 44.0 [CH_2_ (NH*Bn*)], 44.3 [CH_2_ (Pbf)], 45.7 [C_3_], 49.0 [*CH_2_*-diClPh], 50.5 [CH_2_ (N*Bn*)], 50.7 [*CH_2_*-pyrrolidine], 54.1 [C_2_-*CH* and C_α_], 55.1 [2CH_2_ (pyrrolidine)], 56.0 [C_6_], 59.5 [*CH_2_*CO], 63.2 [C_2_], 87.5 [C (Pbf)], 98.9, 117.2 [2CH (Ar)], 118.4 [C (Pbf)], 118.6 [CH (Ar)], 119.7 [C (Ar)], 121.4 [CH (Ar)], 126.2 [C (Pbf)], 127.7, 128.2, 128.7, 129.6, 129.8, 130.1, 130.2, 130.8, 132.1 [17CH (Ar)], 133.5, 135.7 [2C (Pbf)], 136.7, 138.3 [3C (Ar)], 139.0 [C (N*Bn*)], 139.5 [C (Pbf)], 140.3 [C (Ph)], 140.9 [C (NH*Bn*)], 141.9, 143.5 [3C (Ar)], 157.6 [CO (Urea)], 160.9 [C (NHC(NH_2_) = N)], 159.7 [C (Pbf)], 168.3 [C_5_], 171.1 [CO], 173.2 [α-CONH]. (***S***)-**23c** *δ* (ppm): 13.2, 19.0, 20.2 [3CH_3_ (Pbf)], 24.5 [2CH_2_ (pyrrolidine)], 28.6 [2CH_3_ (Pbf)], 39.2 [*CH_2_*-Ph], 44.0 [CH_2_ (NH*Bn*)], 44.3 [CH_2_ (Pbf)], 49.0 [*CH_2_*-diClPh], 50.4 [CH_2_ (N*Bn*)], 50.7 [*CH_2_*-pyrrolidine], 54.1 [C_α_], 55.1 [2CH2 (pyrrolidine)], 87.5 [C (Pbf)], 98.9, 117.2 [2CH (Ar)], 118.4 [C (Pbf)], 118.6 [CH (Ar)], 119.1 [C (Ar)], 121.4 [CH (Ar)], 126.2 [C (Pbf)], 127.7, 128.2, 128.8, 129.6, 129.8, 130.1, 130.2, 131.0, 132.1 [17CH (Ar)], 133.5, 135.7 [2C (Pbf)], 136.7, 138.3 [3C (Ar)], 139.1 [C (N*Bn*)], 139.5 [C (Pbf)], 140.3 [C (Ph)], 140.9 [C (NH*Bn*)], 141.9, 143.5 [3C (Ar)], 158.1 [CO (Urea)], 160.9 [C (NHC(NH_2_) = N)], 159.7 [C (Pbf)]; ES-MS *m/z* 1267.7 [M+1]^+^; C_67_H_78_Cl_2_N_12_O_7_S (%): C: 63.54, H: 6.21, N: 13.27. Found (%): C: 63.78, H: 6.02, N: 13.39.

### 3.15. N-Z Removal in **23b**. Synthesis of N-[2-[4-Benzyl-(2RS)-[(1S)-[3-(1-(2,6-dichlorobenzyl)-3-(pyrrolidin-1-ylmethyl)-1H-indazol-6-yl)ureido]-2-phenylethyl]-5-oxopiperazin-1-yl]acetyl]-Lys-NHBn hydrochloride (**24b**)

It was carried out by applying the general methodology for N-Z removal above described for the synthesis of (**11**–**12**)**a**,**b**. Amorphous solid [(*R*:*S)* = (3:1)] (70 mg, 100%); HPLC *t*_R_: 14.99 min; ^1^H-NMR (500 MHz, DMSO-*d_6_*) (***R***)-**24b** *δ* (ppm): 1.30 (m, 2H, γ-H), 1.50 (m, 2H, δ-H), 1.62 (m, 1H, β-H), 1.72 (m, 1H, β-H), 1.79 (m, 2H, pyrrolidine), 1.85 (m, 2H, pyrrolidine), 2.74 (dd, 1H, *J* = 10 and 14 Hz, *CH_2_*-Ph), 2.96 (m, 1H, *CH_2_*-Ph), 2.65 (m, 3H, 5-H and ε-H), 3.05 (m, 2H, pyrrolidine), 3.35 (m, 2H, pyrrolidine), 3.44–3.98 (m, 6H, 3-H, 6-H and *CH_2_*CO), 4.25 [d, 1H, *J* = 6 Hz, CH_2_ (NH*Bn*), 4.27 [d, 1H, *J* = 6 Hz, CH_2_ (NH*Bn*), 4.33 (m, 2H, 2-*CH* and α-H), 4.46 [m, 1H, CH_2_ (N*Bn*], 4.57 (d, 2H, *J* = 5 Hz, CH_2_-pyrrolidine), 4.72 [d, 1H, *J* = 15 Hz, CH_2_ (N*Bn*], 5.59 (s, 2H, CH_2_-diClPh), 7.01 (d, 1H, *J* = 8 Hz, Ar), 6.96–7.46 (m, 16H, Ar), 7.52 (d, 2H, *J* = 8 Hz, Ar), 7.83 (d, 1H, *J* = 9 Hz, Ar), 7.86 (s, 1H, Ar), 7.95 (m, 3H, NH_2_·HCl), 8.45 (m, 1H, α-NH), 8.63 (t, 1H, *J* = 6 Hz, *NH*Bn), 9.35 (s, 1H, 2-CH*NH*), 11.68 [m, 2H, Indz-*NH* (urea) and N·HCl (pyrrolidine)]. (***S***)-**24b** *δ* (ppm): 1.79 (m, 2H, pyrrolidine), 1.85 (m, 2H, pyrrolidine), 2.74 (m, 1H, *CH_2_*-Ph), 2.96 (m, 1H, *CH_2_*-Ph), 3.05 (m, 2H, pyrrolidine), 3.35 (m, 2H, pyrrolidine), 4.22 [m, 1H, CH_2_ (NH*Bn*), 4.28 [m, 1H, CH_2_ (NH*Bn*), 4.57 (d, 2H, *J* = 5 Hz, CH_2_-pyrrolidine), 4.72 [d, 1H, *J* = 15 Hz, CH_2_ (N*Bn*], 5.59 (s, 2H, CH_2_-diClPh), 7.01 (d, 1H, *J* = 8 Hz, Ar), 6.96–7.46 (m, 16H, Ar), 7.52 (d, 2H, *J* = 8 Hz, Ar), 7.83 (d, 1H, *J* = 9 Hz, Ar), 7.86 (s, 1H, Ar), 7.95 (m, 3H, NH_2_·HCl), 8.45 (m, 1H, α-NH), 8.63 (m, 1H, *NH*Bn), 9.35 (s, 1H, 2-CH*NH*), 11.68 [m, 2H, Indz-*NH* (Urea) and N·HCl (pyrrolidine)]; ^13^C-NMR (125 MHz, DMSO-*d_6_*) (***R***)-**24b** *δ* (ppm): 22.3 [C_γ_], 22.6 [2CH2 (pyrrolidine)], 26.4 [C_δ_], 31.4 [C_β_], 37.9 [*CH_2_*-Ph], 38.4 [C_ε_], 42.0 [CH_2_ (NH*Bn*)], 44.1 [C_3_], 47.2 [*CH_2_*-diClPh], 47.7 [*CH_2_*-pyrrolidine], 49.2 [CH_2_ (N*Bn*)], 49.6 [C_2_-*CH*], 52.5 [2CH2 (pyrrolidine), Cα and CH2CO], 53.9 [C_6_], 60.3 [C_2_], 96.1, 114.5 [2CH (Ar)], 117.7 [C (Ar)], 120.6, 126.7, 127.0, 127.2, 127.6, 128.2, 128.5, 128.7, 129.2 [19CH (Ar)], 130.8, 131.4 [4C (Ar)], 135.4 [C (N*Bn*)], 136.0 [C (Ph)], 139.2 [C (NH*Bn*)], 141.2 [2C (Ar)], 155.2 [CO (urea)], 171.1 [α-CONH]. (***S***)-**24b** *δ* (ppm): 22.6 [2CH2 (pyrrolidine)], 37.8 [*CH_2_*-Ph], 42.0 [CH_2_ (NH*Bn*)], 47.2 [*CH_2_*-diClPh], 47.7 [*CH_2_*-pyrrolidine], 52.5 [2CH2 (pyrrolidine)], 96.1, 114.5 [2CH (Ar)], 117.7 [C (Ar)], 120.6, 126.7, 127.0, 127.2, 127.6, 128.1, 128.5, 128.7, 129.2 [19CH (Ar)], 130.8, 131.4 [4C (Ar)], 135.4 [C (N*Bn*)], 136.0 [C (Ph)], 139.6 [C (NH*Bn*)], 141.2 [2C (Ar)], 155.2 [CO (urea)], 171.1 [α-CONH]; ES-MS *m/z* [(M+2)/2]^+^ calculated for C_54_H_62_Cl_2_N_10_O_4_: 493.2; found: 493.6.

### 3.16. N-Pbf Removal in **23c**. Synthesis of N-[2-[4-Benzyl-(2RS)-[(1S)-[3-(1-(2,6-dichlorobenzyl)-3-(pyrrolidin-1-ylmethyl)-1H-indazol-6-yl)ureido]-2-phenylethyl]-5-oxopiperazin-1-yl]acetyl]-Arg-NHBn Trifluoroacetate (**24c**)

It was carried out by applying the above described methodology for *N*-Pbf removal in the Arg derivative **19c**. Amorphous solid [(*R*:*S)* = (3:1)] (104 mg, 100%); HPLC *t*_R_: 15.14 min [(***R***)-**24c**] and 15.72 min [(***S***)-**24c**]; ^1^H-NMR (500 MHz, (CD_3_)_2_CO) (***R***)-**24c** *δ* (ppm): 1.44 (m, 2H, γ-H), 1.60 (m, 1H, β-H), 1.84 (m, 1H, β-H), 1.83 (m, 4H, pyrrolidine), 2.75 (dd, 1H, *J* = 9.5 and 14 Hz, *CH_2_*-Ph), 3.00 (m, 1H, 2-H), 3.04 (m, 2H, δ-H), 3.06 (m, 1H, *CH_2_*-Ph), 3.20 (m, 1H, *CH_2_*CO), 3.26 (m, 1H, 3-H), 3.32 (m, 1H, 6-H), 3.35 (m, 4H, pyrrolidine), 3.39 (m, 1H, 3-H), 3.42 (m, 1H, *CH_2_*CO), 3.47 (m, 1H, 6-H), 4.21 (m, 1H, 2-*CH*), 4.27 [d, 2H, *J* = 6 Hz, CH_2_ (NH*Bn*)], 4.33 [d, 1H, *J* = 15 Hz, CH_2_ (N*Bn*)], 4.40 (td, 1H, *J* = 5.5 and 8 Hz, α-H), 4.58 (s, 2H, CH_2_-pyrrolidine), 4.74 [d, 1H, *J* = 15 Hz, CH_2_ (N*Bn*)], 5.60 (s, 2H, CH_2_-diClPh), 6.37 (m, 1H, 2-CH*NH*), 6.90–7.44 (m, 18H, Ar), 7.52 [m, 2H, NHC(NH_2_·CF_3_CO_2_H) = NH and Ar], 7.72 (d, 1H, *J* = 9 Hz, Ar), 7.92 (s, 1H, Ar), 8.09 (d, 1H, *J* = 8 Hz, α-NH), 8.58 (t, 1H, *J* = 6 Hz, *NH*Bn), 8.77 [m, 1H, Indz-*NH* (Urea)], 9.95 [m, 1H, N·CF_3_CO_2_H (Pyrrolidine)]. (***S***)-**24c** *δ* (ppm): 1.83 (m, 4H, pyrrolidine), 2.63 (m, 1H, *CH_2_*-Ph), 2.82 (m, 1H, *CH_2_*-Ph), 3.30 (m, 1H, 6-H), 3.35 (m, 4H, pyrrolidine), 3.50 (m, 1H, 6-H), 4.04 (m, 1H, 2-*CH*), 4.14 [m, 1H, CH_2_ (NH*Bn*)], 4.28 [m, 1H, CH_2_ (NH*Bn*)], 4.58 (s, 2H, CH_2_-pyrrolidine), 5.57 (s, 2H, CH_2_-diClPh), 6.90–7.44 (m, 18H, Ar), 7.52 [m, 2H, NHC(NH_2_·CF_3_CO_2_H) = NH and Ar], 7.70 (d, 1H, *J* = 9 Hz, Ar), 7.95 (s, 1H, Ar), 8.57 (m, 1H, *NH*Bn), 9.95 [m, 1H, N·CF_3_CO_2_H (pyrrolidine)]; ^13^C-NMR (125 MHz, (CD_3_)_2_CO) (***R***)-**24c** *δ* (ppm): 23.0 [2CH_2_ (pyrrolidine)], 25.5 [C_γ_], 30.0 [C_β_], 37.8 [*CH_2_*-Ph], 40.4 [C_δ_], 42.5 [CH_2_ (NH*Bn*)], 44.9 [C_3_], 47.7 [*CH_2_*-diClPh], 48.6 [*CH_2_*-pyrrolidine], 49.6 [CH_2_ (N*Bn*)], 50.5 [C_2_-*CH*], 52.2 [C_α_], 53.5 [2CH_2_ (pyrrolidine)], 54.4 [C_6_], 55.2 [*CH_2_*CO], 59.7 [C_2_], 96.8, 115.1 [2CH (Ar)], 118.0 [C (Ar)], 120.7 [CH (Ar)], 126.5, 127.2, 127.5, 128.0, 128.5, 128.7, 129.0, 129.2, 129.7 [18CH (Ar)], 131.4, 131.9, 136.5 [4C (Ar)], 137.4 [C (N*Bn*)], 139.0 [C (Ph)], 139.6 [C (NH*Bn*)], 140.2, 141.9 [2C (Ar)], 157.0 [CO (urea)], 155.3 [C (NHC(NH_2_) = N)], 167.0 [CO], 169.8 [C_5_], 171.6 [α-CONH]. (***S***)-**24c** *δ* (ppm): 23.0 [2CH2 (pyrrolidine)], 37.8 [*CH_2_*-Ph], 42.5 [CH_2_ (NH*Bn*)], 47.7 [*CH_2_*-diClPh], 48.6 [*CH_2_*-pyrrolidine], 50.4 [C_2_-*CH*], 53.5 [2CH2 (pyrrolidine)], 54.4 [C_6_], 96.8, 115.1 [2CH (Ar)], 118.0 [C (Ar)], 120.7 [CH (Ar)], 126.5, 127.4, 127.6, 128.3, 128.5, 128.7, 129.0, 129.2, 129.6 [18CH (Ar)], 131.4, 131.9, 136.5 [4C (Ar)], 137.4 [C (N*Bn*)], 139.0 [C (Ph)], 139.6 [C (NH*Bn*)], 140.2, 141.9 [2C (Ar)], 155.3 [C (NHC(NH_2_) = N)]; ES-MS *m/z* [(M+2)/2]^+^ calculated for C_54_H_62_Cl_2_N_12_O_4_: 507.2; found: 507.

## 4. Conclusions

In summary, a series of highly functionalized Phe-Gly dipeptide-derived piperazinones containing an aromatic urea moiety and a basic amino acid has been prepared and evaluated as human PAR1 antagonists in a platelet aggregation assay. The synthetic strategy involves coupling of a protected basic amino acid benzyl amide to 1,2- and 1,2,4-substituted-piperazinone derivatives, through a carbonylmethyl group at the N_1-_position, followed by formation of an aromatic urea at the exocyclic moiety linked at the C_2_ position of the piperazine ring and removal of protecting groups. In comparison with the 1,2,4,6-tetrasusbtituted-piperazinone analogues **A**, the change of position of the basic amino acid side chain from C_6_ to N_1_ in **B** has led to the complete loss of PAR1 antagonist activity and tumor cell cytotoxicity. 

## Supplementary Materials

Copies of ^1^H and ^13^C-NMR spectra for all new compounds. Supplementary materials can be accessed at: http://www.mdpi.com/1420-3049/19/4/4814/s1.
