# Supplementary material for: Exploring the Phe-Gly Dipeptide-Derived Piperazinone Scaffold in the Search for Antagonists of the Thrombin Receptor PAR1
Source: Molecules. 2014 Apr 16;19(4):4814–46. doi: 10.3390/molecules19044814 (PMC6271095; doi:10.3390/molecules19044814)

Supplementary Material

Spectra (^{1}H-NMR and ^{13}C-NMR) of New Compounds

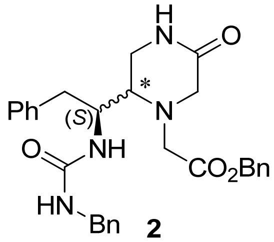

2

2 (300 MHz, $CDCl_3$)

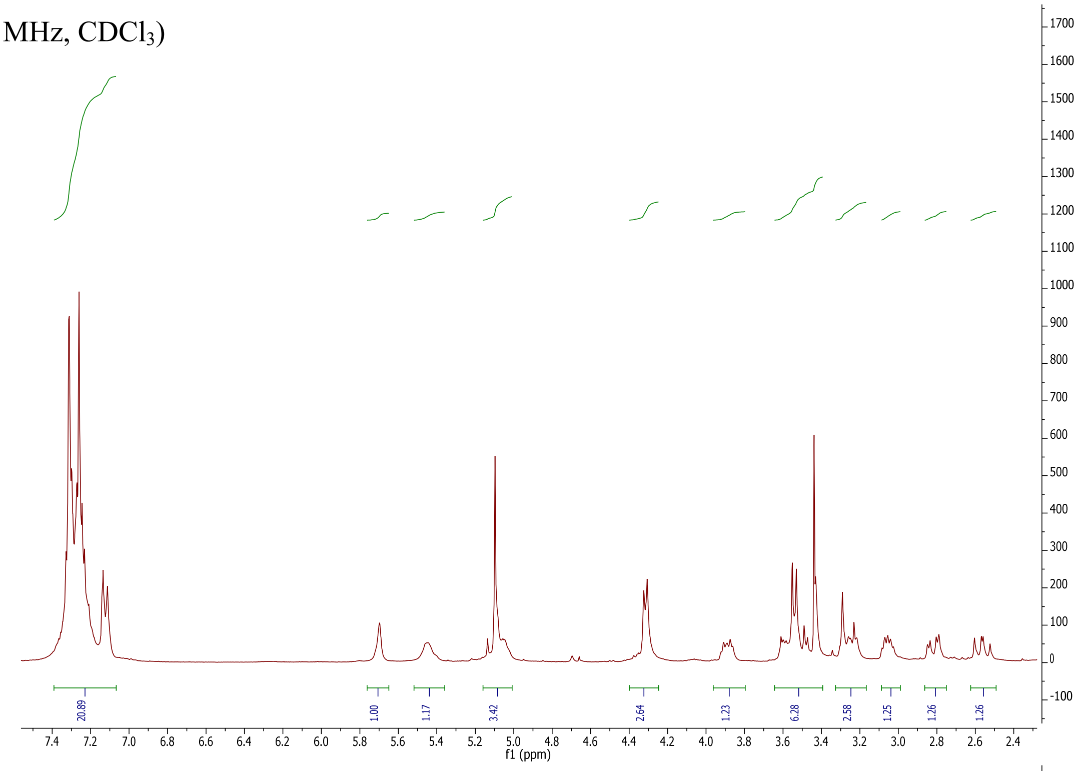

2 (75 MHz, $CDCl_3$)

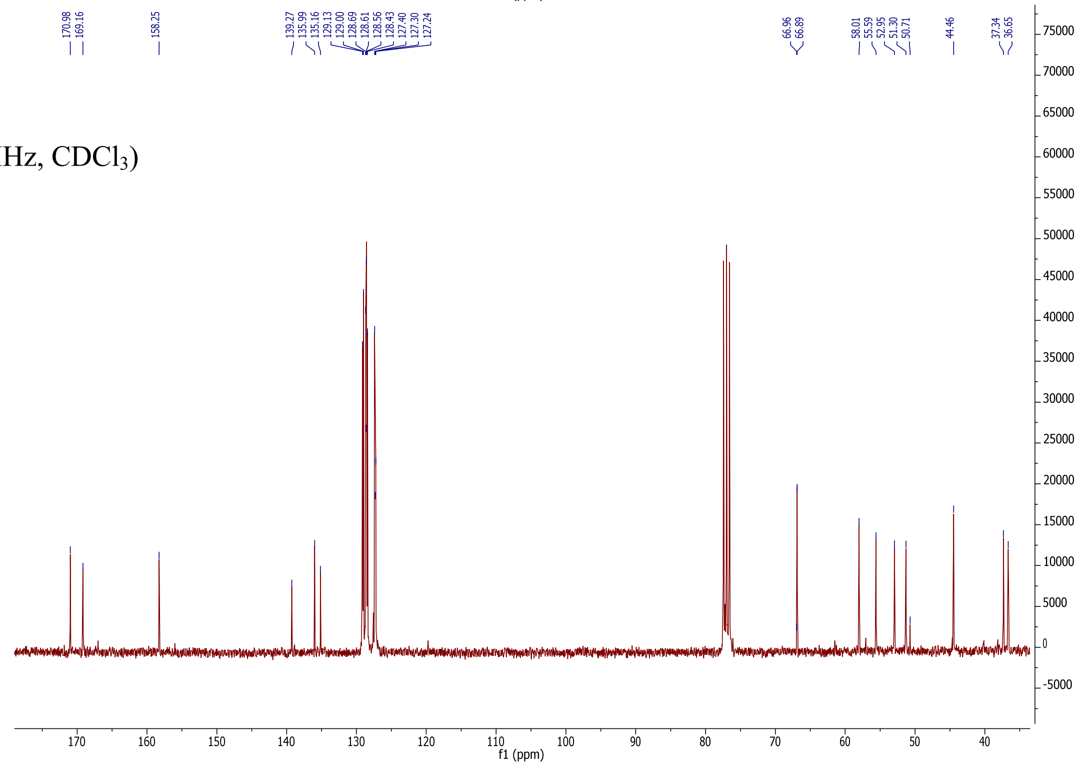

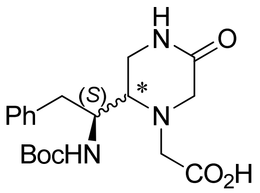

4

4 (500 MHz, DMSO-d_6)

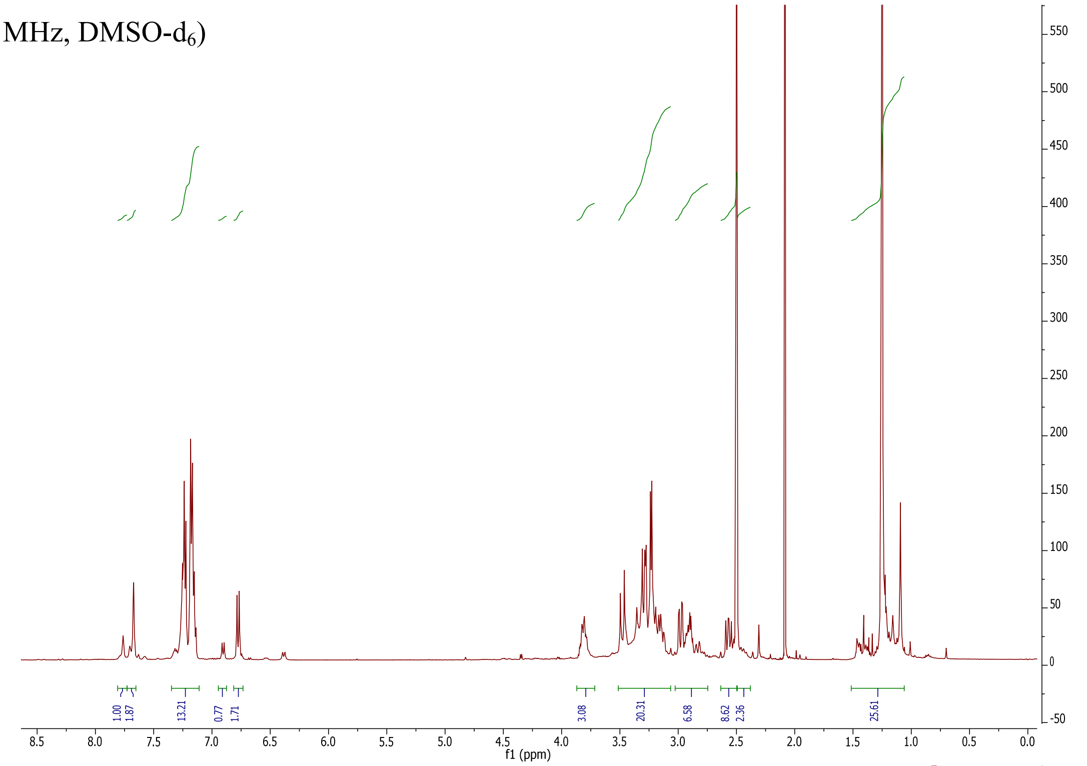

4 (125 MHz, DMSO-d_6)

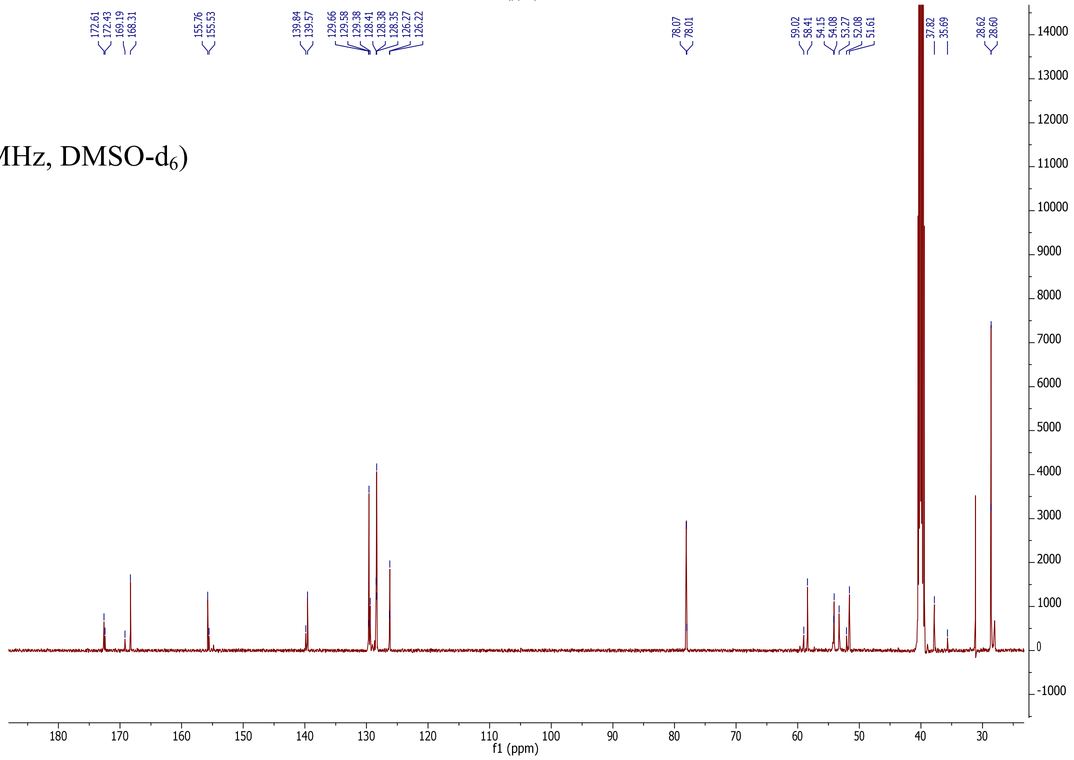

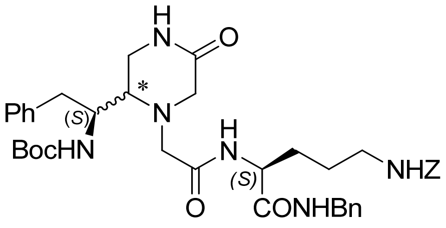

7a

7a (400 MHz, $CDCl_3$)

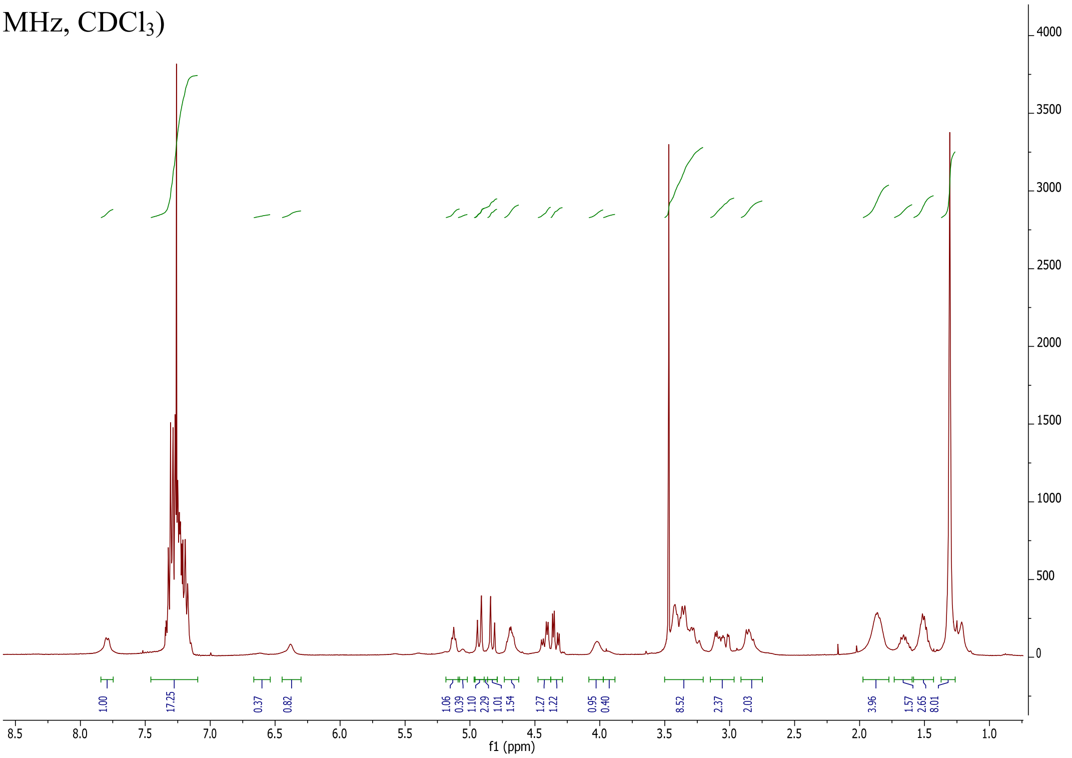

7a (100 MHz, $CDCl_3$)

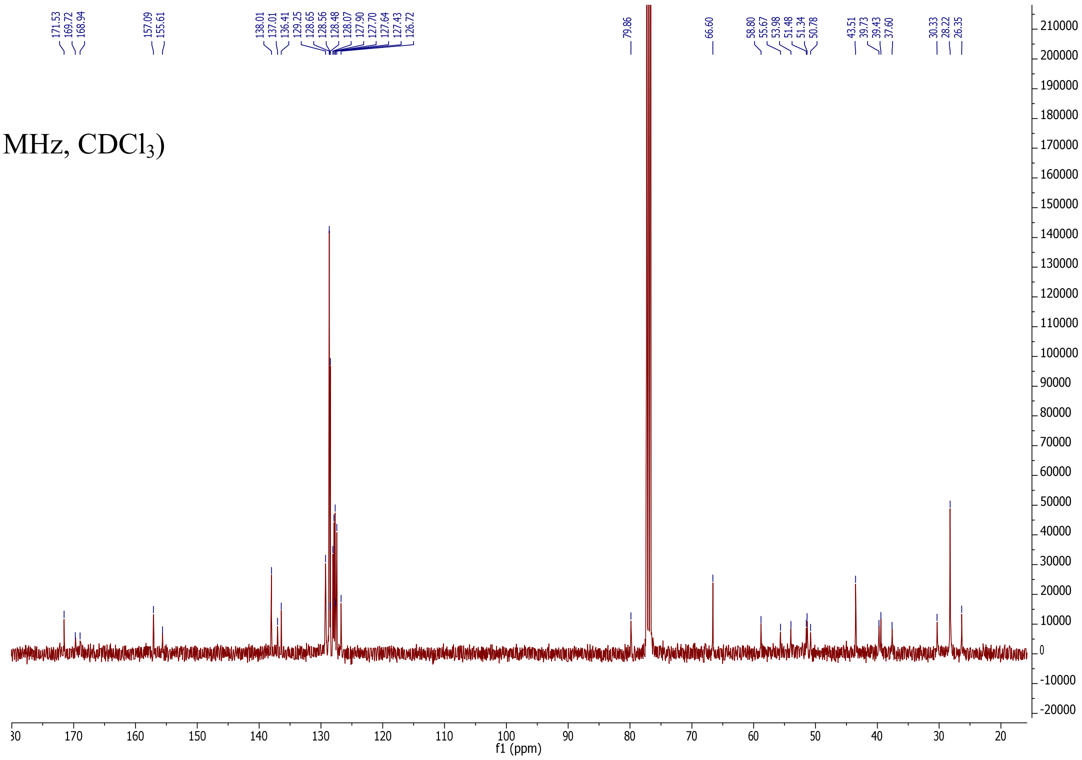

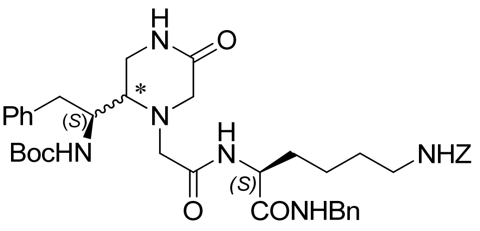

7b

7b (400 MHz, $CDCl_3$)

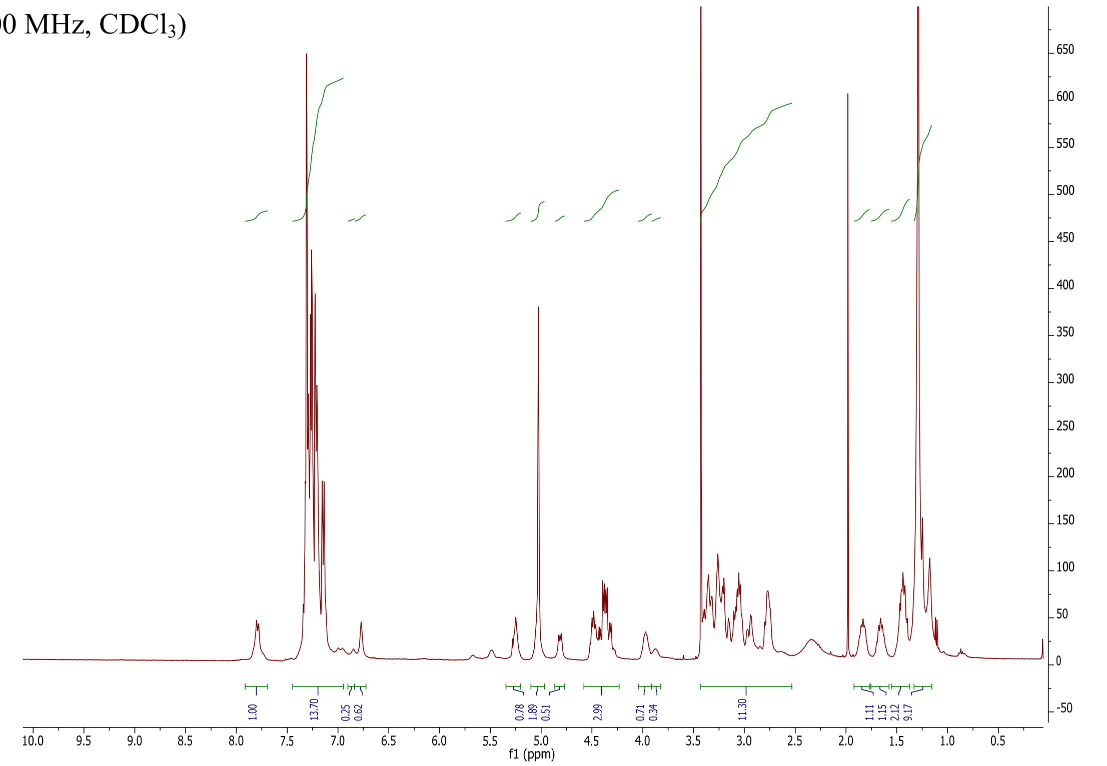

7b (100 MHz, $CDCl_3$)

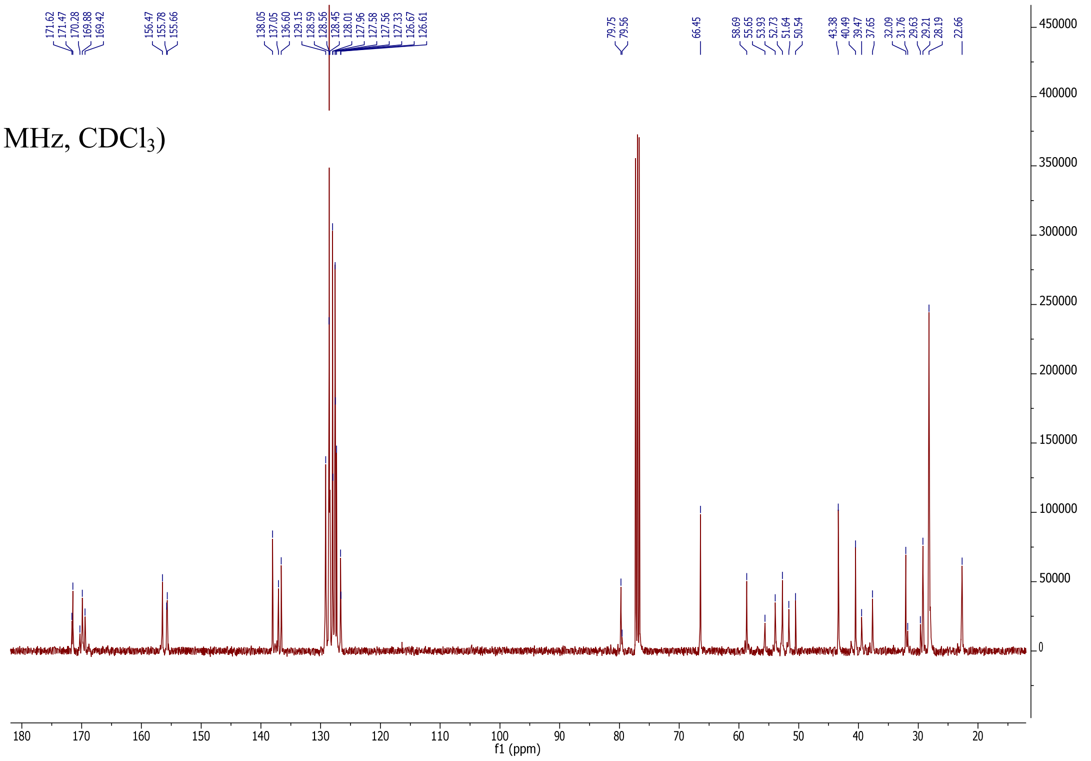

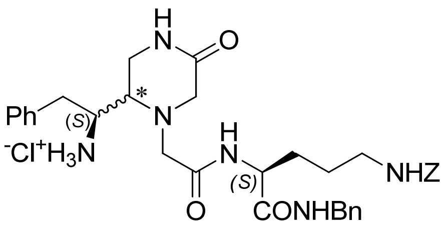

8a

8a (400 MHz, DMSO-d_6)

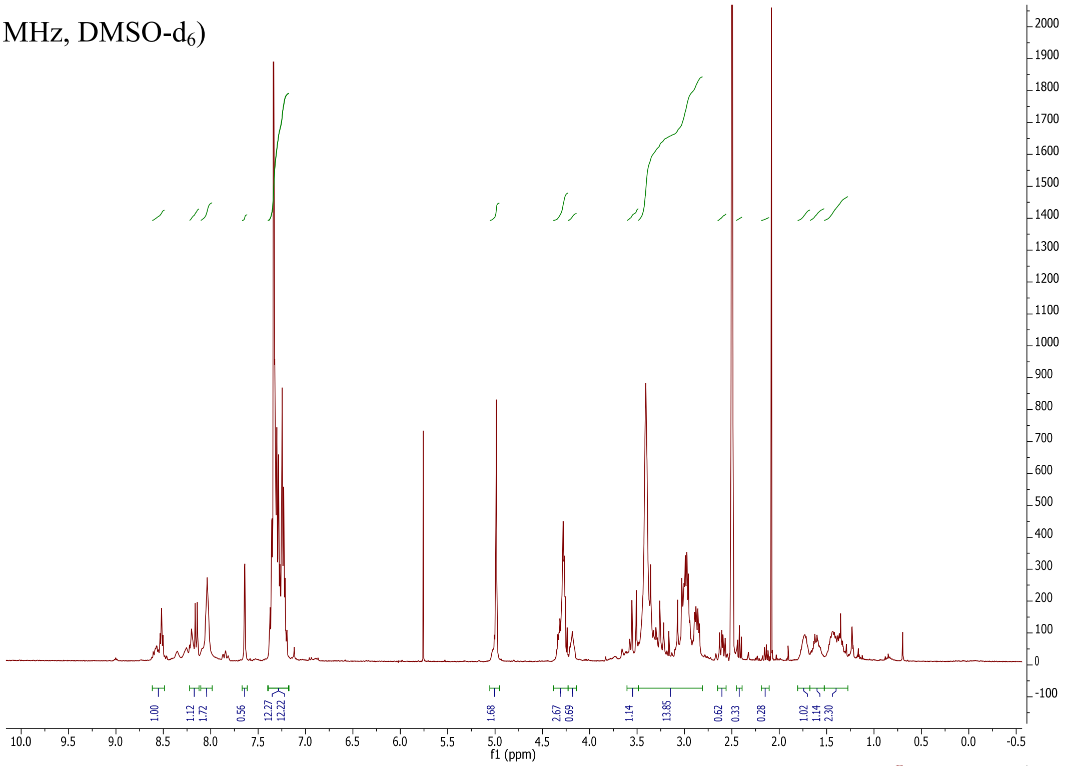

8a (100 MHz, DMSO-d_6)

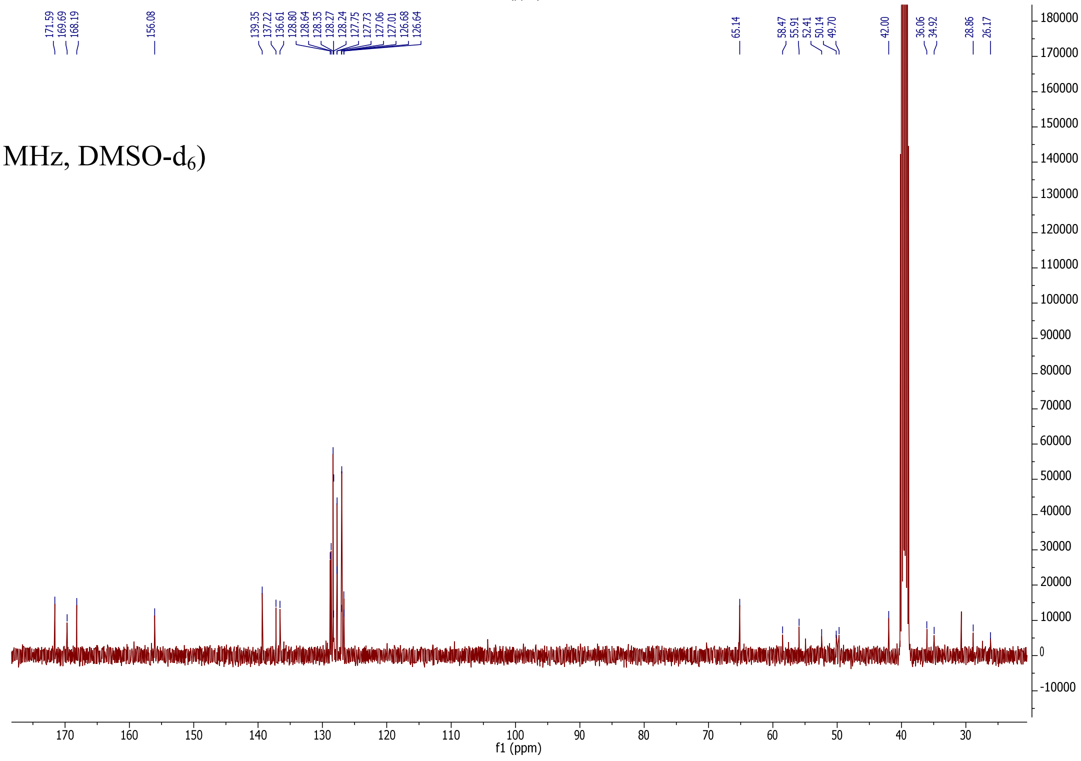

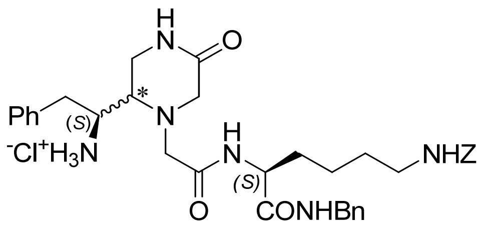

8b

8b (400 MHz, DMSO-d$_6$)

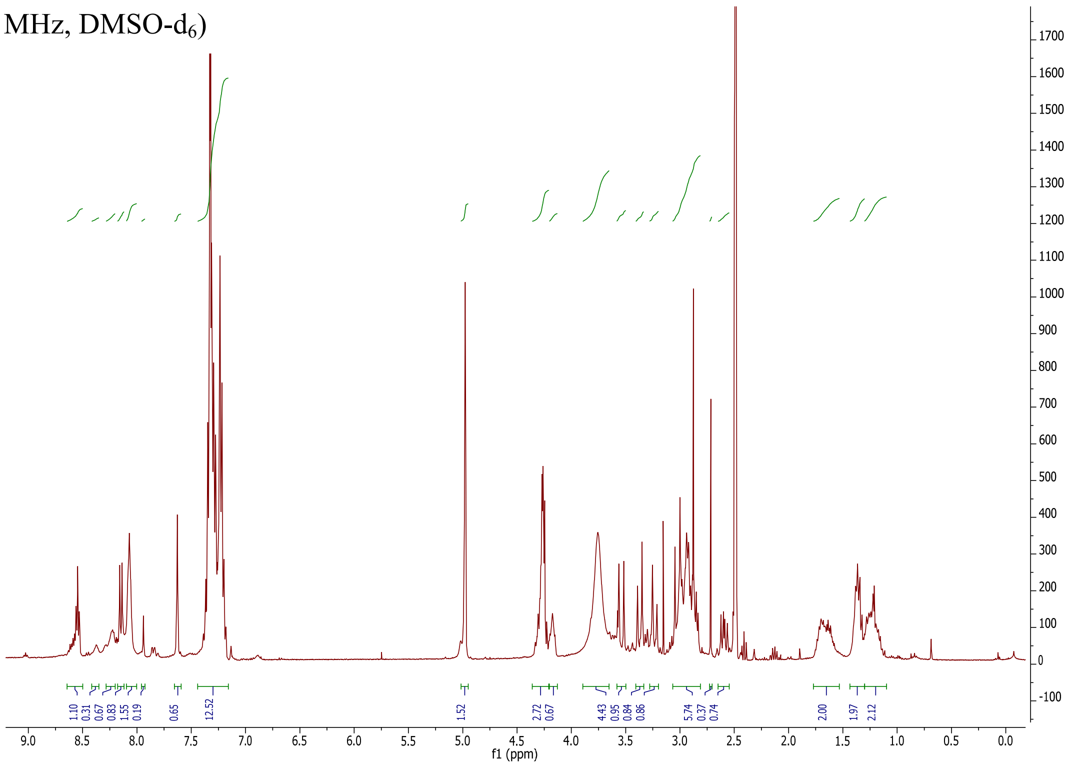

8b (100 MHz, DMSO-d$_6$)

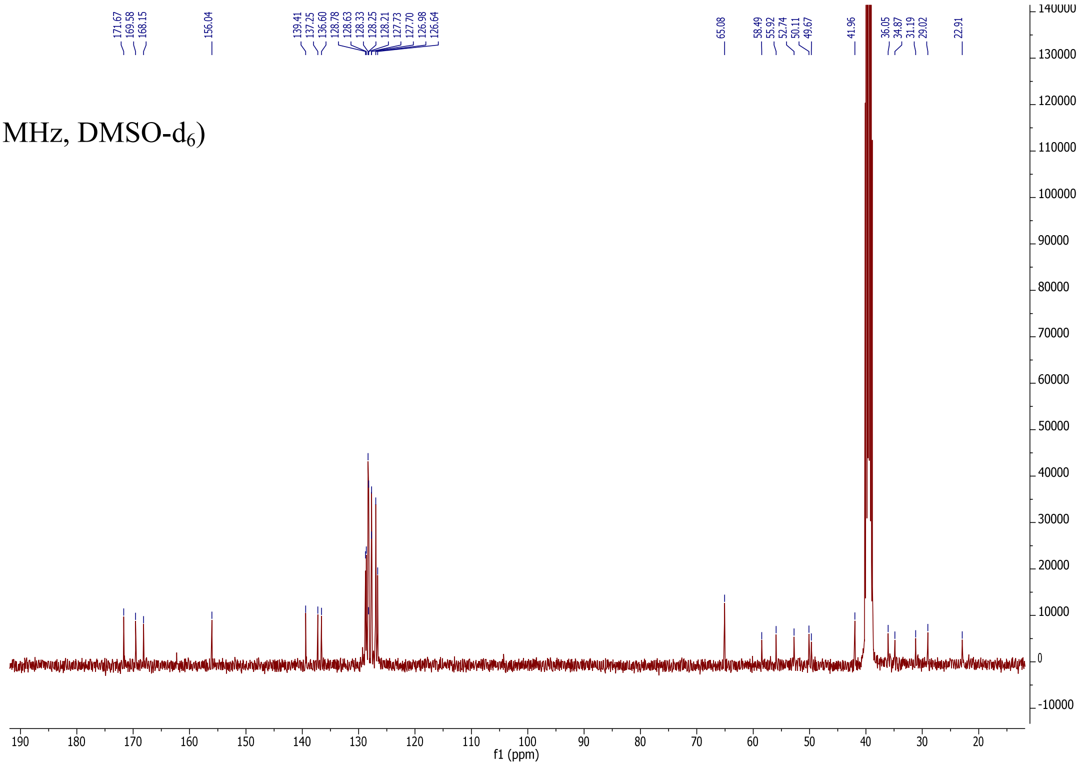

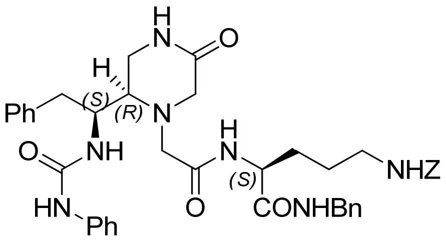

(*R*)-9a

(*R*)-9a (400 MHz, $CDCl_3$)

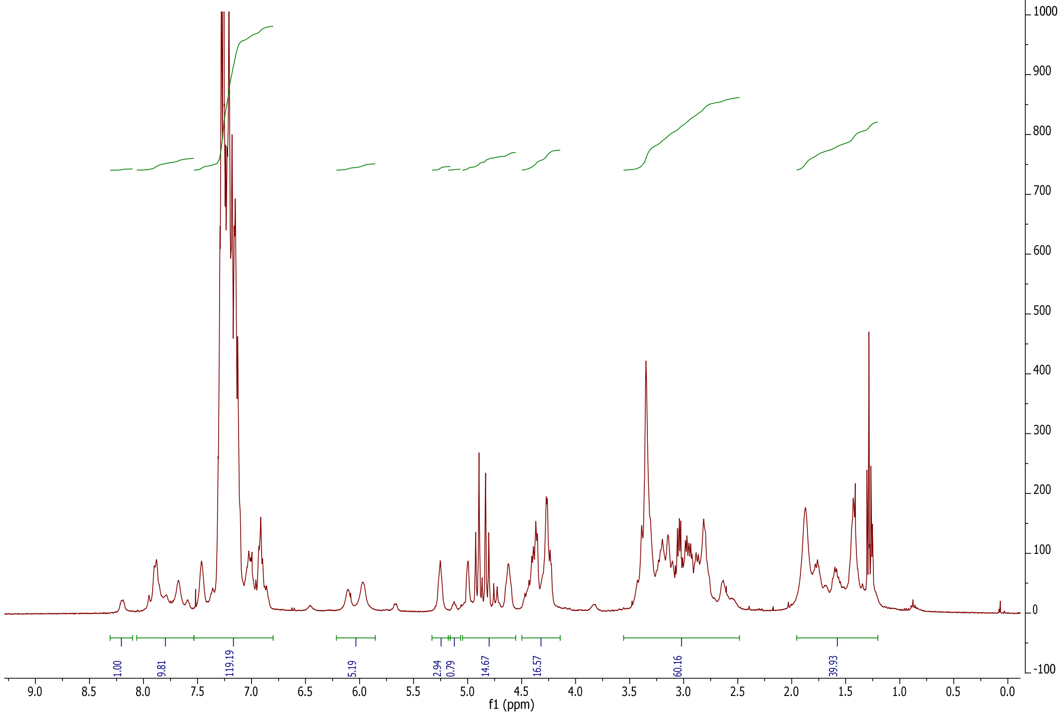

(*R*)-9a (100 MHz, $CDCl_3$)

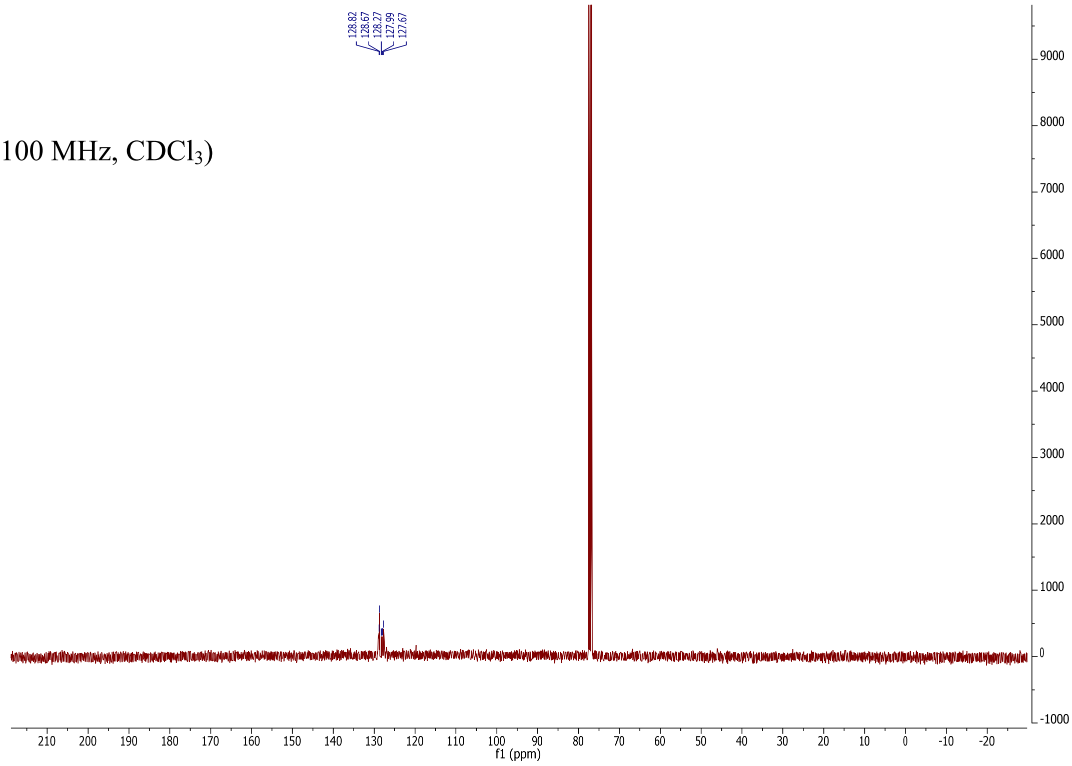

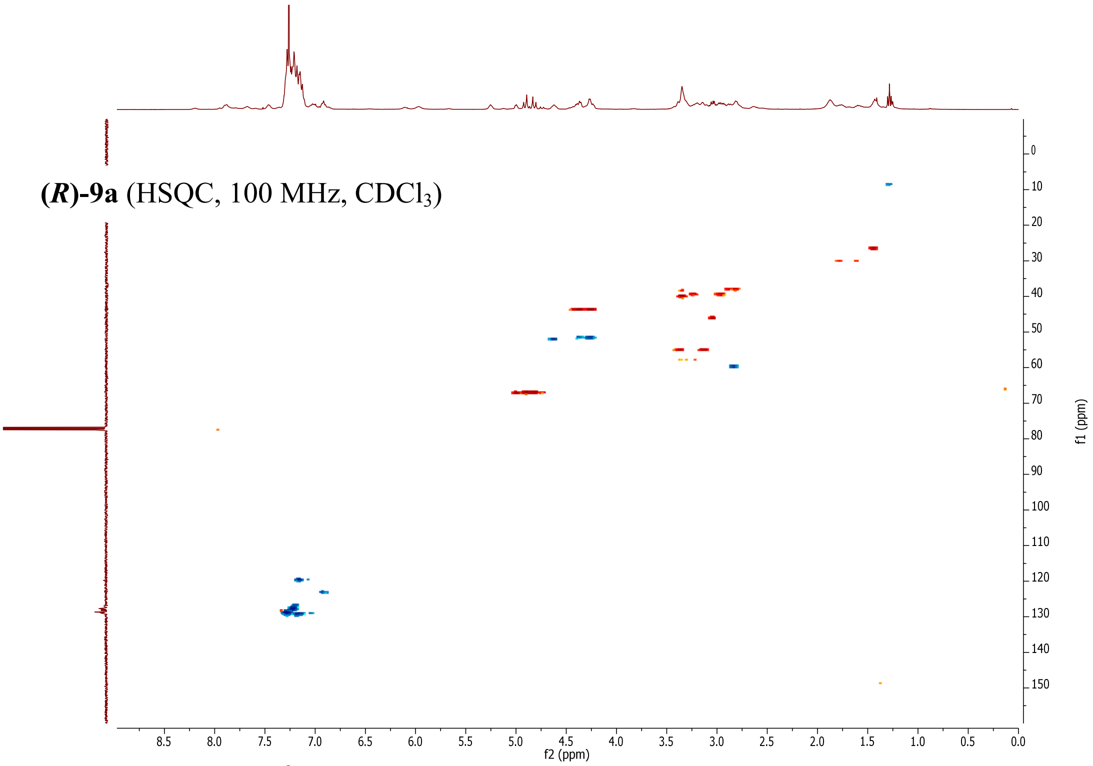
(R)-9a (HSQC, 100 MHz, CDCl3)
f2 (ppm)
f1 (ppm)

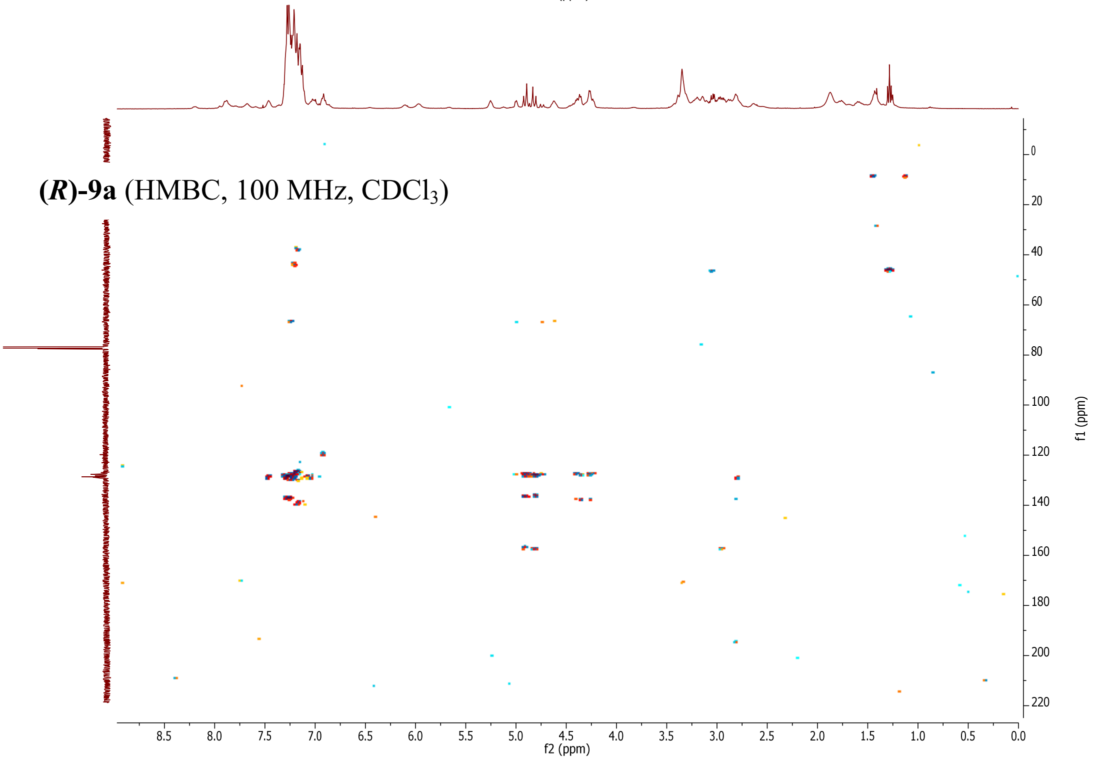
(R)-9a (HMBC, 100 MHz, CDCl3)
f2 (ppm)
f1 (ppm)

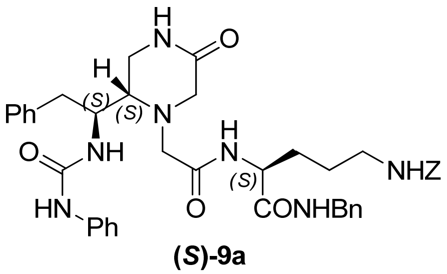

(*S*)-9a

(*S*)-9a (500 MHz, $CDCl_3$)

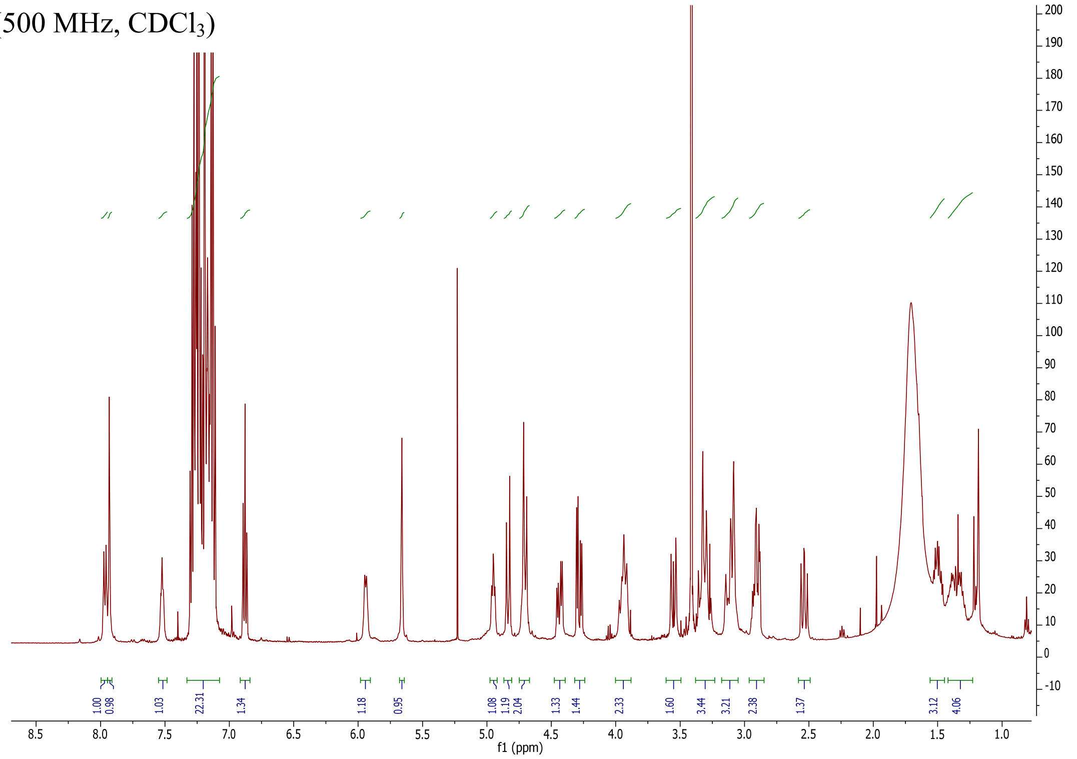

(*S*)-9a (500 MHz, $CDCl_3$)

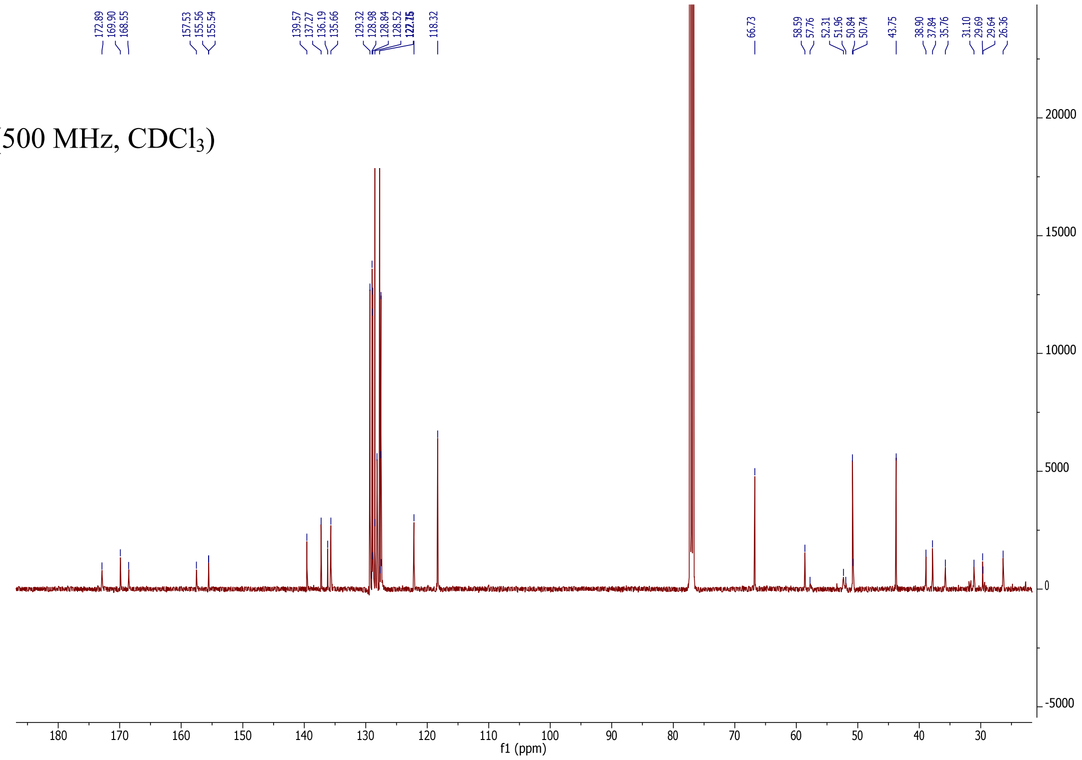

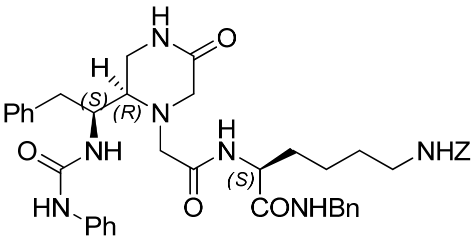

(*R*)-9b

(*R*)-9b (400 MHz, $CDCl_3$)

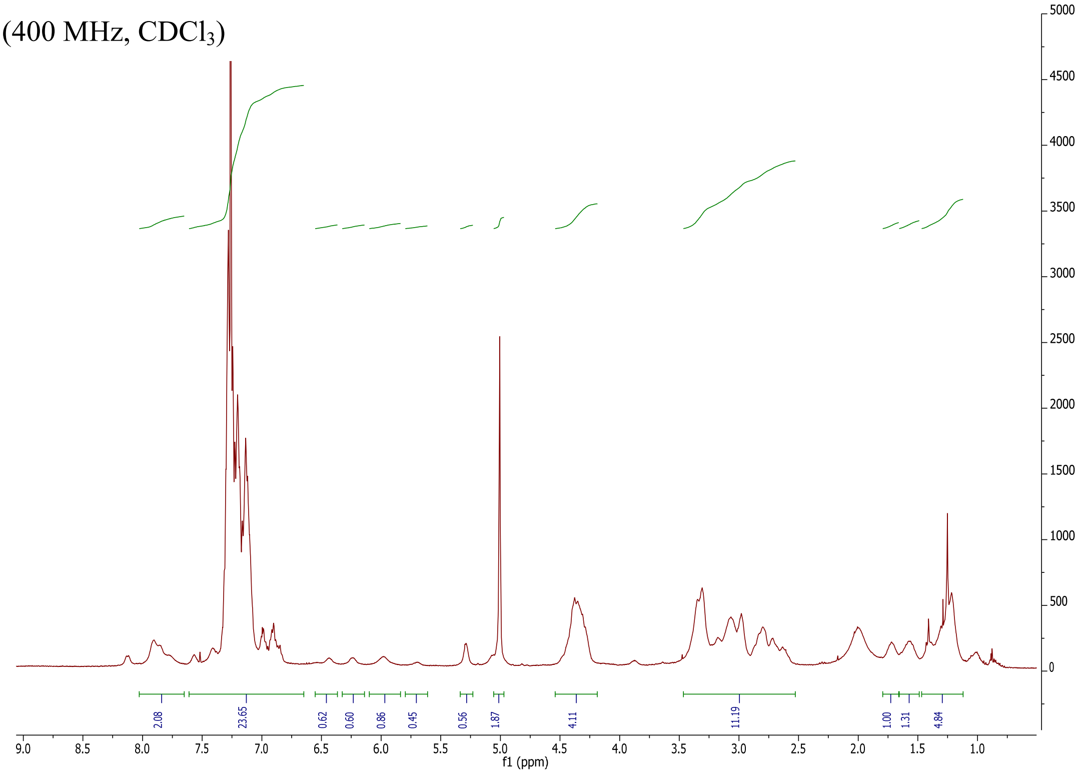

(*R*)-9b (100 MHz, $CDCl_3$)

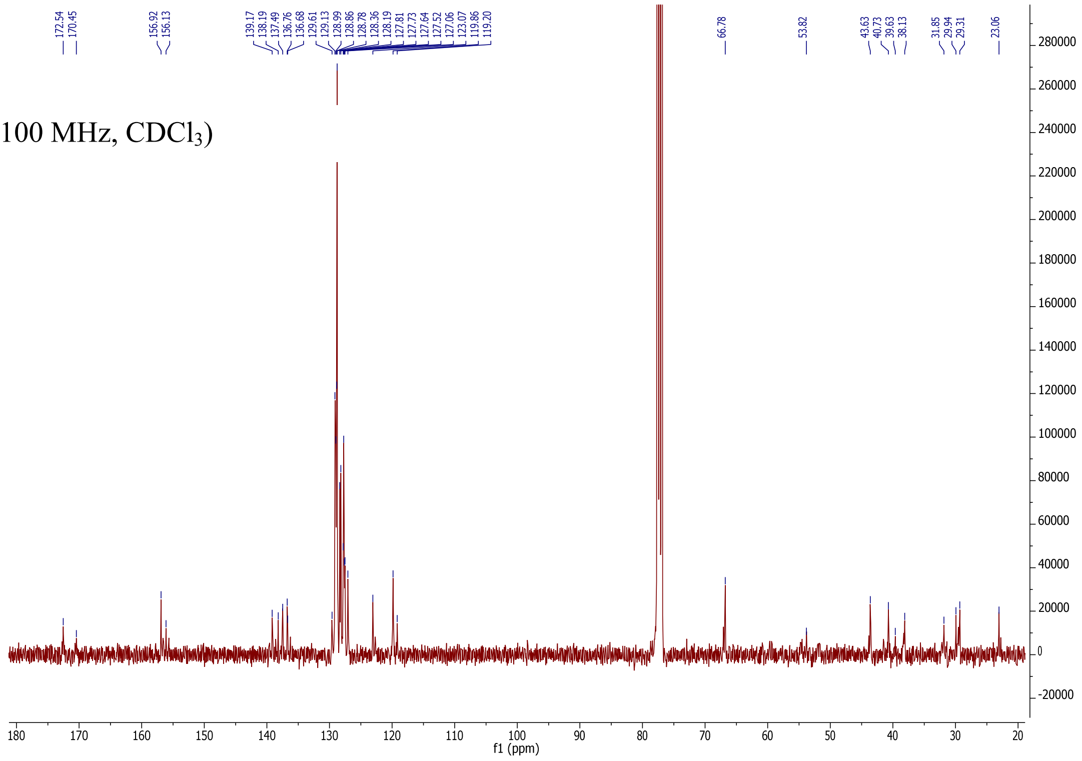

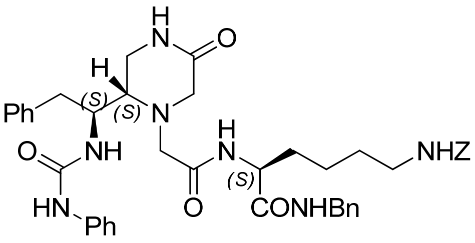

(*S*)-9b

(*S*)-9b (500 MHz, $CDCl_3$)

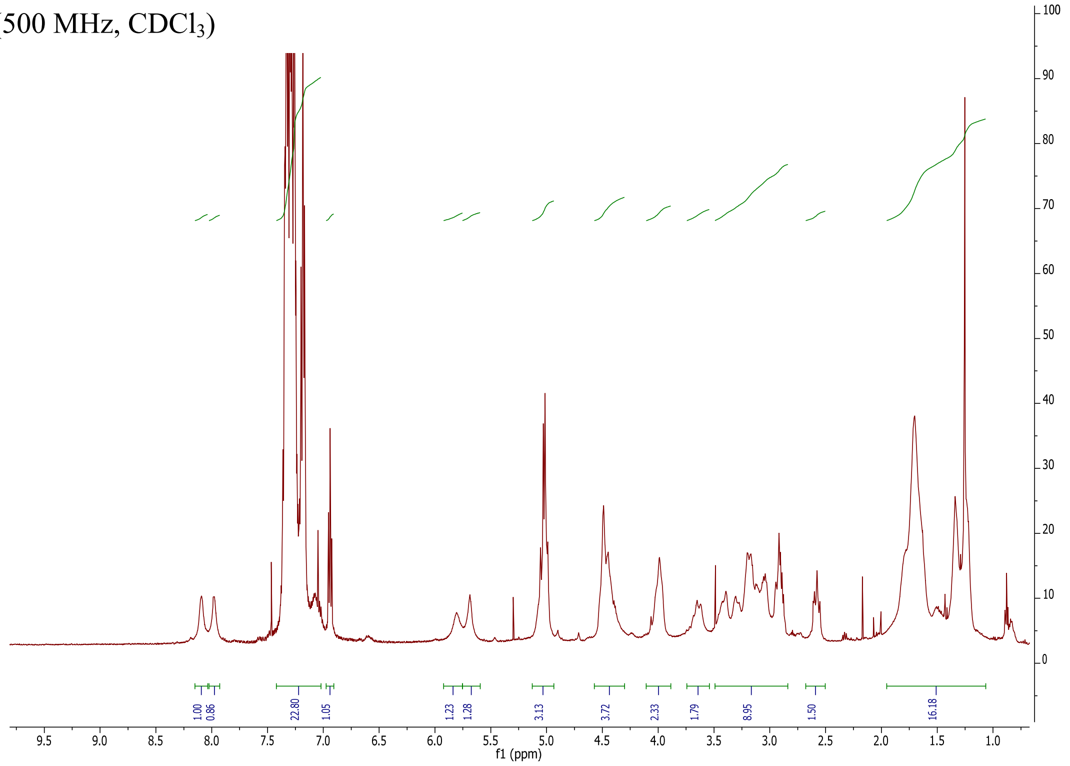

(*S*)-9b (125 MHz, $CDCl_3$)

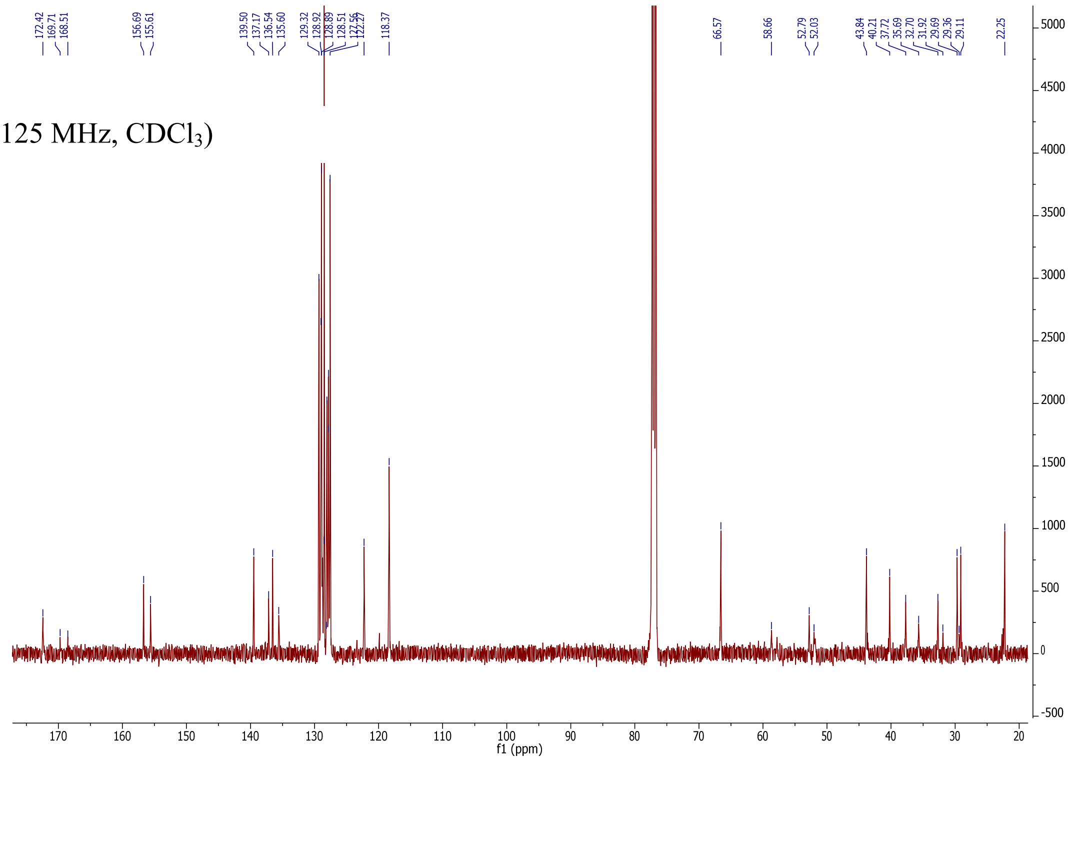

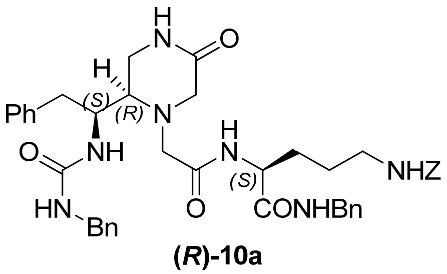

(*R*)-10a

(*R*)-10a (400 MHz, CDCl$_3$)

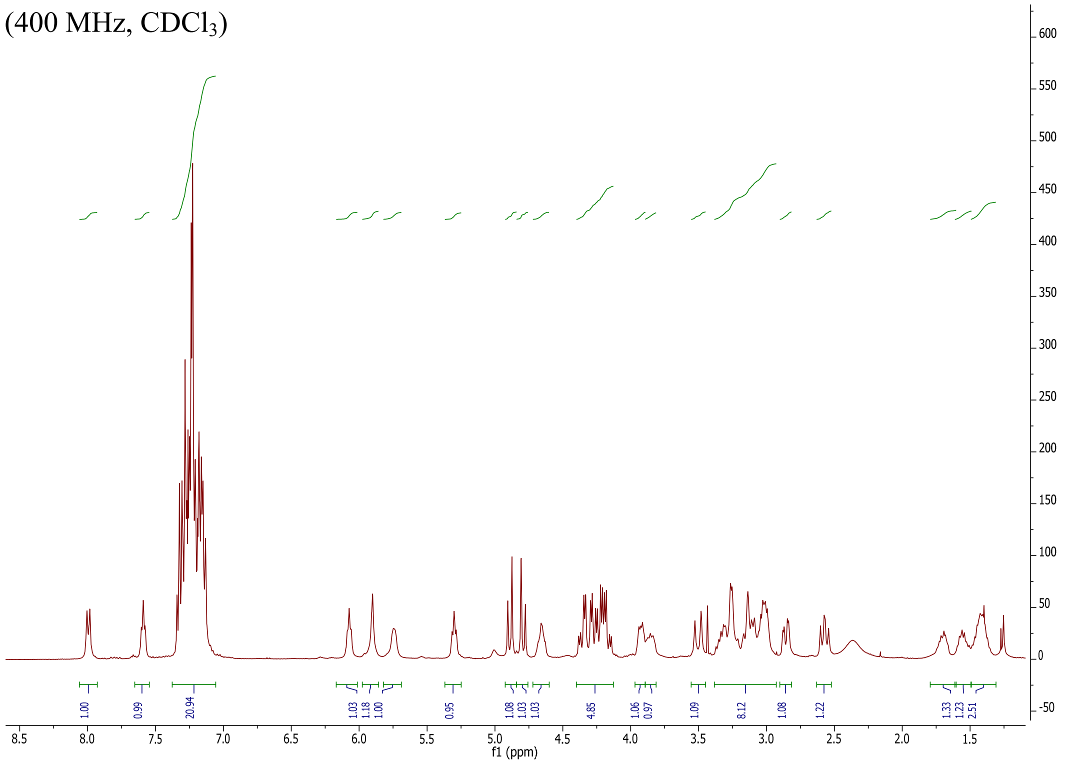

(*R*)-10a (100 MHz, CDCl$_3$)

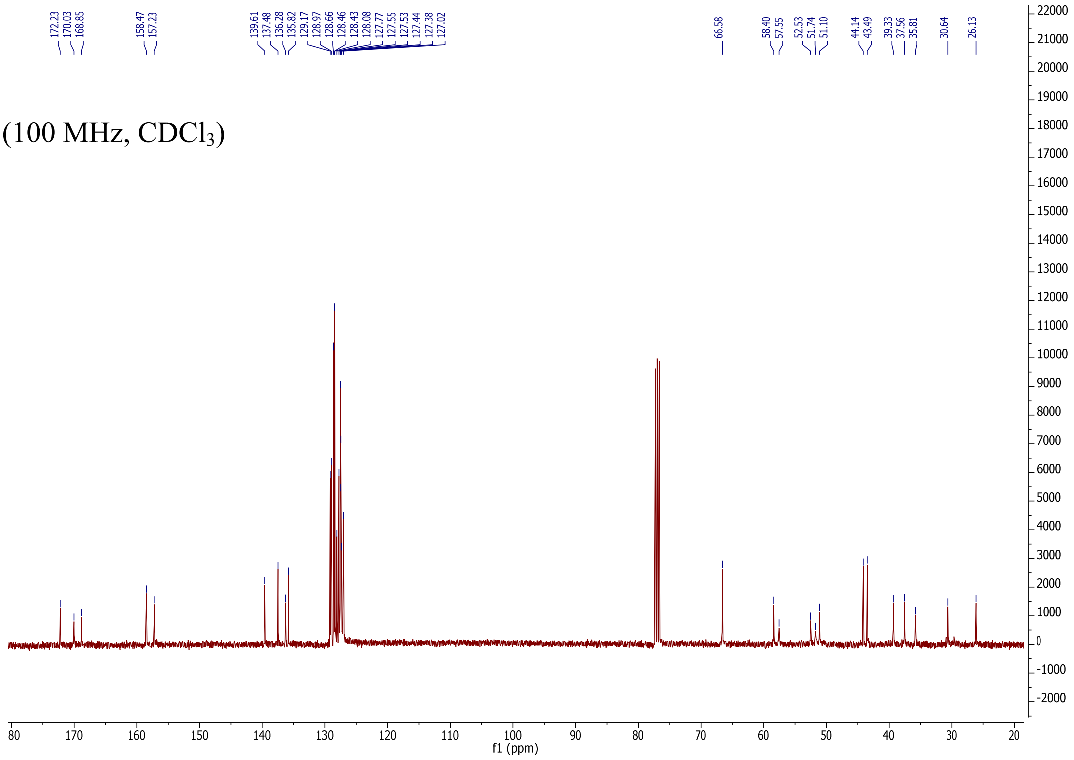

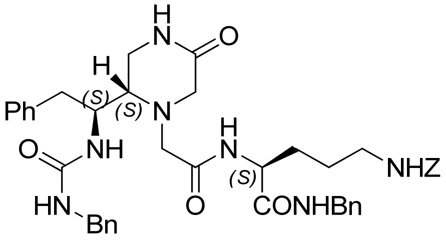

(*S*)-10a

(*S*)-10a (500 MHz, $(CD_3)_2CO$)

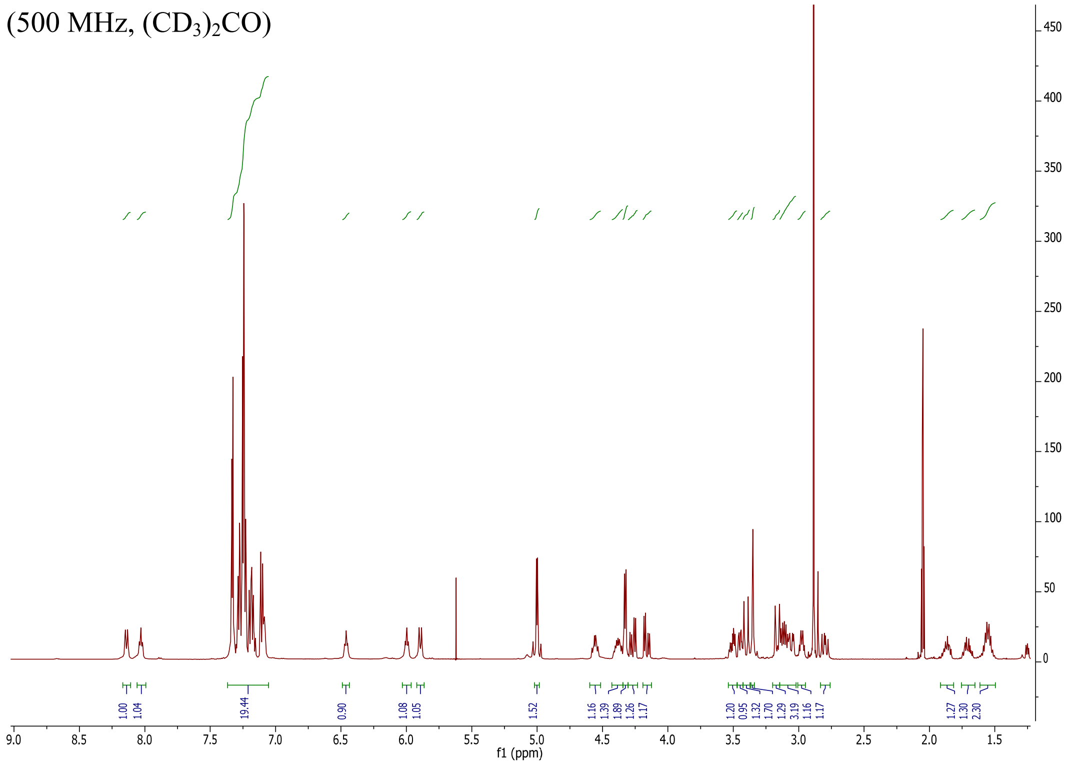

(*S*)-10a (125 MHz, $(CD_3)_2CO$)

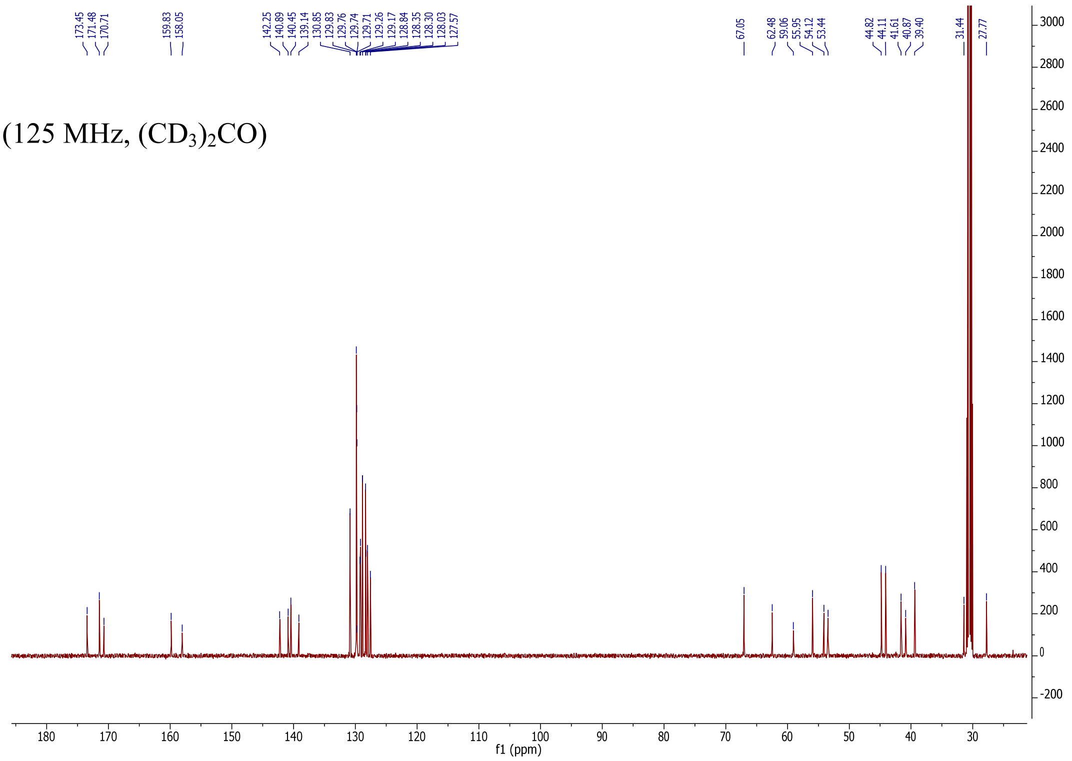

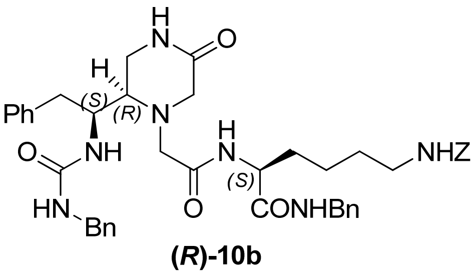

(*R*)-10b

(*R*)-10b (400 MHz, $CDCl_3$)

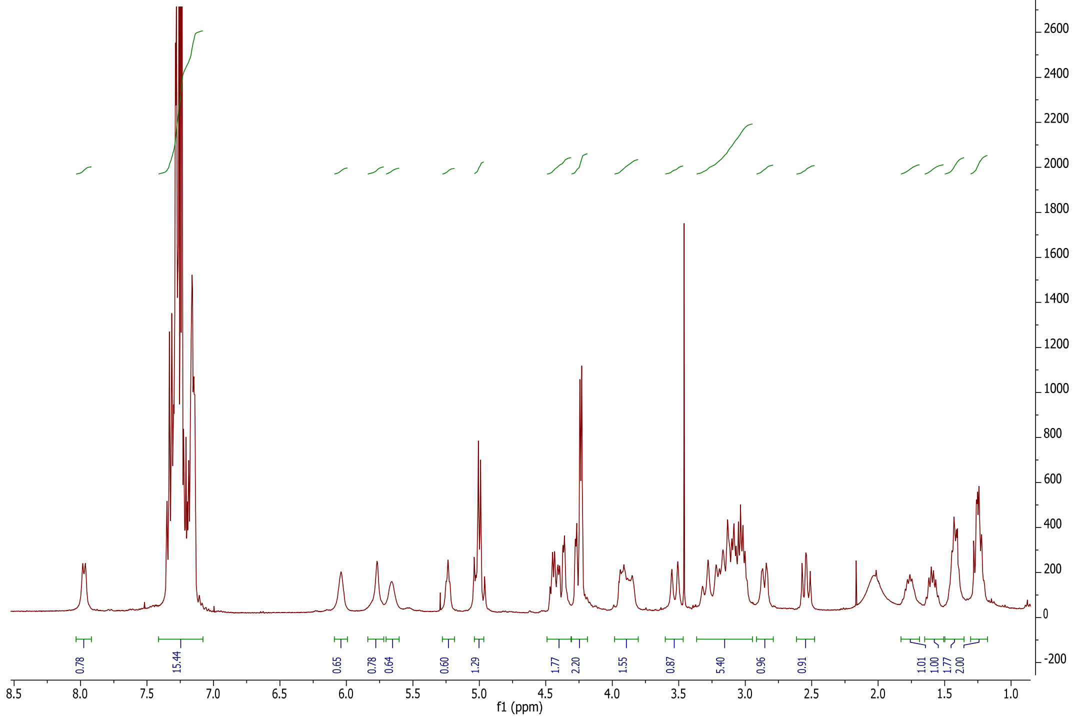

(*R*)-10b (100 MHz, $CDCl_3$)

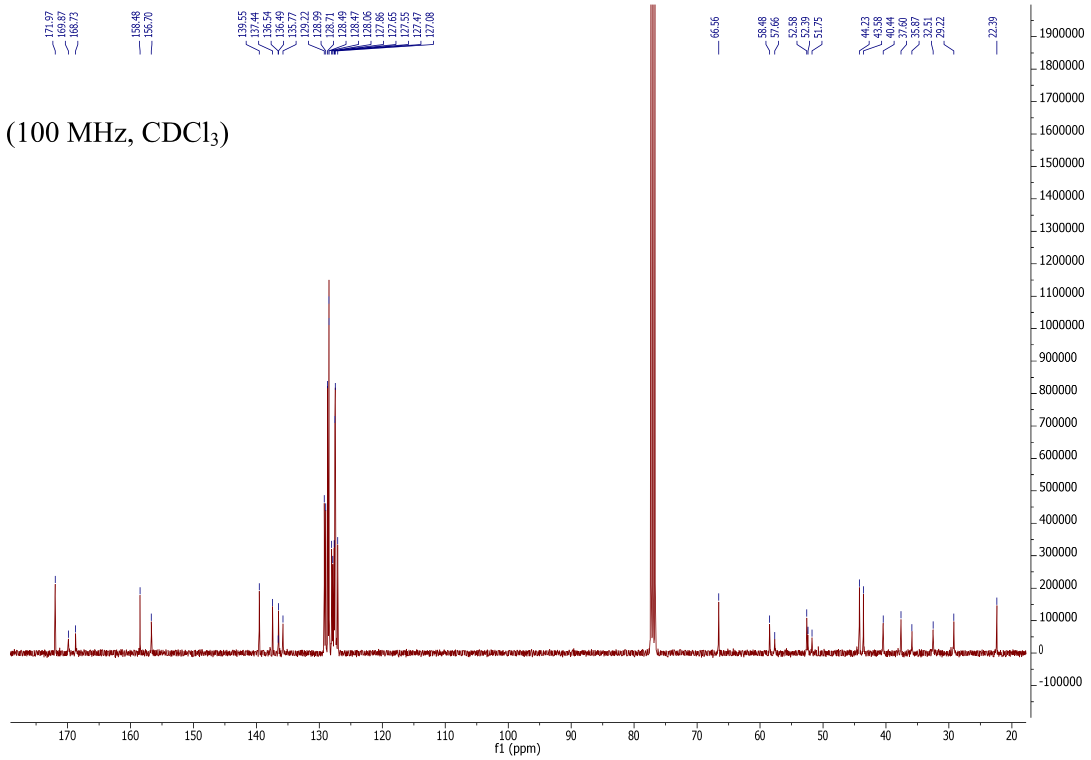

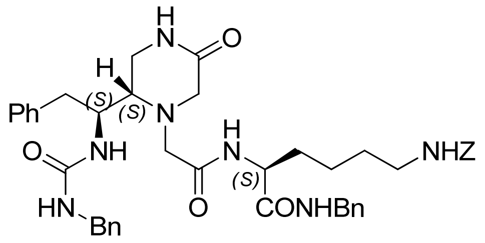

(*S*)-10b

(*S*)-10b (500 MHz, $(CD_3)_2CO$)

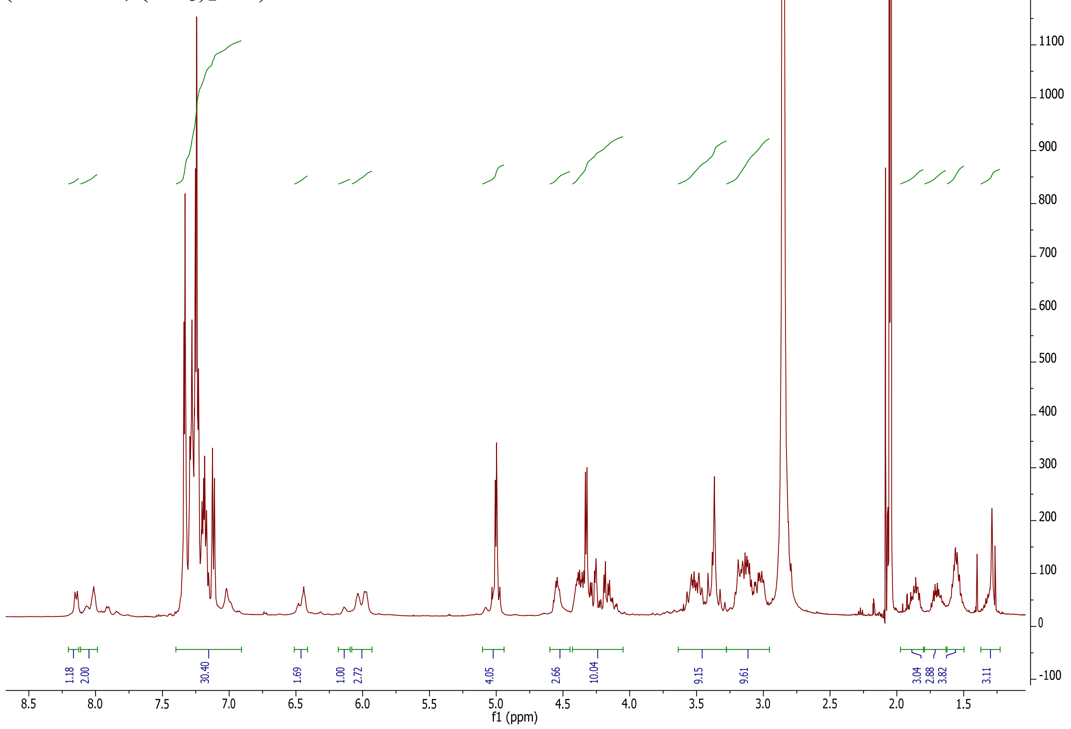

(*S*)-10b (125 MHz, $(CD_3)_2CO$)

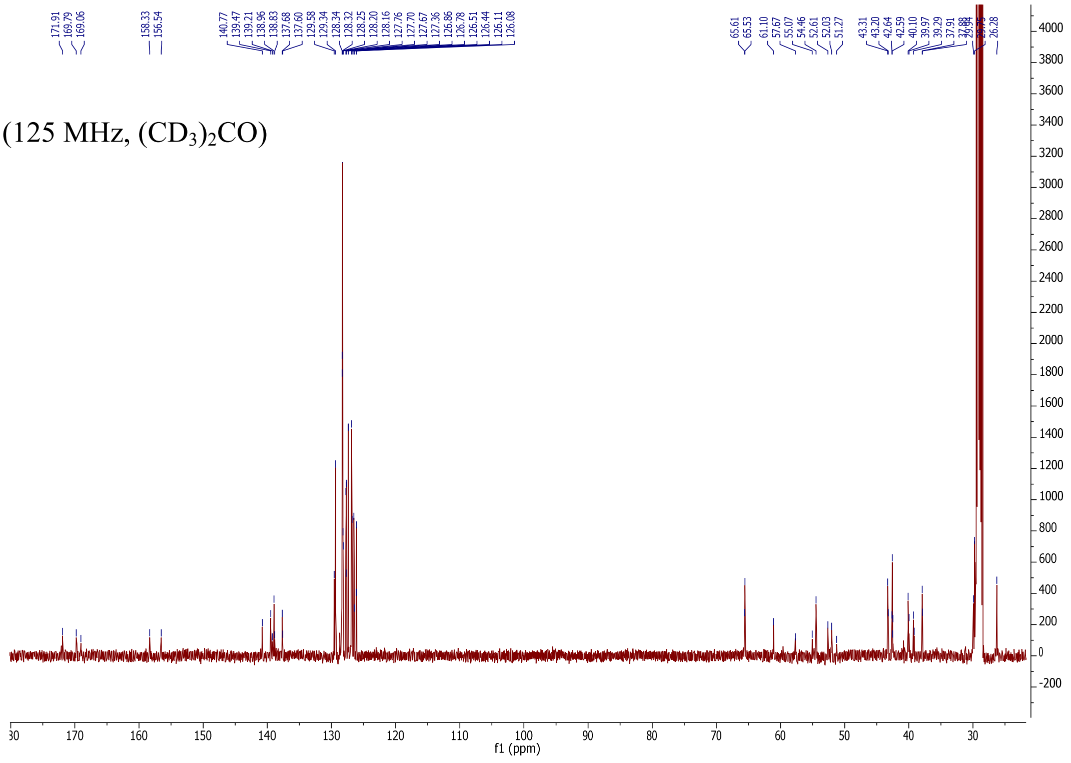

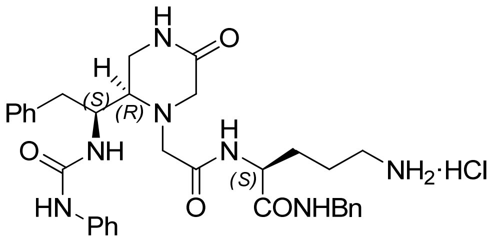

(*R*)-11a

(*R*)-11a (500 MHz, DMSO-d$_6$)

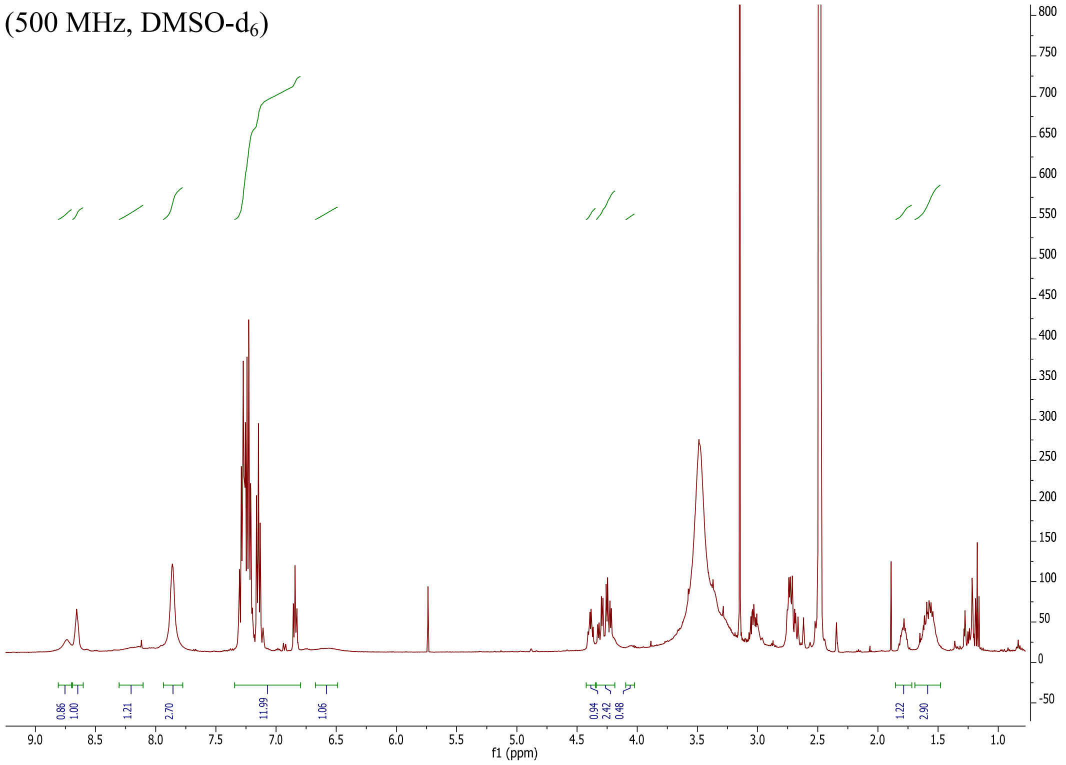

(*R*)-11a (125 MHz, DMSO-d$_6$)

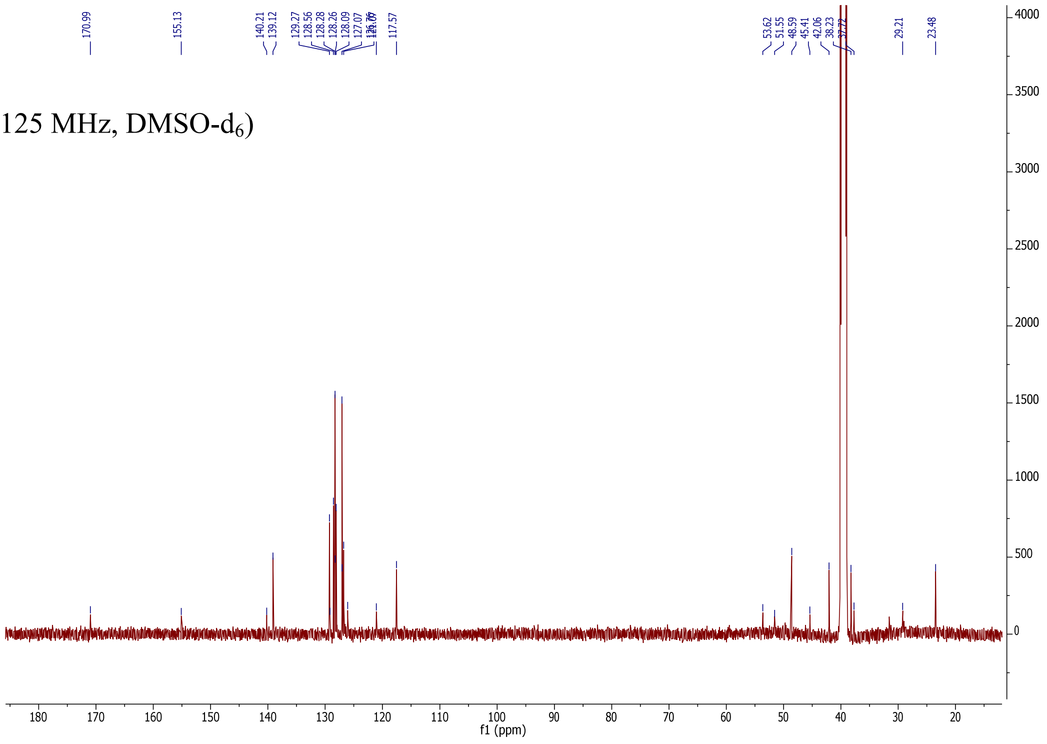

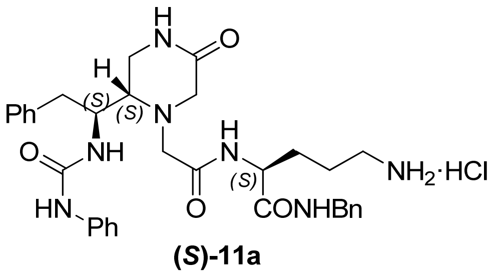

(*S*)-11a

(*S*)-11a (500 MHz, DMSO-d$_6$)

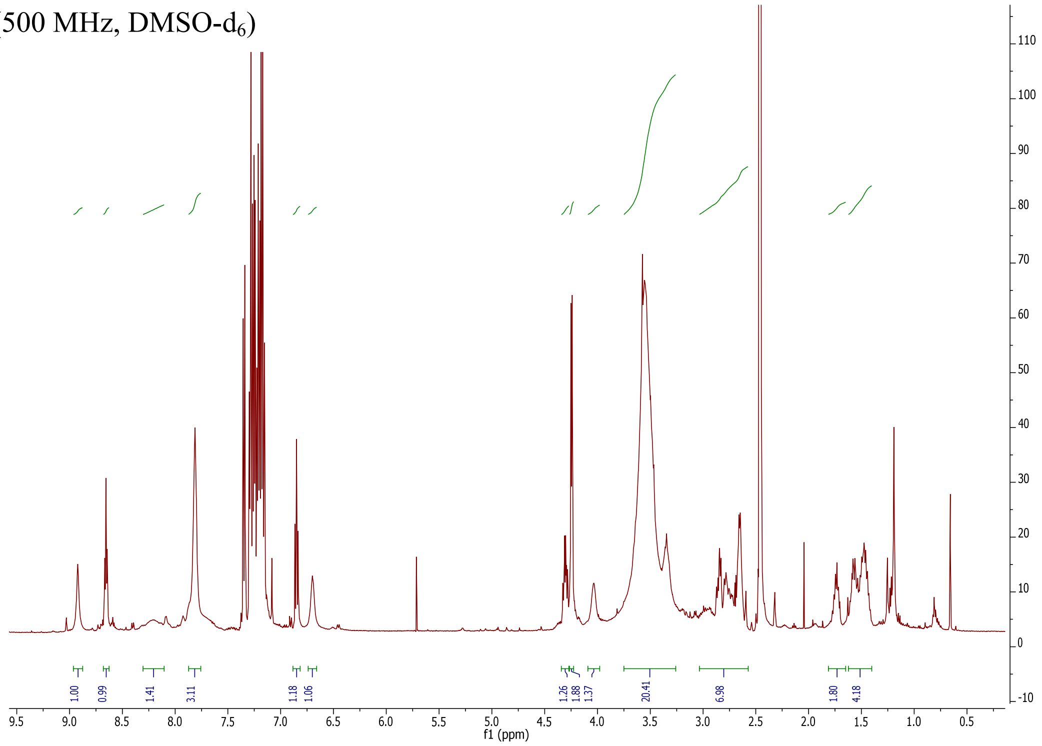

(*S*)-11a (125 MHz, DMSO-d$_6$)

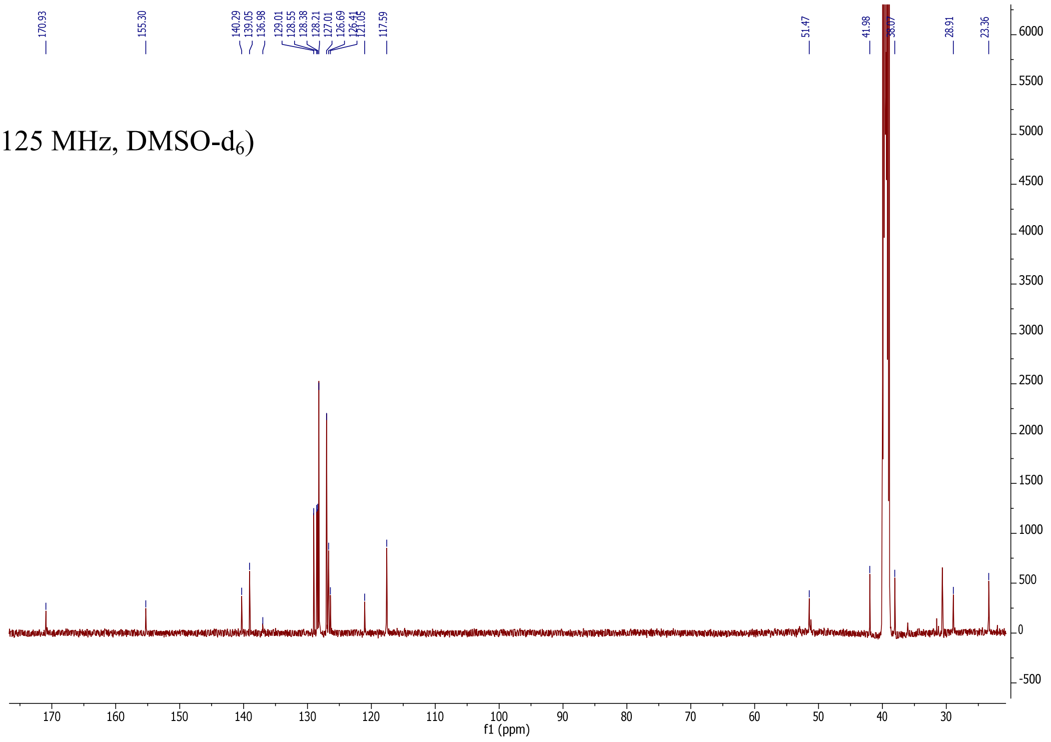

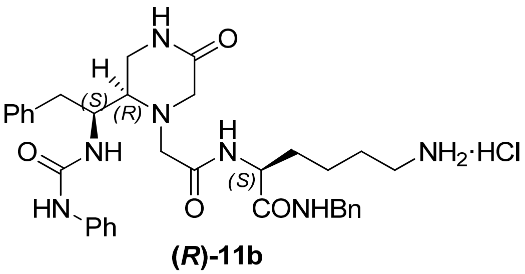

(*R*)-11b

(*R*)-11b (500 MHz, DMSO-d$_6$)

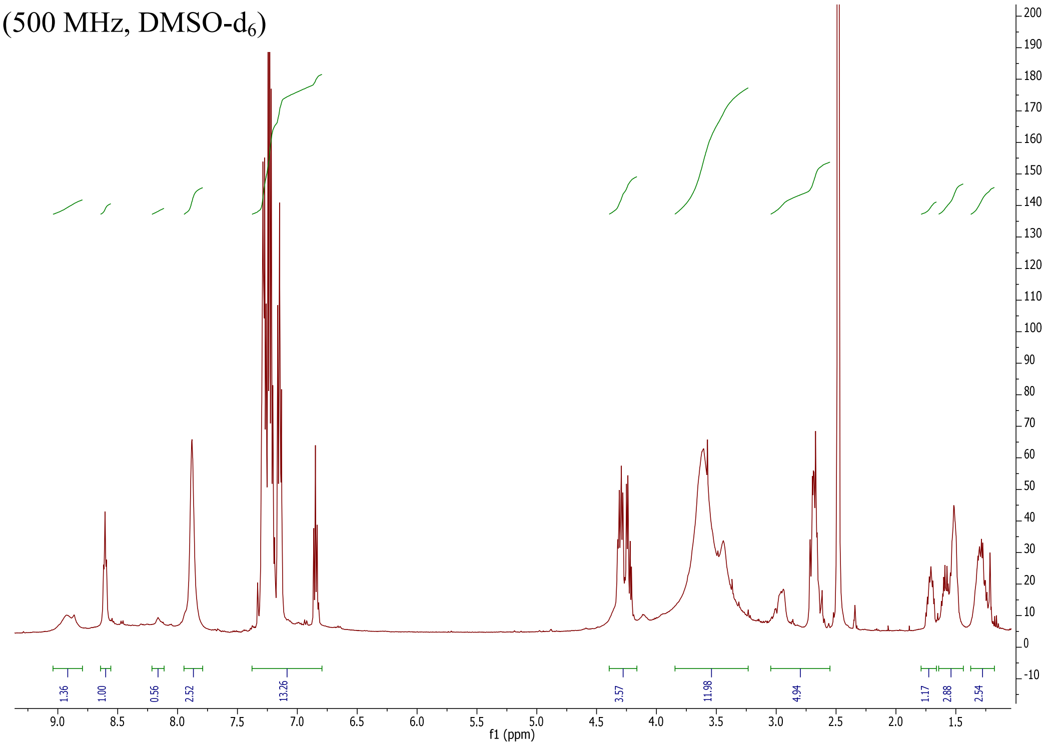

(*R*)-11b (125 MHz, DMSO-d$_6$)

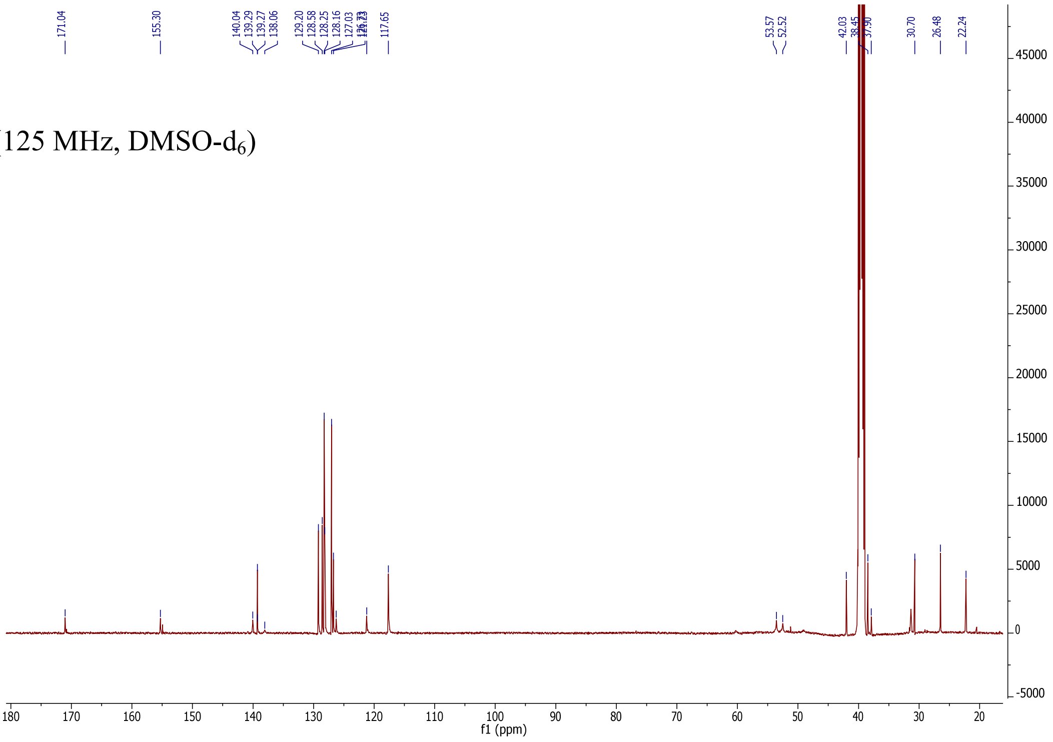

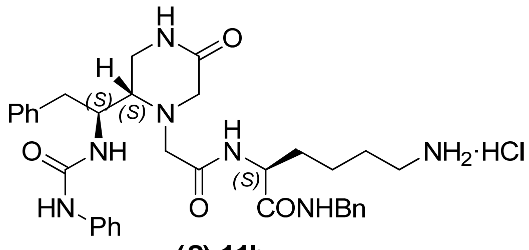

(*S*)-11b

(*S*)-11b (500 MHz, DMSO-d_6)

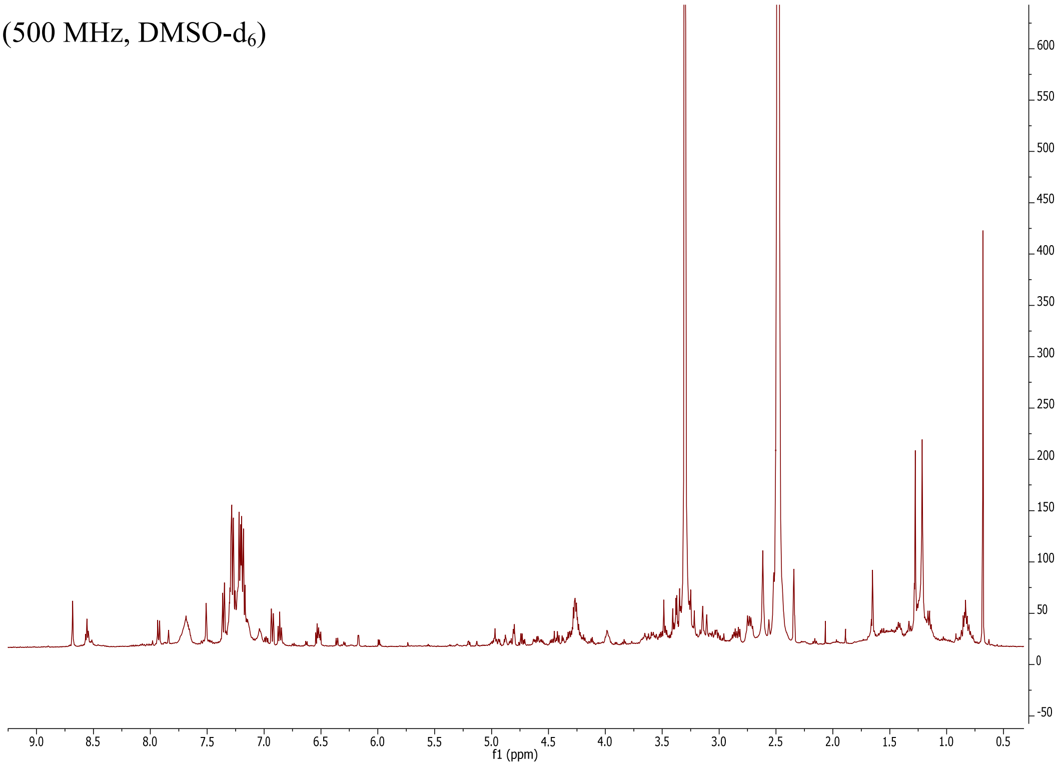

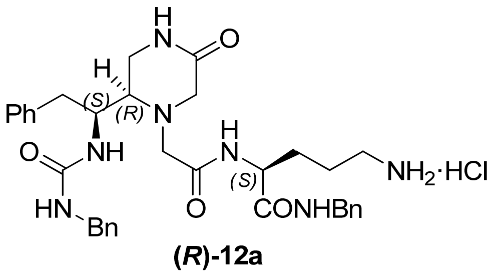

(*R*)-12a

(*R*)-12a (500 MHz, DMSO-d$_6$)

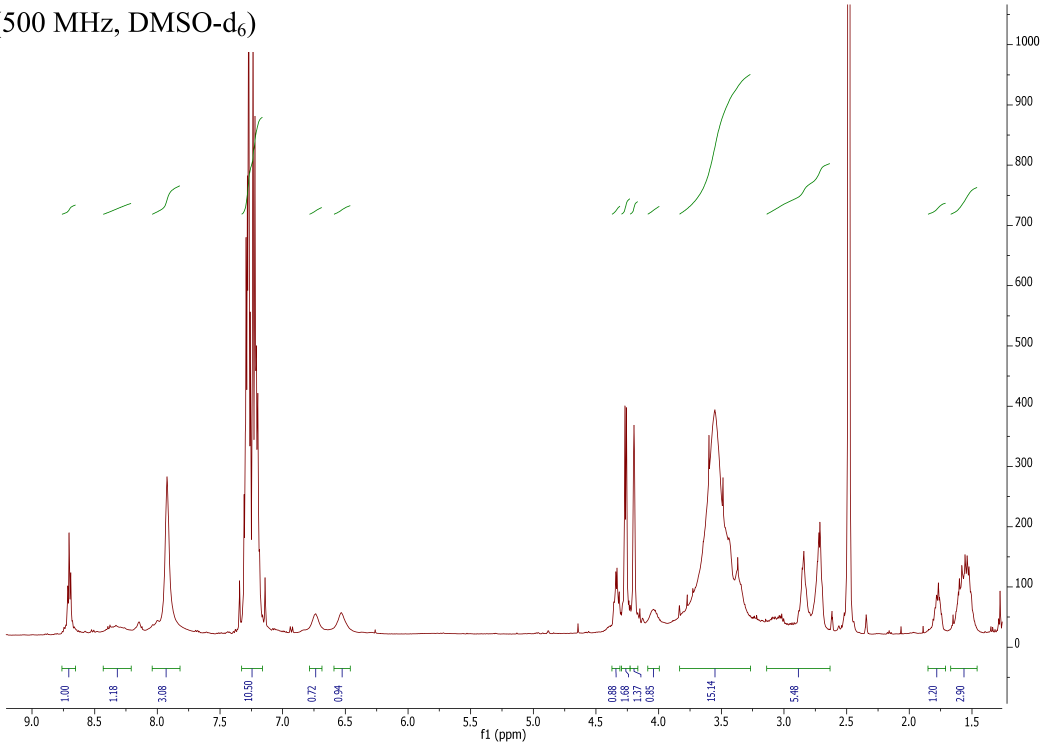

(*R*)-12a (125 MHz, DMSO-d$_6$)

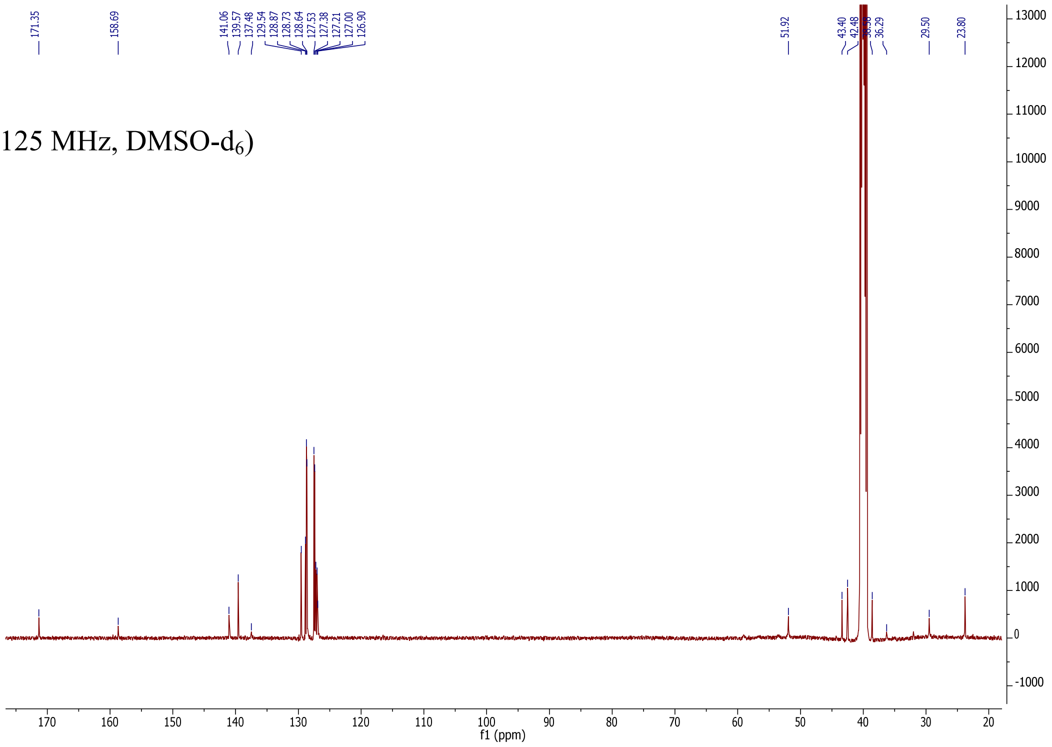

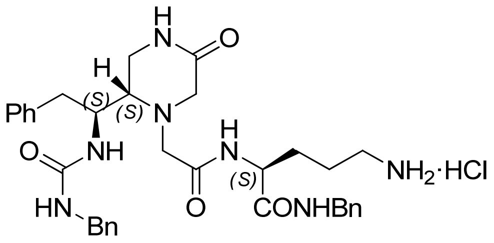

(*S*)-12a

(*S*)-12a (500 MHz, DMSO-d_6)

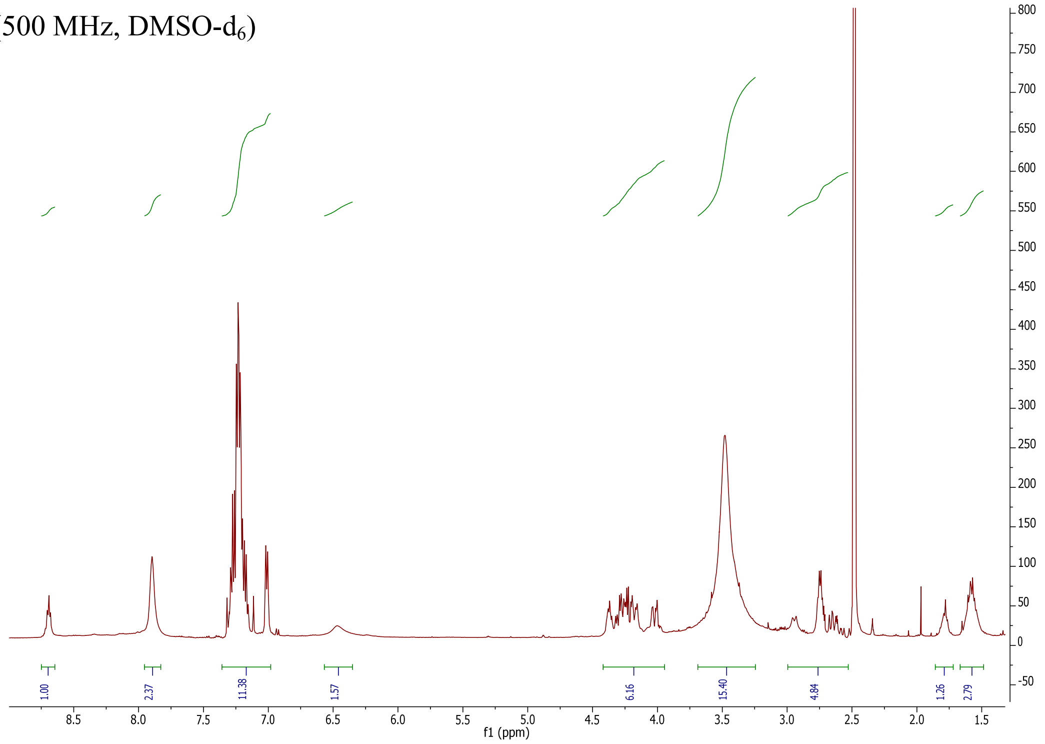

(*S*)-12a (125 MHz, DMSO-d_6)

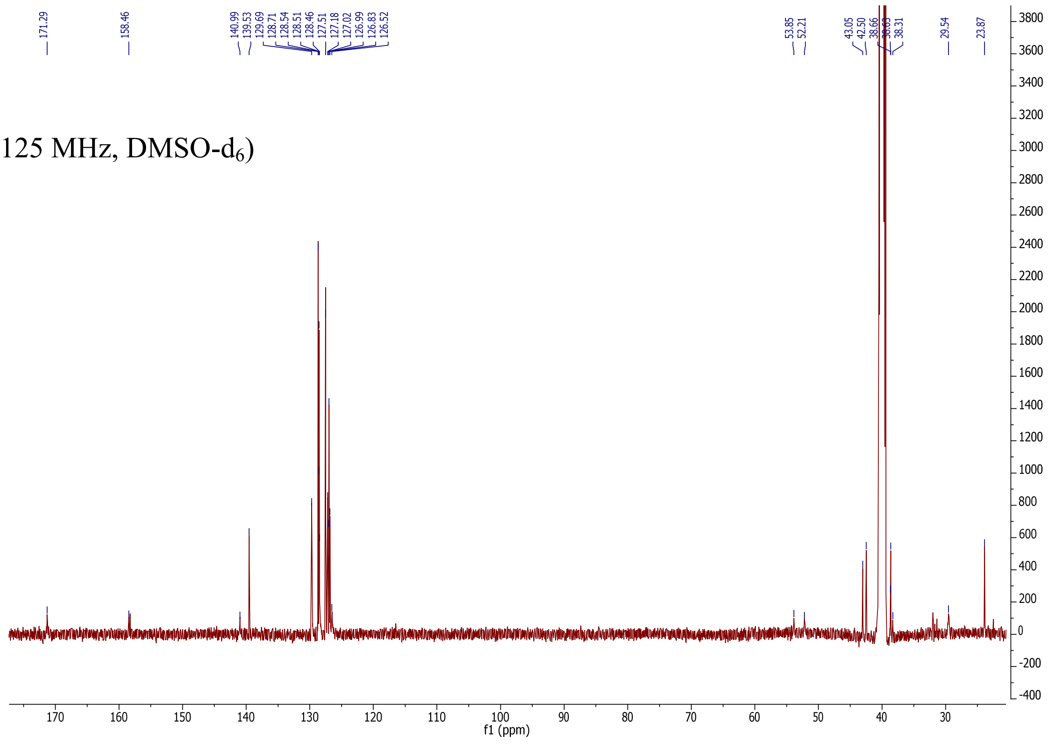

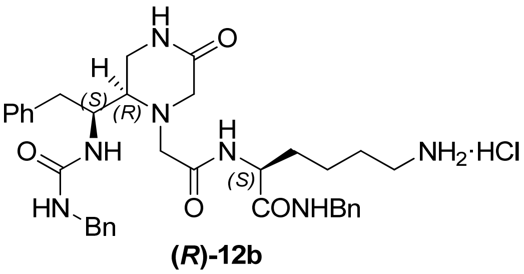

(*R*)-12b

(*R*)-12b (500 MHz, DMSO-d$_6$)

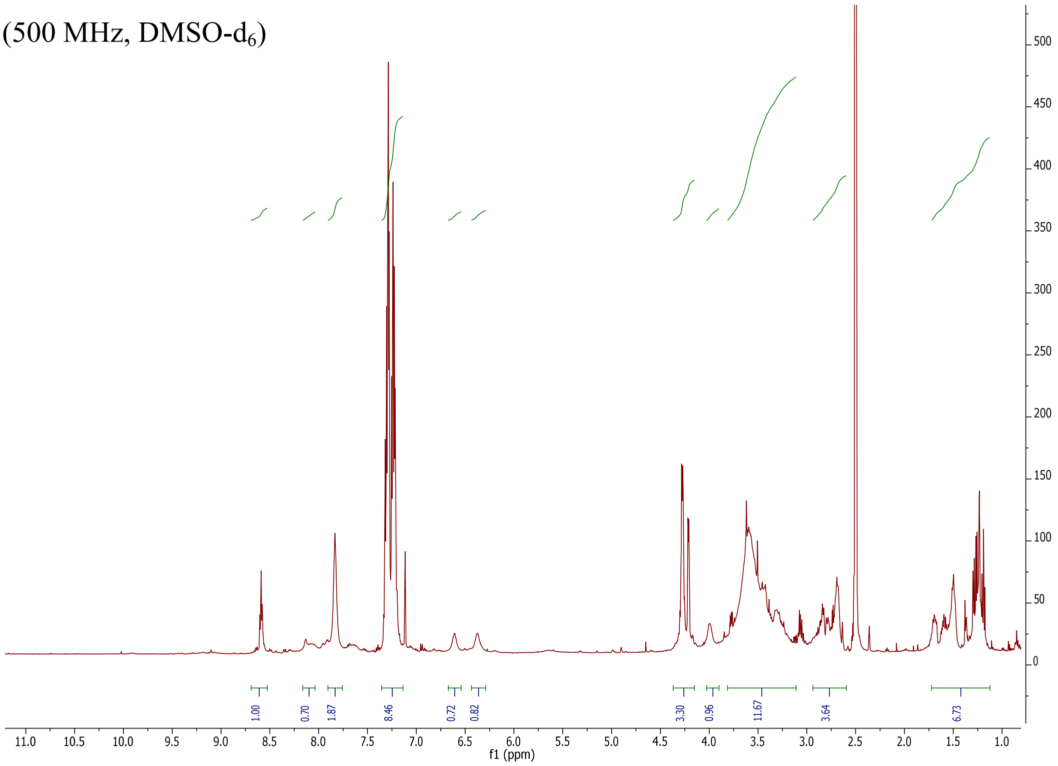

(*R*)-12b (125 MHz, DMSO-d$_6$)

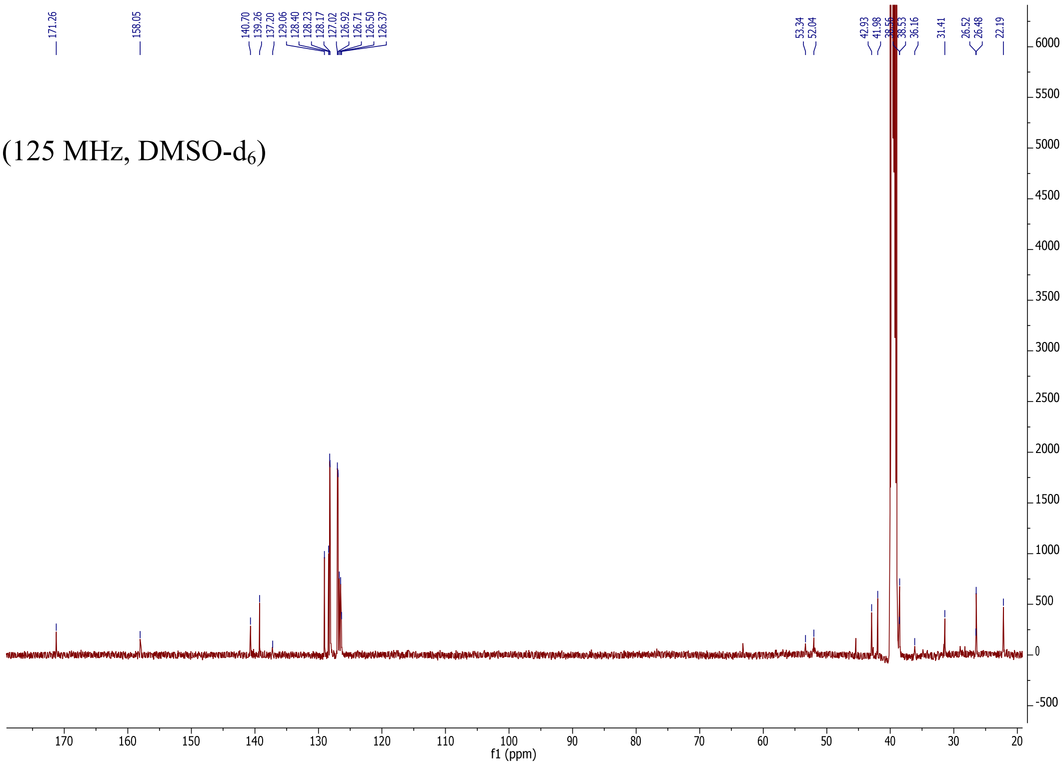

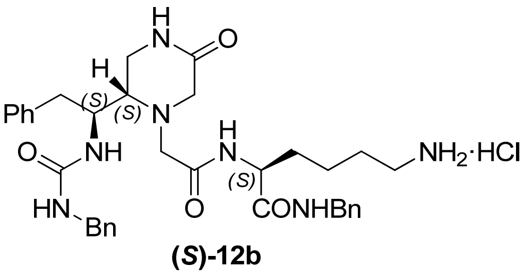

(*S*)-12b

(*S*)-12b (500 MHz, DMSO-d$_6$)

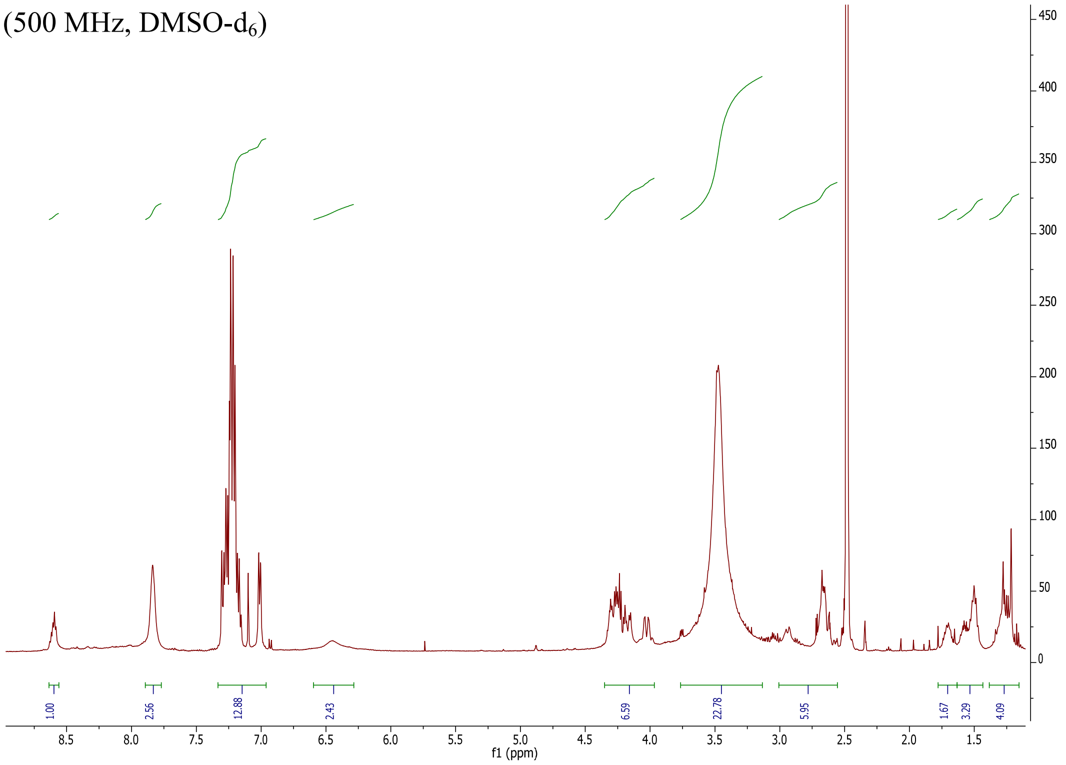

(*S*)-12b (125 MHz, DMSO-d$_6$)

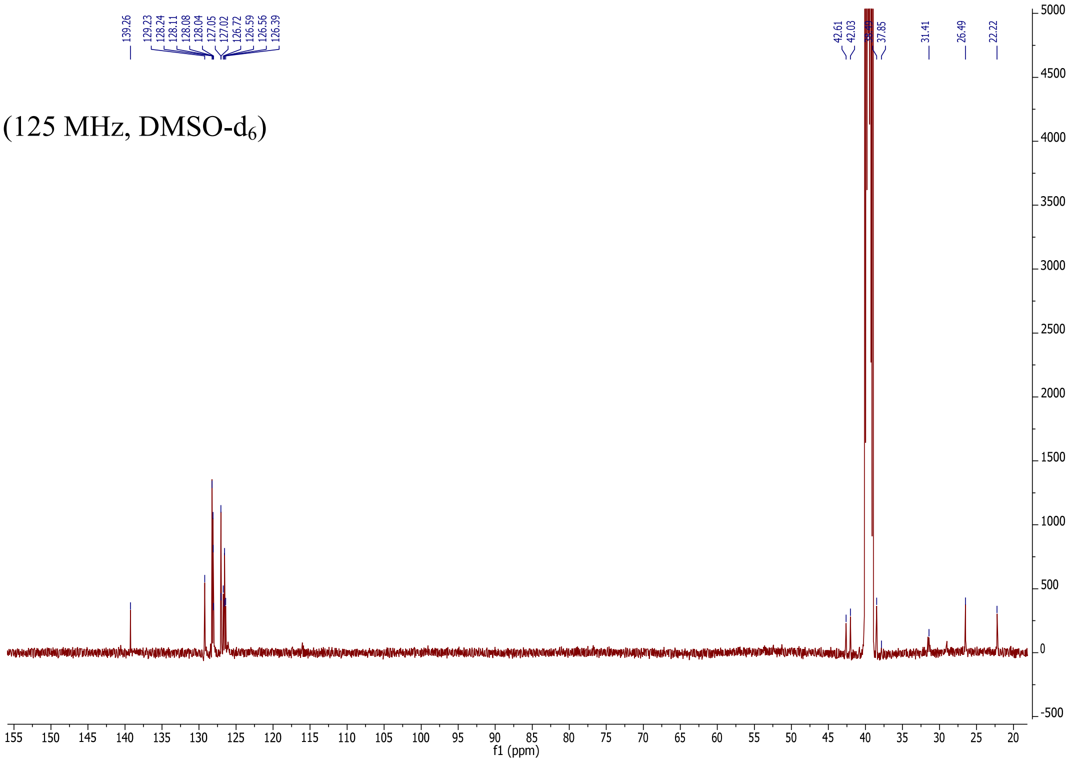

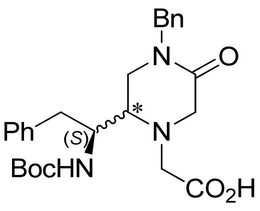

14

14 (500 MHz, $CDCl_3$)

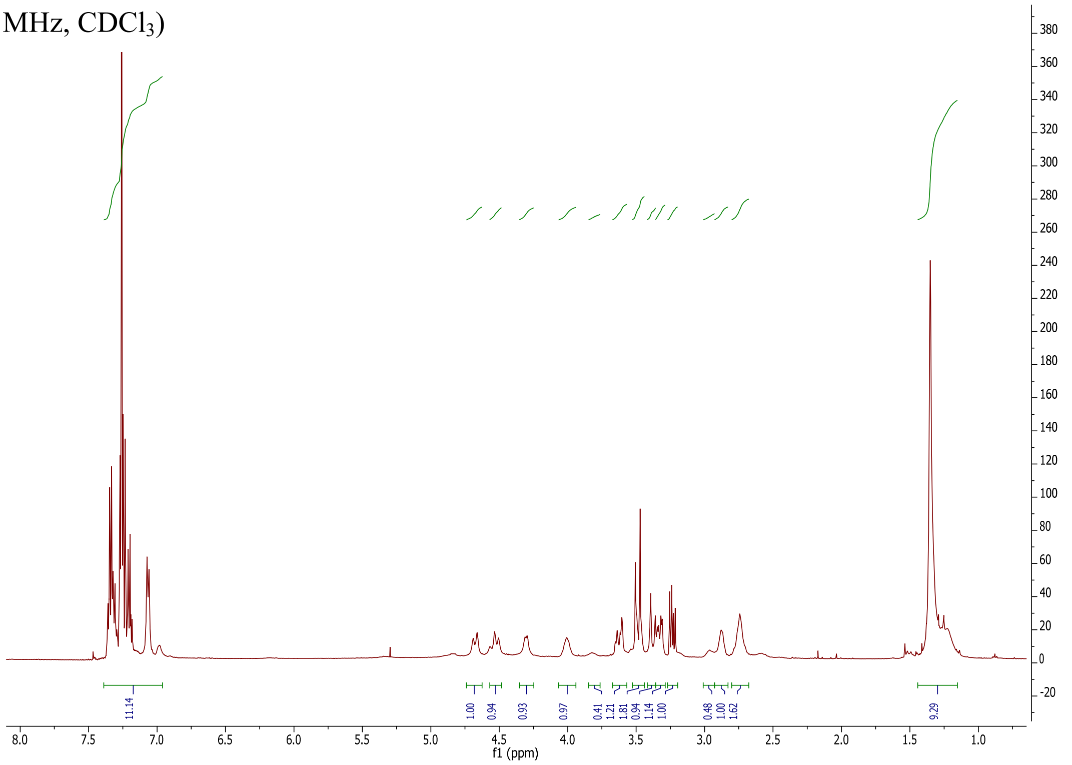

14 (125 MHz, $CDCl_3$)

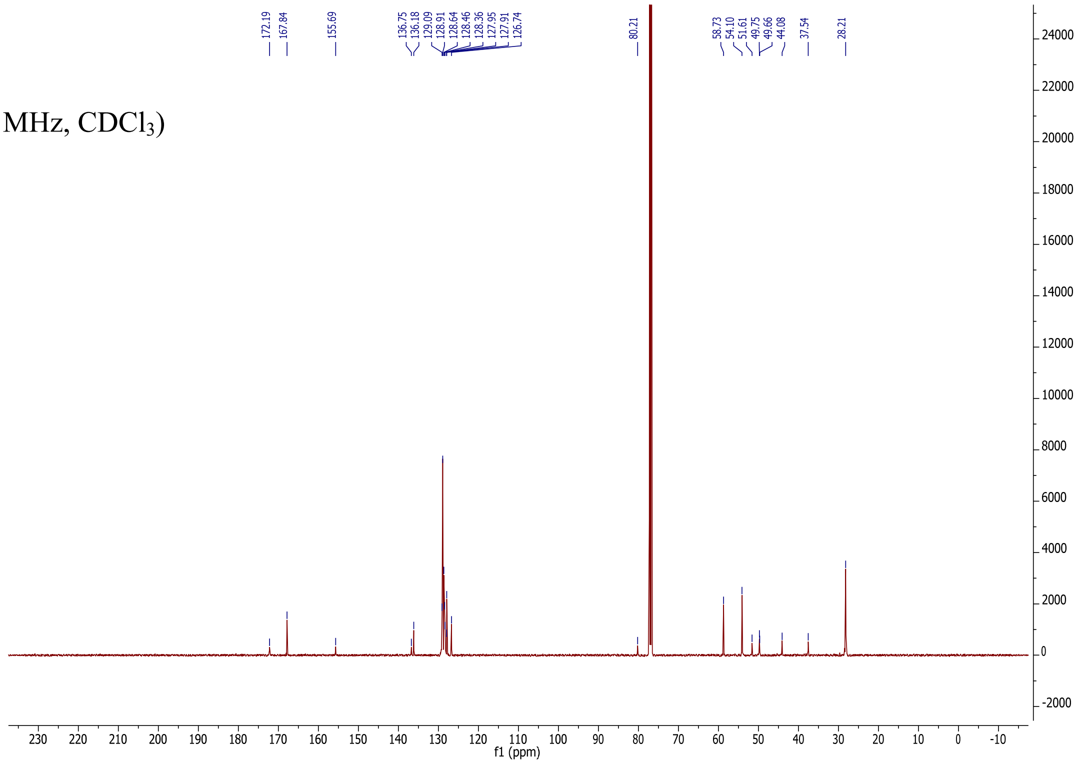

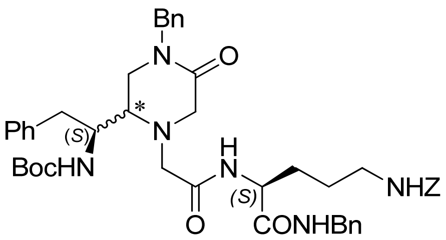

15a

15a (500 MHz, $CDCl_3$)

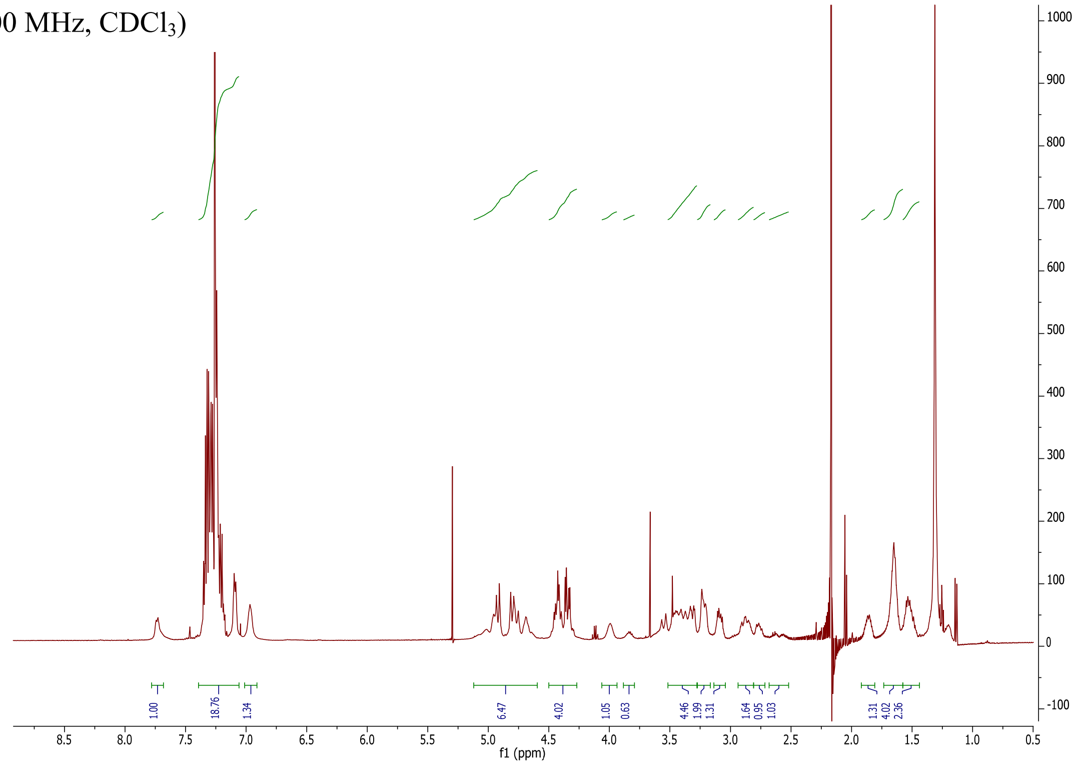

15a (125 MHz, $CDCl_3$)

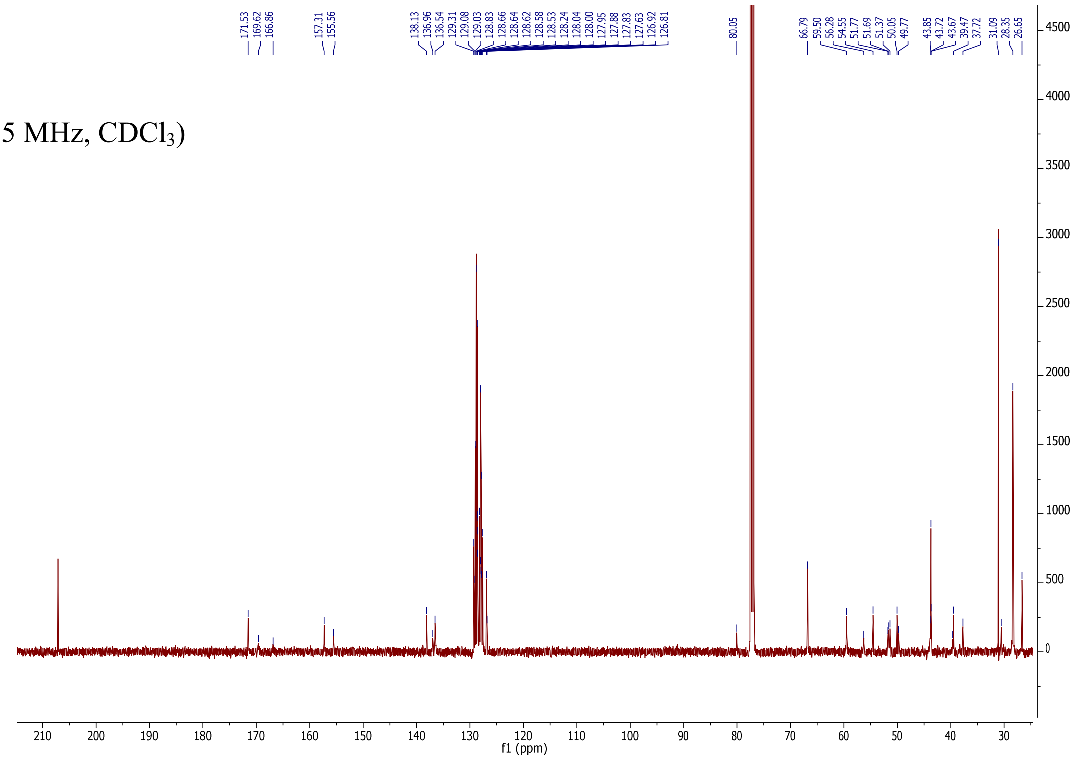

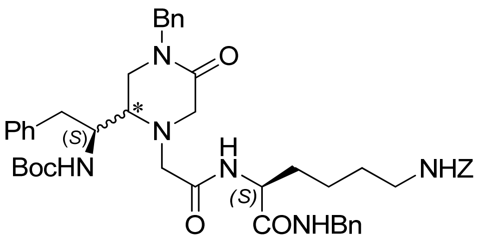

15b

15b (500 MHz, $CDCl_3$)

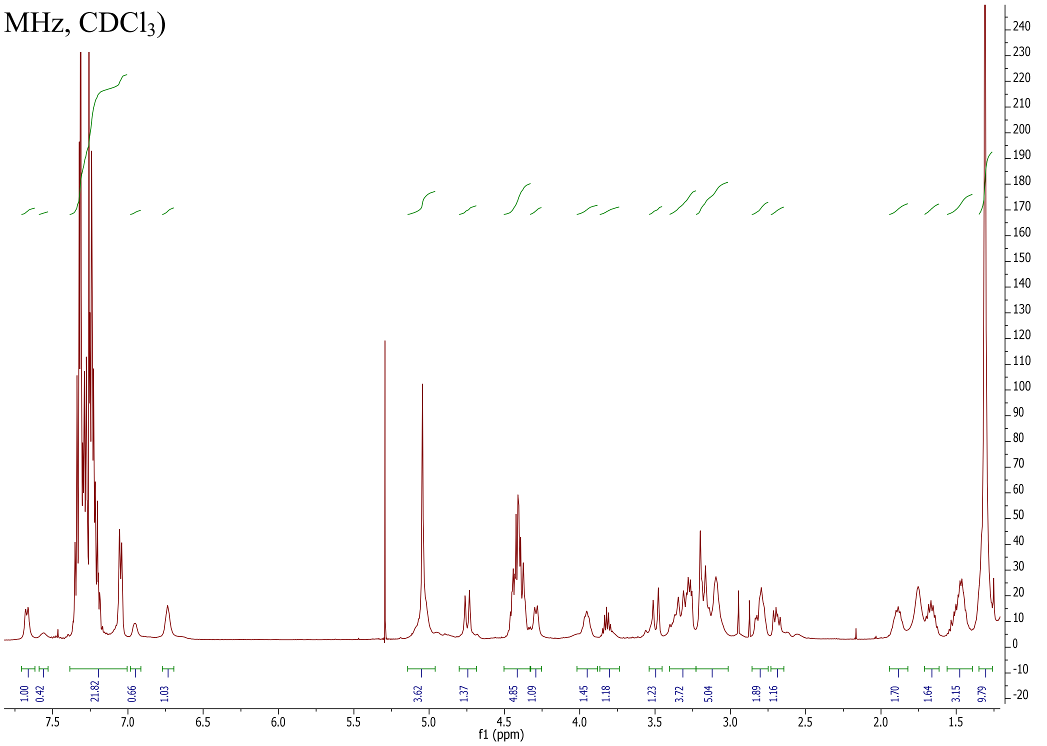

15b (125 MHz, $CDCl_3$)

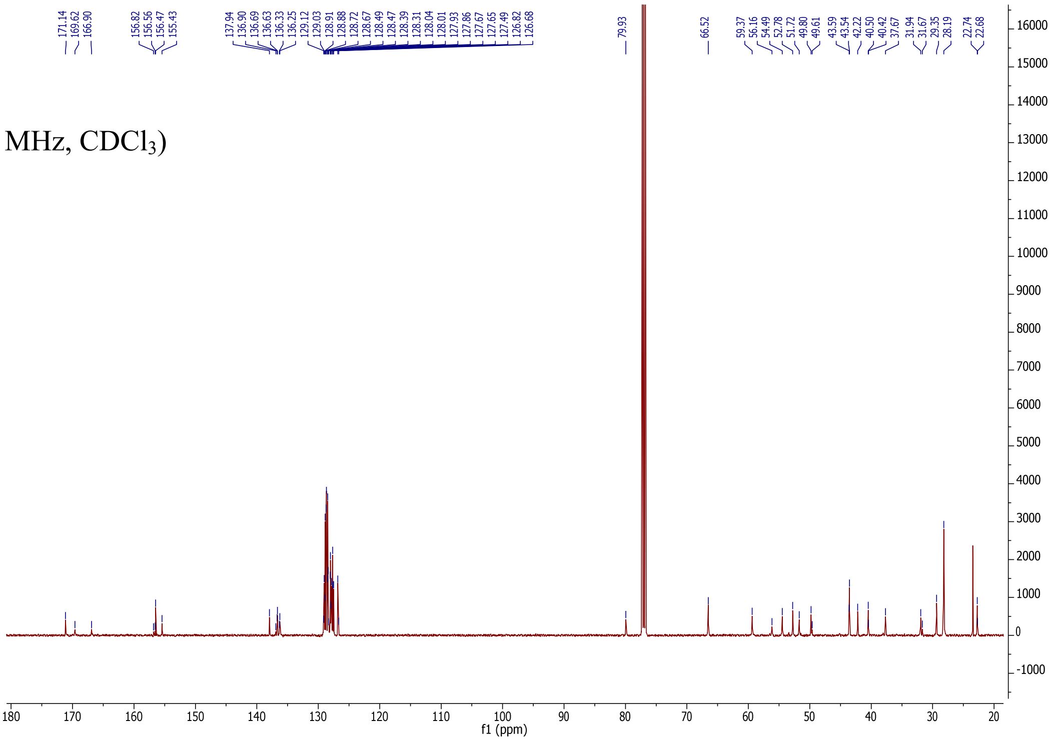

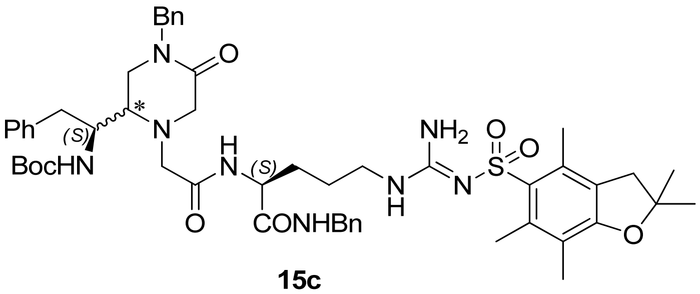

15c

15c (500 MHz, $CDCl_3$)

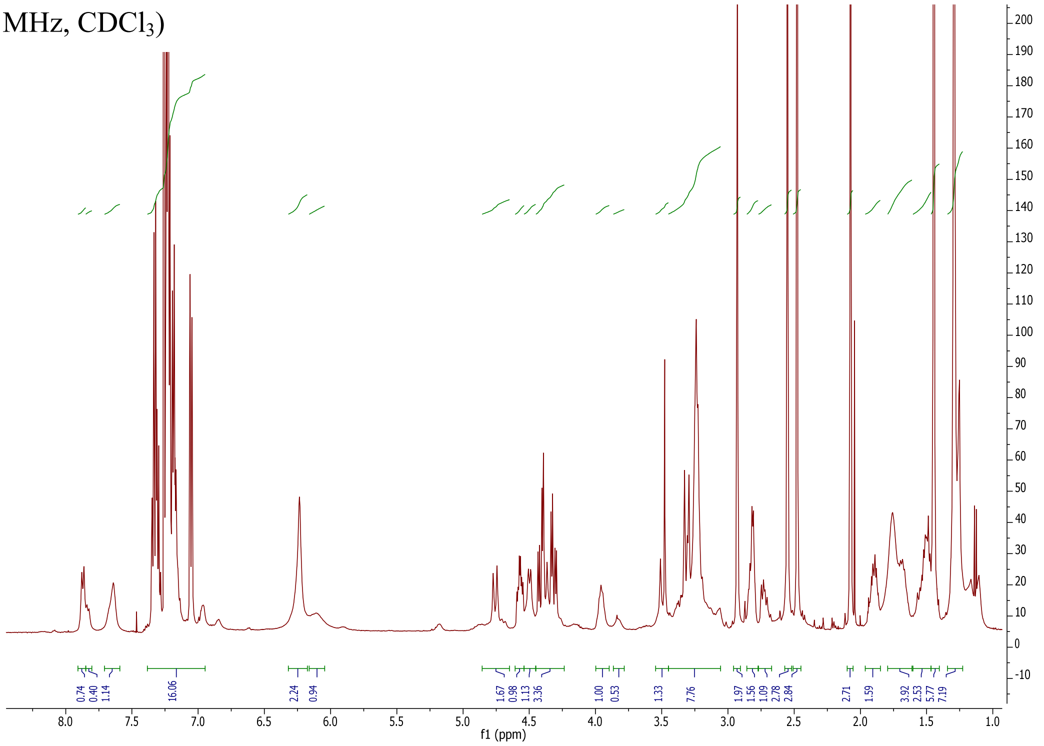

15c (125 MHz, $CDCl_3$)

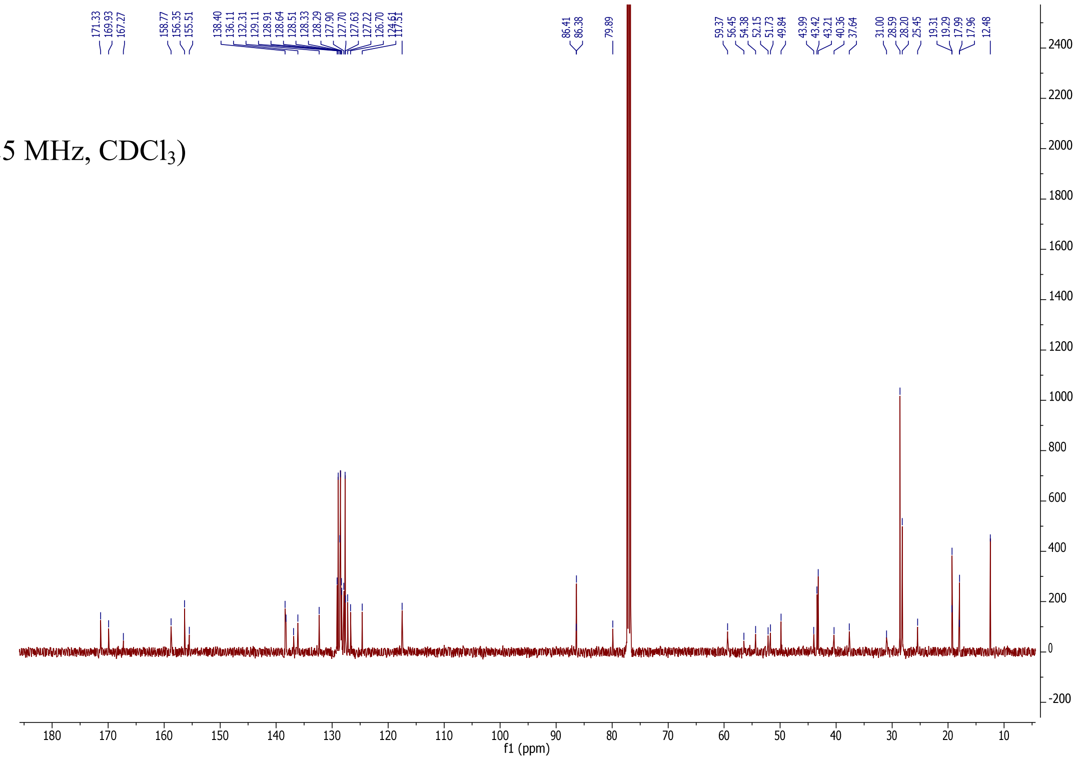

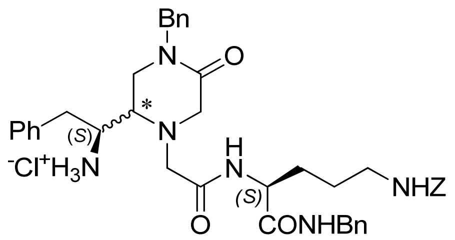

16a

16a (500 MHz, DMSO-d$_6$)

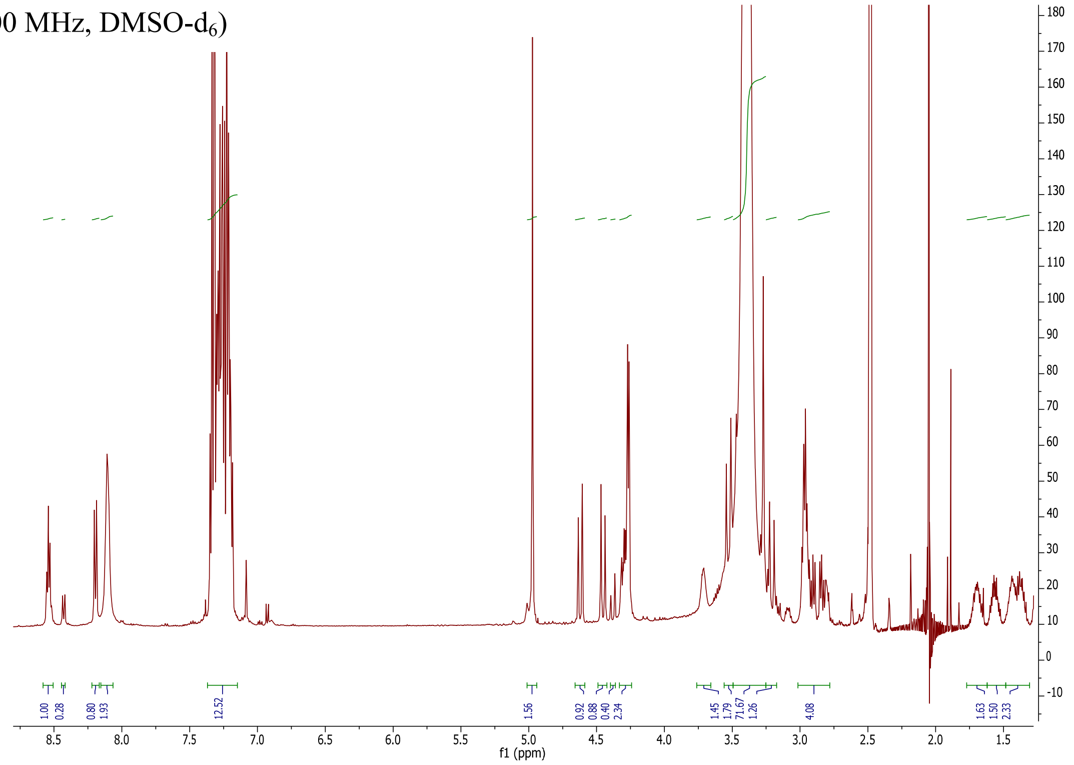

16a (125 MHz, DMSO-d$_6$)

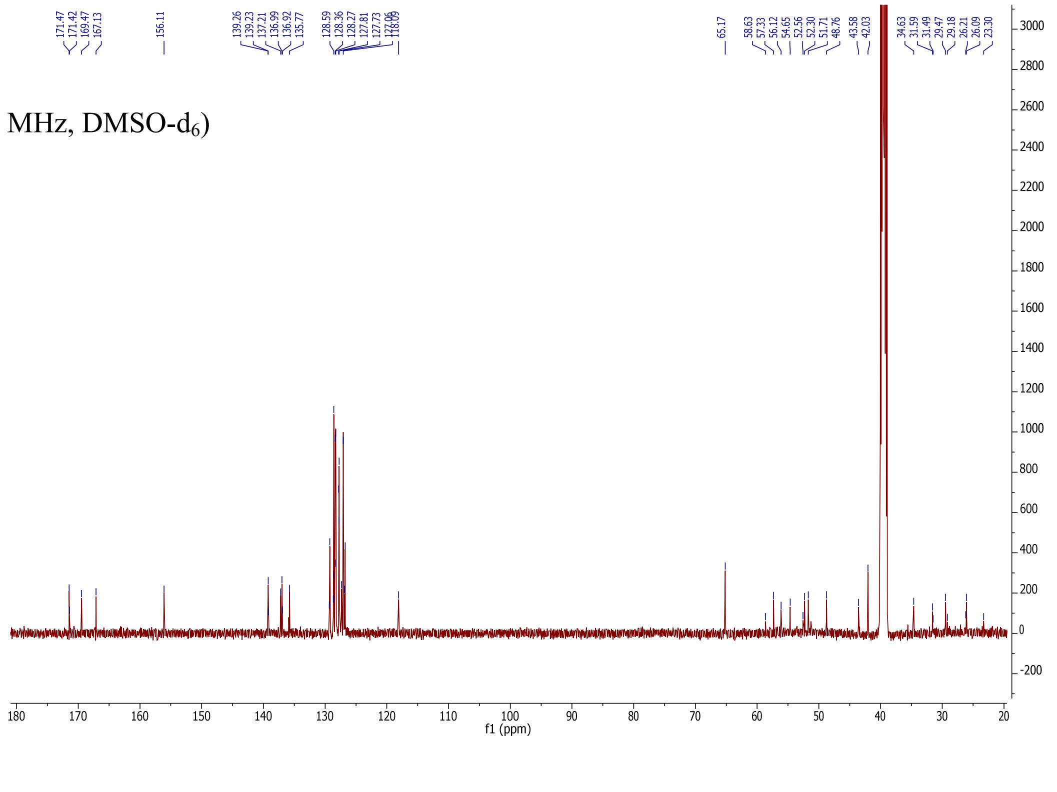

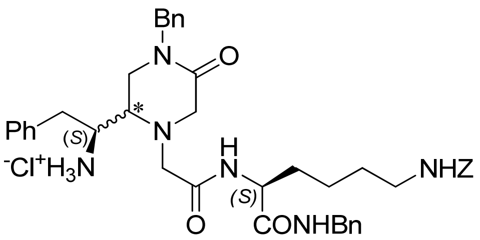

16b

16b (500 MHz, DMSO-d$_6$)

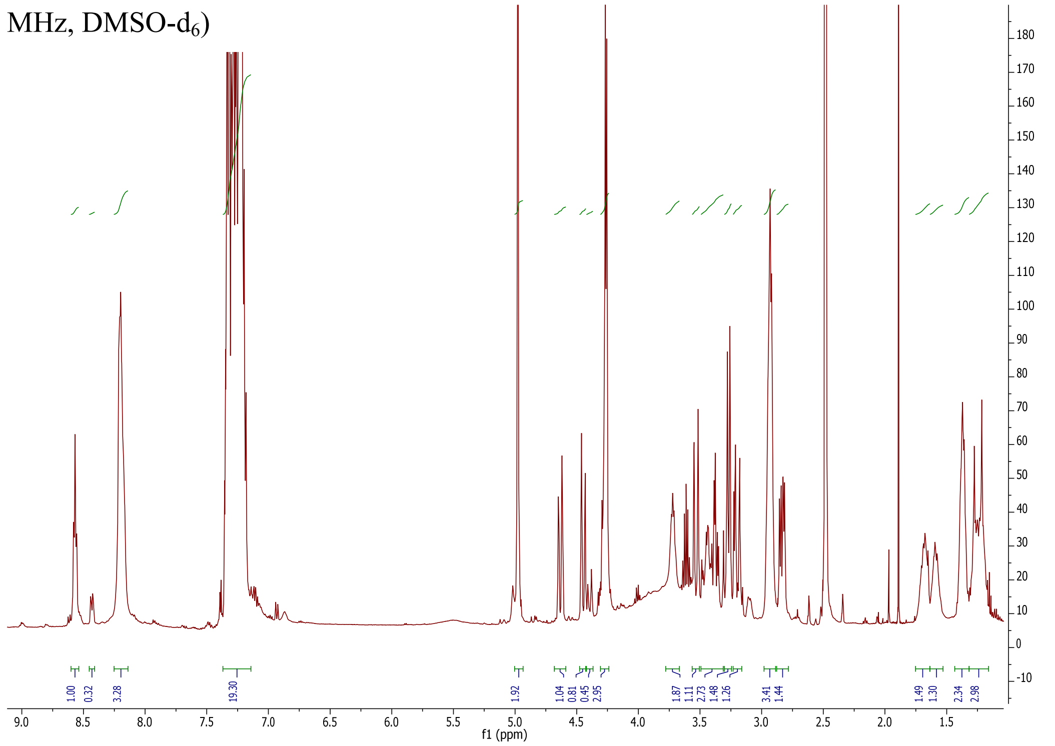

16b (125 MHz, DMSO-d$_6$)

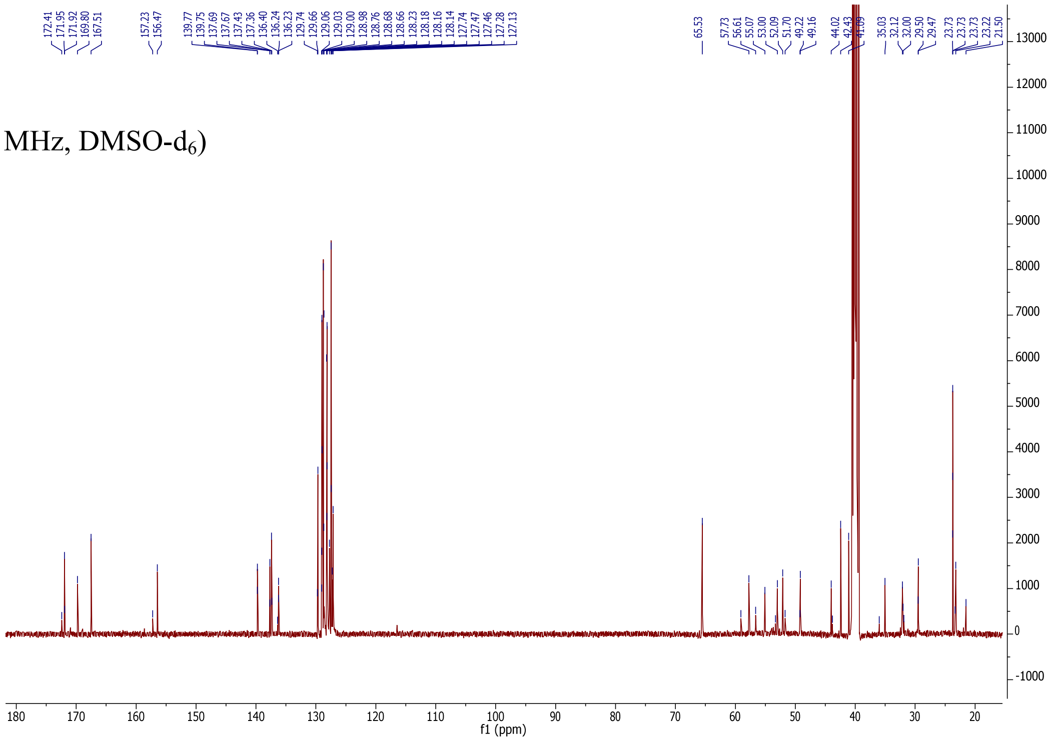

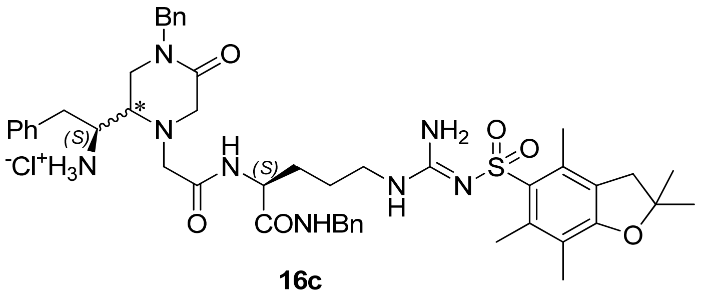

16c

16c (500 MHz, DMSO-d$_6$)

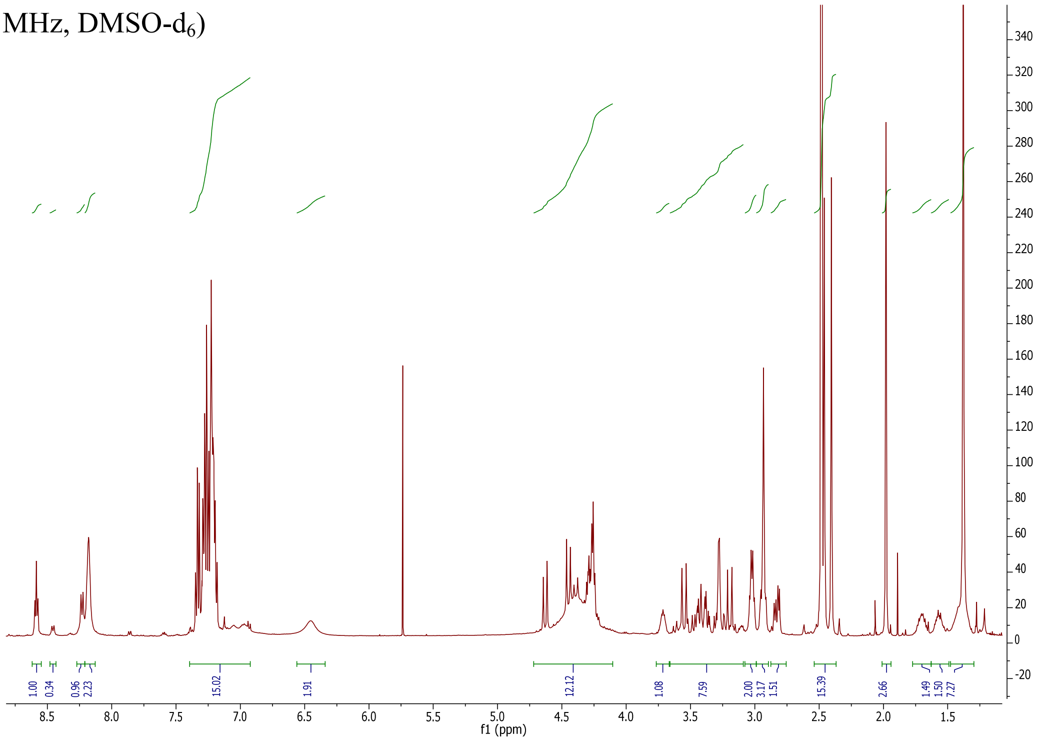

16cc (125 MHz, DMSO-d$_6$)

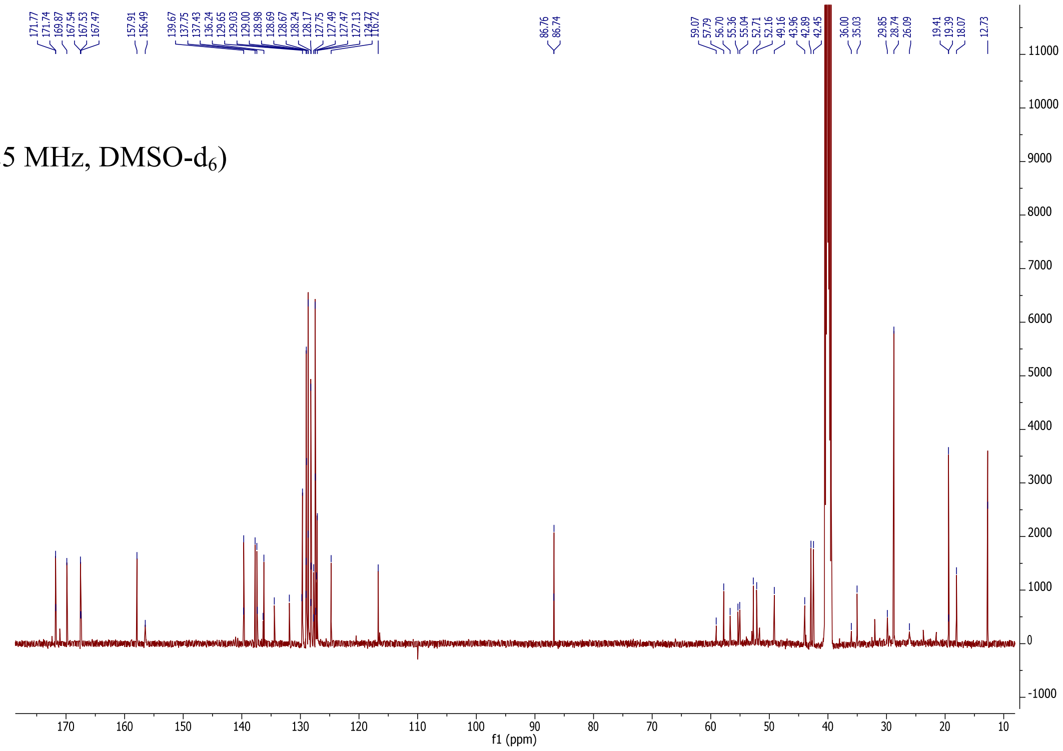

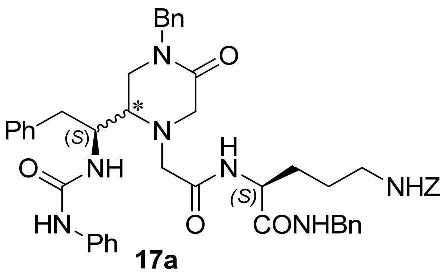

17a

17a (500 MHz, CDCl$_3$)

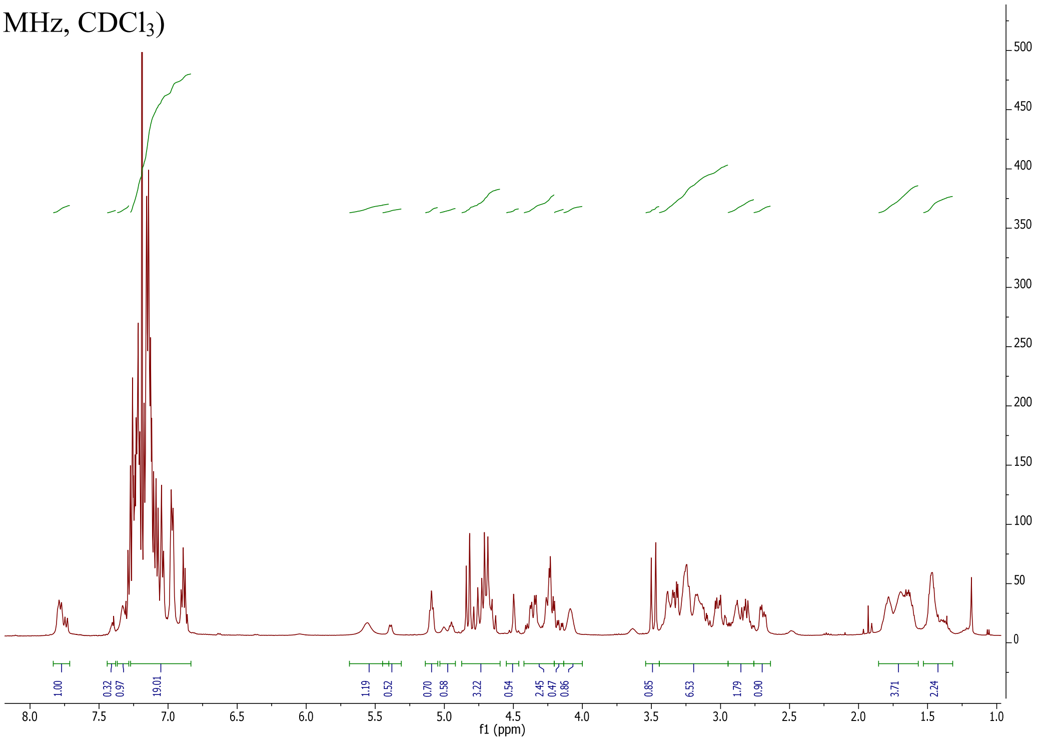

17a (125 MHz, CDCl$_3$)

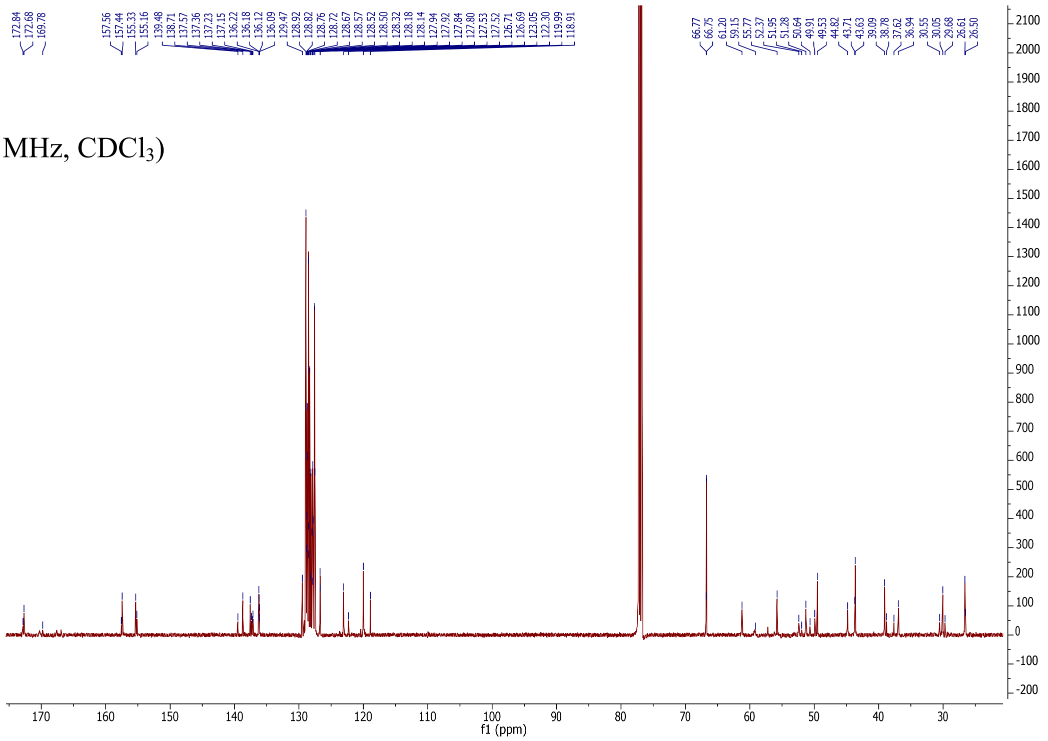

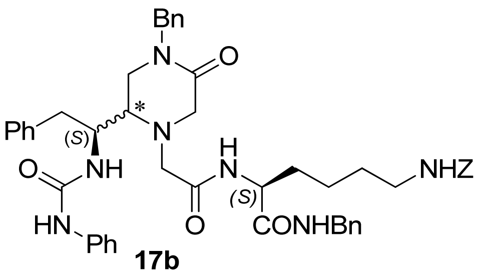

17b

17b (500 MHz, $CDCl_3$)

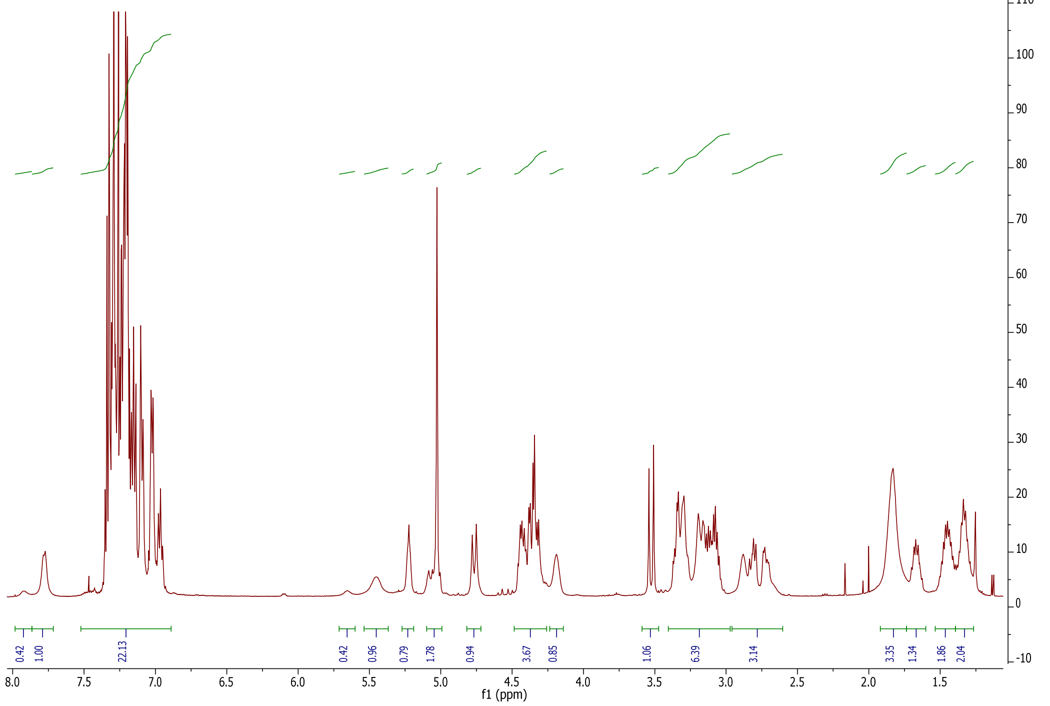

17b (125 MHz, $CDCl_3$)

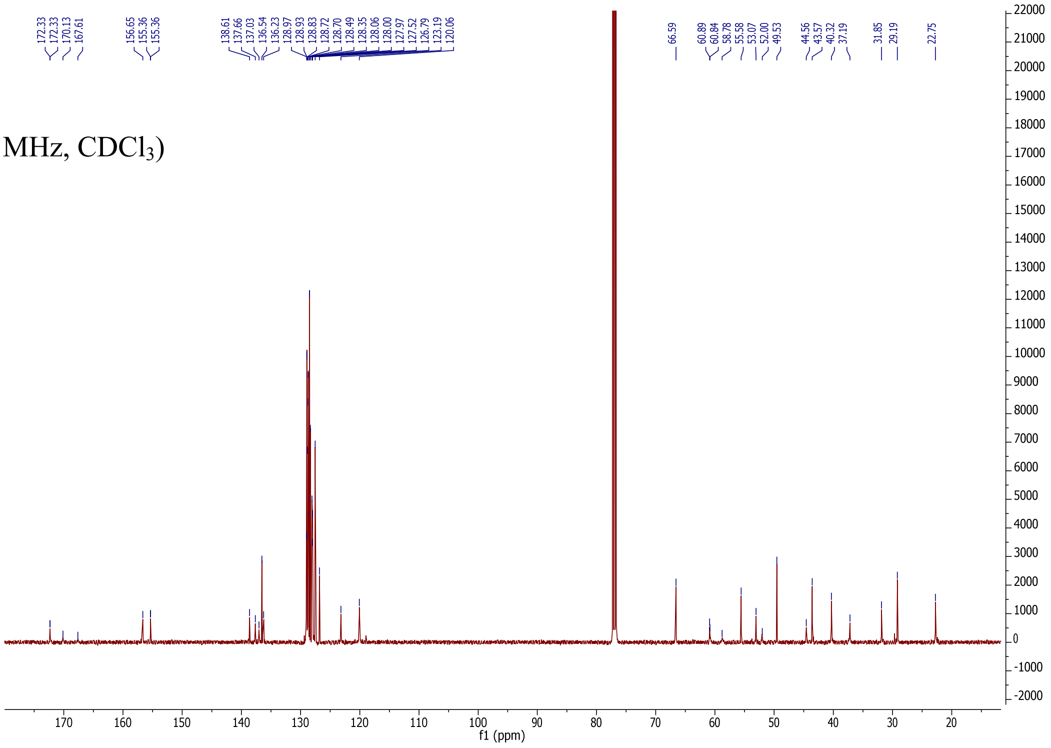

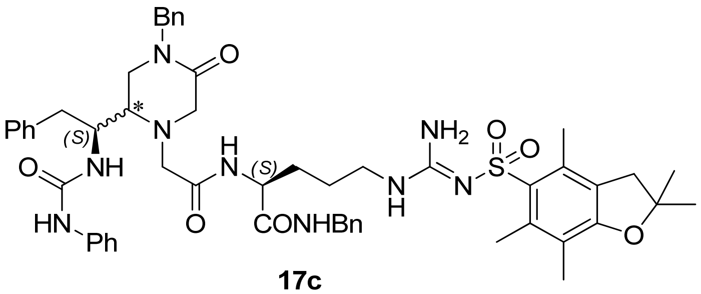

17c

17c (500 MHz, $CDCl_3$)

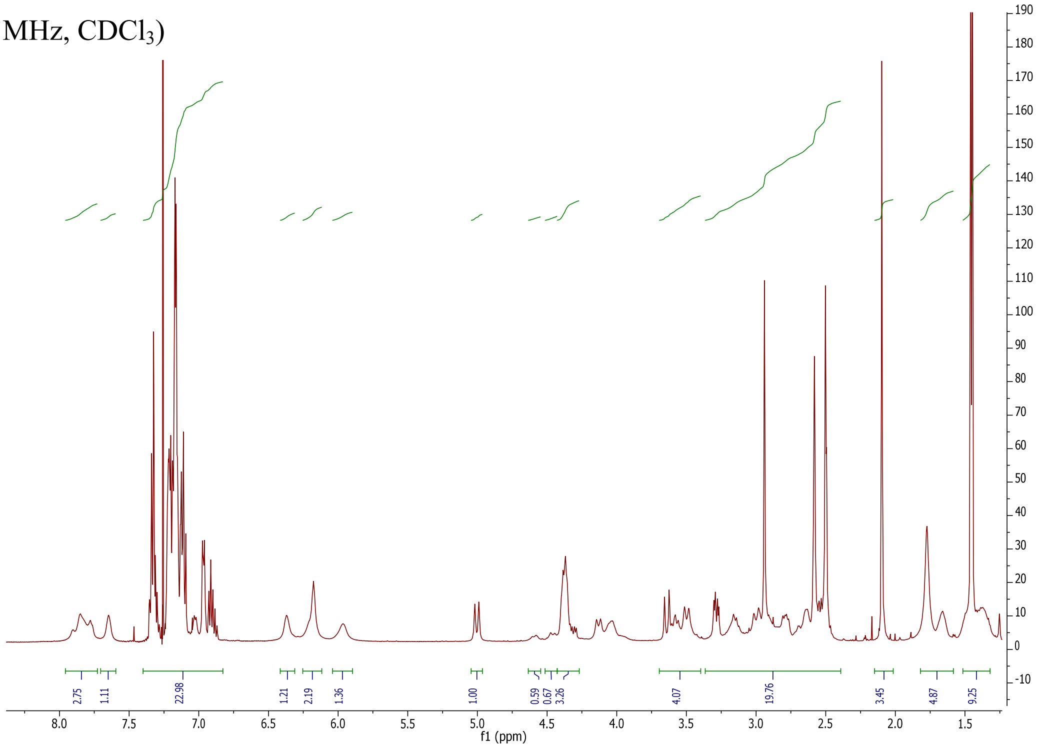

17c (125 MHz, $CDCl_3$)

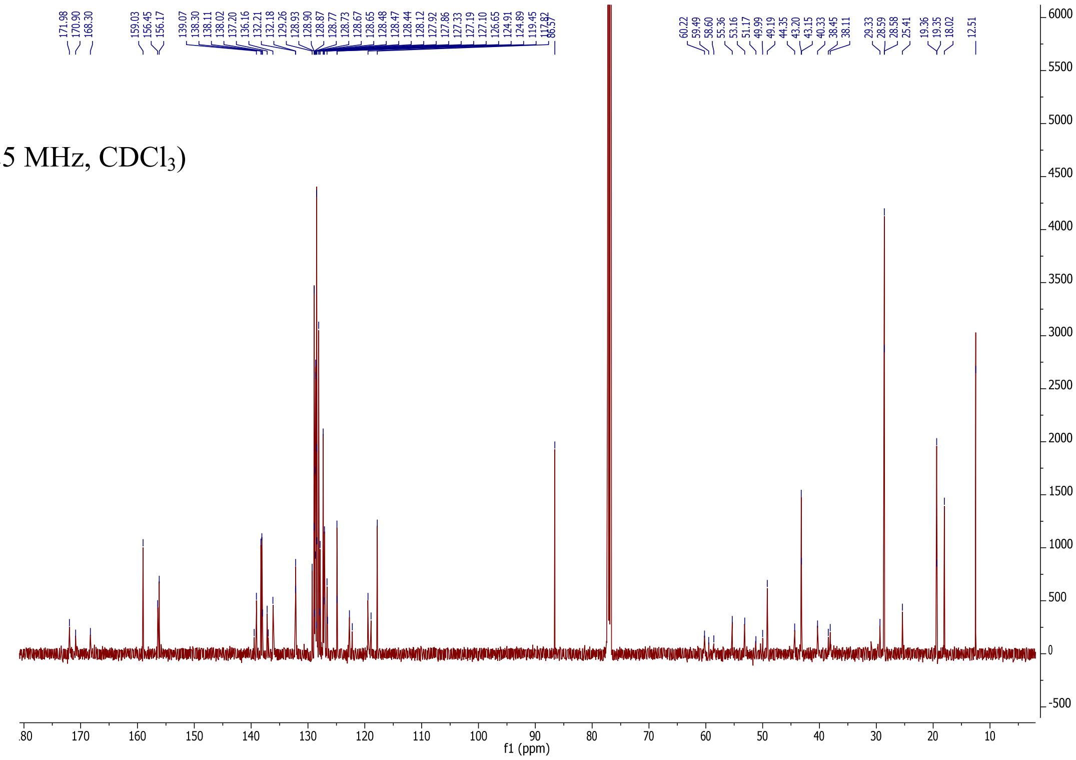

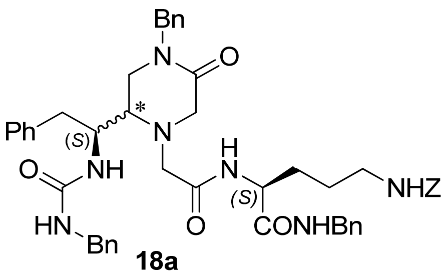

18a

18a (500 MHz, $CDCl_3$)

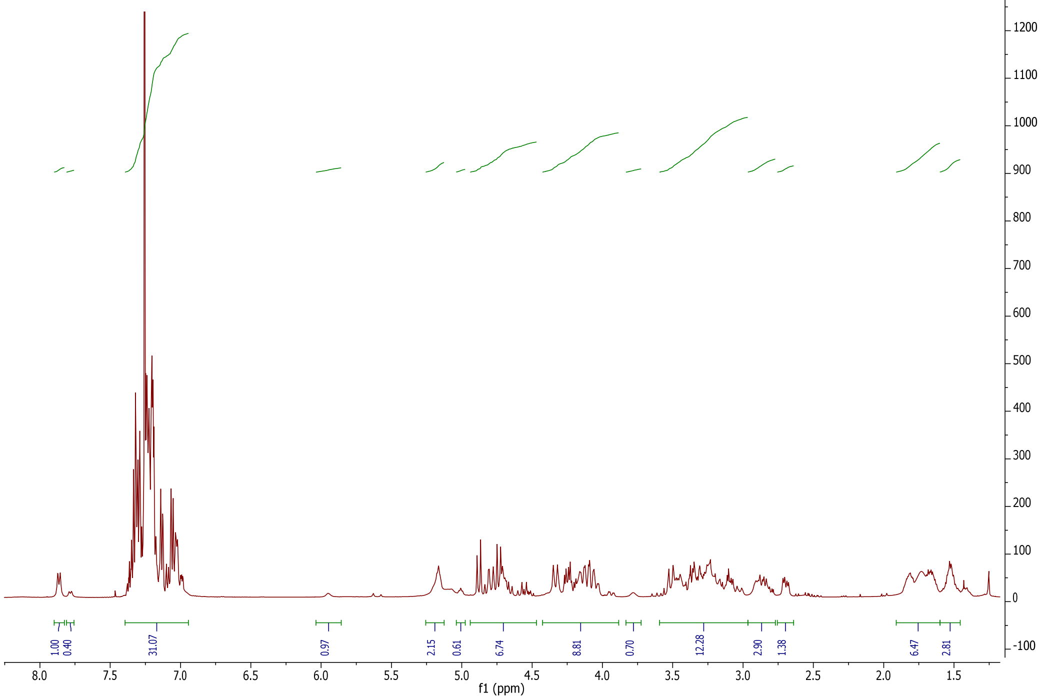

18a (125 MHz, $CDCl_3$)

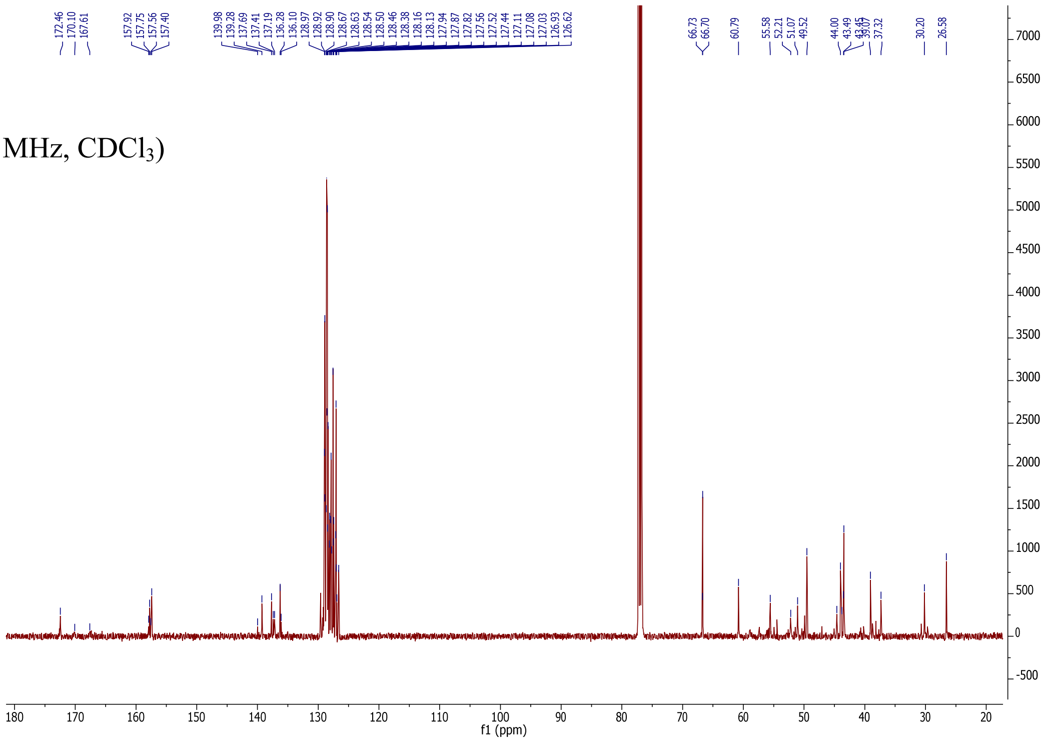

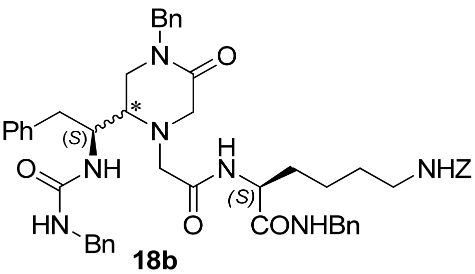

18b

(18b (500 MHz, $CDCl_3$)

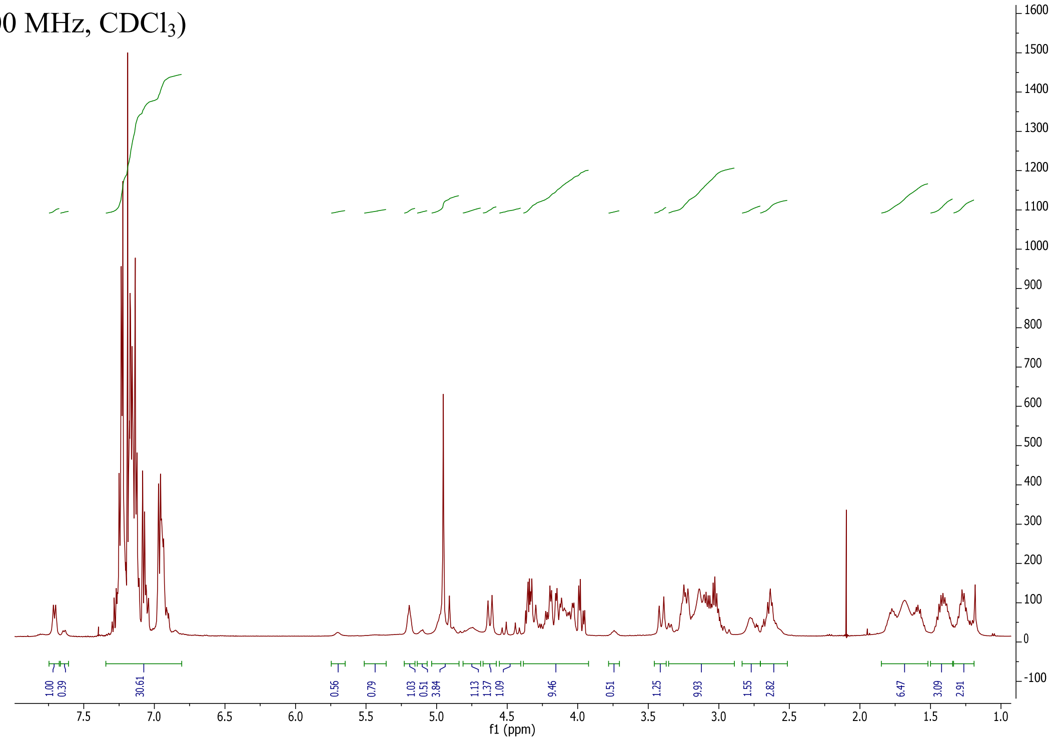

18b (125 MHz, $CDCl_3$)

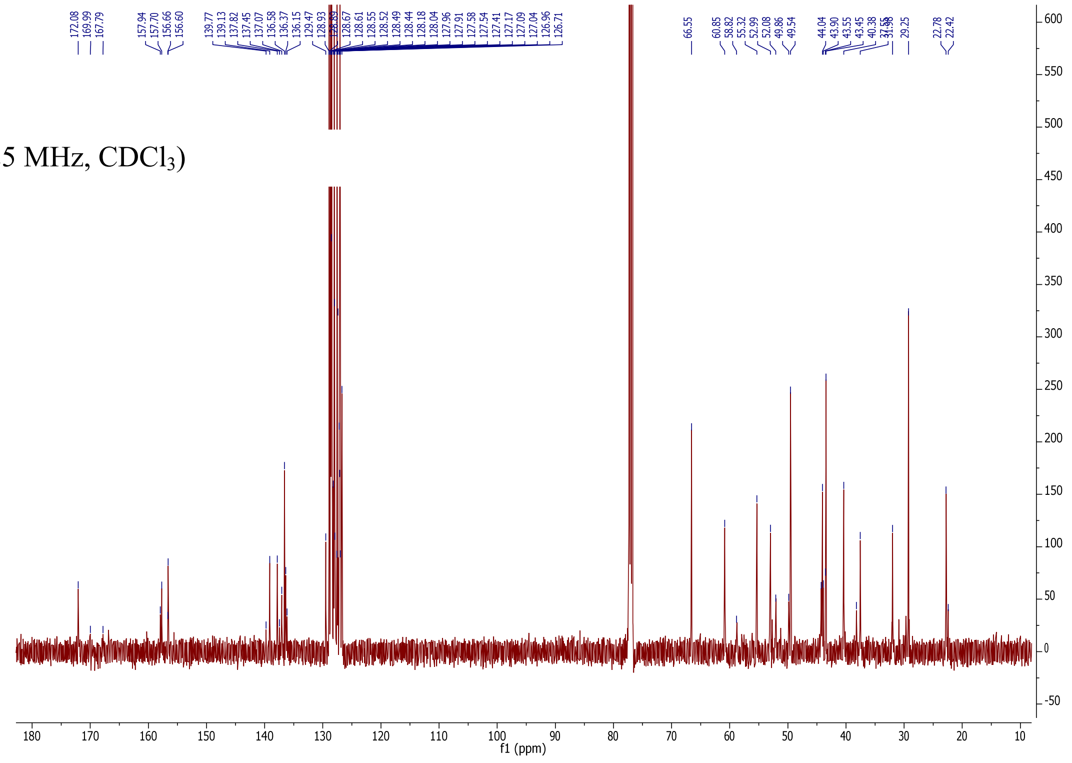

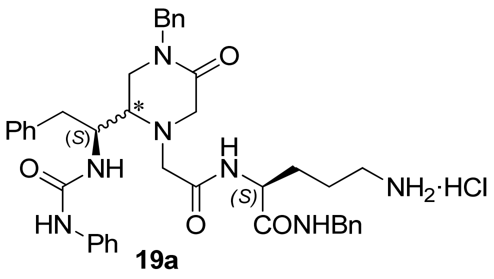

19a (500 MHz, DMSO-d$_6$)

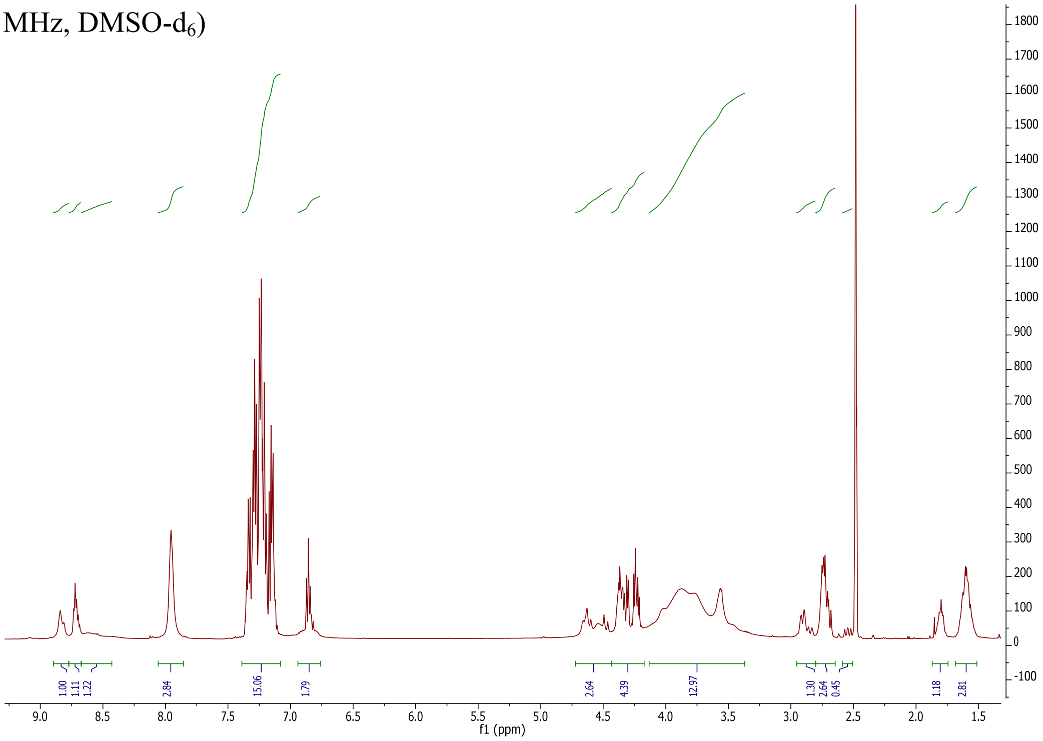

19a (125 MHz, DMSO-d$_6$)

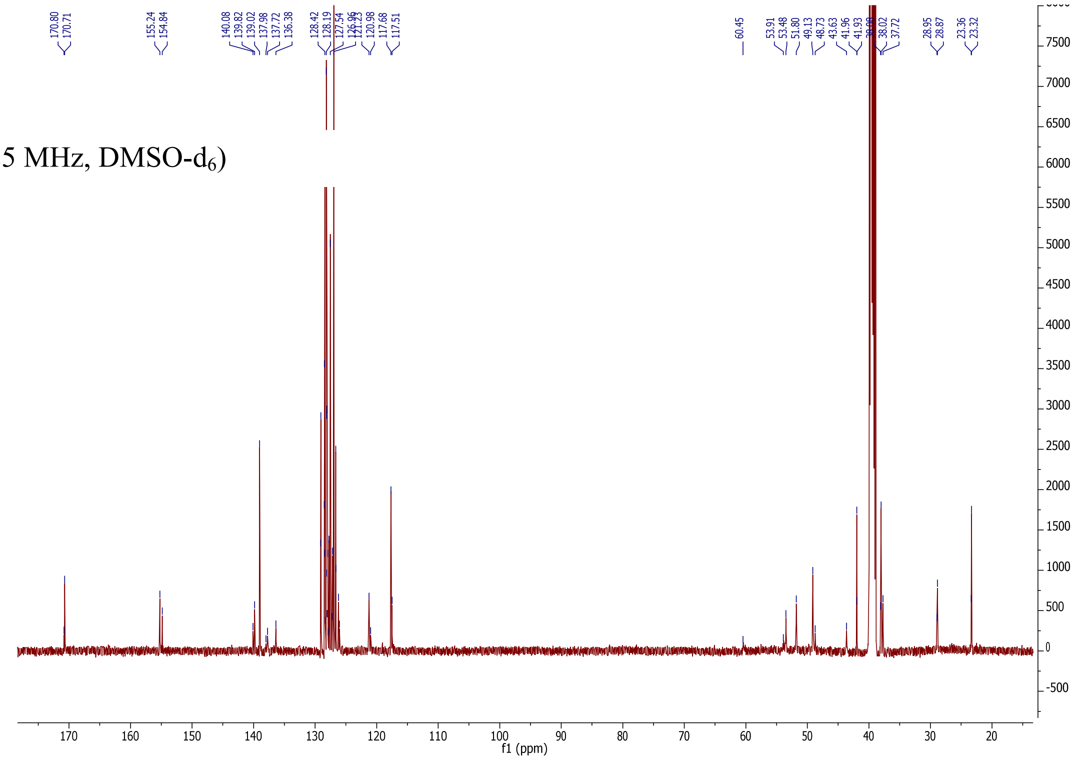

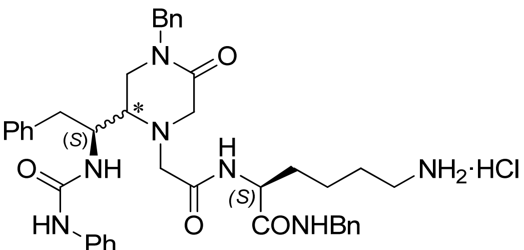

19b

19b (500 MHz, DMSO-d$_6$)

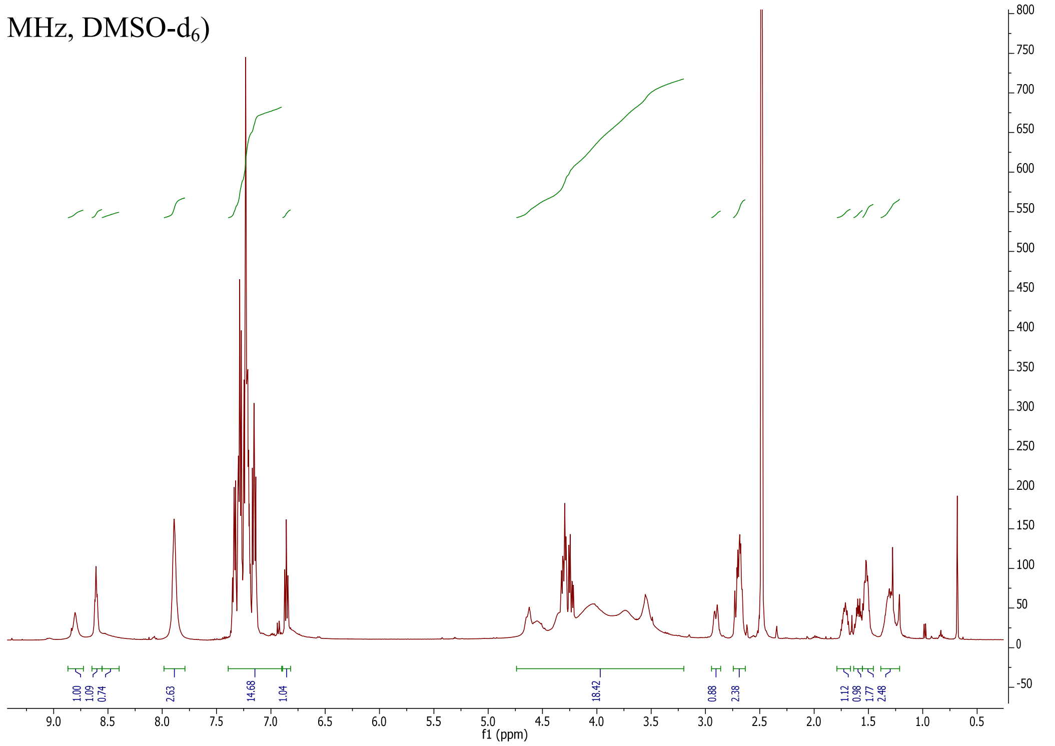

19b (125 MHz, DMSO-d$_6$)

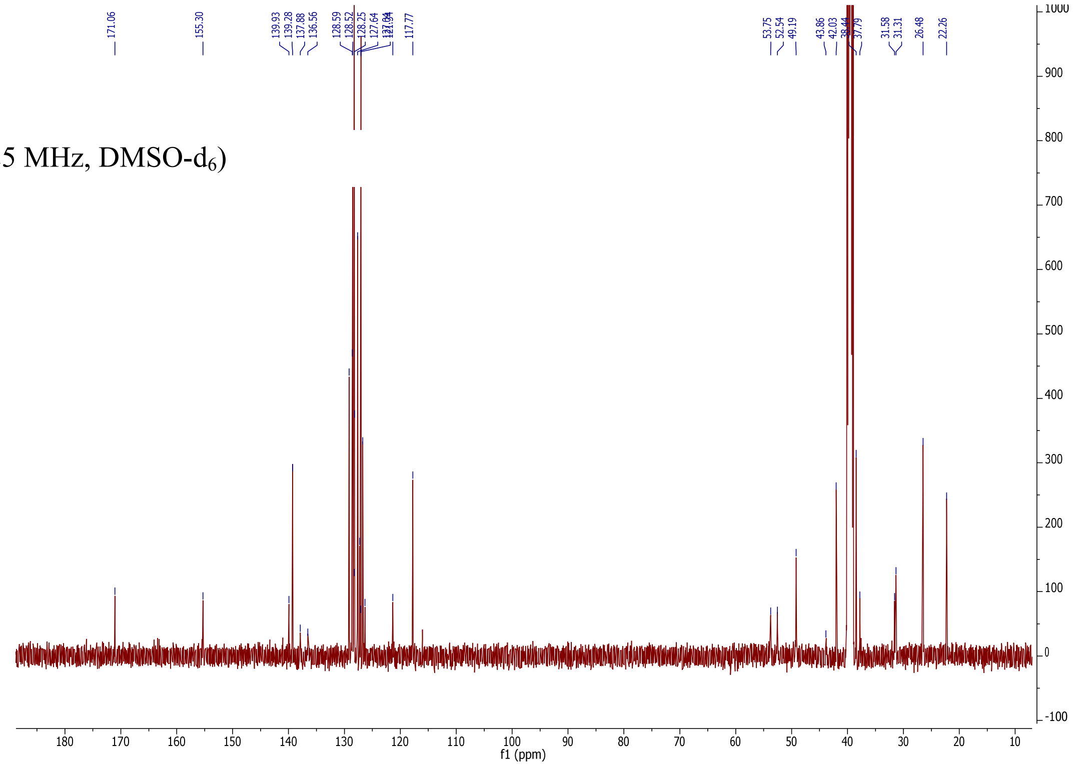

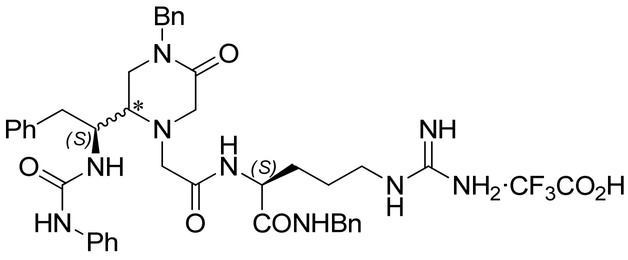

19c

19c (500 MHz, DMSO-d_6)

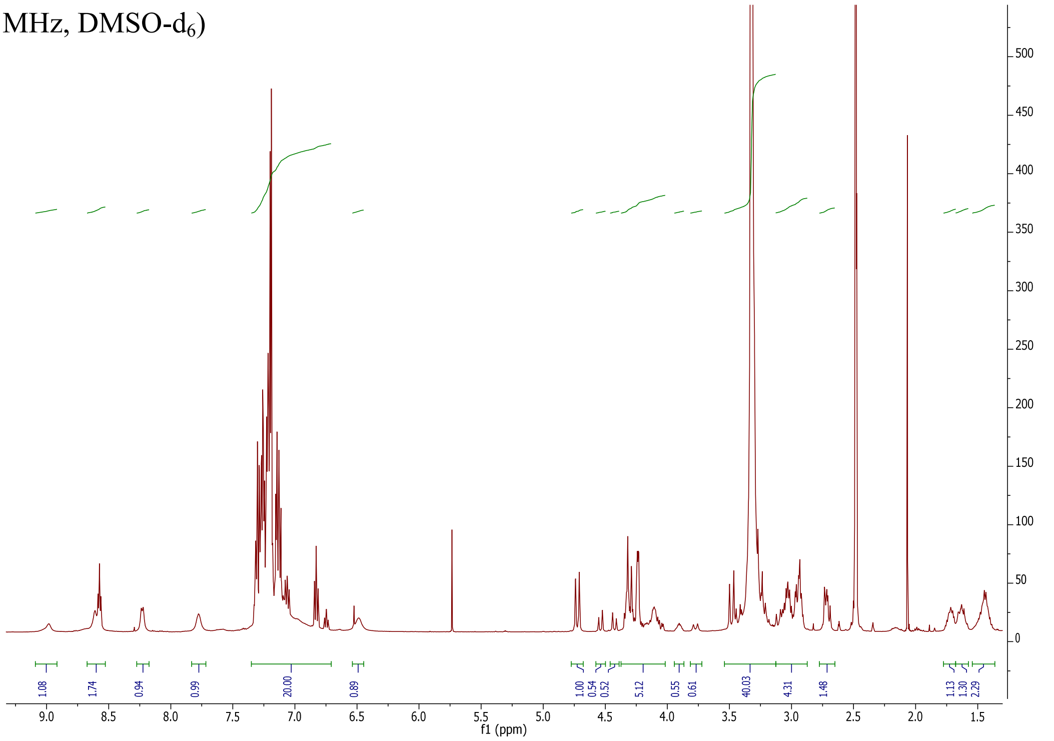

19c (125 MHz, DMSO-d_6)

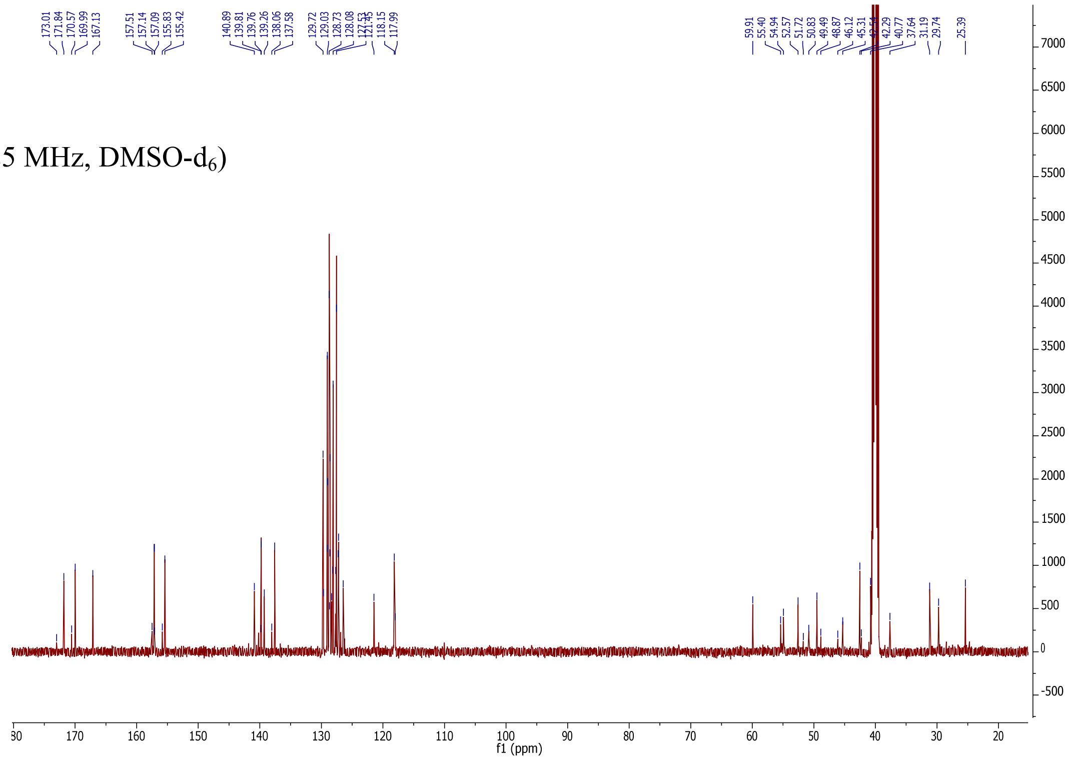

20a (500 MHz, DMSO-d$_6$)

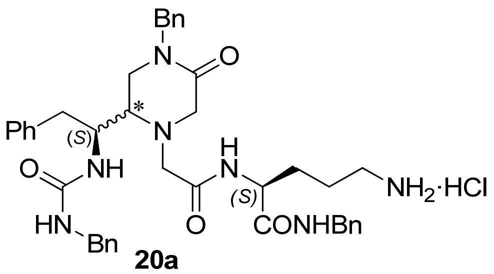

20a

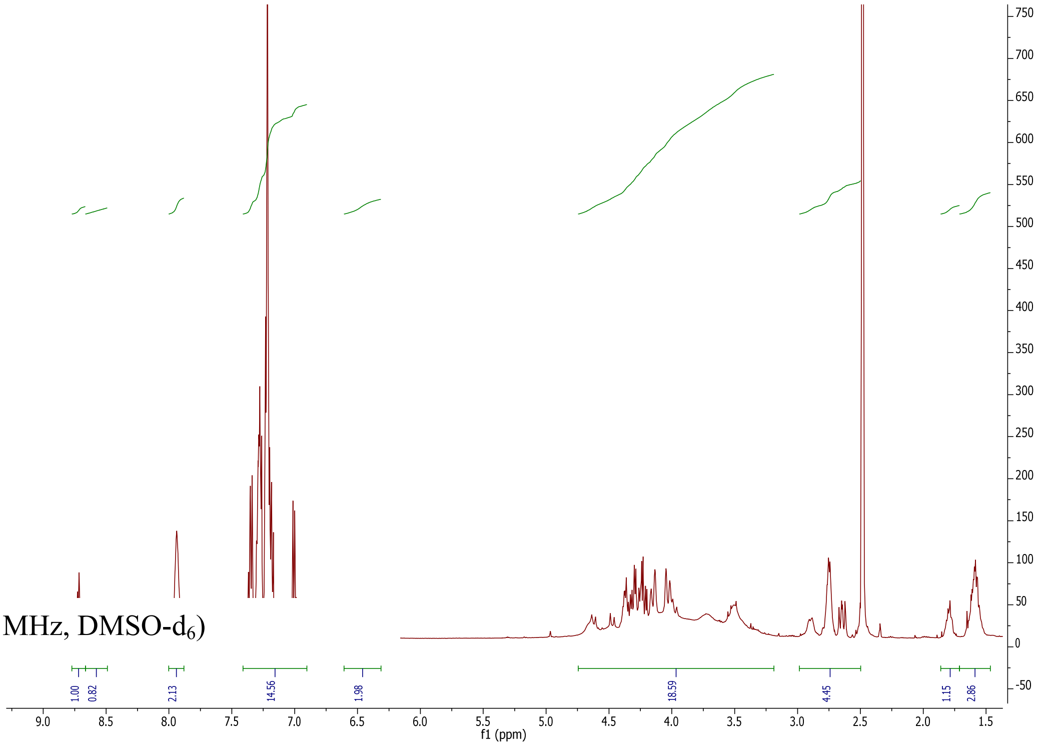

20a (125 MHz, DMSO-d$_6$)

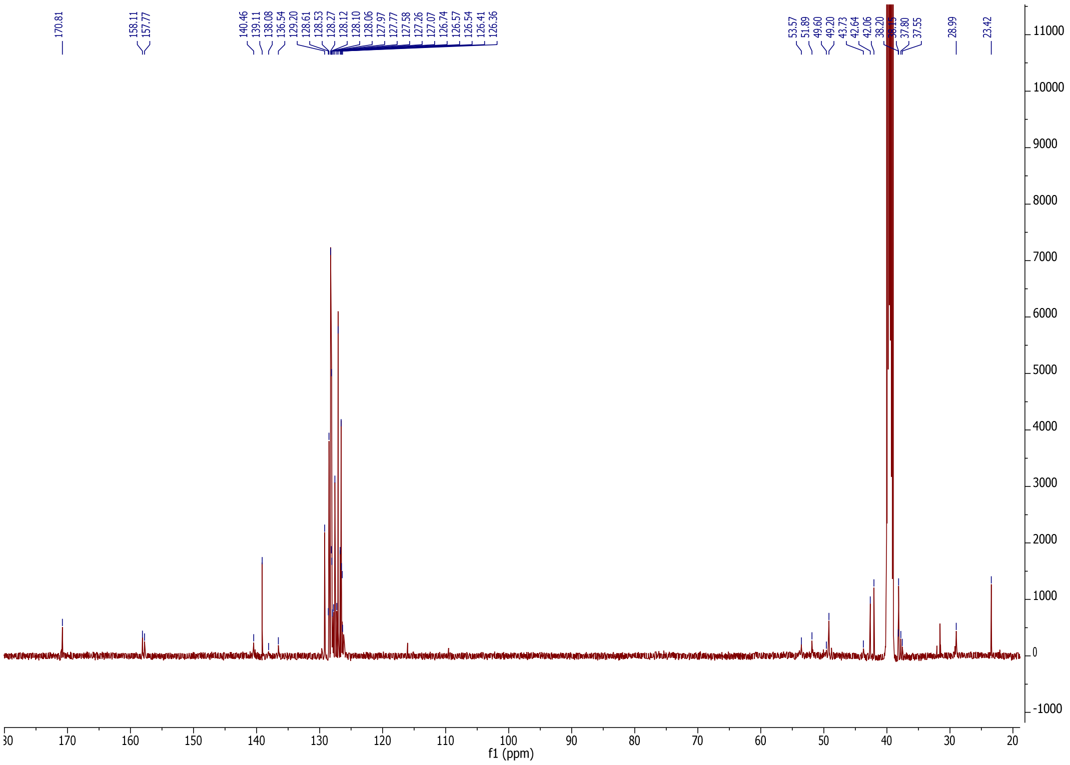

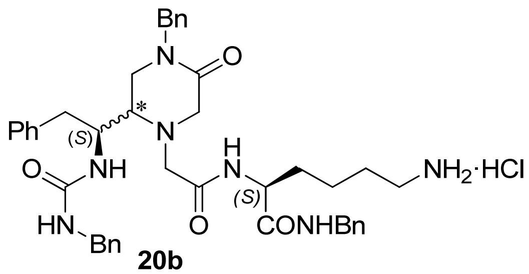

20b

20b (500 MHz, DMSO-d$_6$)

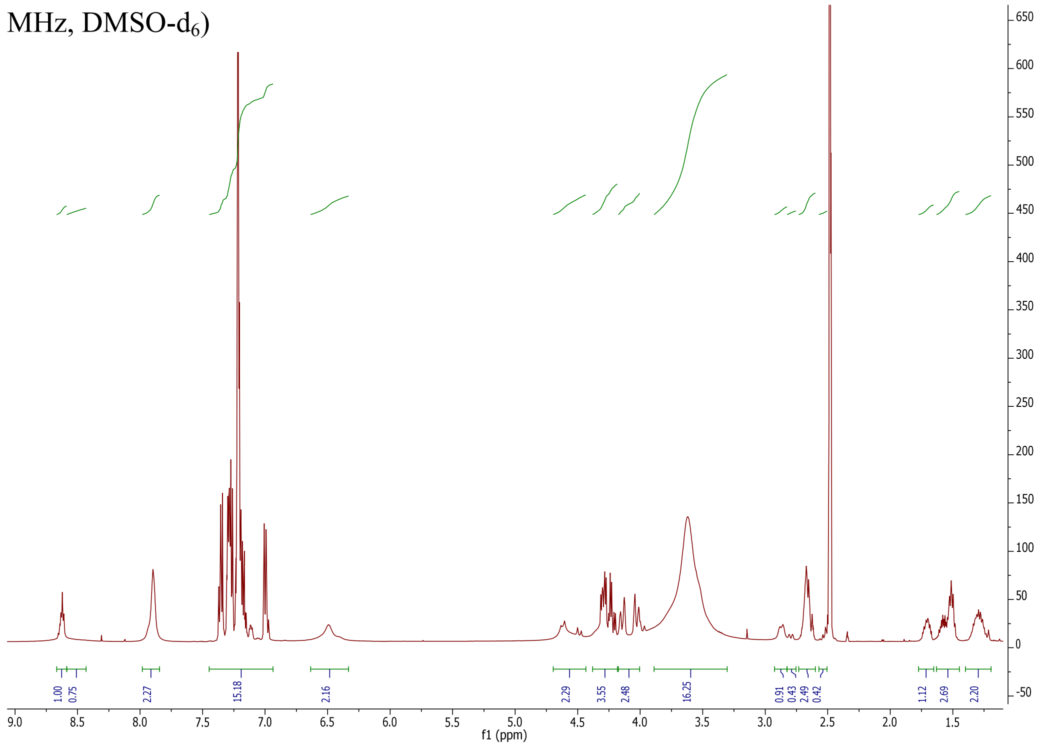

20b (125 MHz, DMSO-d$_6$)

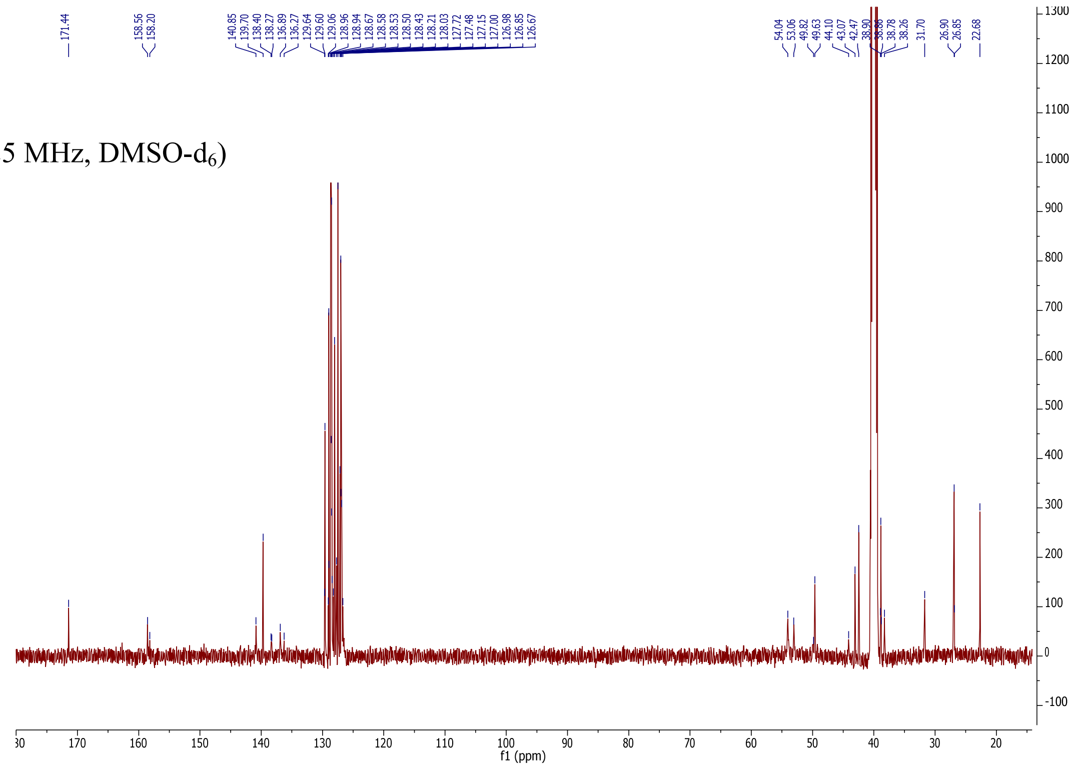

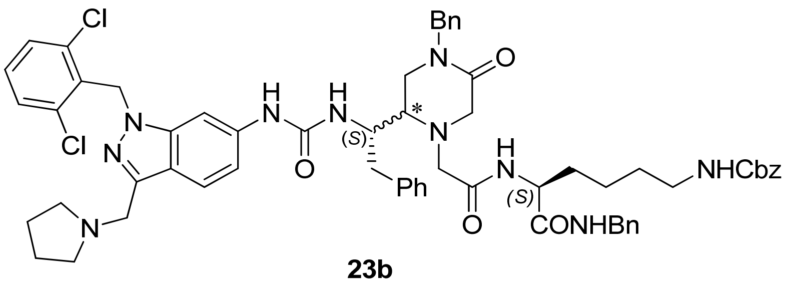

23b

23b (500 MHz, $CDCl_3$)

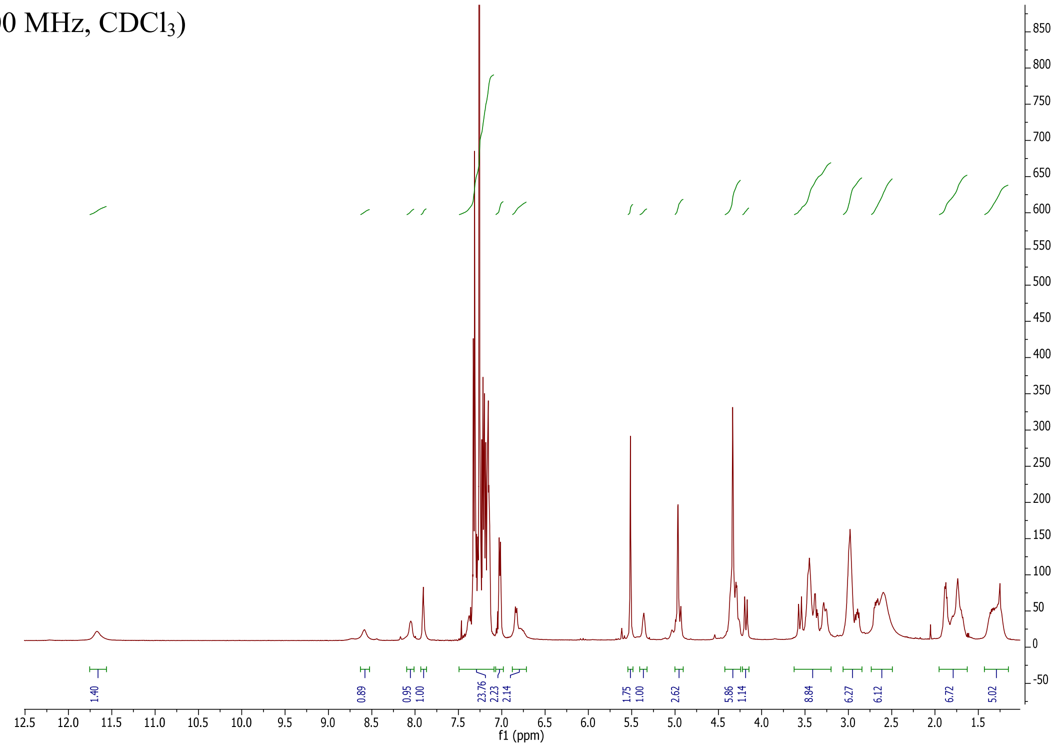

23b (125 MHz, $CDCl_3$)

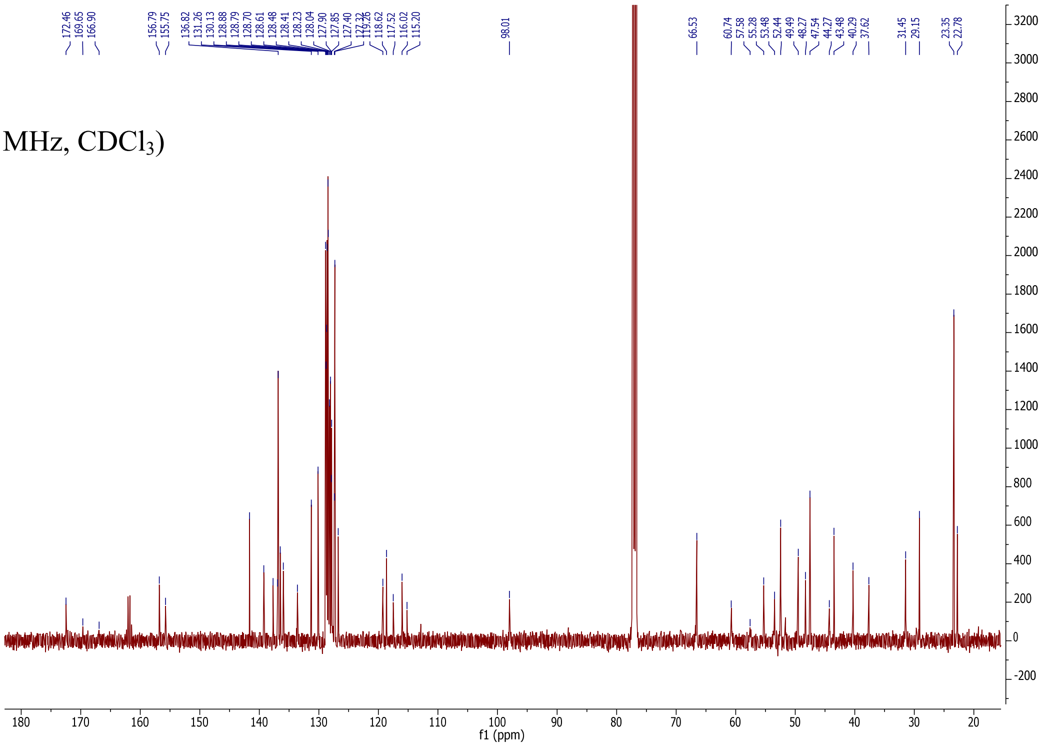

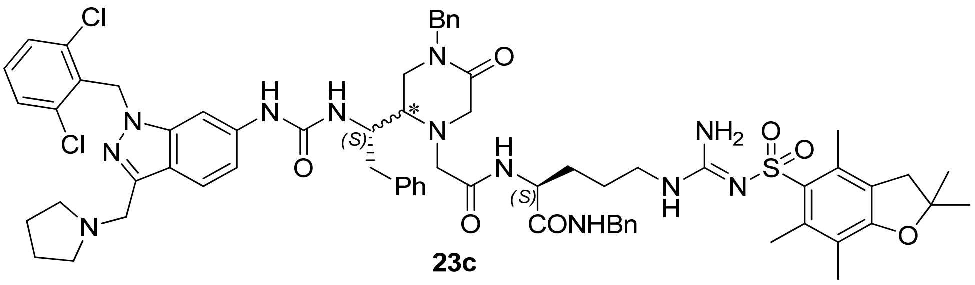

23c

23c (500 MHz, $(CD_3)_2CO$)

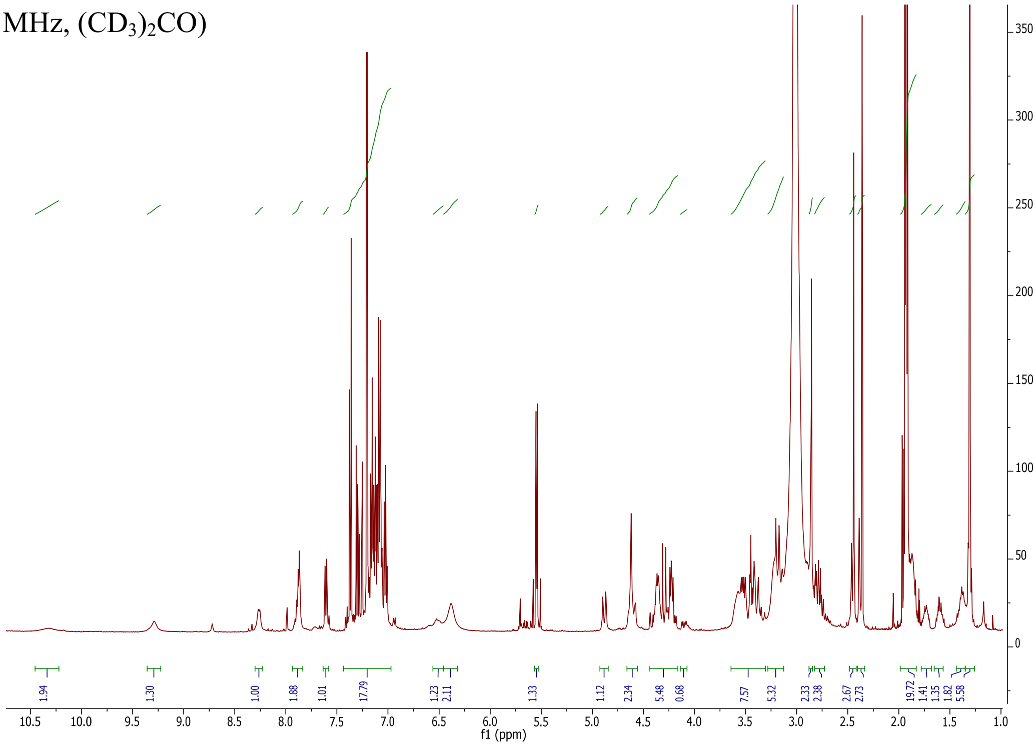

23c (125 MHz, $(CD_3)_2CO$)

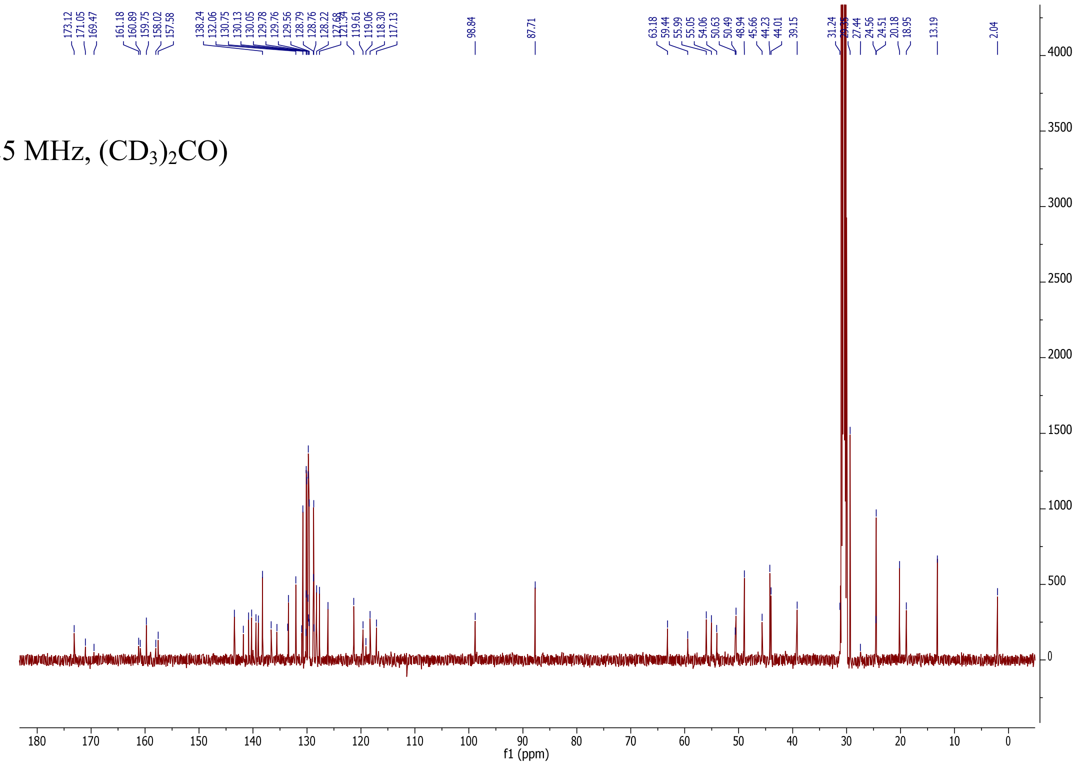

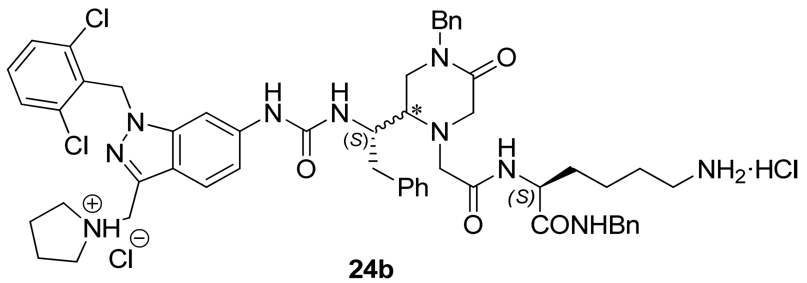

24b

24b (500 MHz, DMSO-d$_6$)

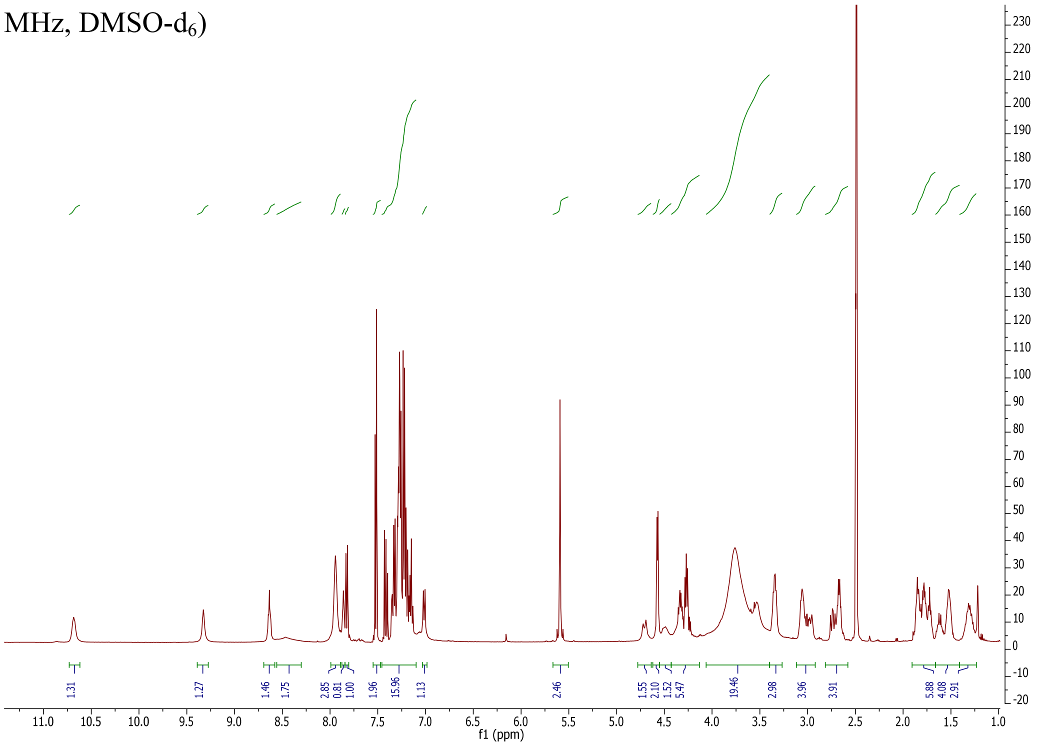

24b (125 MHz, DMSO-d$_6$)

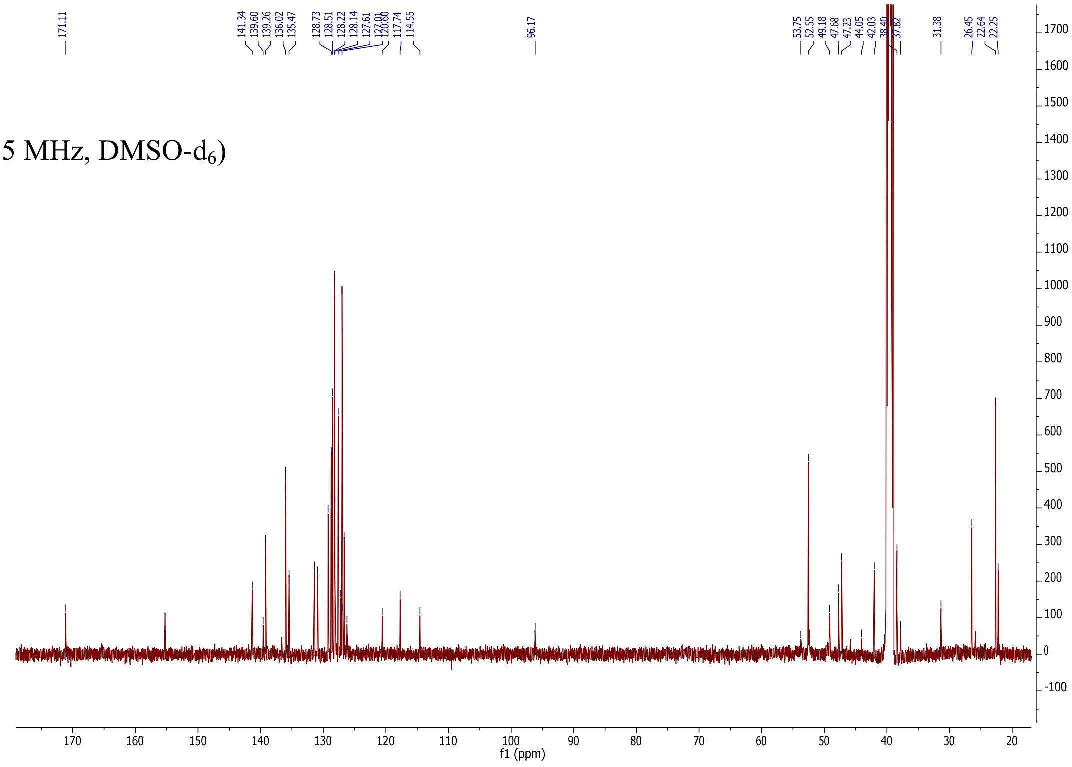

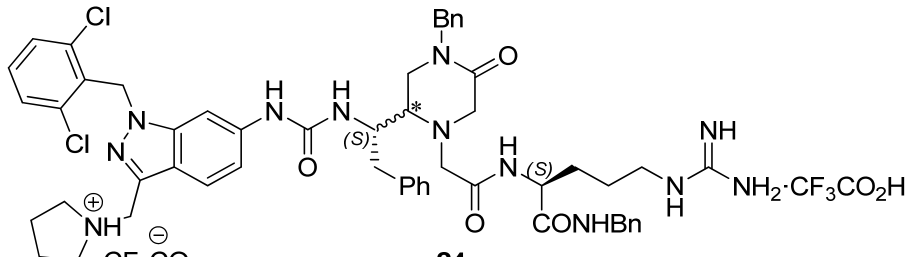

24c

24c (500 MHz, DMSO-d$_6$)

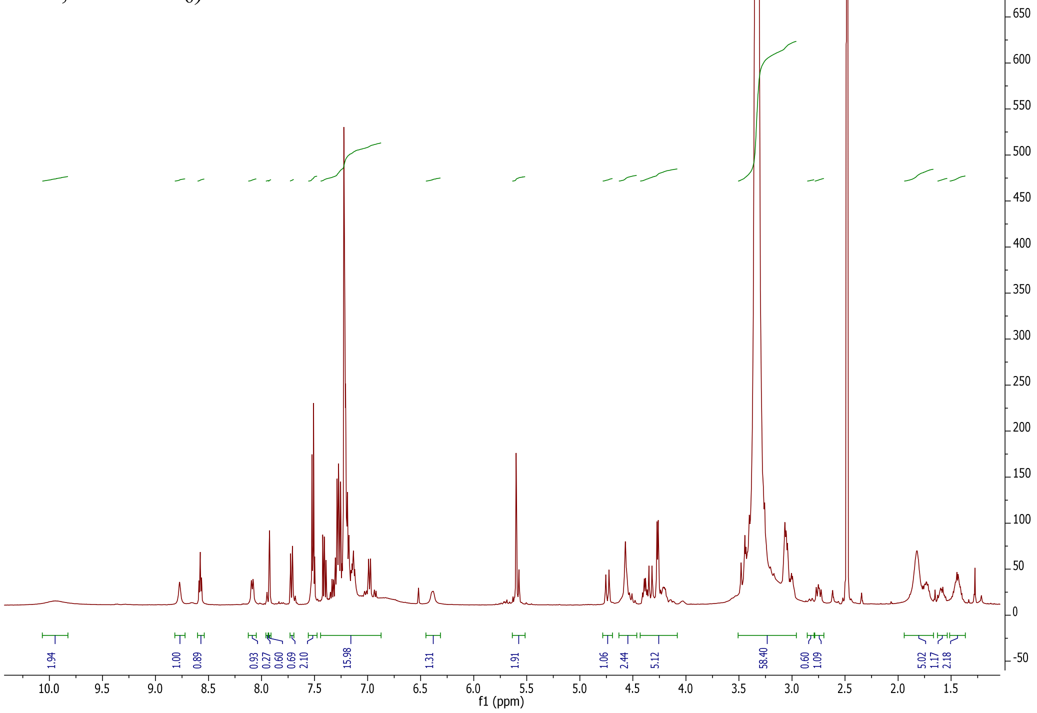

24c (125 MHz, DMSO-d$_6$)

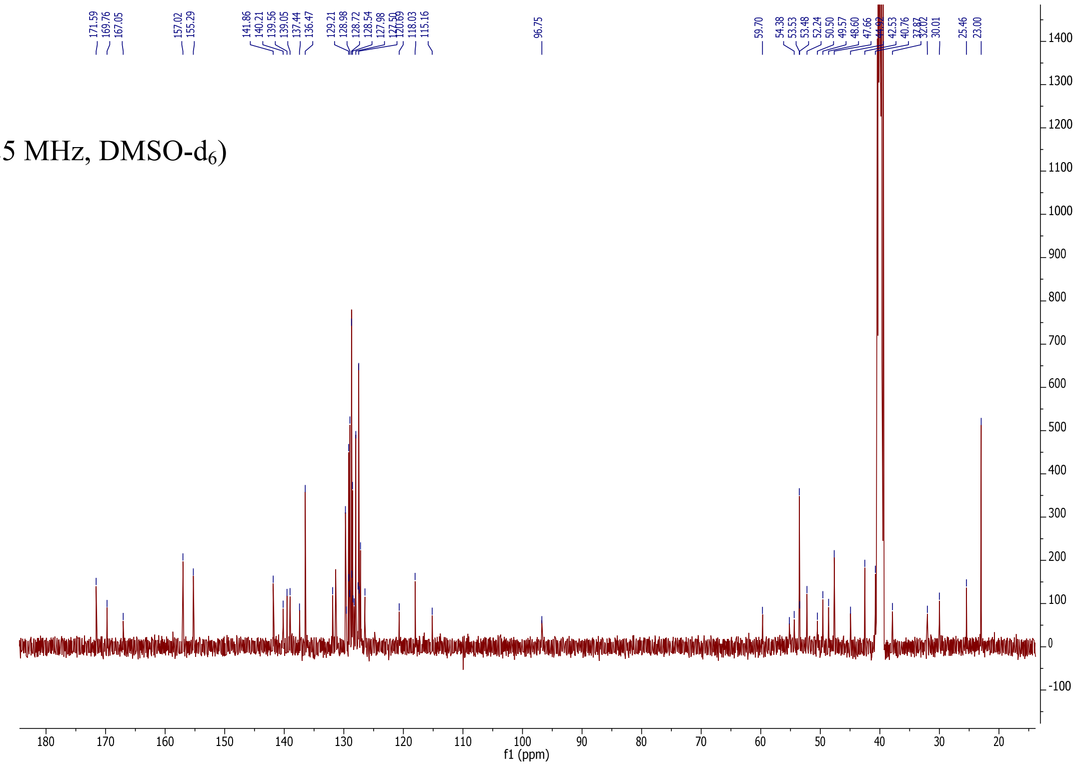

Supplement: Supplementary file 1 [file molecules-19-04814-s001.pdf]
